# Supplementary material for: An integrative taxonomic revision of slug-eating snakes (Squamata: Pareidae: Pareineae) reveals unprecedented diversity in Indochina
Source: PeerJ. 2022 Jan 10;10:e12713. doi: 10.7717/peerj.12713 (PMC8757378; doi:10.7717/peerj.12713)
Supplement: Supplemental Information 1 — Asterisk (*) denotes sequences that were included in the alignment for timetree calibration. [file peerj-10-12713-s001.docx]

**Supplementary Table S1.** Museum voucher information, geographic localities, and GenBank accession numbers of specimens and sequences used in the molecular analyses of this study.

Asterisk (*) denotes sequences that were included in the alignment for timetree calibration.

| **No.** | **Specimen ID** | **Species** | **Locality** | **Coordinates** | ***cyt b*** | ***ND4*** | ***cmos*** | ***RAG1*** |
| --- | --- | --- | --- | --- | --- | --- | --- | --- |
| **1** | NMNS 05618* | *Pareas komaii* | Taiwan, Taitung, Lijia | 22.78 N 121.03 E | KJ642185 | MW287056 | KJ642210 | — |
| **2** | NMNS 05625* | *Pareas komaii* | Taiwan, Hualien | 23.97 N 121.52 E | MZ712215 | MZ712240 | MZ712269 | — |
| **3** | NMNS 05655* | *Pareas iwasakii* | Japan, Okinawa, Ishigaki | 24.42 N 124.17 E | KJ642160 | — | KJ642198 | — |
| **4** | NMNS 05654* | *Pareas iwasakii* | Japan, Okinawa, Iriomote | 24.30 N 123.84 E | MZ712216 | — | MZ712270 | — |
| **5** | NMNS 05594* | *Pareas atayal* | Taiwan, Taoyuan, Beiheng | 24.77 N 121.35 E | KJ642124 | MW287041 | KJ642198 | — |
| **6** | CAS 235254* | *Pareas victorianus* | Myanmar, Chin, Nat Ma Taung N.P. | 21.24 N 93.89 E | MW438300 | MW438302 | MW438308 | MZ712301 |
| **7** | KIZ 014167* | *Pareas monticola* | China, Tibet (Xizang), Motuo | 29.32 N 95.34 E | MK135109 | MK805374 | MK135158 | MK805424 |
| **8** | ZMMU R-16631* | *Pareas monticola* | Myanmar, Sagaing, Ban Mauk | 24.51 N 95.81 E | MW438296 | MW438301 | MW438304 | MZ712302 |
| **9** | CAS235359* | *Pareas andersonii* | Myanmar, Chin, Nat Ma Taung N.P. | 21.24 N 93.89 E | MT968772 | MW287040 | MW287022 | MZ712303 |
| **10** | ZMMU R-16628* | *Pareas macularius* | Laos, Xaisomboun, Long Tien | 19.09 N 102.94 E | MT968770 | MZ712241 | MZ712271 | MZ712306 |
| **11** | ZMMU R-16629* | *Pareas macularius* | Myanmar, Sagaing, Ban Mauk | 24.51 N 95.81 E | MT968771 | MW287057 | MW287037 | MZ712307 |
| **12** | MZMU1293* | *Pareas modestus* | India, Mizoram, Aizawl, Tanhril | 23.74 N 92.67 E | MT968773 | — | — | — |
| **13** | CIB 098271* | *Pareas margaritophorus* | China, Hainan | 18.95 N 109.35 E | MK201376 | MZ712242 | MZ712272 | MK194440 |
| **14** | ZMMU R-13451* | *Pareas margaritophorus* | Vietnam, Binh Puoc, Bu Gia Map N.P. | 12.18 N 107.20 E | KJ642195 | MW287058 | MW287038 | MZ712304 |
| **15** | ZMMU NAP-09759* | *Pareas margaritophorus* | Thailand, Ratchaburi, Suan Phueng | 13.56 N 99.19 E | MZ712217 | MZ712243 | MZ712273 | MZ712305 |
| **16** | KIZ 09966* | *Pareas boulengeri* | China, Hubei, Jiannan | 30.43 N 108.53 E | JF827678 | JF827656 | MK135141 | MK805409 |
| **17** | CIB 010140* | *Pareas chinensis* | China, Sichuan, Tianquan | 30.07 N 102.74 E | JF827691 | JF827669 | MK135137 | MK805405 |
| **18** | HM 2007-S001* | *Pareas stanleyi* | China, Guangxi, Guilin | 25.23 N 110.22 E | JN230704 | JN230705 | MK135135 | MK805403 |
| **19** | CAS 248147* | *Pareas vindumi* | Myanmar, Kachin, Lukpwi | 25.47 N 99.29 E | MW287080 | MW287059 | MW287039 | MZ712308 |
| **20** | CHS 656* | *Pareas nigriceps* | China, Yunnan, Gaoligongshan N.R. | 24.83 N 98.76 E | MK201455 | — | — | — |
| **21** | BNHS 3575* | *Pareas kaduri* | India, Arunachal Pradesh, Lohit, Kamlang W.S. | 23.97 N 121.52 E | MT188734 | — | — | — |
| **22** | BNHS 3574* | *Pareas kaduri* | India, Arunachal Pradesh, Lohit, Kamlang W.S. | 27.91 N 96.18 E | MW026190 | — | — | — |
| **23** | ZMMU NAP-09088* | *Pareas hamptoni* | Vietnam, Lao Cai, Bat Xat | 22.62 N 103.64 E | MW287079 | MW287053 | MW287036 | MZ712309 |
| **24** | YPX 18219* | *Pareas hamptoni* | Myanmar, Kachin | 22.92 N 96.50 E | MK135077 | MK805342 | MK135126 | MK805394 |
| **25** | ZMMU R-16478* | *Pareas geminatus* 1 | Thailand, Chiang Mai | 18.94 N 99.07 E | MW287074 | MW287050 | MW287031 | MZ712310 |
| **26** | ZMMU NAP-09280* | *Pareas geminatus* 2 | Laos, Xaisomboun, Long Tien | 19.09 N 102.94 E | MW287073 | MW287049 | MW287030 | MZ712311 |
| **27** | KIZ-XL1* | *Pareas xuelinensis* | China, Yunnan, Lancang, Xuelin | 23.02 N 99.56 E | MW436709 | — | — | — |
| **28** | NMNS 05637* | *Pareas formosensis* 1 | Taiwan, Nantou | 23.98 N 121.00 E | MW287060 | MW287042 | MW287023 | MZ712314 |
| **29** | YBU 12015* | *Pareas formosensis* 2 | China, Hainan | 18.95 N 109.35 E | MK135068 | MK805333 | MK135117 | MK805390 |
| **30** | H26H26-HAM01* | *Pareas formosensis* 3 | China, Guangdong | 23.18 N 112.51 E | MW287061 | MW287043 | MW287024 | MK805393 |
| **31** | ZMMU NAP-07265* | *Pareas formosensis* 3 | Vietnam, Cao Bang, Phia Oac N.P. | 22.61 N 105.88 E | MW287062 | MW287044 | MW287025 | MZ712313 |
| **32** | ZMMU R-10255* | *Pareas formosensis* 4 (= *P. tonkinensis*) | Vietnam, Vinh Phuc, Tam Dao N.P. | 19.10 N 104.35 E | AY425806 | — | MZ712274 | MZ712312 |
| **33** | ZMMU NAP-08868* | *Pareas formosensis* 5 | Vietnam, Quang Nam, Song Thanh N.P. | 15.54 N 107.38 E | MW287063 | MW287045 | MW287026 | MZ712315 |
| **34** | ZMMU R-13709* | *Pareas formosensis* 5 | Vietnam, Lam Dong, Bidoup - Nui Ba N.P. | 12.10 N 108.65 E | MW287064 | MW287046 | MW287027 | MZ712317 |
| **35** | ZMMU R-14072* | *Pareas formosensis* 5 | Vietnam, Dak Lak, Chu Yang Sin N.P. | 12.37 N 108.35 E | MW287065 | MW287047 | MW287028 | MZ712316 |
| **36** | ZMMU R-16333* | *Pareas formosensis* 5 | Vietnam, Gia Lai, Kon Chu Rang N.R. | 14.50 N 108.54 E | MW287066 | MW287048 | MW287029 | MZ712318 |
| **37** | YBU 14288* | *Pareas mengziensis* | China, Yunnan, Mengzi | 23.39 N 103.36 E | MK135079 | MK805348 | MK135132 | MK805400 |
| **38** | KIZ 059339* | *Pareas niger* | China, Yunnan, Kunming | 24.88 N 102.90 E | MW436706 | — | — | — |
| **39** | CIB 098270 | *Pareas menglaensis* | China, Yunnan, Mengla (Loc. 22) | 21.45 N 101.51 E | JF827676 | JF827652 | MZ712275 | — |
| **40** | GP 1292* | *Pareas menglaensis* | China, Yunnan, Mengla (Loc. 22) | 21.45 N 101.51 E | MK135113 | MK805378 | MK135162 | MK805430 |
| **41** | YBU 14124 | *Pareas menglaensis* | China, Yunnan, Mengla (Loc. 22) | 21.45 N 101.51 E | MK135114 | MK805379 | MK135163 | MK805431 |
| **42** | YBU 14141 | *Pareas menglaensis* | China, Yunnan, Mengla (Loc. 22) | 21.45 N 101.51 E | MK135115 | MK805380 | MK135164 | MK805432 |
| **43** | YBU 14142 | *Pareas menglaensis* | China, Yunnan, Mengla (Loc. 22) | 21.45 N 101.51 E | MK135116 | MK805381 | MK135165 | MK805433 |
| **44** | AUP 01573* | *Pareas berdmorei berdmorei* | Thailand, Chiang Mai (Loc. 18) | 18.52 N 98.49 E | MZ712218 | MZ712244 | MZ712276 | MZ712319 |
| **45** | CAS 240362* | *Pareas berdmorei berdmorei* | Myanmar, Mon, Kin Pon Chaung (Loc. 17) | 17.41 N 97.08 E | MZ712219 | MZ712245 | MZ712277 | MZ712320 |
| **46** | ZMMU R-16803 | *Pareas berdmorei berdmorei* | Thailand, Ratchaburi, Suan Phueng (Loc. 14) | 13.56 N 99.19 E | MZ712220 | MZ712246 | MZ712278 | MZ712321 |
| **47** | ZMMU R-16801 | *Pareas berdmorei truongsonicus* **ssp. nov.** | Laos, Khammouan, Nahin (Loc. 27) | 17.47 N 104.85 E | MZ712221 | MZ712247 | MZ712288 | MZ712323 |
| **48** | ZMMU R-14796* | *Pareas berdmorei truongsonicus* **ssp. nov.** | Vietnam, Quang Binh, Thanh Thach (Loc. 28) | 18.00 N 105.90 E | MZ712222 | MZ712248 | MZ712289 | MZ712322 |
| **49** | ZMMU R-13753-1 | *Pareas berdmorei unicolor* | Vietnam, Dong Nai, Ma Da N.R. (Loc. 32) | 11.21 N 107.03 E | MZ712223 | MZ712249 | MZ712279 | MZ712324 |
| **50** | ZMMU R-13753-2* | *Pareas berdmorei unicolor* | Vietnam, Dong Nai, Ma Da N.R. (Loc. 32) | 11.21 N 107.03 E | MZ712224 | MZ712250 | MZ712280 | MZ712325 |
| **51** | ZMMU R-14013 | *Pareas berdmorei unicolor* | Vietnam, Dong Nai, Ma Da (Loc. 32) | 11.21 N 107.03 E | MZ712225 | MZ712251 | MZ712281 | MZ712326 |
| **52** | ZMMU R-14263* | *Pareas berdmorei unicolor* | Vietnam, Tay Ninh, Lo Go - Xa Mat N.P. (Loc. 34) | 11.60 N 105.89 E | MZ712226 | MZ712252 | MZ712282 | MZ712327 |
| **53** | ZMMU R-14421 | *Pareas berdmorei unicolor* | Vietnam, Lam Dong, Loc Bao (Loc. 36) | 11.83 N 107.66 E | MZ712227 | MZ712253 | MZ712283 | MZ712328 |
| **54** | SIEZC 20216* | *Pareas berdmorei unicolor* | Vietnam, Lam Dong, Di Linh (Loc. 37) | 11.43 N 108.06 E | MZ712228 | MZ712254 | MZ712284 | MZ712329 |
| **55** | ZMMU R-13679-1 | *Pareas berdmorei unicolor* | Vietnam, Dong Nai, Cat Tien N.P. (Loc. 33) | 11.42 N 107.42 E | MZ712229 | MZ712255 | MZ712285 | MZ712330 |
| **56** | ZMMU R-13679-2* | *Pareas berdmorei unicolor* | Vietnam, Dong Nai, Cat Tien N.P. (Loc. 33) | 11.42 N 107.42 E | MZ712230 | MZ712256 | MZ712286 | MZ712331 |
| **57** | ZMMU R-14165* | *Pareas berdmorei unicolor* | Vietnam, Binh Puoc, Bu Gia Map N.P. (Loc. 35) | 12.18 N 107.20 E | MZ712231 | MZ712257 | MZ712287 | MZ712332 |
| **58** | ZMMU R-16802* | *Pareas kuznetsovorum* **sp. nov.** | Vietnam, Phu Yen, Song Hinh (Loc. 41) | 12.77 N 109.05 E | MZ712232 | MZ712258 | MZ712296 | MZ712335 |
| **59** | CAS 247982* | *Pareas carinatus tenasserimicus* **ssp. nov.** | Myanmar, Tanintharyi, Yaephyu (Loc. 15) | 14.68 N 98.32 E | MZ712233 | MZ712259 | MZ712291 | MZ712334 |
| **60** | ZMMU R-16800* | *Pareas carinatus tenasserimicus* **ssp. nov.** | Thailand, Ratchaburi, Suan Phueng (Loc. 14) | 13.56 N 99.19 E | MZ712234 | MZ712260 | MZ712290 | MZ712333 |
| **61** | LSUHC 10604* | *Pareas carinatus carinatus* | Malaysia, Kedah, Sungai Sedim (Loc. 13) | 5.48 N 100.70 E | KC916748 | MZ712261 | MZ712292 | MZ712336 |
| **62** | DL 2008-S039 | *Pareas carinatus carinatus* | Malaysia (peninsular) | - | JF827677 | JF827653 | MK135162 | — |
| **63** | GP 1079 | *Pareas carinatus carinatus* | Malaysia (peninsular) | - | MK135110 | MK805375 | MK135159 | MK805427 |
| **64** | KIZ 011972* | *Pareas carinatus carinatus* | Malaysia (peninsular) | - | MK135111 | MK805376 | MK135160 | MK805428 |
| **65** | KIZ 011970 | *Pareas carinatus carinatus* | Malaysia (peninsular) | - | MK135112 | MK805377 | MK135161 | MK805429 |
| **66** | ZMMU R-16393* | *Pareas abros* **sp. nov.** | Vietnam, Quang Nam, Song Thanh N.P. (Loc. 42) | 15.54 N 107.38 E | MZ712235 | MZ712262 | MZ712293 | MZ712337 |
| **67** | ZMMU R-16392 | *Pareas abros* **sp. nov.** | Vietnam, Thua Thien-Hue, A Roang (Loc. 43) | 16.10 N 107.44 E | MZ712236 | MZ712263 | MZ712294 | MZ712338 |
| **68** | ZMMU R-14788* | *Pareas abros* **sp. nov.** | Vietnam, Thua Thien-Hue, A Roang (Loc. 43) | 16.10 N 107.44 E | MZ712237 | MZ712264 | MZ712295 | MZ712339 |
| **69** | ZMMU R-13656* | *Pareas temporalis* | Vietnam, Lam Dong, Cat Loc (Loc. 38) | 11.69 N 107.31 E | MZ712238 | MZ712265 | MZ712297 | MZ712340 |
| **70** | SIEZC 20215 | *Pareas temporalis* | Vietnam, Lam Dong, Di Linh (Loc. 37) | 11.43 N 108.06 E | MZ712239 | MZ712266 | MZ712298 | MZ712341 |
| **71** | UNS 09992 | *Pareas temporalis* | Vietnam, Lam Dong, Da Huoai (Loc. 39) | 11.34 N 107.62 E | MZ603793 | MZ603792 | — | — |
| **72** | FK 2626* | *Pareas nuchalis* | Brunei, Brunei Darussalam, Belait (Loc. 47) | 4.37 N 114.62 E | — | U49311 | — | — |
| **73** | LSUHC 7248* | *Aplopeltura boa* | Malaysia, Sabah, Sepilok | 5.87 N 117.94 E | KC916746 | U49312 | AF544715 | — |
| **74** | KIZ 011963* | *Aplopeltura boa* | Malaysia (peninsular) | - | JF827673 | JF827650 | JF827696 | — |
| **75** | FMNH 241296* | *Asthenodipsas laevis* | Malaysia, Penang, Pulau Pinang | 5.42 N 100.26 E | KX660468 | KX660596 | KX660335 | — |
| **76** | —* | *Asthenodipsas tropidonota* | Indonesia, Sumatra | - | AY425808 | — | — | — |
| **77** | LSUHC 9098* | *Asthenodipsas lasgalenensis* | Malaysia, Pahang, Fraser’s Hill | 3.71 N 101.73 E | KC916755 | MZ712267 | MZ712299 | MZ712342 |
| **78** | —* | *Asthenodipsas vertebralis* | Malaysia, Perak, Bukit Larut | 4.86 N 100.78 E | KC916750 | MZ712268 | MZ712300 | MZ712343 |
| **79** | FMNH 273617* | *Asthenodipsas borneensis* | Malaysia, Sarawak, Kelabit Highlands, Bario | 4.22 N 114.35 E | KX660469 | KX660597 | KX660336 | — |
| **80** | BNHS 3376* | *Xylophis captaini* | India | - | MK340914 | MK340912 | MK344195 | — |
| **81** | BNHS 3582* | *Xylophis perroteti* | India | - | MN970042 | MN970046 | MN970049 | MK340913 |
