# Supplementary material for: An integrative taxonomic revision of slug-eating snakes (Squamata: Pareidae: Pareineae) reveals unprecedented diversity in Indochina
Source: PeerJ. 2022 Jan 10;10:e12713. doi: 10.7717/peerj.12713 (PMC8757378; doi:10.7717/peerj.12713)
Supplement: Supplemental Information 2 — “F” – forward primer, “R” – reverse primer. [file peerj-10-12713-s002.docx]

**Supplementary Table S2. Primers used for DNA amplification and sequencing.**

“F” – forward primer, “R” – reverse primer.

| **Gene** | **Primer name** | **Direction** | **Primer sequence (5'—3')** | **Reference** |
| --- | --- | --- | --- | --- |
| cyt *b* | L14910 | F | 5ʹ-GACCTGTGATMTGAAAAACCAYCGTTGT-3ʹ | *de Queiroz et al., 2002* |
| cyt *b* | H16064 | R | 5ʹ-CTTTGGTTTACAAGAACAATGCTTTA-3ʹ | *de Queiroz et al., 2002* |
| *ND4* | ND4F | F | 5ʹ-CACCTATGACTACCAAAAGCTCATGTAGAAGC-3ʹ | *Salvi et al., 2013* |
| *ND4* | ND4LEUR | R | 5ʹ-CATTACTTTTACTTGGATTTGCACCA-3ʹ | *Salvi et al., 2013* |
| *c-mos* | cmos S77 | F | 5ʹ-CATGGACTGGGATCAGTTATG-3ʹ | *Slowinski & Lawson, 2002* |
| *c-mos* | cmos S78 | R | 5ʹ-CCTTGGGTGTGATTTTCTCACCT-3ʹ | *Slowinski & Lawson, 2002* |
| *RAG1* | RAG1_MartFL1 | F | 5ʹ-AGCTGCAGYCARTAYCAYAARATGTA-3ʹ | *Chiari et al., 2004* |
| *RAG1* | RAG1_AmpR1 | R | 5ʹ-AACTCAGCTGCATTKCCAATRTCA-3ʹ | *Chiari et al., 2004* |
| *RAG1* | RepRAG1-R13 | F | 5ʹ-TCTGAATGGAAATTCAAGCTGTT-3ʹ | *Groth & Barrowclough, 1999* |
| *RAG1* | RepRAG1-R18 | R | 5ʹ-GATGCTGCCTCGGTCGGCCACCTTT-3ʹ | *Groth & Barrowclough, 1999* |
