# Supplementary material for: An integrative taxonomic revision of slug-eating snakes (Squamata: Pareidae: Pareineae) reveals unprecedented diversity in Indochina
Source: PeerJ. 2022 Jan 10;10:e12713. doi: 10.7717/peerj.12713 (PMC8757378; doi:10.7717/peerj.12713)
Supplement: Supplemental Information 3 — Total length (in b.p.), number of conservative (Cons.), variable (Var.) and parsimony-informative (Pars.-Inf.) sites are given (data presented only for the ingroup). The optimal partitioning scheme and model fit was estimated as suggested by the Akaike information criterion (AIC). [file peerj-10-12713-s003.docx]

**Supplementary Table S3. Characteristics of analyzed mtDNA and nuDNA sequences and the proposed optimal evolutionary models for gene and codon partitions as estimated in PartitionFinder v1.0.1.**

Total length (in b.p.), number of conservative (Cons.), variable (Var.) and parsimony-informative (Pars.-Inf.) sites are given (data presented only for the ingroup). The optimal partitioning scheme and model fit was estimated as suggested by the Akaike information criterion (AIC).

| **Genetic marker** | | **Sites (in b.p.)** | | | | **Substitution Model** | |
| --- | --- | --- | --- | --- | --- | --- | --- |
|  |  | **Cons.** | **Var.** | **Pars.-Inf.** | **Total** | **Codon partition** | **Model** |
| **1** | cyt *b* | 443 | 683 | 601 | 1126 | cyt *b* – 1 | GTR+I+G |
|  |  |  |  |  |  | cyt *b* – 2 | GTR+G |
|  |  |  |  |  |  | cyt *b* – 3 | GTR+I+G |
| **2** | *ND4* | 285 | 390 | 345 | 678 | *ND4* – 1 | HKY+G |
|  |  |  |  |  |  | *ND4* – 2 | HKY+G |
|  |  |  |  |  |  | *ND4* – 3 | GTR+G |
| **3** | *c-mos* | 624 | 110 | 78 | 734 | *cmos* – 1 | K2P |
|  |  |  |  |  |  | *cmos* – 2 | K2P+I |
|  |  |  |  |  |  | *cmos* – 3 | GTR+G |
| **4** | *RAG1* | 904 | 119 | 76 | 1023 | *RAG1* – 1 | HKY |
|  |  |  |  |  |  | *RAG1* – 2 | HKY |
|  |  |  |  |  |  | *RAG1* – 3 | HKY+I+G |
