# Supplementary material for: An integrative taxonomic revision of slug-eating snakes (Squamata: Pareidae: Pareineae) reveals unprecedented diversity in Indochina
Source: PeerJ. 2022 Jan 10;10:e12713. doi: 10.7717/peerj.12713 (PMC8757378; doi:10.7717/peerj.12713)
Supplement: Supplemental Information 4 — Node – tree node used for calibration, for node names see Supplementary Figure S1; divergence time given in millions years (mya). [file peerj-10-12713-s004.docx]

**Supplementary Table S4. Calibration points for divergence time estimates.**

Node – tree node used for calibration, for node names see Supplementary Figure S1; divergence time given in millions years (mya).

| **Node** | **Cladogenetic event** | **Estimated date (mya)** | **HPD (mya)** | **Prior distribution** | **Reference** |
| --- | --- | --- | --- | --- | --- |
| **1** | Split between *Xylophis* and Pareinae | 44.9 | 38.9 – 51.2 | normal | *Deepak et al., 2019* |
| **3** | Split between *Asthenodipsas* and (*Aplopeltura + Pareas*) | 38.0 | 31.1 – 44.6 | normal | *Deepak et al., 2019* |
| **8** | Split between *Aplopeltura* and *Pareas* | 28.4 | 21.7 – 35.7 | normal | *Deepak et al., 2019* |
| **2** | Split between *Xylophis captaini* and *X. perroteti* | 32.5 | 24.4 – 40.8 | normal | *Deepak et al., 2019* |
