# Supplementary material for: An integrative taxonomic revision of slug-eating snakes (Squamata: Pareidae: Pareineae) reveals unprecedented diversity in Indochina
Source: PeerJ. 2022 Jan 10;10:e12713. doi: 10.7717/peerj.12713 (PMC8757378; doi:10.7717/peerj.12713)
Supplement: Supplemental Information 5 — Geographic regions: (A) Mainland East Asia; (B) Eastern Indochina; (C) Western Indochina; (D) East Himalaya and Indoburma; (E) Sundaland; (F) East Asian Islands; see Fig. 3. No. corresponds to specimen number in Table S1. [file peerj-10-12713-s005.docx]

**Supplementary Table S5.** Matrix of modern species distribution within the subfamily Pareinae.

Geographic regions: (A) Mainland East Asia; (B) Eastern Indochina; (C) Western Indochina; (D) East Himalaya and Indoburma; (E) Sundaland; (F) East Asian Islands; see Fig. 3. No. corresponds to specimen number in Supplementary Table S1.

| **No.** | **Specimen ID** | **Species** | **Territory** |
| --- | --- | --- | --- |
| **1** | NMNS 05618 | *Pareas komaii* | F |
| **2** | NMNS 05625 | *Pareas komaii* | F |
| **3** | NMNS 05655 | *Pareas iwasakii* | F |
| **4** | NMNS 05654 | *Pareas iwasakii* | F |
| **5** | NMNS 05594 | *Pareas atayal* | F |
| **6** | CAS 235254 | *Pareas victorianus* | D |
| **7** | SYN U04(II)149 | *Pareas monticola* | D |
| **8** | ZMMU R-16631 | *Pareas monticola* | D |
| **9** | CAS235359 | *Pareas andersonii* | D |
| **10** | ZMMU R-16628 | *Pareas macularius* | B |
| **11** | ZMMU R-16629 | *Pareas macularius* | D |
| **12** | MZMU1293 | *Pareas modestus* | D |
| **13** | CIB 098271 | *Pareas margaritophorus* | A |
| **14** | ZMMU R-13451 | *Pareas margaritophorus* | B |
| **15** | ZMMU NAP-09759 | *Pareas margaritophorus* | C |
| **16** | KIZ 09966 | *Pareas boulengeri* | A |
| **17** | CIB 010140 | *Pareas chinensis* | A |
| **18** | HM 2007-S001 | *Pareas stanleyi* | A |
| **19** | CAS 248147 | *Pareas vindumi* | D |
| **20** | CHS 656 | *Pareas nigriceps* | D |
| **21** | BNHS 3575 | *Pareas kaduri* | D |
| **22** | BNHS 3574 | *Pareas kaduri* | D |
| **23** | ZMMU NAP-09088 | *Pareas hamptoni* | B |
| **24** | YPX 18219 | *Pareas hamptoni* | C |
| **25** | ZMMU R-16478 | *Pareas geminatus* 1 | C |
| **26** | ZMMU NAP-09280 | *Pareas geminatus* 2 | B |
| **27** | KIZ-XL1 | *Pareas xuelinensis* | C |
| **28** | NMNS 05637 | *Pareas formosensis* 1 | F |
| **29** | YBU 12015 | *Pareas formosensis* 2 | A |
| **30** | H26H26-HAM01 | *Pareas formosensis* 3 | A |
| **31** | ZMMU NAP-07265 | *Pareas formosensis* 3 | A |
| **32** | ZMMU R-10255 | *Pareas formosensis* 4 (= *P. tonkinensis*) | A |
| **33** | ZMMU NAP-08868 | *Pareas formosensis* 5 | B |
| **34** | ZMMU R-13709 | *Pareas formosensis* 5 | B |
| **35** | ZMMU R-14072 | *Pareas formosensis* 5 | B |
| **36** | ZMMU R-16333 | *Pareas formosensis* 5 | B |
| **37** | YBU 14288 | *Pareas mengziensis* | C |
| **38** | KIZ 059339 | *Pareas niger* | A |
| **40** | GP 1292 | *Pareas menglaensis* | B |
| **44** | AUP 01573 | *Pareas berdmorei berdmorei* | C |
| **45** | CAS 240362 | *Pareas berdmorei berdmorei* | C |
| **48** | ZMMU R-14796 | *Pareas berdmorei truongsonicus* **ssp. nov.** | B |
| **50** | ZMMU R-13753-2 | *Pareas berdmorei unicolor* | B |
| **52** | ZMMU R-14263 | *Pareas berdmorei unicolor* | B |
| **54** | SIEZC 20216 | *Pareas berdmorei unicolor* | B |
| **56** | ZMMU R-13679-2 | *Pareas berdmorei unicolor* | B |
| **57** | ZMMU R-14165 | *Pareas berdmorei unicolor* | B |
| **58** | ZMMU R-16802 | *Pareas kuznetsovorum* **sp. nov.** | B |
| **59** | CAS 247982 | *Pareas carinatus tenasserimicus* **ssp. nov.** | C |
| **60** | ZMMU R-16800 | *Pareas carinatus tenasserimicus* **ssp. nov.** | C |
| **61** | LSUHC 10604 | *Pareas carinatus carinatus* | E |
| **64** | KIZ 011972 | *Pareas carinatus carinatus* | E |
| **66** | ZMMU R-16393 | *Pareas abros* **sp. nov.** | B |
| **68** | ZMMU R-14788 | *Pareas abros* **sp. nov.** | B |
| **69** | ZMMU R-13656 | *Pareas temporalis* | B |
| **71** | FK 2626 | *Pareas nuchalis* | E |
| **72** | LSUHC 7248 | *Aplopeltura boa* | E |
| **73** | KIZ 011963 | *Aplopeltura boa* | E |
| **74** | FMNH 241296 | *Asthenodipsas laevis* | E |
| **75** | — | *Asthenodipsas tropidonota* | E |
| **76** | LSUHC 9098 | *Asthenodipsas lasgalenensis* | E |
| **77** | — | *Asthenodipsas vertebralis* | E |
| **78** | FMNH 273617 | *Asthenodipsas borneensis* | E |
