# Supplementary material for: An integrative taxonomic revision of slug-eating snakes (Squamata: Pareidae: Pareineae) reveals unprecedented diversity in Indochina
Source: PeerJ. 2022 Jan 10;10:e12713. doi: 10.7717/peerj.12713 (PMC8757378; doi:10.7717/peerj.12713)
Supplement: Supplemental Information 6 — Letters encode the geographic regions: (A) Mainland East Asia; (B) Eastern Indochina; (C) Western Indochina; (D) East Himalaya and Indoburma; (E) Sundaland; (F) East Asian Islands; see Fig. 3. [file peerj-10-12713-s006.docx]

**Supplementary Table S6. Step-matrix showing dispersal constraints between biogeographic areas.**

Letters encode the geographic regions: (A) Mainland East Asia; (B) Eastern Indochina; (C) Western Indochina; (D) East Himalaya and Indoburma; (E) Sundaland; (F) East Asian Islands; see Fig. 3.

|  | **A** | **B** | **C** | **D** | **E** | **F** |
| --- | --- | --- | --- | --- | --- | --- |
| **A** |  | 1 | 0 | 1 | 0 | 1 |
| **B** | 1 |  | 1 | 1 | 1 | 0 |
| **C** | 0 | 1 |  | 1 | 1 | 0 |
| **D** | 1 | 1 | 1 |  | 0 | 0 |
| **E** | 0 | 1 | 1 | 0 |  | 0 |
| **F** | 1 | 0 | 0 | 0 | 0 |  |
