# Supplementary material for: An integrative taxonomic revision of slug-eating snakes (Squamata: Pareidae: Pareineae) reveals unprecedented diversity in Indochina
Source: PeerJ. 2022 Jan 10;10:e12713. doi: 10.7717/peerj.12713 (PMC8757378; doi:10.7717/peerj.12713)
Supplement: Supplemental Information 8 — Uncorrected average interspecific (below diagonal), intraspecific (on the diagonal) genetic p-distances and error estimates (above the diagonal) for ND4 mtDNA gene fragment (in percentage) are given for species of the genus Pareas (1–29). [file peerj-10-12713-s008.docx]

**Supplementary Table S8. Genetic divergence of the genus *Pareas* in *ND4* gene sequences.**

Uncorrected average interspecific (below diagonal), intraspecific (on the diagonal) genetic p-distances and error estimates (above the diagonal) for *ND4* mtDNA gene fragment (in percentage) are given for species of the genus *Pareas* (1–29).

|  | **Taxon** | **1** | **2** | **3** | **4** | **5** | **6** | **7** | **8** | **9** | **10** | **11** | **12** | **13** | **14** | **15** | **16** | **17** | **18** | **19** | **20** | **21** | **22** | **23** | **24** | **25** | **26** | **27** | **28** | **29** |
| --- | --- | --- | --- | --- | --- | --- | --- | --- | --- | --- | --- | --- | --- | --- | --- | --- | --- | --- | --- | --- | --- | --- | --- | --- | --- | --- | --- | --- | --- | --- |
| **1** | *P.b.unicolor* | **0.4** | 0.7 | 0.9 | 1.2 | 1.3 | 1.3 | 1.2 | 1.3 | 1.6 | 1.7 | — | 1.5 | 1.6 | 1.7 | — | 1.7 | 1.7 | 1.6 | — | — | 1.7 | 1.7 | 1.8 | 1.6 | — | 1.6 | 1.5 | 1.5 | 1.6 |
| **2** | *P.b.truongsonicus* **ssp.nov.** | 5.2 | **0.2** | 0.9 | 1.1 | 1.3 | 1.3 | 1.3 | 1.3 | 1.6 | 1.7 | — | 1.5 | 1.6 | 1.7 | — | 1.6 | 1.6 | 1.6 | — | — | 1.7 | 1.7 | 1.8 | 1.5 | — | 1.6 | 1.5 | 1.5 | 1.5 |
| **3** | *P.b.berdmorei* | 6.6 | 6.8 | **0.8** | 1.3 | 1.3 | 1.2 | 1.3 | 1.2 | 1.4 | 1.6 | — | 1.5 | 1.6 | 1.7 | — | 1.7 | 1.6 | 1.7 | — | — | 1.7 | 1.6 | 1.8 | 1.5 | — | 1.5 | 1.5 | 1.4 | 1.6 |
| **4** | *P.kuznetsovorum* **sp.nov.** | 14.2 | 13.8 | 13.9 | — | 1.4 | 1.2 | 1.7 | 1.5 | 1.6 | 1.5 | — | 1.5 | 1.5 | 1.7 | — | 1.5 | 1.6 | 1.7 | — | — | 1.6 | 1.7 | 1.7 | 1.7 | — | 1.6 | 1.5 | 1.5 | 1.6 |
| **5** | *P.c.carinatus* | 15.0 | 14.3 | 14.1 | 15.1 | **0.0** | 0.9 | 1.1 | 1.2 | 1.5 | 1.6 | — | 1.5 | 1.6 | 1.7 | — | 1.6 | 1.5 | 1.5 | — | — | 1.4 | 1.4 | 1.6 | 1.7 | — | 1.6 | 1.3 | 1.4 | 1.5 |
| **6** | *P.c.tenasserimicus* **ssp.nov.** | 14.1 | 14.0 | 13.1 | 14.0 | 5.7 | **4.2** | 1.2 | 1.3 | 1.4 | 1.6 | — | 1.5 | 1.6 | 1.6 | — | 1.5 | 1.4 | 1.6 | — | — | 1.5 | 1.5 | 1.7 | 1.6 | — | 1.6 | 1.3 | 1.5 | 1.6 |
| **7** | *P.abros* **sp.nov.** | 17.8 | 19.1 | 19.4 | 20.1 | 18.9 | 19.7 | **0.2** | 1.2 | 1.7 | 1.5 | — | 1.5 | 1.5 | 1.6 | — | 1.5 | 1.5 | 1.5 | — | — | 1.6 | 1.4 | 1.4 | 1.6 | — | 1.5 | 1.4 | 1.6 | 1.5 |
| **8** | *P.temporalis* | 18.6 | 19.5 | 19.0 | 19.0 | 19.2 | 18.6 | 9.6 | **0.0** | 1.6 | 1.4 | — | 1.5 | 1.5 | 1.5 | — | 1.4 | 1.4 | 1.5 | — | — | 1.6 | 1.5 | 1.4 | 1.5 | — | 1.5 | 1.3 | 1.5 | 1.5 |
| **9** | *P.nuchalis* | 18.0 | 18.2 | 17.9 | 19.6 | 19.0 | 18.4 | 17.5 | 17.4 | — | 1.7 | — | 1.7 | 1.7 | 1.6 | — | 1.9 | 1.8 | 1.6 | — | — | 1.8 | 1.7 | 1.6 | 1.4 | — | 1.7 | 1.4 | 1.6 | 1.8 |
| **10** | *P.formosensis* | 21.5 | 22.1 | 20.9 | 20.1 | 21.5 | 21.0 | 19.3 | 19.2 | 20.5 | **3.9** | — | 1.1 | 1.0 | 1.0 | — | 1.2 | 1.4 | 1.2 | — | — | 1.5 | 1.2 | 1.5 | 1.4 | — | 1.3 | 1.3 | 1.4 | 1.4 |
| **11** | *P.xuelinensis* | — | — | — | — | — | — | — | — | — | — | — | — | — | — | — | — | — | — | — | — | — | — | — | — | — | — | — | — | — |
| **12** | *P.geminatus* | 20.7 | 21.6 | 21.5 | 19.8 | 21.1 | 20.2 | 18.4 | 18.7 | 20.4 | 9.4 | — | **0.0** | 0.9 | 1.2 | — | 1.3 | 1.5 | 1.3 | — | — | 1.5 | 1.4 | 1.5 | 1.4 | — | 1.3 | 1.5 | 1.3 | 1.5 |
| **13** | *P.hamptoni* | 20.5 | 20.7 | 20.9 | 18.9 | 20.7 | 20.2 | 19.5 | 19.5 | 21.0 | 8.8 | — | 5.4 | **0.6** | 1.0 | — | 1.4 | 1.4 | 1.4 | — | — | 1.5 | 1.4 | 1.5 | 1.5 | — | 1.4 | 1.4 | 1.4 | 1.5 |
| **14** | *P.niger* | 22.9 | 22.7 | 22.0 | 21.3 | 21.3 | 20.6 | 20.0 | 18.1 | 21.3 | 8.9 | — | 10.1 | 9.0 | — | — | 1.3 | 1.3 | 1.3 | — | — | 1.4 | 1.3 | 1.5 | 1.6 | — | 1.3 | 1.3 | 1.4 | 1.5 |
| **15** | *P.iwasakii* | — | — | — | — | — | — | — | — | — | — | — | — | — | — | — | — | — | — | — | — | — | — | — | — | — | — | — | — | — |
| **16** | *P.atayal* | 21.1 | 21.6 | 21.8 | 21.1 | 21.1 | 20.9 | 22.3 | 19.9 | 23.4 | 16.4 | — | 16.0 | 16.3 | 16.0 | — | — | 1.0 | 1.2 | — | — | 1.4 | 1.5 | 1.4 | 1.5 | — | 1.2 | 1.3 | 1.3 | 1.6 |
| **17** | *P.komaii* | 22.3 | 23.0 | 22.3 | 21.3 | 21.6 | 21.3 | 22.0 | 19.6 | 23.7 | 16.4 | — | 17.2 | 17.4 | 16.1 | — | 7.5 | **0.0** | 1.4 | — | — | 1.5 | 1.5 | 1.4 | 1.6 | — | 1.3 | 1.2 | 1.5 | 1.6 |
| **18** | *P.vindumi* | 20.0 | 20.7 | 21.2 | 19.3 | 20.4 | 19.8 | 19.3 | 18.7 | 20.1 | 13.1 | — | 13.4 | 13.0 | 13.6 | — | 13.9 | 14.2 | — | — | — | 1.5 | 1.5 | 1.5 | 1.5 | — | 1.4 | 1.4 | 1.5 | 1.5 |
| **19** | *P.kaduri* | — | — | — | — | — | — | — | — | — | — | — | — | — | — | — | — | — | — | — | — | — | — | — | — | — | — | — | — | — |
| **20** | *P.nigriceps* | — | — | — | — | — | — | — | — | — | — | — | — | — | — | — | — | — | — | — | — | — | — | — | — | — | — | — | — | — |
| **21** | *P.boulengeri* | 21.4 | 20.7 | 22.6 | 19.9 | 21.0 | 21.6 | 21.2 | 20.2 | 22.3 | 19.3 | — | 18.1 | 18.0 | 17.7 | — | 18.4 | 19.0 | 15.5 | — | — | — | 1.2 | 1.5 | 1.6 | — | 1.5 | 1.4 | 1.3 | 1.4 |
| **22** | *P.chinensis* | 22.3 | 22.7 | 22.7 | 21.7 | 22.6 | 22.5 | 21.2 | 18.7 | 22.5 | 17.6 | — | 18.1 | 16.4 | 17.0 | — | 20.1 | 19.2 | 15.7 | — | — | 10.1 | — | 1.3 | 1.6 | — | 1.4 | 1.6 | 1.2 | 1.4 |
| **23** | *P.stanleyi* | 23.5 | 23.3 | 23.7 | 22.4 | 22.5 | 22.2 | 21.7 | 20.1 | 23.0 | 19.2 | — | 19.6 | 20.4 | 19.0 | — | 19.5 | 18.6 | 16.5 | — | — | 14.8 | 16.6 | — | 1.3 | — | 1.3 | 1.5 | 1.4 | 1.5 |
| **24** | *P.andersoni* | 20.4 | 20.7 | 21.1 | 21.5 | 23.0 | 22.5 | 20.4 | 20.0 | 19.8 | 19.6 | — | 18.0 | 19.1 | 18.9 | — | 19.1 | 19.7 | 16.2 | — | — | 18.0 | 18.3 | 18.0 | — | — | 1.1 | 1.1 | 1.5 | 1.5 |
| **25** | *P.modestus* | — | — | — | — | — | — | — | — | — | — | — | — | — | — | — | — | — | — | — | — | — | — | — | — | — | — | — | — | — |
| **26** | *P.macularius* | 23.0 | 22.9 | 22.5 | 22.7 | 23.1 | 23.4 | 22.3 | 21.8 | 22.8 | 20.5 | — | 19.7 | 20.1 | 20.0 | — | 19.6 | 20.4 | 17.9 | — | — | 18.9 | 19.8 | 18.1 | 12.0 | — | **10.4** | 1.1 | 1.4 | 1.4 |
| **27** | *P.margaritophorus* | 22.2 | 22.2 | 22.0 | 21.3 | 21.6 | 21.1 | 21.3 | 20.3 | 22.5 | 19.5 | — | 20.3 | 19.5 | 18.5 | — | 20.1 | 20.5 | 17.7 | — | — | 18.2 | 19.8 | 20.2 | 14.3 | — | 15.1 | **4.9** | 1.4 | 1.5 |
| **28** | *P.monticola* | 20.3 | 20.7 | 21.1 | 19.8 | 20.4 | 20.9 | 21.3 | 19.2 | 21.0 | 19.8 | — | 18.8 | 18.3 | 19.4 | — | 19.3 | 20.8 | 17.4 | — | — | 18.0 | 18.0 | 19.6 | 19.2 | — | 20.5 | 20.1 | **5.7** | 1.2 |
| **29** | *P.victorianus* | 21.2 | 22.3 | 22.8 | 21.5 | 20.7 | 21.2 | 20.4 | 18.3 | 23.6 | 18.2 | — | 17.4 | 17.4 | 18.6 | — | 18.4 | 19.0 | 15.6 | — | — | 18.1 | 18.6 | 19.5 | 18.3 | — | 19.9 | 19.5 | 13.5 | — |
