# Supplementary material for: An integrative taxonomic revision of slug-eating snakes (Squamata: Pareidae: Pareineae) reveals unprecedented diversity in Indochina
Source: PeerJ. 2022 Jan 10;10:e12713. doi: 10.7717/peerj.12713 (PMC8757378; doi:10.7717/peerj.12713)
Supplement: Supplemental Information 9 — Node No. – estimated tree node, for node names see Fig. S1; divergence time given in millions years (mya). [file peerj-10-12713-s009.docx]

**Supplementary Table S9. Results of divergence time estimates for Pareinae.**

Node No. – estimated tree node, for node names see Supplementary Figure S1; divergence time given in millions years (mya).

| **Node No.** | **Estimated age (mya)** | |
| --- | --- | --- |
|  | **mean** | **95% CI range** |
| **1** | 42.23 | [34.17 – 50.17] |
| **2** | 26.85 | [18.02 – 36.6] |
| **3** | 39.26 | [31.65 – 47.08] |
| **4** | 29.96 | [19.12 – 40.65] |
| **5** | 7.75 | [4.35 – 12.18] |
| **6** | 4.65 | [2.17 – 7.75] |
| **7** | 6.16 | [3.01 – 9.91] |
| **8** | 33.62 | [26.33 – 40.36] |
| **9** | 12.31 | [6.89 – 19.29] |
| **10** | 31.25 | [24.65 – 37.92] |
| **11** | 22.65 | [16.76 – 28.56] |
| **12** | 13.36 | [9.44 – 17.64] |
| **13** | 11.29 | [7.7 – 15.19] |
| **14** | 5.9 | [3.94 – 8.27] |
| **15** | 4.04 | [2.67 – 5.78] |
| **16** | 1.58 | [0.96 – 2.27] |
| **17** | 1.41 | [0.87 – 2.05] |
| **18** | 0.88 | [0.48 – 1.4] |
| **19** | 0.61 | [0.23 – 1.13] |
| **20** | 1.32 | [0.7 – 2.2] |
| **21** | 0.52 | [0.2 – 0.95] |
| **22** | 4.98 | [3.02 – 7.3] |
| **23** | 0.4 | [0.12 – 0.79] |
| **24** | 3.23 | [1.79 – 4.97] |
| **25** | 17.15 | [10.92 – 23.47] |
| **26** | 9.32 | [5.41 – 13.66] |
| **27** | 0.66 | [0.27 – 1.23] |
| **28** | 23.93 | [18.23 – 29.86] |
| **29** | 21.87 | [16.69 – 27.51] |
| **30** | 20.74 | [15.36 – 25.97] |
| **31** | 14.19 | [10.45 – 18.34] |
| **32** | 12.39 | [9.13 – 16.17] |
| **33** | 11.96 | [8.48 – 15.55] |
| **34** | 7.21 | [5.08 – 9.65] |
| **35** | 3.62 | [2.5 – 4.93] |
| **36** | 3.15 | [2.11 – 4.31] |
| **37** | 2.87 | [1.93 – 4] |
| **38** | 2.14 | [1.2 – 3.17] |
| **39** | 2.42 | [1.5 – 3.54] |
| **40** | 2.05 | [1.28 – 3.04] |
| **41** | 0.66 | [0.29 – 1.18] |
| **42** | 0.93 | [0.42 – 1.66] |
| **43** | 6.35 | [4.32 – 8.53] |
| **44** | 5.22 | [3.42 – 7.23] |
| **45** | 1.98 | [1.01 – 3.25] |
| **46** | 0.72 | [0.28 – 1.36] |
| **47** | 0.76 | [0.32 – 1.47] |
| **48** | 0.21 | [0.03 – 0.6] |
| **49** | 7.81 | [3.78 – 12.54] |
| **50** | 1.08 | [0.36 – 2.26] |
| **51** | 5.68 | [3.48 – 8.3] |
| **52** | 4.39 | [2.45 – 6.84] |
| **53** | 0.56 | [0.15 – 1.23] |
| **54** | 0.58 | [0.21 – 1.11] |
| **55** | 12.37 | [8.73 – 16.08] |
| **56** | 10.83 | [7.58 – 14.45] |
| **57** | 7.23 | [4.12 – 11.18] |
| **58** | 8.15 | [5.15 – 11.4] |
| **59** | 5.22 | [3.21 – 7.49] |
| **60** | 2.87 | [1.52 – 4.63] |
| **61** | 15.21 | [9.93 – 21.2] |
| **62** | 7.07 | [3.97 – 11.07] |
| **63** | 14.1 | [8.99 – 19.98] |
| **64** | 3.38 | [1.7 – 5.43] |
