# Supplementary material for: An integrative taxonomic revision of slug-eating snakes (Squamata: Pareidae: Pareineae) reveals unprecedented diversity in Indochina
Source: PeerJ. 2022 Jan 10;10:e12713. doi: 10.7717/peerj.12713 (PMC8757378; doi:10.7717/peerj.12713)
Supplement: Supplemental Information 10 — Abbreviations are listed in the Materials and methods. [file peerj-10-12713-s010.docx]

**Supplementary Table S10.** Summary statistics and principal component analysis scores for the *Pareas carinatus – P. nuchalis* complex. Abbreviations are listed in the Materials and methods.

|  | **PC1** | **PC2** | **PC3** | **PC4** | **PC5** | **PC6** | **PC7** | **PC8** | **PC9** | **PC10** | **PC11** | **PC12** |
| --- | --- | --- | --- | --- | --- | --- | --- | --- | --- | --- | --- | --- |
| **Eigenv.** | **5.7227** | **4.5876** | **3.0140** | **2.9138** | **2.0082** | **1.8558** | **1.5717** | **1.3683** | **1.2197** | **1.0650** | **0.8289** | **0.6550** |
| **Total var.** | **18.4604** | **14.7987** | **9.7225** | **9.3994** | **6.4780** | **5.9865** | **5.0701** | **4.4138** | **3.9346** | **3.4355** | **2.6740** | **2.1129** |
| **Cum. eigen.** | **5.7227** | **10.3103** | **13.3243** | **16.2381** | **18.2463** | **20.1021** | **21.6738** | **23.0421** | **24.2618** | **25.3268** | **26.1557** | **26.8108** |
| **Cum. var.** | **18.4604** | **33.2591** | **42.9816** | **52.3810** | **58.8590** | **64.8455** | **69.9156** | **74.3293** | **78.2639** | **81.6994** | **84.3734** | **86.4863** |
| **SVL** | 0.1161 | 0.6723 | -0.4468 | 0.2512 | 0.3894 | 0.0246 | -0.1704 | 0.1346 | -0.0032 | -0.0955 | 0.0740 | 0.0981 |
| **TaL** | -0.6151 | 0.5930 | -0.2907 | 0.1933 | 0.2681 | 0.0823 | -0.0934 | 0.1214 | 0.0927 | -0.0193 | -0.0256 | 0.0676 |
| **TL** | -0.1188 | 0.7290 | -0.4305 | 0.2913 | 0.3149 | 0.0220 | -0.1373 | 0.1076 | 0.0354 | -0.0831 | 0.0564 | 0.0524 |
| **TaL/TL** | -0.9119 | 0.1089 | 0.0409 | -0.0427 | 0.0485 | 0.1045 | 0.0050 | 0.0720 | 0.1001 | 0.0701 | -0.1162 | 0.0394 |
| **DORkeel** | 0.5233 | 0.5680 | -0.0325 | 0.1122 | -0.1989 | 0.1774 | 0.1400 | 0.1548 | -0.0670 | 0.2998 | -0.1131 | -0.0372 |
| **KMD** | 0.2266 | 0.7549 | -0.0290 | 0.1948 | -0.3188 | -0.0459 | 0.2770 | 0.0398 | -0.0507 | 0.0283 | -0.1368 | -0.0356 |
| **VSE** | 0.4946 | -0.1936 | -0.4069 | 0.3746 | -0.1889 | -0.0569 | 0.0500 | 0.0791 | -0.0018 | 0.0276 | 0.2926 | 0.2446 |
| **VEN** | -0.7545 | 0.0176 | -0.4573 | -0.0250 | 0.0005 | 0.0856 | 0.1737 | -0.1186 | -0.1945 | -0.1196 | -0.0442 | -0.0429 |
| **preVEN** | 0.1628 | -0.2797 | 0.0624 | -0.4324 | 0.4838 | 0.2119 | 0.0262 | 0.4838 | -0.0464 | -0.0064 | 0.0401 | 0.2697 |
| **SC** | -0.8998 | 0.0475 | -0.3065 | -0.1078 | 0.0187 | -0.0012 | 0.1057 | -0.0726 | 0.0012 | 0.0003 | -0.0563 | -0.0673 |
| **SL** | -0.4820 | 0.4593 | 0.1114 | -0.2562 | -0.1973 | 0.1270 | 0.2448 | -0.2551 | -0.2142 | 0.1484 | 0.2067 | 0.1751 |
| **SL-eye** | -0.4727 | -0.2351 | -0.2570 | 0.0602 | 0.2835 | 0.2456 | 0.4471 | -0.2139 | 0.0488 | 0.2790 | -0.0099 | 0.0310 |
| **At** | -0.2726 | 0.0511 | -0.2228 | -0.1696 | -0.2131 | -0.3427 | -0.2200 | 0.5178 | -0.0765 | 0.2647 | -0.3684 | -0.2166 |
| **Pt** | -0.1592 | 0.0447 | -0.3203 | 0.2165 | -0.2241 | -0.4940 | -0.0398 | 0.1559 | -0.3908 | 0.3668 | 0.0218 | 0.2279 |
| **LOR** | -0.4063 | -0.3700 | -0.1370 | -0.1856 | -0.1967 | -0.2874 | -0.2405 | 0.1197 | -0.2186 | -0.4979 | -0.0030 | 0.0765 |
| **Preoc** | -0.0042 | 0.2498 | 0.3957 | -0.4671 | 0.0497 | 0.2266 | 0.3179 | 0.4731 | -0.1955 | -0.0433 | 0.1920 | -0.0365 |
| **Presuboc** | 0.1070 | -0.2601 | -0.2409 | -0.3711 | 0.2007 | 0.5178 | -0.3397 | -0.0388 | -0.0976 | 0.3591 | -0.0935 | -0.1038 |
| **Prefr-eye** | -0.6898 | -0.4990 | -0.2979 | -0.1667 | 0.0380 | -0.0137 | 0.0986 | -0.1001 | -0.1215 | -0.0237 | 0.0123 | 0.0119 |
| **SoO** | -0.2542 | 0.2167 | 0.1335 | -0.3397 | -0.3658 | 0.0499 | -0.4514 | -0.1507 | -0.0350 | 0.3112 | 0.3308 | 0.1628 |
| **SoO-PoO** | -0.1341 | -0.0158 | 0.2188 | 0.3152 | 0.5266 | -0.4043 | 0.2594 | 0.0011 | 0.0949 | 0.1641 | 0.0376 | -0.0081 |
| **PoO** | -0.0295 | 0.5109 | -0.1396 | -0.6502 | -0.1184 | -0.0228 | -0.1837 | -0.1193 | -0.0164 | -0.1907 | -0.1495 | 0.1792 |
| **IL** | 0.2836 | 0.4484 | -0.1760 | 0.2011 | 0.1553 | 0.4053 | -0.4094 | -0.1919 | -0.1473 | -0.1217 | 0.0021 | -0.1329 |
| **ED** | -0.3457 | -0.0923 | 0.0518 | -0.0493 | -0.2217 | 0.0700 | -0.1411 | 0.1629 | 0.7810 | 0.1025 | -0.0835 | 0.2365 |
| **Eye-nos** | -0.3466 | 0.4734 | 0.6443 | -0.3269 | 0.1734 | -0.0137 | -0.0974 | -0.0359 | -0.0211 | 0.0572 | 0.0081 | -0.0840 |
| **Eye-mouth** | -0.4200 | 0.0447 | -0.0988 | 0.1673 | -0.3024 | 0.3714 | 0.1344 | 0.4235 | 0.0141 | -0.1453 | 0.2422 | -0.1618 |
| **HW** | -0.3191 | -0.2880 | 0.4073 | 0.4981 | -0.0355 | 0.1860 | -0.2293 | -0.0286 | -0.2861 | 0.0951 | -0.0346 | -0.0718 |
| **HL** | -0.3291 | 0.7079 | 0.3136 | 0.0253 | -0.2759 | -0.1746 | 0.1445 | -0.0975 | 0.1699 | -0.1054 | 0.0752 | -0.1242 |
