# Supplementary material for: An integrative taxonomic revision of slug-eating snakes (Squamata: Pareidae: Pareineae) reveals unprecedented diversity in Indochina
Source: PeerJ. 2022 Jan 10;10:e12713. doi: 10.7717/peerj.12713 (PMC8757378; doi:10.7717/peerj.12713)
Supplement: Supplemental Information 11 — Pareas abros sp. nov., P. kuznetsovorum sp. nov., P. carinatus (including two subspecies: P. c. carinatus and P. c. tenasserimicus ssp. nov.), P. berdmorei (including three subspecies: P. berdmorei truongsonicus ssp. nov., P. berdmorei berdmorei, and P. berdmorei unicolor), P. nuchalis and P. temporalis. Abbreviations are listed in the Materials and methods. (? = not available). [file peerj-10-12713-s011.docx]

**Supplementary Table S11.** Measurements and scale counts of members of the subgenus *Pareas*: *Pareas abros* **sp. nov.**, *P. kuznetsovorum* **sp. nov**., P. carinatus (including two subspecies: *P.* c. *carinatus* and *P.* c. *tenasserimicus* **ssp. nov.**), P. berdmorei (including three subspecies: *P.* berdmorei *truongsonicus* **ssp. nov.**, *P.* berdmorei *berdmorei*, and *P.* berdmorei *unicolor*), *P. nuchalis* and *P. temporalis*. Abbreviations are listed in the Materials and methods. (? = not available). (Continued on the next page)

| **#** | **Species** | **Type** | **Voucher number** | **Locality** | **Sex** | **SVL** | **TaL** | **VEN** | **SC** |
| --- | --- | --- | --- | --- | --- | --- | --- | --- | --- |
| 1 | *P. abros* **sp. nov.** | Holotype | ZMMU R-16393 | Quang Nam, Vietnam | M | 314 | 120 | 184 | 92 |
| 2 | *P. abros* **sp. nov.** | Paratype | ZMMU R-16392 | Thua Thien-Hue, Vietnam | M | 403 | 162 | 180 | 95 |
| 3 | *P. abros* **sp. nov.** | Paratype | ZMMU R-14788 | Thua Thien-Hue, Vietnam | F | 383 | 138 | 184 | 83 |
| 1 | *P. kuznetsovorum* **sp. nov.** | Holotype | ZMMU R-16802 | Song Hinh, Phu Yen, Vietnam | M | 478.5 | 161 | 167 | 87 |
| 1 | *P. c. carinatus* | 0 | NMW 28131.1 | Borneo, Malaysia | M | 385 | 104 | 167 | 66 |
| 2 | *P. c. carinatus* | 0 | NMW 28131.2 | Borneo, Malaysia | M | 311 | 82 | 167 | 60 |
| 3 | *P. c. carinatus* | 0 | NMW 28134.3 | Java, Indonesia | M | 366 | 112 | 160 | 66 |
| 4 | *P. c. carinatus* | 0 | NMW 28134.4 | Java, Indonesia | M | 395 | 112 | 168 | 69 |
| 5 | *P. c. carinatus* | 0 | NMW 28134.8 | Java, Indonesia | M | 363 | 106 | 174 | 71 |
| 6 | *P. c. carinatus* | 0 | NMW 39664.2 | Fraser's Hill, Pahang, Malaysia | M | 435 | 136 | 183 | 84 |
| 7 | *P. c. carinatus* | Lectotype | RMNH 954 (C) | Java, Indonesia | M | 262 | 75 | 164 | 70 |
| 8 | *P. c. carinatus* | Paralectotype | RMNH 954 (A) | Java, Indonesia | M | 373 | 101 | 170 | 67 |
| 9 | *P. c. carinatus* | 0 | SMF 25995 | Bogor, Java, Indonesia | M | 343 | 97 | 164 | 69 |
| 10 | *P. c. carinatus* | 0 | SMF 37825 | Ranau, Sumatra | M | 345 | 98 | 165 | 60 |
| 11 | *P. c. carinatus* | 0 | SMF 37826 | Ranau, Sumatra | M | 351 | 97 | 166 | 63 |
| 12 | *P. c. carinatus* | 0 | SMF 55295 | Karimund, Java, Indonesia | M | 401 | 133 | 161 | 71 |
| 13 | *P. c. carinatus* | 0 | ZMH R11547 | East Java, Indonesia | M | 345 | 100 | 158 | 62 |
| 14 | *P. c. carinatus* | 0 | ZMH R11548 | East Java, Indonesia | M | 407 | 118 | 169 | 69 |
| 15 | *P. c. carinatus* | 0 | ZMH 4053 | Kutai Kartanegara, Borneo, Indonesia | M | 402 | 117 | 173 | 73 |
| 16 | *P. c. carinatus* | 0 | NMW 28131.3 | Muara Taweh, Borneo, Indonesia | F | ? | ? | 176 | 67 |
| 17 | *P. c. carinatus* | 0 | NMW 39664.9 | West Malaysia | F | 435 | 122 | 175 | 75 |
| 18 | *P. c. carinatus* | 0 | NMW 39664.11 | West Malaysia | F | 438 | 121 | 178 | 79 |
| 19 | *P. c. carinatus* | 0 | NMW 39664.15 | Trengganu, Malaysia | F | 476 | 132 | 188 | 78 |
| 20 | *P. c. carinatus* | Paralectotype | RMNH 954 (B) | Java, Indonesia | F | 365 | 81 | 165 | 54 |
| 21 | *P. c. carinatus* | 0 | SMF 20797 | Bogor, Java, Indonesia | F | 405 | 91 | 173 | 61 |
| 22 | *P. c. carinatus* | 0 | ZMH R05520-1 | Java, Indonesia | F | 381 | ? | 175 | ? |
| 23 | *P. c. carinatus* | 0 | ZMH R11546 | East Java, Indonesia | F | 374 | 93 | 162 | 57 |
| 24 | *P. c. carinatus* | 0 | ZMH R11542 | West Java, Indonesia | F | 381 | 101 | 170 | 69 |
| 25 | *P. c. carinatus* | 0 | ZSM 154.1999 | North Sumatra, Indonesia | F | 371 | 102 | 190 | 75 |
| 26 | *P. c. tenasserimicus* **ssp. nov.** | Holotype | ZMMU R-16800 | Suan Phueng, Ratchaburi, Thailand | M | 524 | 178 | 194 | 96 |
| 1 | *P. b. truongsonicus* **ssp. nov.** | Paratype | ZMMU R-14796 | Tuyen Hoa, Quang Binh, Vietnam | M | 499.0 | 123.0 | 187 | 66 |
| 2 | *P. b. truongsonicus* **ssp. nov.** | Holotype | ZMMU R-16801 | Ban Nahin-Nai, Khammouan, Laos | M | 502.0 | 135.0 | 187 | 73 |
| 3 | *P.* cf. *b. truongsonicus* **ssp. nov.** | 0 | ZFMK 82890 | Phong Nha-Ke Bang, Quang Binh, Vietnam | M | 365.0 | 123.0 | 167 | 78 |
| 4 | *P.* cf. *b. truongsonicus* **ssp. nov.** | 0 | VNUH 15.6.’05-1 | Phong Nha-Ke Bang, Quang Binh, Vietnam | M | 457.0 | 144.0 | 177 | 80 |
| 3 | *P. b. berdmorei* | Topotype | CAS 240362 | Mon, Myanmar | M | 522 | 154 | 185 | 73 |
| 4 | *P. b. berdmorei* | 0 | CIB 725061 | Xishuangbannna, Yunnan, China | M | 488.0 | 122.0 | 183 | 83 |
| 5 | *P. b. berdmorei* | 0 | CIB 736216 | Pu'er, Yunnan, China | M | 410.0 | 120.0 | 179 | 89 |
| 6 | *P. b. berdmorei* | 0 | EHT-HMS 31796 | Loei, Thailand | M | 430.0 | ? | 181 | ? |
| 7 | *P. b. berdmorei* | 0 | NHMUK 1912147 | Lai Chau, Vietnam | M | 465 | ? | 183 | ? |
| 8 | *P. b. berdmorei* | Paratype of *P.menglangensis* | YBU 14141 | Mengla, Yunnan, China | M | 448 | 137 | 176 | 74 |
| 9 | *P. b. berdmorei* | Paratype of *P.menglangensis* | YBU 14142 | Mengla, Yunnan, China | M | 353 | 98 | 176 | 74 |
| 10 | *P. b. berdmorei* | Lectotype | ZSI 8022 | Mon, Myanmar | F | 490 | 120 | 174 | 64 |
| 11 | *P. b. berdmorei* | 0 | KIZ 7410023 | Pu'er, Yunnan, China | M | ? | ? | 172 | 77 |
| 12 | *P. b. berdmorei* | 0 | KIZ 40 | Pu'er, Yunnan, China | M | ? | ? | 177 | ? |
| 13 | *P. b. berdmorei* | 0 | EHT-HMS 3626 | Chiangmai, Thailand | F | 488.0 | 108.0 | 174 | 72 |
| 14 | *P. b. berdmorei* | 0 | EHT-HMS 31797 | Loei, Thailand | F | 522.0 | 111.0 | 178 | 71 |
| 15 | *P. b. berdmorei* | 0 | HNUE MNR.15 | Muong Nhe, Dien Bien, Vietnam | F | 429 | 103 | 186 | 75 |
| 16 | *P. b. berdmorei* | 0 | NMW 39664:3 | Vinh Phuc, Vietnam | F | 412 | 117 | 186 | 78 |
| 17 | *P. b. berdmorei* | 0 | MNHN RA-1896.556 | Luang Prabang, Laos | F | 447 | 110 | 177 | 64 |
| 18 | *P. b. berdmorei* | 0 | TBU LC.2018.11 | Sin Ho, Lai Chau, Vietnam | F | 595 | 175 | 185 | 82 |
| 19 | *P. b. berdmorei* | Holotype of *P.menglangensis* | YBU 14124 | Mengla, Yunnan, China | F | 472 | 111 | 177 | 65 |
| 20 | *P. b. berdmorei* | 0 | ZMMU R-16803 | Suan Phueng, Ratchaburi, Thailand | F | 404 | 101 | 166 | 57 |
| 21 | *P. b. berdmorei* | 0 | KIZ 7911081 | Pu'er, Yunnan, China | F | 468.0 | 115.0 | 176 | 71 |
| 22 | *P. b. berdmorei* | 0 | KIZ 741212 | Pu'er, Yunnan, China | F | 375.0 | 140.0 | 174 | 77 |
| 23 | *P. b. berdmorei* | 0 | KIZ 79110081 | Pu'er, Yunnan, China | F | 500.0 | 120.0 | 175 | 72 |
| 24 | *P. b. unicolor* | 0 | MNHN 1970.480 | Cambodia | M | 383 | 122 | 172 | 73 |
| 25 | *P. b. unicolor* | 0 | ZMMU NAP-10584 | Cat Tien, Dong Nai, Vietnam | M | 468 | 108 | 172 | 73 |
| 26 | *P. b. unicolor* | 0 | ZMMU NAP-10585 | Cat Tien, Dong Nai, Vietnam | M | 466 | 109.5 | 180 | 75 |
| 27 | *P. b. unicolor* | 0 | DTU 472 | Cat Tien, Dong Nai, Vietnam | F | 412.8 | 114.5 | 176 | 75 |
| 28 | *P. b. unicolor* | 0 | DTU 473 | Cat Tien, Dong Nai, Vietnam | F | 385.8 | 81.3 | 177 | 69 |
| 29 | *P. b. unicolor* | 0 | DTU 474 | Bay Nui, An Giang, Vietnam | F | 426.9 | 111.2 | 174 | 67 |
| 30 | *P. b. unicolor* | Holotype | MNHN 1938.0149 | Kampong Speu, Cambodia | F | 390 | 96 | 164 | 64 |
| 31 | *P. b. unicolor* | 0 | MNHN RA-1937.27 | Trang Bom, Dong Nai, Vietnam | F | 366 | 93 | 173 | 65 |
| 32 | *P. b. unicolor* | 0 | SIEZC 20216 | Di Linh, Lam Dong, Vietnam | F | 415 | 98 | 176 | 58 |
| 33 | *P. berdmorei* ssp. | 0 | NHMUK 62.7.28.8 | Laos | M | 355 | 100 | 173 | 70 |
| 34 | *P. berdmorei* ssp. | 0 | MNHN RA-1896.655 | Northern Laos | F | 482 | 125 | 175 | 73 |
| 35 | *P. berdmorei* ssp. | 0 | MNHN RA-1896.656 | Northern Laos | F | 406 | 96 | 173 | 64 |
| 36 | *P. berdmorei* ssp. | 0 | MNHN RA-1896.657 | Laos | F | 421 | ? | 182 | ? |
| 1 | *P. nuchalis* | Holotype | NHMUK 1912247 | Saribas, Sarawak, Malaysia | M | 489 | 189 | 220 | 115 |
| 2 | *P. nuchalis* | 0 | FMNH 131635 | Niah, Sarawak, Malaysia | M | 415 | 145 | 210 | 111 |
| 3 | *P. nuchalis* | 0 | FMNH 239902 | Tenom, Sabah, Malaysia | M | 263 | 82 | 211 | 108 |
| 4 | *P. nuchalis* | 0 | FMNH 239903 | Tenom, Sabah, Malaysia | M | 351 | 147 | 211 | 112 |
| 5 | *P. nuchalis* | 0 | FMNH 269040 | Bintulu, Sarawak, Malaysia | M | 367 | 147 | 207 | 119 |
| 6 | *P. nuchalis* | 0 | USNM 070863 | Kepahiang, Sumatra, Indonesia | M | 415 | 164 | 214 | 120 |
| 7 | *P. nuchalis* | 0 | FMNH 131636 | Niah, Sarawak, Malaysia | F | 309 | 103 | 208 | 102 |
| 8 | *P. nuchalis* | 0 | FMNH 269041 | Bintulu, Sarawak, Malaysia | F | 263 | 89 | 201 | 106 |
| 9 | *P. nuchalis* | 0 | ZMH R3971 | Indragiri, Sumatra, Indonesia | F | 368 | 135 | 207 | 107 |
| 1 | *P. temporalis* | Holotype | UNS 09992 | Da Huoai, Lam Dong, Vietnam | F | 426 | 152 | 191 | 92 |
| 2 | *P. temporalis* | 0 | ZMMU R-13656 | Cat Loc, Lam Dong, Vietnam | M | 413 | 142 | 198 | 92 |
| 3 | *P. temporalis* | 0 | DTU 471 | Di Linh, Lam Dong, Vietnam | F | 443.5 | 146.3 | 188 | 86 |
| 4 | *P. temporalis* | 0 | DTU 487 | Di Linh, Lam Dong, Vietnam | F | 380 | 120 | 185 | 87 |
| 5 | *P. temporalis* | 0 | DTU 488 | Di Linh, Lam Dong, Vietnam | F | 410 | 135 | 187 | 89 |
| 6 | *P. temporalis* | 0 | SIEZC 20214 | Gia Bac, Lam Dong, Vietnam | F | 460 | 152 | 187 | 87 |
| 7 | *P. temporalis* | 0 | SIEZC 20215 | Bidoup, Lam Dong, Vietnam | F | 508 | 157 | 187 | 88 |

**Supplementary Table S11.** **Continued.**

| **#** | **Species** | **Voucher number** | **KMD** | **VSE** | **SL** | **IL** | **At** | **Pt** | **SoO** | **PoO** | **Source** |
| --- | --- | --- | --- | --- | --- | --- | --- | --- | --- | --- | --- |
| 1 | *P. abros* **sp. nov.** | ZMMU R-16393 | 11 | 1 | 9/9 | 8/8 | 3/3 | 3/3 | 3/3 | 2/2 | *this study* |
| 2 | *P. abros* **sp. nov.** | ZMMU R-16392 | 11 | 1 | 9/9 | 8/9 | 3/3 | 3/3 | 3/3 | 2/2 | *this study* |
| 3 | *P. abros* **sp. nov.** | ZMMU R-14788 | 9 | 1 | 9/9 | 8/8 | 3/3 | 3/3 | 3/3 | 2/2 | *this study* |
| 1 | *P. kuznetsovorum* **sp. nov.** | ZMMU R-16802 | 0 | 1 | 7/7 | 8/7 | 3/3 | 4/4 | 2/2 | 1/1 | *this study* |
| 1 | *P. c. carinatus* | NMW 28131.1 | 1 | 3 | 7/7 | 8/8 | 3/3 | 4/4 | 2/2 | 1/1 | *this study* |
| 2 | *P. c. carinatus* | NMW 28131.2 | 5 | 3 | 7/7 | 8/8 | 3/3 | 4/4 | 2/2 | 1/1 | *this study* |
| 3 | *P. c. carinatus* | NMW 28134.3 | 3 | 3 | 7/7 | 8/7 | 3/3 | 3/3 | 2/2 | 0/1 | *this study* |
| 4 | *P. c. carinatus* | NMW 28134.4 | 5 | 3 | 7/7 | 8/8 | 3/3 | 4/4 | 2/2 | 1/1 | *this study* |
| 5 | *P. c. carinatus* | NMW 28134.8 | 9 | 3 | 7/7 | 7/8 | 3/3 | 4/4 | 2/2 | 1/1 | *this study* |
| 6 | *P. c. carinatus* | NMW 39664.2 | 3 | 3 | 8/7 | 9/9 | 3/2 | 3/4 | 2/1 | 1/1 | *this study* |
| 7 | *P. c. carinatus* | RMNH 954 (C) | ? | 3 | 7/7 | 8/? | 3/3 | 3/3 | 1/1 | 1/1 | *this study* |
| 8 | *P. c. carinatus* | RMNH 954 (A) | ? | 3 | 7/7 | 9/9 | 3/3 | 4/4 | 2/2 | 1/1 | *this study* |
| 9 | *P. c. carinatus* | SMF 25995 | 5 | 3 | 6/7 | 7/7 | 4/3 | 4/4 | 2/2 | 1/1 | *this study* |
| 10 | *P. c. carinatus* | SMF 37825 | ? | 3 | 7/7 | 8/7 | 2/2 | 3/3 | 2/1 | 1/1 | *this study* |
| 11 | *P. c. carinatus* | SMF 37826 | ? | 3 | 7/7 | 7/7 | 3/2 | 3/3 | 1/2 | 1/1 | *this study* |
| 12 | *P. c. carinatus* | SMF 55295 | 11 | 3 | 8/8 | 7/7 | 3/3 | 4/4 | 1/1 | 1/1 | *this study* |
| 13 | *P. c. carinatus* | ZMH R11547 | 7 | 3 | 7/7 | 7/7 | 4/3 | 4/4 | 2/2 | 0/1 | *this study* |
| 14 | *P. c. carinatus* | ZMH R11548 | 3 | 3 | 9/7 | 8/8 | 3/3 | 4/4 | 3/3 | 1/1 | *this study* |
| 15 | *P. c. carinatus* | ZMH 4053 | 11 | 3 | 7/6 | 9/8 | 4/3 | 3/3 | 2/3 | 1/1 | *this study* |
| 16 | *P. c. carinatus* | NMW 28131.3 | 9 | 3 | 7/7 | 7/8 | 3/3 | 4/4 | 2/1 | 1/1 | *this study* |
| 17 | *P. c. carinatus* | NMW 39664.9 | 9 | 3 | 7/8 | 7/8 | 3/3 | 4/4 | 2/2 | 1/1 | *this study* |
| 18 | *P. c. carinatus* | NMW 39664.11 | 7 | 3 | 8/8 | 7/8 | 3/3 | 5/4 | 2/1 | 1/1 | *this study* |
| 19 | *P. c. carinatus* | NMW 39664.15 | 7 | 3 | 8/8 | 9/9 | 2/2 | 3/3 | 2/2 | 1/1 | *this study* |
| 20 | *P. c. carinatus* | RMNH 954 (B) | ? | 3 | 8/8 | ?/9 | 2/2 | 3/3 | 2/2 | 1/1 | *this study* |
| 21 | *P. c. carinatus* | SMF 20797 | 5 | 3 | 7/7 | 7/7 | 3/3 | 4/4 | 2/3 | 1/1 | *this study* |
| 22 | *P. c. carinatus* | ZMH R05520-1 | 3 | 3 | 8/7 | 8/8 | 2/2 | 3/3 | 3/3 | 1/1 | *this study* |
| 23 | *P. c. carinatus* | ZMH R11546 | 0 | 3 | 7/7 | 8/8 | 3/3 | 3/3 | 2/2 | 1/1 | *this study* |
| 24 | *P. c. carinatus* | ZMH R11542 | ? | 3 | 7/7 | 9/9 | 3/3 | 2/4 | 2/1 | 1/1 | *this study* |
| 25 | *P. c. carinatus* | ZSM 154.1999 | 11 | 3 | 8/8 | 7/7 | 3/2 | 4/5 | 2/3 | 1/1 | *this study* |
| 26 | *P. c. tenasserimicus* **ssp. nov.** | ZMMU R-16800 | 7 | 3 | 7/7 | 9/9 | 3/3 | 3/3 | 2/2 | 2/2 | *this study* |
| 1 | *P. b. truongsonicus* **ssp. nov.** | ZMMU R-14796 | 13 | 3 | 7/7 | 9/9 | 3/3 | 4/4 | 1/1 | 1/1 | *this study* |
| 2 | *P. b. truongsonicus* **ssp. nov.** | ZMMU R-16801 | 13 | 3 | 7/7 | 9/10 | 3/3 | 4/4 | 1/1 | 1/1 | *this study* |
| 3 | *P.* cf. *b. truongsonicus* **ssp. nov.** | ZFMK 82890 | ? | ? | 6/7 | ? | ? | ? | ? | ? | *Ziegler et al. 2006* |
| 4 | *P.* cf. *b. truongsonicus* **ssp. nov.** | VNUH 15.6.’05-1 | ? | ? | 6/7 | ? | ? | ? | ? | ? | *Ziegler et al. 2006* |
| 3 | *P. b. berdmorei* | CAS 240362 | 6 | 3 | 7/7 | 10/10 | 3/3 | 4/4 | 3/3 | 1/1 | *this study* |
| 4 | *P. b. berdmorei* | CIB 725061 | 11 | 3 | ? | ? | 3/3 | 3/3 | ? | ? | *Yang & Rao, 2008* |
| 5 | *P. b. berdmorei* | CIB 736216 | 11 | 3 | ? | ? | 3/3 | 4/4 | ? | ? | *Yang & Rao, 2008* |
| 6 | *P. b. berdmorei* | EHT-HMS 31796 | ? | 3 | 7/7 | 8/9 | ? | ? | ? | ? | *Yang & Rao, 2008* |
| 7 | *P. b. berdmorei* | NHMUK 1912147 | 13 | 3 | 9/9 | 8/9 | 4/3 | 4/4 | 2/2 | 1/1 | *this study* |
| 8 | *P. b. berdmorei* | YBU 14141 | 11 | 3 | 7/7 | 8/7 | 3/3 | 4/4 | 2/2 | 1/1 | *Wang et al., 2020* |
| 9 | *P. b. berdmorei* | YBU 14142 | 11 | 3 | 7/7 | 7/8 | 3/3 | 4/4 | 2/2 | 1/1 | *Wang et al., 2020* |
| 10 | *P. b. berdmorei* | ZSI 8022 | 9 | 3 | 7/7 | 8/8 | 3/3 | 4/4 | 2/2 | 1/1 | *this study* |
| 11 | *P. b. berdmorei* | KIZ 7410023 | 9 | 3 | ? | ? | 3/3 | 4/4 | ? | ? | *Yang & Rao, 2008* |
| 12 | *P. b. berdmorei* | KIZ 40 | 11 | 3 | ? | ? | 3/3 | 3/4 | ? | ? | *Yang & Rao, 2008* |
| 13 | *P. b. berdmorei* | EHT-HMS 3626 | ? | 3 | 8/7 | 9/8 | ? | ? | ? | ? | *Taylor, 1962* |
| 14 | *P. b. berdmorei* | EHT-HMS 31797 | ? | 3 | 6/7 | 8/? | ? | ? | ? | ? | *Taylor, 1962* |
| 15 | *P. b. berdmorei* | HNUE MNR.15 | ? | 3 | 8/8 | 9/9 | 2/2 | 2/3 | 2/2 | 1/1 | *Le et al., 2014* |
| 16 | *P. b. berdmorei* | NMW 39664:3 | ? | 3 | 7/7 | 8/8 | 3/4 | 4/5 | 2/2 | 1/1 | *this study* |
| 17 | *P. b. berdmorei* | MNHN RA-1896.556 | 7 | 1 | 8/7 | 8/8 | 3/3 | 3/3 | 2/2 | 0/0 | *this study* |
| 18 | *P. b. berdmorei* | TBU LC.2018.11 | ? | 3 | 7/7 | 8/8 | 2/2 | 3/3 | 2/2 | 2/2 | *Pham & Nguyen, 2019* |
| 19 | *P. b. berdmorei* | YBU 14124 | 11 | 3 | 7/7 | 8/7 | 3/3 | 4/4 | 2/2 | 1/1 | *Wang et al., 2020* |
| 20 | *P. b. berdmorei* | ZMMU R-16803 | 5 | 3 | 7/7 | 8/? | 2/2 | 2/2 | 2/2 | 1/1 | *this study* |
| 21 | *P. b. berdmorei* | KIZ 7911081 | 9 | 3 | ? | ? | 3/3 | 3/3 | ? | ? | *Yang & Rao, 2008* |
| 22 | *P. b. berdmorei* | KIZ 741212 | 11 | 3 | ? | ? | 3/3 | 3/3 | ? | ? | *Yang & Rao, 2008* |
| 23 | *P. b. berdmorei* | KIZ 79110081 | 9 | 3 | ? | ? | 2/2 | 2/2 | ? | ? | *Yang & Rao, 2008* |
| 24 | *P. b. unicolor* | MNHN 1970.480 | 5 | 1 | 8/8 | 8/8 | 3/3 | 3/3 | 2/2 | 1/1 | *this study* |
| 25 | *P. b. unicolor* | ZMMU NAP-10584 | 7 | 3 | 7/7 | 8/8 | 3/3 | 4/4 | 2/2 | 2/2 | *this study* |
| 26 | *P. b. unicolor* | ZMMU NAP-10585 | 7 | 3 | 7/7 | 8/8 | 3/3 | 3/3 | 2/2 | 2/1 | *this study* |
| 27 | *P. b. unicolor* | DTU 472 | 9 | 3 | 8/8 | 8/8 | 3/3 | 4/4 | 3/3 | 2/2 | *this study* |
| 28 | *P. b. unicolor* | DTU 473 | 9 | 3 | 7/7 | 8/8 | 3/3 | 4/4 | 3/3 | 2/2 | *this study* |
| 29 | *P. b. unicolor* | DTU 474 | 7 | 3 | 7/7 | 9/9 | 3/3 | 3/3 | 2/2 | 2/2 | *this study* |
| 30 | *P. b. unicolor* | MNHN 1938.0149 | 7 | 3 | 7/7 | 7/7 | 3/3 | 3/3 | 1/1 | 1/1 | *this study* |
| 31 | *P. b. unicolor* | MNHN RA-1937.27 | 7 | 3 | 7/7 | 7/7 | 3/3 | 3/3 | 1/1 | 1/1 | *this study* |
| 32 | *P. b. unicolor* | SIEZC 20216 | 3 | 3 | 7/8 | 7/7 | 3/3 | 4/4 | 2/2 | 1/1 | *this study* |
| 33 | *P. berdmorei* ssp. | NHMUK 62.7.28.8 | 3 | 3 | ?/8 | 8/8 | 3/3 | 3/3 | 3/2 | 1/1 | *this study* |
| 34 | *P. berdmorei* ssp. | MNHN RA-1896.655 | 11 | 3 | 7/8 | 7/8 | 3/3 | 4/4 | 2/2 | 1/0 | *this study* |
| 35 | *P. berdmorei* ssp. | MNHN RA-1896.656 | 11 | 1 | 6/7 | 6/6 | 3/3 | 3/3 | 1/1 | 0/1 | *this study* |
| 36 | *P. berdmorei* ssp. | MNHN RA-1896.657 | 9 | 3 | 7/7 | 7/7 | 3/3 | 4/4 | 1/1 | 0/0 | *this study* |
| 1 | *P. nuchalis* | NHMUK 1912247 | ? | 3 | 8/8 | 8/8 | 3/3 | 4/4 | 1/1 | 1/1 | *this study* |
| 2 | *P. nuchalis* | FMNH 131635 | 0 | 3 | 8/8 | 8/7 | 4/3 | 5/3 | 3/3 | 2/2 | *this study* |
| 3 | *P. nuchalis* | FMNH 239902 | 0 | 1 | 7/7 | 7/7 | 2/3 | 4/4 | 3/1 | 1/1 | *this study* |
| 4 | *P. nuchalis* | FMNH 239903 | 0 | 1 | 8/8 | 7/7 | 4/4 | 4/4 | 3/1 | 1/2 | *this study* |
| 5 | *P. nuchalis* | FMNH 269040 | 0 | 3 | 8/8 | 7/7 | 4/3 | 4/4 | 3/3 | 1/1 | *this study* |
| 6 | *P. nuchalis* | USNM 070863 | 0 | 1 | 8/7 | ? | 3/3 | 3/3 | 1/1 | 1/1 | *this study* |
| 7 | *P. nuchalis* | FMNH 131636 | 0 | 3 | 7/7 | ? | 3/3 | 4/3 | 1/1 | 1/1 | *this study* |
| 8 | *P. nuchalis* | FMNH 269041 | 0 | 3 | 8/8 | 6/7 | 3/3 | 4/3 | 3/3 | 1/1 | *this study* |
| 9 | *P. nuchalis* | ZMH R3971 | 0 | 3 | 8/8 | 8/7 | 3/3 | 4/4 | 2/2 | 1/1 | *this study* |
| 1 | *P. temporalis* | UNS 09992 | 15 | 3 | 9/8 | 8/9 | 4/5 | 3/3 | 2/2 | 2/3 | *Le et al., 2021* |
| 2 | *P. temporalis* | ZMMU R-13656 | 15 | 3 | 7/8 | 7/8 | 3/3 | 4/3 | 2/2 | 1/0 | *this study* |
| 3 | *P. temporalis* | DTU 471 | 15 | 3 | 8/8 | 8/8 | 3/3 | 4/4 | 2/2 | 2/2 | *this study* |
| 4 | *P. temporalis* | DTU 487 | 15 | 3 | 8/7 | 8/8 | 3/3 | 4/4 | 2/2 | 2/2 | *this study* |
| 5 | *P. temporalis* | DTU 488 | 15 | 3 | 8/8 | 8/8 | 3/3 | 4/4 | 2/2 | 2/2 | *this study* |
| 6 | *P. temporalis* | SIEZC 20214 | 15 | 3 | 8/8 | 8/8 | 3/3 | 4/4 | 2/2 | 2/2 | *this study* |
| 7 | *P. temporalis* | SIEZC 20215 | 15 | 3 | 8/8 | 8/8 | 3/3 | 4/4 | 2/2 | 2/2 | *this study* |
