# Supplementary material for: An integrative taxonomic revision of slug-eating snakes (Squamata: Pareidae: Pareineae) reveals unprecedented diversity in Indochina
Source: PeerJ. 2022 Jan 10;10:e12713. doi: 10.7717/peerj.12713 (PMC8757378; doi:10.7717/peerj.12713)
Supplement: Supplemental Information 12 — Minimum (Min), Maximum (Max), and mean values are given; SD – standard deviation, n – sample size. [file peerj-10-12713-s012.docx]

**Supplementary Table S12.** Measurements and meristic characters of subspecies of *Pareas berdmorei* **comb. nov.** and P. carinatus. Minimum (Min), Maximum (Max), and mean values are given; SD – standard deviation, n – sample size.

| **Characters** | | ***Pareas berdmorei* comb. nov.** | | | ***Pareas carinatus*** | |
| --- | --- | --- | --- | --- | --- | --- |
|  | | *P. b. berdmorei*  **comb. et stat. nov** | *P. b. unicolor*  **comb. nov** | *P. b. truongsonicus*  **ssp. nov.** | *P. c. carinatus* | *P. c.* tenasserimicus  **ssp. nov** |
| **TL** | Min–Max | 451–770 | 459–576 | 488–637 | 337–608 | 702 |
|  | Mean±SD | 581.5±73.4 | 516.3±42.5 | 587.0±67.6 | 485.2±59.7 |  |
|  | n | 17 | 9 | 4 | 23 | 1 |
| **TaL/TL** | Min–Max | 0.18–0.27 | 0.17–0.24 | 0.20–0.25 | 0.18–0.25 | 0.25 |
|  | Mean±SD | 0.21±0.02 | 0.20±0.02 | 0.23±0.02 | 0.22±0.02 |  |
|  | n | 17 | 9 | 4 | 23 | 1 |
| **VEN** | Min–Max | 166–186 | 162–180 | 167–187 | 158–190 | 194 |
|  | Mean±SD | 178.1±5.2 | 173.6±5.1 | 179.5±9.6 | 170.4±8.2 |  |
|  | n | 21 | 9 | 4 | 25 | 1 |
| **SC** | Min–Max | 57–89 | 57–75 | 66–80 | 54–84 | 96 |
|  | Mean±SD | 73.2±7.6 | 68.0±6.9 | 74.3±6.2 | 68.1±7.2 |  |
|  | n | 18 | 9 | 4 | 25 | 1 |
| **KMD** | Min–Max | 5–13 | 3–9 | 13 | 3–11 | 7 |
|  | Mean±SD | 9.6±2.2 | 6.8±1.9 |  | 6.5±2.9 |  |
|  | n | 15 | 9 | 2 | 19 | 1 |
| **VSC** | Min–Max | 1–3 | 1–3 | 3 | 3 | 3 |
|  | Mean±SD | 2.9±0.4 | 2.8±0.7 |  | 3.0±0.0 |  |
|  | n | 21 | 9 | 2 | 25 | 1 |
