# Supplementary material for: An integrative taxonomic revision of slug-eating snakes (Squamata: Pareidae: Pareineae) reveals unprecedented diversity in Indochina
Source: PeerJ. 2022 Jan 10;10:e12713. doi: 10.7717/peerj.12713 (PMC8757378; doi:10.7717/peerj.12713)
Supplement: Supplemental Information 13 — Exceptional values are shown in parentheses. Symbol characters are: ① Frontal scale shape: 0= subhexagonal with the lateral sides converging posteriorly, 1=hexagonal with the lateral sides parallel; ② Anterior pair of chin shields: 0= longer than broad, 1= broader than long; ③ Loreal–eye contact: 0= present, 1= absent; ④ Prefrontal–eye contact: 0= present, 1= absent; ⑤ Number of preoculars; ⑥ Number of suboculars; ⑦ Number of postoculars; ⑧ Number of infralabials; ⑨ Number of temporals; ⑩ Number of keeled dorsal scale rows at midbody; ⑪ Number of enlarged vertebral scale rows; ⑫ Ventral scales number; ⑬ Subcaudal scales number. [file peerj-10-12713-s013.docx]

**Supplementary Table S13.** Diagnostic features of scalation and color pattern of *Pareas abros* **sp. nov.** and *P. kuznetsovorum* **sp. nov.** in comparison with the other 24 currently recognized species of the genus *Pareas.* Exceptional values are shown in parentheses. **Symbol characters are:** ① Frontal scale shape: 0= subhexagonal with the lateral sides converging posteriorly, 1=hexagonal with the lateral sides parallel; **②** Anterior pair of chin shields: 0= longer than broad, 1= broader than long; **③** Loreal–eye contact: 0= present, 1= absent; **④** Prefrontal–eye contact: 0= present, 1= absent; **⑤** Number of preoculars; **⑥** Number of suboculars; **⑦** Number of postoculars; **⑧** Number of infralabials; **⑨** Number of temporals; **⑩** Number of keeled dorsal scale rows at midbody; **⑪** Number of enlarged vertebral scale rows; **⑫** Ventral scales number; **⑬** Subcaudal scales number.

| **Species** | **①** | **②** | **③** | **④** | **⑤** | **⑥** | **⑦** | **⑧** | **⑨** | **⑩** | **⑪** |
| --- | --- | --- | --- | --- | --- | --- | --- | --- | --- | --- | --- |
| *P. abros* **sp. nov** | 1 | 1 | 0 | 0 | 1 | 3 | 2 | 8(9) | 3+3 | 9–11 | 1 |
| *P. kuznetsovorum* **sp. nov.** | 1 | 1 | 0 | 0 | 1 | 2 | 1 | 7 | 3+4 | 0 | 1 |
| *P. andersonii* | 0 | 0 | 0 | 1 | 1 | 1 | 1 | 7–8 | 2+3 | 5–9 | 0 |
| *P. atayal* | 0 | 0 | 0 | 1 | 1 | 1 | 1 | 7–9 | 2+4 | 5–9 | 3 |
| *P. berdmorei* **comb. nov.** | 1 | 1 | 0 | 0 | 1 or 2 | 2(1 or 3) | 1(0 or 2) | 7–10 | 3+4 or 3+3 | 3–13 | 1–3 |
| *P. boulengeri* | 0 | 0 | 1 | 1 | 0 | Fused | Fused | 8(7, 9) | 2+3(1+2) | 0 | 0 |
| *P. carinatus* | 1 | 1 | 0 | 0 | 1 | 1–3 | 1(0) | 7–9 | 3+4 or 3+3 | 0–11 | 3 |
| *P. chinensis* | 0 | 0 | 0(1) | 1 | 1 | 1 | 1 | 8(7, 9) | 2+3 | 0(7) | 3 |
| *P. formosensis* | 0 | 0 | 0 | 1 | 1 | 1 | 1 | 6–8 | 2+3 | 0 | 3 |
| *P.* geminatus | 0 | 0 | 0 | 1 | 1 | Fused | Fused | 8 | 1+2 or 2+3 | 3–5 | 1 |
| *P. hamptoni* | 0 | 0 | 0 | 1 | 1 | 1 or Fused | 1 or Fused | 6–9 | 1+2 or 2+3 | 5–9 | 1 |
| *P. iwasakii* | 0 | 0 | 0 | 1 | 1 | 1 | 1 | 9–11 | 3+4 or 2+3 | 5–7 | 1 |
| *P. komaii* | 0 | 0 | 0 | 1 | 1 | 1 | 1 | 6–9 | 3+4 or 2+3 | 9–13 | 3 |
| *P. kaduri* | 0 | 0 | 0(1) | 1 | 1 | 1 | 1 | 7 | 2+3 | 5–8 | 1 |
| *P. macularius* | 0 | 0 | 0 | 1 | 1 | 1 | 1 | 7 | 2+3 | 7–13 | 0 |
| *P. margaritophorus* | 0 | 0 | 0 | 1 | 1 | 1 | 1 | 7 | 2+3 | 0 | 0 |
| *P. modestus* | 0 | 0 | 0 | 1 | 1 | 1 | 1 | 7 | 2+3 | 3–5 | 0 |
| *P. monticola* | 0 | 0 | 1 | 1 | 1 | 1 | 1 | 7–8 | 2+3 | 0 | 1–3 |
| *P. niger* | 0 | 0 | 0 | 1 | 1 | Fused | Fused | 7(8) | 2+3 | 3–9 | 1–3 |
| *P. nigriceps* | 0 | 0 | 0 | 0 or 1 | 1 | Fused | Fused | 7 | 1+2 or 1+3 | 5–9 | 1 |
| *P. nuchalis* | 1 | 1 | 0 | 1 | 1 | 1–3 | 1–2 | 7(6, 8) | 3+3 or 3+4 | 0 | 1–3 |
| *P. temporalis* | 1 | 1 | 0 | 0 | 1 | 2 | 2(1 or 0) | 8(7) | 3+3(3+4) | 15 | 3 |
| *P. stanleyi* | 0 | 0 | 1 | 1 | 0 | 1 | 1 | 7(8) | 2+2 or 2+3 | 13 | 0 |
| *P. victorianus* | 0 | 0 | 1 | 1 | 0 | 1 or Fused | 1 or Fused | 6 or 7 | 2+3 | 7 | 1 |
| *P. vindumi* | 0 | 0 | 1 | 1 | 1 | 0 | 1 | 6 | 2+3 | 11 | 0 |
| *P. xuelinensis* | 0 | 0 | 0 | 1 | 1 | Fused | Fused | 7 | 2+2 | 3–5 | 0 |
| *P.* cf. *yunnanensis* | 0 | 0 | 0 | 1 | 1 | Fused | Fused | 7 (8 or 6) | 1+2 or 2+3 | 3–7 | 1–3 |

(Continues on next page)

**Supplementary Table S13**. (Continued).

| **Species** | **⑫** | **⑬** | Head and neck pattern | **Distribution** | **Sources** |
| --- | --- | --- | --- | --- | --- |
| *P. abros* **sp. nov** | 180–184 | 185–198 | A thin postorbital stripe from postocular to neck, ring-shaped blotch on nuchal area | Vietnam | 16 |
| *P. kuznetsovorum* **sp. nov.** | 167 | 87 | A thin postorbital stripe from postocular to neck, large black blotch on nuchal area | Vietnam | 16 |
| *P. andersonii* | 141–162 | 35–47 | No markings on the head, no collar | Myanmar, India, China | 9, 16 |
| *P. atayal* | 174–188 | 71–79 | Two black lines from postorbital, lower reaching mouth angle, upper going behind head basis and contacting with short black line on neck | Taiwan | 5, 16 |
| *P. berdmorei* **comb. nov.** | 162–187 | 57–89 | A thin postorbital stripe extending from postocular to neck | Myanmar, China, Indochina, Thailand | 16 |
| *P. boulengeri* | 164–187 | 63–78 | A black line from behind eye to angle of mouth | China | 16 |
| *P. carinatus* | 158–194 | 54–96 | A black line from eye to nape, and another from behind eye to angle of mouth | Thailand, Malaysia, Indonesia | 16 |
| *P. chinensis* | 169–180 | 69–76 | A black line from eye extending along nape, and another from the eye to the angle of the mouth | China | 1, 16 |
| *P. formosensis* | 170–180 | 69–82 | A black line from rear of the supraocular to neck, and another from lower anterior-temporal to angle of mouth | Taiwan, China, Vietnam, Laos | 10, 16 |
| *P.* geminatus | 170–188 | 67–91 | Two black longitudinal streaks on the back of the head and nape | China, Laos, Thailand^?^ | 10, 16 |
| *P. hamptoni* | 185–195 | 91–99 | Two black longitudinal streaks on the back of the head and nape | Myanmar, China | 10, 16 |
| *P. iwasakii* | 189–194 | 76–84 | A vertical black line from behind eye to neck, another line from behind eye to angle of mouth and to chin | Japan | 5, 16 |
| *P. komaii* | 162–182 | 60–76 | Two black lines from postorbital, lower reaching mouth angle, upper going behind head basis and contacting with short black line on neck | Taiwan | 5, 16 |
| *P. kaduri* | 160–183 | 52–71 | Dense dark dusting all over the head, postocular stripes absent, vertical black line head basis | India, Myanmar | 11, 13, 16 |
| *P. macularius* | 151–173 | 39–53 | A pink, cream or yellow entire or tripartite collar or spot without fine brown speckling | India, China, Myanmar, Thailand,  Laos, Cambodia, Vietnam | 9, 16 |
| *P. margaritophorus* | 133–160 | 35–54 | A butterfly or W shaped collar with moderate or dense speckling | China, Myanmar, Laos, Cambodia, Vietnam , Thailand, Malaysia, Indonesia, Singapore | 9, 16 |
| *P. modestus* | 151–159 | 35–46 | No markings on the head, no collar | Myanmar, India | 9,16 |
| *P. monticola* | 178–199 | 69–90 | A black line from eye to nape, and another from behind eye to angle of mouth | India, China, Vietnam | 13, 16 |
| *P. nigriceps* | 175–184 | 73–77 | A big black oval patch on back of head, two round black spots on each side of head, a black nuchal band | China | 3, 16 |
| *P. niger* | 154–172 | 54–66 | A large black area on the back of head, two black spots on each side of head | China | 12, 16 |
| *P. nuchalis* | 201–220 | 102–120 | Oblique black line from lower corner of eye to front edge of last upper labial, and usually a thin, vertical black line at rear of head | Indonesia, Malaysia | 16 |
| *P. stanleyi* | 151–160 | 48–60 | A big black spot on the back of the head which separates into two vertical black lines behind the neck; a black line from behind the eye to the nape | China | 2, 16 |
| *P. temporalis* | 83–95 | 86–92 | A thick postorbital stripe from postocular to neck, ring-shaped blotch on nuchal area | Vietnam | 15; 16 |
| *P. victorianus* | 164 | 58 | A black line from eye to nape, and another from behind eye to angle of mouth | Myanmar | 13, 16 |
| *P. vindumi* | 173–175 | 60–61 | No markings on the head, no collar | Myanmar, China | 6, 14 |
| *P. xuelinensis* | 182–197 | 68–93 | Two black longitudinal streaks on the back of the head and nape | China, Thailand | 12, 16 |
| *P.* cf. *yunnanensis* | 160–175 | 57–65 | A large black area on the back of head, two black spots on each side of head | China | 16 |

**Sources**: 1= Jiang (2004); 2= Guo *et al*. (2004); 3= Guo & Deng(2009); 4= Stuebing *et al*. (2014); 5= You *et al*. (2015); 6= Vogel (2015); 7= Yang *et al*. (2019); 8= Wang *et al*. (2020); 9= Vogel *et al*. (2020); 10= Ding *et al.* (2020); 11= Bhosale *et al.* (2020); 12= Liu & Rao (2021); 13= Vogel *et al*. (2021); 14= Yang *et al*. (2021); 15= Le *et al.* (2021); 16=our data.
