# Supplementary material for: An integrative taxonomic revision of slug-eating snakes (Squamata: Pareidae: Pareineae) reveals unprecedented diversity in Indochina
Source: PeerJ. 2022 Jan 10;10:e12713. doi: 10.7717/peerj.12713 (PMC8757378; doi:10.7717/peerj.12713)
Supplement: Supplemental Information 14 — Question mark (?) denotes tentative identifications; asterisk (*) denotes type species; n-dash (—) denotes missing data. [file peerj-10-12713-s014.docx]

**Supplementary Table S14.** Comparisons of morphological characteristics of Pareinae genera and subgenera. Question mark (?) denotes tentative identifications; asterisk (*) denotes type species; n-dash (—) denotes missing data.

| **Characteristics** | ***Aplopeltura*** | ***Asthenodipsas*** | | ***Pareas*** | |
| --- | --- | --- | --- | --- | --- |
|  |  | ***Asthenodipsas***  (senior synonym of *Internatus*) | ***Spondylodipsas***  **subgen. nov.** | ***Pareas*** | ***Eberhardtia***  **stat. nov.** |
| Dorsal scale rows at midbody | 13 | 15 | 15 | 15 | 15 |
| Dorsal scales | smooth | smooth | smooth | keeled (except *P. kuznetsovorum* **sp. nov.**) | smooth or keeled |
| Vertebral keel | weak | developed | developed | weak | weak or absent |
| Number of loreals | 2 or 3 | 1 | 1 | 1 (2) | 1 |
| Number of anterior temporals | 3 | 2 | 2 | 3 | 2 or 1 |
| Shape of frontal scale | subhexagonal with the lateral sides converging posteriorly | subhexagonal with the lateral sides converging posteriorly | subhexagonal with the lateral sides converging posteriorly | hexagonal with the lateral sides parallel | subhexagonal with the lateral sides converging posteriorly |
| Structure  of chin scales | inframaxillary shield absent; 3 or 4 pairs of chin shields; 1^st^ and 2^nd^ pairs of infralabials in contact | 1 inframaxillary shield;  2 pairs of chin shields; 3^rd^ pair of infralabials in contact | 1 inframaxillary shield;  3 pairs of chin shields;  1^st^ pair of infralabials in contact | inframaxillary shield absent; 3 pairs of chin shields; 1^st^ pairs of infralabials in contact | inframaxillary shield absent; 3 pairs of chin shields; 1^st^ pairs of infralabials in contact |
| Anterior pair of chin shields | broader than long | as broad as long | as broad as long | broader than long | longer than broad |
| Subcaudals | undivided | divided | divided | divided | divided |
| Suboculars | present | absent | absent | present | present |
| Supralabials in contact with the eye | no | yes | yes | no | no (except *P*. *monticola*,  *P*. *stanleyi*) |
| Ultrastructure of dorsal scales | — | — | — | ravine-like | with pores and arc structures, forming characteristic lines |
| Content | 1. *A. boa** | 1. *A. borneensis*  2. *A. jamilinaisi*  3. *A. laevis*  4. *A. malaccana**  (senior synonym of *Pareas dorsopictus* and *Amblycephalus malaccanus ventrilineatus*)  5. *A. stuebingi* | 1. *A. lasgalenensis*  2. *A. tropidonota**  3. *A. vertebralis* | 1. *P. abros* **sp. nov.**  2. *P.* *berdmorei*  (senior synonym of  *Pareas menglaensis*)  3. *P. carinatus**  4. *P. nuchalis*  5. *P. temporalis*  6. *P. kuznetsovorum* **sp. nov.** | 1. *P. andersonii*  2. *P. atayal*  3. *P. boulengeri*  4. *P. chinensis* (senior synonym of *Amblycephalus yunnanensis^?^*)  5. *P. formosensis*  (senior synonym of  *Psammodynastes compressus*,  *Eberhardtia tonkinensis*, *Amblycephalus carinatus hainanus*,  *Amblycephalus kuangtungensis*)  6. *P. geminatus*  7. *P. hamptoni**  8*. P. iwasakii*  9*. P. kaduri*  10*. P. komaii*  11. *P. macularius*  12. *P. margaritophorus*  (senior synonym of  *Pareas moellendorffi*,  *Amblycephalus tamdaoensis*)  13. *P. modestus*  14. *P. monticola*  15. *P. niger* (senior synonym of  *Pareas mengziensis*)  16. *P. nigriceps*  17. *P. stanleyi*  18. *P. victorianus*  19. *P. vindumi*  20. *P. xuelinensis^?^* |
