# Supplementary material for: An integrative taxonomic revision of slug-eating snakes (Squamata: Pareidae: Pareineae) reveals unprecedented diversity in Indochina
Source: PeerJ. 2022 Jan 10;10:e12713. doi: 10.7717/peerj.12713 (PMC8757378; doi:10.7717/peerj.12713)
Supplement: Supplemental Information 15 — Abbreviations are listed in the Materials and methods. Other abbreviations: ASR= number of dorsal scales at neck; MSR= number of dorsal scales at midbody; PSR= number of dorsal scales at vent. (?= not available). [file peerj-10-12713-s015.docx]

**Supplementary Table S15.** Measurements and scale counts of members of genera *Aplopeltura* (*Ap. boa*); *Asthenodipsas* (including *As. borneensis*, *As. laevis*, *As. lasgalensis*, *As. malaccana*, *As. stuebingi*, *As*. *tropidonota*, and *As. vertebralis*)*.* Abbreviations are listed in the Materials and methods. Other abbreviations: ASR= number of dorsal scales at neck; MSR= number of dorsal scales at midbody; PSR= number of dorsal scales at vent. (?= not available). (Continues on the next page)

| **Species** | **Type** | **Voucher number** | **Locality** | **Sex** | **SVL** | **TaL** | **VEN** | **SC** |
| --- | --- | --- | --- | --- | --- | --- | --- | --- |
| *Ap. boa* | 0 | ZSM 363.1920 | Nias, Indonesia | F | 338 | 73 | 151 | 104 |
| *Ap. boa* | 0 | ZSM 512.1909 | Batang Gadis, Sumatra, Indonesia | F | 464 | ? | 162 | 108 |
| *As. borneensis* | 0 | ZSM 143.1907 | Kalimata, Borneo, Indonesia | M | 372 | 73 | 164 | 45 |
| *As. laevis* | 0 | FMNH 273617 | Bintulu, Sarawak, Malaysia | F | 348 | 52 | 167 | 38 |
| *As. laevis* | 0 | NMW 13382 | Padang, Sumatra | F | 338 | 60 | 169 | 43 |
| *As. laevis* | 0 | NMW 21823.2 | Padang, Sumatra | F | 305 | 53 | 163 | 43 |
| *As. laevis* | 0 | NMW 28123.5 | Sarawak, Borneo, Malaysia | M | 321 | 75 | 158 | 49 |
| *As. laevis* | Lectotype | RMNH 986B | Java, Indonesia | F | 326 | 48 | 165 | 36 |
| *As. laevis* | Paralectotype | RMNH 986A | Java, Indonesia | F | 292 | 42 | 164 | 37 |
| *As. laevis* | Paralectotype | RMNH 986C | Java, Indonesia | F | 144 | 28 | 149 | 44 |
| *As. laevis* | 0 | SMF 81195 | Sumatra, Indonesia | M | 250 | 52 | 166 | 44 |
| *As. laevis* | 0 | SMF 20788 | Java, Indonesia | M | 298 | 61 | 158 | 42 |
| *As. laevis* | 0 | SMF 20789 | Java, Indonesia | M | 111 | ? | 151 | 46 |
| *As. laevis* | 0 | ZMA 16245 | Banka, Indonesia | M | 314 | 74 | 154 | 46 |
| *As. laevis* | 0 | ZMB 2728 | Bukit Lawang, Sumatra, Indonesia | F | 292 | 45 | 165 | 38 |
| *As. laevis* | 0 | ZMB 5231 | Java, Indonesia | M | 241 | 46 | 153 | 40 |
| *As. laevis* | 0 | ZMB 14159 | Malang, Java, Indonesia | M | 258 | 51 | 155 | 41 |
| *As. laevis* | 0 | ZSM 126.1947 | Indonesia | F | 272 | 45 | 161 | 40 |
| *As. lasgalensis* | 0 | ZFMK 53098 | Cameron Highlands, Pahang, Malaysia | M | 536 | 141 | 186 | 70 |
| *As. lasgalensis* | 0 | ZMB 49675 | Cameron Highlands, Pahang, Malaysia | F | 425 | 89 | 183 | 58 |
| *As. lasgalensis* | 0 | ZMB 55198 | Cameron Highlands, Pahang, Malaysia | F | 565 | 142 | 184 | 67 |
| *As. lasgalensis* | 0 | ZMB 57111 | Cameron Highlands, Pahang, Malaysia | F | 495 | 131 | 181 | 62 |
| *As. lasgalensis* | 0 | ZMB 57112 | Cameron Highlands, Pahang, Malaysia | M | 565 | 143 | 183 | 71 |
| *As. malaccana* | Syntype of *P. m. ventrilineatus* | MNHN 1939.201 | Batavia, Java, Indonesia | M | 304 | 51 | 154 | 34 |
| *As. malaccana* | Syntype of *P. m ventrilineatus* | MNHN 1939.202 | Batavia, Java, Indonesia | M | 299 | 51 | 154 | 33 |
| *As. malaccana* | Syntype of *P. m. ventrilineatus* | MNHN 1939.203 | Batavia, Java, Indonesia | F | 236 | 21 | 166 | 23 |
| *As. malaccana* | Syntype of *P. m. ventrilineatus* | MNHN 1939.204 | Batavia, Java, Indonesia | M | 266 | 38 | 157 | 32 |
| *As. malaccana* | 0 | NMW 28126.1 | West Sumatra, Indonesia | M | 336 | 55 | 152 | 34 |
| *As. malaccana* | 0 | NMW 28126.2 | West Sumatra, Indonesia | M | 320 | 49 | 155 | 33 |
| *As. malaccana* | 0 | SMF 32580 | Perak, Malaysia | M | 347 | 62 | 164 | 41 |
| *As. malaccana* | 0 | ZMB 3592 | Bengkulu, Sumatra, Indonesia | M | 318 | 50 | 157 | 34 |
| *As. malaccana* | Lectotype | ZMB 5041 | Malacca, Malaysia | F | 192 | 26 | 153 | 26 |
| *As. malaccana* | 0 | ZMB 50673 | Thung Song, Nakhon Si Thammarat, Thailand | F | 359 | 35 | ? | 26 |
| *As. stuebingi* | 0 | ZMB 65429 | Mt. Kinabalu, Borneo, Malaysia | F | 492 | 74 | 174 | 35 |
| *As. tropidonota* | 0 | NMW 28126.3 | Padang, Sumatra, Indonesia | M | 531 | 120 | 196 | 67 |
| *As. tropidonota* | 0 | NMW 28126.4 | Padang, Sumatra, Indonesia | F | 552 | 118 | 198 | 65 |
| *As. tropidonota* | 0 | ZMB 1816 | Mt. Pesogi, Lampung, Sumatra, Indonesia | M | 501 | 133 | 206 | 78 |
| *As. tropidonota* | 0 | ZMB 3725 | Kabu Peraku, Lampung, Sumatra, Indonesia | M | ? | ? | 203 | ? |
| *As. tropidonota* | 0 | Padang University | Sumatra, Indonesia | M | 366 | 93 | 198 | 75 |
| *As. vertebralis* | 0 | ZMB 52072 | Fraser’s Hill, Pahang, Malaysia | M | 573 | 151 | 201 | 77 |

**Supplementary Table S14.** (Continued).

| **Species** | **Voucher number** | **ASR** | **MSR** | **PSR** | **KMD** | **VSE** | **SL** | **IL** | **At** | **Pt** | **SoO** | **PoO** |
| --- | --- | --- | --- | --- | --- | --- | --- | --- | --- | --- | --- | --- |
| *Ap. boa* | ZSM 363.1920 | 13 | 13 | 13 | 0 | 1 | 9/9 | 8/8 | 5/4 | 5/4 | 5/3 | 1/1 |
| *Ap. boa* | ZSM 512.1909 | 13 | 13 | 13 | 0 | 1 | 9/9 | 8/8 | 2/3 | 4/3 | 3/2 | 1/1 |
| *As. borneensis* | ZSM 143.1907 | 15 | 15 | 15 | 0 | develop | 6/6 | 4/4 | 2/2 | 2/3 | 0/0 | 2/1 |
| *As. laevis* | FMNH 273617 | 15 | 15 | 13 | 0 | develop | 7/6 | 4/5 | 2/2 | 1/2 | 0/0 | 2/2 |
| *As. laevis* | NMW 13382 | 15 | 15 | 13 | 0 | develop | 6/6 | 5/5 | 2/2 | 2/2 | 0/0 | 2/2 |
| *As. laevis* | NMW 21823.2 | 15 | 15 | ? | 0 | develop | 6/6 | 5/5 | 2/2 | 2/2 | 0/0 | 2/2 |
| *As. laevis* | NMW 28123.5 | 15 | 15 | 13 | 0 | develop | 6/6 | 4/4 | 2/2 | 2/2 | 0/0 | 1/1 |
| *As. laevis* | RMNH 986B | 15 | 15 | 13 | 0 | develop | 6/6 | 5/5 | 2/2 | 2/2 | 0/0 | 1/2 |
| *As. laevis* | RMNH 986A | 15 | 15 | 13 | 0 | develop | 6/6 | 4/4 | 2/2 | 2/2 | 0/0 | 1/1 |
| *As. laevis* | RMNH 986C | 15 | 15 | 13 | 0 | develop | 6/6 | 5/5 | 2/2 | 2/2 | 0/0 | 1/1 |
| *As. laevis* | SMF 81195 | 15 | 15 | 13 | 0 | develop | 6/6 | ? | 2/2 | 2/2 | 0/0 | 1/1 |
| *As. laevis* | SMF 20788 | 15 | 15 | 13 | 0 | develop | 6/6 | 5/5 | 2/2 | 2/2 | 0/0 | 2/1 |
| *As. laevis* | SMF 20789 | 15 | 15 | 13 | 0 | develop | 6/6 | ? | 2/2 | 2/2 | 0/0 | 0/0 |
| *As. laevis* | ZMA 16245 | 15 | 15 | 13 | 0 | develop | 6/6 | 5/5 | 2/2 | 2/2 | 0/0 | 2/1 |
| *As. laevis* | ZMB 2728 | 15 | 15 | 13 | 0 | develop | 6/6 | 5/5 | 2/2 | 2/2 | 0/0 | 1/1 |
| *As. laevis* | ZMB 5231 | 15 | 15 | 13 | 0 | develop | 6/6 | 4/4 | 2/2 | 2/2 | 0/0 | 1/1 |
| *As. laevis* | ZMB 14159 | 15 | 15 | 13 | 0 | develop | 7/7 | 6/6 | 1/2 | 2/2 | 0/0 | 1/1 |
| *As. laevis* | ZSM 126.1947 | 15 | 15 | 13 | 0 | develop | 6/6 | 4/4 | 2/2 | 2/2 | 0/0 | 1/1 |
| *As. lasgalensis* | ZFMK 53098 | 15 | 15 | 15 | 0 | develop | 7/7 | 7/7 | 3/2 | 2/3 | 0/0 | 2/2 |
| *As. lasgalensis* | ZMB 49675 | 15 | 15 | 15 | 0 | develop | 7/7 | 5/6 | 2/2 | 2/2 | 0/0 | 2/2 |
| *As. lasgalensis* | ZMB 55198 | 15 | 15 | 15 | 0 | develop | 7/7 | 7/6 | 2/2 | 2/2 | 0/0 | 2/2 |
| *As. lasgalensis* | ZMB 57111 | 15 | 15 | 15 | 0 | develop | 7/7 | 7/7 | 2/2 | 2/2 | 0/0 | 2/2 |
| *As. lasgalensis* | ZMB 57112 | 15 | 15 | 15 | 0 | develop | 7/7 | 6/6 | 2/2 | 2/2 | 0/0 | 2/2 |
| *As. malaccana* | MNHN 1939.201 | 15 | 15 | 15 | 0 | develop | 7/7 | 4/4 | 2/2 | 1/1 | 0/0 | 2/2 |
| *As. malaccana* | MNHN 1939.202 | 15 | 15 | 15 | 0 | develop | 7/7 | 5/5 | 2/2 | 2/2 | 0/0 | 2/2 |
| *As. malaccana* | MNHN 1939.203 | 15 | 15 | 15 | 0 | develop | 7/7 | 4/4 | 2/1 | 2/2 | 0/0 | 1/2 |
| *As. malaccana* | MNHN 1939.204 | 15 | 15 | 15 | 0 | develop | 7/7 | 4/4 | 2/2 | 2/2 | 0/0 | 2/2 |
| *As. malaccana* | NMW 28126.1 | 15 | 15 | 15 | 0 | develop | 7/7 | 4/4 | 2/1 | 2/2 | 0/0 | 2/2 |
| *As. malaccana* | NMW 28126.2 | 15 | 15 | 15 | 0 | develop | 7/7 | 4/4 | 2/2 | 2/2 | 0/0 | 2/2 |
| *As. malaccana* | SMF 32580 | 15 | 15 | 15 | 0 | develop | 7/7 | 6/6 | 3/2 | 3/2 | 0/0 | 2/2 |
| *As. malaccana* | ZMB 3592 | 15 | 15 | 15 | 0 | develop | 7/7 | 4/4 | 2/2 | 1/1 | 0/0 | 2/2 |
| *As. malaccana* | ZMB 5041 | 15 | 15 | 15 | 0 | develop | 7/7 | 5/5 | 2/2 | 1/2 | 0/0 | 2/2 |
| *As. malaccana* | ZMB 50673 | 15 | 15 | 15 | 0 | develop | 6/7 | 4/4 | 2/2 | 1/2 | 0/0 | 2/2 |
| *As. stuebingi* | ZMB 65429 | 15 | 15 | 15 | 0 | develop | 6/6 | 4/4 | 2/2 | 2/2 | 0/0 | 2/2 |
| *As. tropidonota* | NMW 28126.3 | 15 | 15 | 15 | 0 | develop | 7/7 | 6/6 | 3/2 | 2/2 | 0/0 | 2/2 |
| *As. tropidonota* | NMW 28126.4 | 15 | 15 | 15 | 0 | develop | 7/7 | 6/6 | 2/2 | 2/2 | 0/0 | 2/2 |
| *As. tropidonota* | ZMB 1816 | 15 | 15 | 15 | 0 | develop | 7/7 | ? | 2/2 | 2/2 | 0/0 | 2/2 |
| *As. tropidonota* | ZMB 3725 | 15 | 15 | 15 | 0 | develop | 7/7 | 5/5 | 2/2 | 2/2 | 0/0 | 2/2 |
| *As. tropidonota* | Padang University | 15 | 15 | 15 | 0 | develop | 7/7 | 6/6 | 2/2 | 2/2 | 0/0 | 2/2 |
| *As. vertebralis* | ZMB 52072 | 15 | 15 | 15 | 0 | develop | 7/7 | 6/6 | 2/2 | 2/2 | 0/0 | 2/1 |
