# Supplementary material for: An integrative taxonomic revision of slug-eating snakes (Squamata: Pareidae: Pareineae) reveals unprecedented diversity in Indochina
Source: PeerJ. 2022 Jan 10;10:e12713. doi: 10.7717/peerj.12713 (PMC8757378; doi:10.7717/peerj.12713)
Supplement: Supplemental Information 16 — Abbreviations are listed in the Materials and methods. Other abbreviations: Lor–E: Loreal contact with eye (0= no; 1= yes); SL–E: supralabial contact with eye (0= no; 1= yes); ? = not available. [file peerj-10-12713-s016.docx]

**Supplementary Table S16.** Measurements and scale counts of members of the subgenus *Eberhardtia*. Abbreviations are listed in the Materials and methods. Other abbreviations: Lor–E: Loreal contact with eye (0= no; 1= yes); SL–E: supralabial contact with eye (0= no; 1= yes); ? = not available.

(Continues on the next page).

| **#** | ***Species*** | **Type** | **Voucher number** | **Locality** | **Sex** | **SVL** | **TaL** | **KMD** | **VSC** | **VEN** | **SC** |
| --- | --- | --- | --- | --- | --- | --- | --- | --- | --- | --- | --- |
| **1** | *P. andersonii* | 0 | CAS 233330 | Haka, Chin, Myanmar | F | 407 | 74 | 5 | 0 | 155 | 36 |
| **2** | *P. andersonii* | 0 | CAS 235218 | Chin, Myanamar | F | 367 | 67 | ? | 0 | 155 | 41 |
| **3** | *P. andersonii* | 0 | CAS 235359 | Chin, Myanmar | M | 266 | 56 | ? | 0 | 153 | 46 |
| **4** | *P. andersonii* | 0 | CAS 241270 | Kachin, Myanmar | F | 271 | 42 | ? | 0 | 160 | 35 |
| **5** | *P. andersonii* | 0 | CAS 245296 | Saguing, Sagian, Myanmar | M | 346 | 70 | ? | 0 | 153 | 42 |
| **6** | *P. andersonii* | 0 | CAS 245377 | Saguing, Sagian, Myanmar | M | 307 | 58 | ? | 0 | 153 | 41 |
| **7** | *P. andersonii* | 0 | MZMU 916 | Mizoram, India | F | 297 | 53 | 5 | 0 | 156 | 40 |
| **8** | *P. andersonii* | 0 | NHMUK 19121115 | Kyatpyin, Mandalay, Myanmar | F | 382 | 76 | 5 | 0 | 159 | 45 |
| **9** | *P. andersonii* | 0 | NHMUK 1912117b | Mogok, Mandalay, Myanmar | M | 332 | 73 | 7 | 0 | 152 | 47 |
| **10** | *P. andersonii* | 0 | NHMUK 1912117a | Mogok, Mandalay, Myanmar | F | 381 | 65 | 7 | 0 | 159 | 40 |
| **11** | *P. andersonii* | 0 | NHMUK 1912119 | Myanmar | F | 387 | 69 | 9 | 0 | 162 | 43 |
| **12** | *P. andersonii* | 0 | NHMUK 430232 | Shweli, Mongmit, Shan, Myanmar | M | 233 | 45 | 5 | 0 | 144 | 40 |
| **13** | *P. andersonii* | 0 | NHMUK 430234 | Kalaw, Taunggyi, Shan, Myanmar | M | 262 | 57 | 7 | 0 | 141 | 42 |
| **1** | *P. atayal* | 0 | FMNH 127998 | Yangminshan N.P., Taiwan | M | 149 | 42 | 3 | 3 | 168 | 68 |
| **2** | *P. atayal* | 0 | FMNH 169315 | Yangminshan N.P., Taiwan | F | 502 | 131 | 7 | 1 | 183 | 70 |
| **3** | *P. atayal* | 0 | FMNH 169392 | Yangminshan N.P., Taiwan | M | 382 | 112 | 5 | 3 | 177 | 71 |
| **4** | *P. atayal* | 0 | FMNH 169395 | Yangminshan N.P., Taiwan | F | 516 | 143 | 7 | 3 | 182 | 63 |
| **5** | *P. atayal* | 0 | NMW 28130.17 | Taiwan | F | 340 | 90 | 7 | 3 | 168 | 64 |
| **6** | *P. atayal* | Paratype | ZMMU R-14435 | Taoyuan, Taiwan | M | 421 | 136 | 7 | ? | 178 | 78 |
| **1** | *P. boulengeri* | 0 | CIB 10084 | Leishan, Guizhou, China | F | 482 | 122 | 0 | 1 | 182 | 65 |
| **2** | *P. boulengeri* | 0 | DL 026 | Ebian, Sichuan, China | F | 391 | 112 | 0 | 1 | 177 | 74 |
| **3** | *P. boulengeri* |  | DL 027 | Ebian, Sichuan, China | F | 377 | 96 | 5 | 1 | 172 | 63 |
| **4** | *P. boulengeri* | 0 | DL 2018.06.29.01 | Xianju, Zhejiang, China | F | 484 | 136 | 0 | 3 | 183 | 73 |
| **5** | *P. boulengeri* | 0 | DL 2018.06.29.02 | Xianju, Zhejiang, China | F | 398 | 110 | 5 | 1 | 172 | 68 |
| **6** | *P. boulengeri* | 0 | DL 2018.08.10.01 | Xianju, Zhejiang, China | F | 465 | 131 | 0 | 3 | 182 | 71 |
| **7** | *P. boulengeri* | 0 | DL 2019.08.16.01 | Jiangcheng, Yangjiang, Guangdong, China | F | 465 | 131 | 0 | 3 | 182 | 71 |
| **8** | *P. boulengeri* | 0 | DL 2019.09.23.04 | Xingou, Yaan, Sichuan, China | F | 452 | 121 | 5 | 0 | 172 | 63 |
| **9** | *P. boulengeri* | Syntypes | MNHN 1912.349 | Guizhou, China | M | 381 | 105 | 0 | 0 | 178 | 68 |
| **10** | *P. boulengeri* | Syntypes | MNHN 1912.350 | Guizhou, China | F | 152 | ? | 0 | 0 | 173 | ? |
| **11** | *P. boulengeri* | Syntypes | MNHN 1912.351 | Guizhou, China | F | 378 | 92 | 0 | 0 | 184 | 63 |
| **1** | *P. chinensis* | 0 | DL 051 | Mt. Jiguan, Sichuan, China | M | 391 | 120 | ? | 0 | 169 | 74 |
| **2** | *P. chinensis* | 0 | FMNH 170632 | Sichuan, China | F | 373 | 106 | 0 | 3 | 175 | 71 |
| **3** | *P. chinensis* | 0 | FMNH 232812 | Hongya, Sichuan, China | F | 427 | 113 | 0 | 3 | 176 | 69 |
| **4** | *P. chinensis* | 0 | FMNH 232813 | Hongya, Sichuan, China | F | 407 | 112 | 0 | 3 | 174 | 70 |
| **5** | *P. chinensis* | 0 | FMNH 232814 | Hongya, Sichuan, China | F | 426 | 122 | 0 | 3 | 178 | 73 |
| **6** | *P. chinensis* | 0 | NMW 39540.1 | Mt. Jiguan, Sichuan, China | M | 386 | 128 | 5 | 3 | 176 | 76 |
| **7** | *P. chinensis* | 0 | NMW 39540.2 | Mt. Jiguan, Sichuan, China | M | 309 | 99 | 5 | 3 | 176 | 75 |
| **1** | *P. formosensis* | Holotype of *Amblycephalus carinatus hainanus* | BMNH 1924.5.22.11 | Mt. Wuzhi, Hainan, China | M | 384 | 130 | 0 | 1 | 193 | 93 |
| **2** | *P. formosensis* | 0 | CIB 10145 | Mt. Wuyishan, Fujian, China | F | 415 | 120 | 0 | 3 | 183 | 79 |
| **3** | *P. formosensis* | 0 | CIB 10147 | Mt. Wuyishan, Fujian, China | M | 402 | 131 | 0 | 3 | 181 | 83 |
| **4** | *P. formosensis* | 0 | CIB GX201304417 | Mt. Shiwan, Guangxi, China | F | 401 | 119 | 0 | 3 | 185 | 81 |
| **5** | *P. formosensis* | 0 | DTU 488 | Gung Re, Di Linh, Lam Dong | F | 391 | 109 | 5 | 1 | 192 | 80 |
| **6** | *P. formosensis* | 0 | DTU 489 | Gung Re, Di Linh, Lam Dong | F | 370 | 107 | 5 | 1 | 188 | 81 |
| **7** | *P. formosensis* | 0 | FMNH 24988 | Mt. Wuyishan, Fujian, China | M | 260 | ? | 0 | 3 | 185 | 83 |
| **8** | *P. formosensis* | 0 | FMNH 24989 | Mt. Wuyishan, Fujian, China | M | 434 | 146 | 0 | 1 | 181 | 86 |
| **9** | *P. formosensis* | 0 | FMNH 255567 | Pu Mat, Nghe An, Vietnam | F | 437 | 133 | 0 | 3 | 188 | 84 |
| **10** | *P. formosensis* | Holotype of *Eberhardtia tonkinensis* | MNHN 1908.206 | Sa Pa, Lao Cai, Vietnam | F | 403 | 129 | 0 | 1 | 194 | 88 |
| **11** | *P. formosensis* | 0 | NMW 39665.1 | Tam Dao, Vinh Phuc, Vietnam | M | 321 | 103 | 0 | 1 | 191 | 91 |
| **12** | *P. formosensis* | 0 | NHMUK 1912148 | Sa Pa, Lao Cai, Vietnam | F | 381 | 126 | 0 | 3 | 187 | 82 |
| **13** | *P. formosensis* | Paratype of *Amblycephalus carinatus hainanus* | NHMUK 1912152 | Mt. Wuzhi, Hainan, China | F | 361 | 109 | 0 | 3 | 190 | 83 |
| **14** | *P. formosensis* | 0 | NHMUK 1912153 | Kon Tum, Vietnam | F | 455 | 152 | 0 | 1 | 195 | 99 |
| **15** | *P. formosensis* | 0 | NHMUK 1912160a | Hong Kong, China | F | 347 | 99 | 0 | 1 | 180 | 75 |
| **16** | *P. formosensis* | 0 | NHMUK 1912160b | Hong Kong, China | F | 370 | 97 | 0 | 1 | 178 | 71 |
| **17** | *P. formosensis* | 0 | NHMUK 1912160c | Hong Kong, China | F | 292 | 77 | 0 | 3 | 175 | 73 |
| **18** | *P. formosensis* | 0 | NMW 28130.12 | Taiwan | F | 459 | 130 | 0 | 3 | 181 | 75 |
| **19** | *P. formosensis* | 0 | NMW 28130.14 | Taiwan | M | 375 | 118 | 0 | 1 | 168 | 74 |
| **20** | *P. formosensis* | 0 | NMW 28130.16 | Taiwan | M | 351 | 122 | 0 | 3 | 166 | 78 |
| **21** | *P. formosensis* | 0 | NMW 28130.18 | Taiwan | M | 429 | 141 | 0 | 3 | 172 | 78 |
| **22** | *P. formosensis* | 0 | NMW 28130.20 | Kaoshiung, Taiwan | F | 356 | 112 | 0 | 3 | 171 | 77 |
| **23** | *P. formosensis* | 0 | NMW 28130.3 | Alishan, Chiayi, Taiwan | M | 282 | 93 | 0 | 3 | 171 | 83 |
| **24** | *P. formosensis* | 0 | NMW 28130.7 | Alishan, Chiayi, Taiwan | M | 365 | 120 | 0 | 3 | 170 | 77 |
| **25** | *P. formosensis* | 0 | NMW 28130.8 | Alishan, Chiayi, Taiwan | F | 360 | 111 | 0 | 3 | 175 | 77 |
| **26** | *P. formosensis* | 0 | NMW 28130.9 | Alishan, Chiayi, Taiwan | M | 366 | 112 | 0 | 3 | 173 | 73 |
| **27** | *P. formosensis* | Holotype of *Amblycephalus monticola kuangtungensis* | ZMB 27661 | Mt. Luofu, Shiliuguan, Guangdong, China | M | 331 | 105 | 0 | 1 | 181 | 81 |
| **28** | *P. formosensis* | 0 | ZMB 30585 | Alishan, Chiayi, Taiwan | M | 432 | 142 | 0 | 3 | 175 | 74 |
| **29** | *P. formosensis* | 0 | ZMB 65430 | Loshiang, Mt. Yao Shan, Guangxi, China | M | 411 | 155 | 0 | 1 | 184 | 95 |
| **30** | *P. formosensis* | Paratype of *Amblycephalus monticola kuangtungensis* | ZMB 65437 | Longtoushan, Guangzhou, Guangdong, China | M | 190 | 61 | 0 | 1 | ? | 81 |
| **1** | *P. geminatus* | Holotype | CIB 118021 | Jiangchen, Yunnan, China | M | 428 | 138 | 5 | 1 | 180 | 79 |
| **2** | *P. geminatus* | Paratype | CIB 118022 | Jiangchen, Yunnan, China | F | 305 | 81 | 5 | 1 | 186 | 67 |
| **3** | *P. geminatus* | Paratype | CIB 118023 | Jiangchen, Yunnan, China | F | 317 | 82 | 5 | 1 | 181 | 70 |
| **4** | *P. geminatus* | 0 | DL 2019072910 | Jiangchen, Yunnan, China | M | 411 | 110 | 5 | 1 | 182 | 77 |
| **5** | *P. geminatus* | 0 | DL 20190930001 | Jiangchen, Yunnan, China | F | 362 | 94 | 5 | 1 | 174 | 67 |
| **6** | *P. geminatus* | 0 | DL 20190930002 | Jiangchen, Yunnan, China | M | 348 | 103 | 5 | 1 | 170 | 75 |
| **7** | *P. geminatus* | Paratype | MNHN 171S | Huaphanh, Laos | M | 290 | 102 | 3 | 1 | 175 | 91 |
| **8** | *P. geminatus* | Paratype | QSMI 1013 | Tak, Thailand | F | 225 | 72 | 0 | 1 | 188 | 87 |
| **1** | *P. hamptoni* | 0 | CAS 221489 | Naung Mon, Putao, Kachin, Myanmar | M | 469 | 134 | 9 | 1 | 194 | 91 |
| **2** | *P. hamptoni* | 0 | MNHN-RA 1935.87 | Sa Pa, Lao Cai, Vietnam | M | 483 | ? | 5 | 3 | 185 | 92 |
| **3** | *P. hamptoni* | 0 | MNHN-RA 1935.88 | Sa Pa, Lao Cai, Vietnam | M | 446 | 155 | 7 | 3 | 193 | 99 |
| **4** | *P. hamptoni* | Holotype | NHMUK 430223 | Mandalay, Myanmar | M | 403 | 142 | 5 | 3 | 195 | 96 |
| **5** | *P. hamptoni* | 0 | RMNH 6512 | Sa Pa, Lao Cai, Vietnam | M | 471 | 164 | 7 | 1 | 194 | 96 |
| **1** | *P. kaduri* | 0 | CAS 224415 | Hkakabo Razi NP, Khachin, Myanmar | F | 377 | 100 | 5 | 1 | 182 | 71 |
| **1** | *P. komaii* | 0 | NMW 28130.1 | Alishan, Chiayi, Taiwan | F | 355 | 97 | 7 | 0 | 168 | 65 |
| **2** | *P. komaii* | 0 | NMW 28130.10 | Alishan, Chiayi, Taiwan | F | 313 | 92 | 7 | 3 | 176 | 72 |
| **3** | *P. komaii* | 0 | NMW 28130.13 | Taiwan | F | 405 | 113 | 7 | 3 | 173 | 68 |
| **4** | *P. komaii* | 0 | NMW 28130.2 | Alishan, Chiayi, Taiwan | M | 351 | 117 | 7 | 3 | 172 | 79 |
| **5** | *P. komaii* | 0 | NMW 28130.21 | Kaoshiung, Taiwan | F | 415 | 123 | 5 | 3 | 167 | 70 |
| **6** | *P. komaii* | 0 | NMW 28130.22 | Kaoshiung, Taiwan | F | 330 | 89 | 5 | 3 | 169 | 70 |
| **7** | *P. komaii* | 0 | NMW 28130.23 | Kaoshiung, Taiwan | M | 361 | 112 | 7 | 3 | 167 | 77 |
| **8** | *P. komaii* | 0 | NMW 28130.24 | Kaoshiung, Taiwan | M | 293 | 90 | 7 | 3 | 171 | 73 |
| **9** | *P. komaii* | 0 | NMW 28130.5 | Alishan, Chiayi, Taiwan | M | 407 | 118 | 9 | 3 | 177 | 70 |
| **1** | *P. macularius* | 0 | BMNH 1947.1.1.14 | Lam Dong, Vietnam | F | 403 | 71 | 5 | 0 | 152 | 39 |
| **2** | *P. macularius* | 0 | CAS 206620 | Bajo, Bago, Myanmar | M | 355 | 73 | 9 | 0 | 166 | 49 |
| **3** | *P. macularius* | 0 | CAS 247899 | Tanintharyi, Myanmar | F | 331 | 58 | ? | 0 | 173 | 44 |
| **4** | *P. macularius* | 0 | CIB 10155 (725035) | Jianfengling, Hainan, China | M | 322 | 80 | 9 | 0 | 151 | 51 |
| **5** | *P. macularius* | 0 | DL 2019.07.29012 | Jiangcheng, Yunnan, China | F | 392 | 72 | 7 | 0 | 156 | 43 |
| **6** | *P. macularius* | 0 | DTU 479 | Ba Vi, Ha Noi, Vietnam | M | 363 | 79 | 7 | 0 | 154 | 45 |
| **7** | *P. macularius* | 0 | FMNH 135331 | Dansai, Loei, Thailand | M | 209 | 43 | 9 | 0 | 161 | 53 |
| **8** | *P. macularius* | 0 | FMNH 175332 | Ngan Son, Bac Kan, Vietnam | F | 270 | 49 | 5 | 0 | 160 | 43 |
| **9** | *P. macularius* | Syntype of *Amblycephalus tamdaoensis* | MNHN 1938.148 | Tam Dao, Vinh Phuc, Vietnam | F | 388 | 79 | 5 | 0 | 156 | 44 |
| **10** | *P. macularius* | Syntype of *Amblycephalus tamdaoensis* | MNHN 1938.89 | Tam Dao, Vinh Phuc, Vietnam | M | 281 | 57 | 7 | 0 | 152 | 53 |
| **11** | *P. macularius* | Holotype | NHMUK 1912159 | Martaban, Tanintharyi, Myanmar | M | 333 | 70 | ? | 0 | 161 | 50 |
| **12** | *P. macularius* | 0 | NMW 39964.1 | Tam Dao, Vinh Phuc, Vietnam | F | 432 | 85 | 7 | 0 | 154 | 41 |
| **13** | *P. macularius* | 0 | ZFMK 82925 | Nghe An, Vietnam | M | 280 | 67 | 11 | 0 | ? | 49 |
| **14** | *P. macularius* | 0 | ZFMK 86446 | Phong Nha-Ke Bang, Quang Binh, Vietnam | F | 370 | 76 | 9 | 0 | 156 | 45 |
| **15** | *P. macularius* | 0 | ZMMU R-16629 | Ban Mauk, Sagaing, Myanmar | F | 304 | 64 | 7 | 0 | 159 | 44 |
| **1** | *P. margaritophorus* | 0 | CAS 14949 | Hainan, China | F | 215 | 40 | 0 | 0 | 160 | 44 |
| **2** | *P. margaritophorus* | 0 | CIB 10157 (665081) | Diaoluo Shan, Hainan, China | F | 327 | 65 | 0 | 0 | 155 | 43 |
| **3** | *P. margaritophorus* | 0 | CIB 10158 (665080) | Diaoluo Shan, Hainan, China | F | 317 | 60 | 0 | 0 | 157 | 40 |
| **4** | *P. margaritophorus* | 0 | CIB 10160 (705015) | Yuling, Hainan, China | M | 253 | 71 | 0 | 0 | 145 | 49 |
| **5** | *P. margaritophorus* | 0 | CIB 10162 (64III5159) | Wuzhi Shan, Hainan, China | F | 278 | 49 | 0 | 0 | 156 | 41 |
| **6** | *P. margaritophorus* | 0 | CIB 83792 (665082) | Diaoluo Shan, Hainan, China | F | 287 | 57 | 0 | 0 | 150 | 44 |
| **7** | *P. margaritophorus* | 0 | DTU 475 | Pu Mat, Nghe An, Vietnam | F | 300 | 50 | 0 | 0 | 149 | 41 |
| **8** | *P. margaritophorus* | 0 | DTU 476 | Cuc Phuong, Ninh Binh, Vietnam | F | 262 | 67 | 0 | 0 | 133 | 47 |
| **9** | *P. margaritophorus* | 0 | DTU 477 | Cuc Phuong, Ninh Binh, Vietnam | F | 300 | 49 | 0 | 0 | 140 | 40 |
| **10** | *P. margaritophorus* | 0 | DTU 478 | Cuc Phuong, Ninh Binh, Vietnam | F | 249 | 65 | 0 | 0 | 133 | 44 |
| **11** | *P. margaritophorus* | 0 | FMNH 178389 | Pattani, Thailand | F | 241 | 45 | 0 | 0 | 144 | 39 |
| **12** | *P. margaritophorus* | 0 | FMNH 178390 | Chiang Mai, Thailand | F | 265 | 48 | 0 | 0 | 152 | 38 |
| **13** | *P. margaritophorus* | 0 | FMNH 180219 | Nakhon Ratchasima, Thailand | F | 268 | 48 | 0 | 0 | 143 | 36 |
| **14** | *P. margaritophorus* | 0 | FMNH 180220 | Nakhon Ratchasima, Thailand | F | 330 | 59 | 0 | 0 | 138 | 36 |
| **15** | *P. margaritophorus* | 0 | FMNH 233357 | Pahang, Malaysia | F | 235 | 47 | 0 | 0 | 148 | 42 |
| **16** | *P. margaritophorus* | 0 | FMNH 252128 | An Khe, Gia Lai, Vietnam | F | 202 | 35 | 0 | 0 | 149 | 39 |
| **17** | *P. margaritophorus* | 0 | FMNH 256973 | Hong Kong, China | M | 274 | 77 | 0 | 0 | 138 | 49 |
| **18** | *P. margaritophorus* | 0 | FMNH 263022 | Siem Pang, Stung Treng, Cambodia | M | 175 | 42 | 0 | 0 | 145 | 50 |
| **19** | *P. margaritophorus* | 0 | FMNH 263791 | Prachin Buri, Nadi Bu Phram | F | 282 | 48 | 0 | 0 | 145 | 35 |
| **20** | *P. margaritophorus* | 0 | FMNH 267738 | Areng ChumNoab, Koh Kong, Cambodia | F | 274 | 52 | 0 | 0 | 155 | 42 |
| **21** | *P. margaritophorus* | 0 | FMNH 6661 | Hainan, China | F | 258 | 48 | 0 | 0 | 149 | 42 |
| **22** | *P. margaritophorus* | 0 | FMNH 66621 | Hainan, China | F | 245 | 43 | 0 | 0 | 145 | 40 |
| **23** | *P. margaritophorus* | 0 | FMNH 71137 | Hong Kong, China | F | 243 | 52 | 0 | 0 | 143 | 42 |
| **24** | *P. margaritophorus* | 0 | FMNH 71704 | Da Lat, Lam Dong, Vietnam | M | 249 | 65 | 0 | 0 | 135 | 47 |
| **25** | *P. margaritophorus* | 0 | FMNH 71705 | Da Lat, Lam Dong, Vietnam | M | 252 | 63 | 0 | 0 | 137 | 45 |
| **26** | *P. margaritophorus* | Holotype | MNHN 599 | Thailand | M | 241 | 66 | 0 | 0 | 139 | 51 |
| **27** | *P. margaritophorus* | 0 | NMW 28128.10 | Phuoc Son, Quang Nam, Vietnam | M | 267 | 75 | 0 | 0 | 146 | 51 |
| **28** | *P. margaritophorus* | 0 | NMW 28128.12 | Phuoc Son, Quang Nam, Vietnam | M | 172 | 41 | 0 | 0 | 143 | 54 |
| **29** | *P. margaritophorus* | 0 | NMW 28128.2 | Vietnam | M | 192 | 48 | 0 | 0 | 143 | 52 |
| **30** | *P. margaritophorus* | 0 | NMW 28128.3 | Vietnam | F | 274 | ? | 0 | 0 | 145 | ? |
| **31** | *P. margaritophorus* | 0 | NMW 28128.4 | Cambodia | F | 313 | 58 | 0 | 0 | 145 | 39 |
| **32** | *P. margaritophorus* | 0 | NMW 28128.5 | Vietnam | M | 252 | 70 | 0 | 0 | 138 | 49 |
| **33** | *P. margaritophorus* | 0 | NMW 28128.6 | Phuoc Son, Quang Nam, Vietnam | F | 250 | 46 | 0 | 0 | 152 | 41 |
| **34** | *P. margaritophorus* | 0 | NMW 28128.8 | Phuoc Son, Quang Nam, Vietnam | M | 223 | 63 | 0 | 0 | 134 | 48 |
| **35** | *P. margaritophorus* | 0 | NMW 28128.9 | Phuoc Son, Quang Nam, Vietnam | M | 182 | 50 | 0 | 0 | 140 | 51 |
| **36** | *P. margaritophorus* | 0 | NMW 28129.3 | Vietnam | F | 270 | 53 | 0 | 0 | 148 | 40 |
| **37** | *P. margaritophorus* | 0 | NMW 39964.1 | Nahe Ibok, Trengganu, Malaysia | F | 265 | 55 | 0 | 0 | 152 | 40 |
| **38** | *P. margaritophorus* | Holotype of *Pareas moellendorffi* | SMF 20790 | Lo Fou Shan, Guangzhou, China | F | 244 | 43 | 0 | 0 | 149 | 37 |
| **39** | *P. margaritophorus* | 0 | SMF 20791 | Hong Kong, China | F | 286 | 51 | 0 | 0 | 149 | 37 |
| **40** | *P. margaritophorus* | 0 | SMF 20792 | Hong Kong, China | M | 222 | 58 | 0 | 0 | 136 | 46 |
| **41** | *P. margaritophorus* | 0 | ZFMK 70584 | Kuala Lumpur, Malaysia | F | 210 | 46 | 0 | 0 | 148 | 43 |
| **42** | *P. margaritophorus* | 0 | ZFMK 76107 | Mesa, Chiang Mai, Thailand | M | 232 | 65 | 0 | 0 | 138 | 48 |
| **43** | *P. margaritophorus* | 0 | ZFMK 80664 | Phong Nha-Ke Bang, Quang Binh, Vietnam | M | 197 | 51 | 0 | 0 | 141 | 48 |
| **44** | *P. margaritophorus* | 0 | ZFMK 81479 | Ke Go, Ha Tinh, Vietnam | F | 337 | 57 | 0 | 0 | 153 | 38 |
| **45** | *P. margaritophorus* | 0 | ZFMK 82924 | Nghe An, Vietnam | M | 233 | 52 | 0 | 0 | 139 | 46 |
| **46** | *P. margaritophorus* | 0 | ZFMK 90378 | Phnom Kulen, Siem Riep, Cambodia | F | 276 | 46 | 0 | 0 | 149 | 35 |
| **47** | *P. margaritophorus* | 0 | ZFMK 92636 | Phnom Kulen, Siam Reap, Cambodia | M | 262 | 76 | 0 | 0 | 136 | 48 |
| **48** | *P. margaritophorus* | 0 | ZFMK 92637 | Phnom Kulen, Siam Reap, Cambodia | M | 244 | 70 | 0 | 0 | 141 | 49 |
| **49** | *P. margaritophorus* | 0 | ZFMK 95197 | Bai Tu Long, Quang Ninh, Vietnam | F | 315 | 56 | 0 | 0 | 147 | 38 |
| **50** | *P. margaritophorus* | 0 | ZMB 50680 | Perak, Malaysia | M | 230 | 63 | 0 | 0 | 138 | 49 |
| **51** | *P. margaritophorus* | 0 | ZSM 2271.0 | Vietnam | M | 247 | 61 | 0 | 0 | 145 | 47 |
| **1** | *P. modestus* | 0 | MZMU 1193 | Mizoram, India | F | 357 | 53 | 5 | 0 | 157 | 35 |
| **2** | *P. modestus* | 0 | MZMU 1293 | Mizoram, India | M | 226 | 52 | 5 | 0 | 156 | 46 |
| **3** | *P. modestus* | 0 | MZMU 1487 | Mizoram, India | M | 267 | 53 | 5 | 0 | 155 | 45 |
| **4** | *P. modestus* | 0 | MZMU 1537 | Mizoram, India | M | 310 | 75 | 5 | 0 | 151 | 46 |
| **5** | *P. modestus* | 0 | MZMU 1604 | Mizoram, India | F | 304 | 57 | 5 | 0 | 156 | 37 |
| **6** | *P. modestus* | 0 | MZMU 1665 | Mizoram, India | F | 310 | 54 | 5 | 0 | 159 | 38 |
| **7** | *P. modestus* | 0 | MZMU 274 | Mizoram, India | M | 424 | 71 | 3 | 0 | 157 | 42 |
| **8** | *P. modestus* | 0 | MZMU 275 | Mizoram, India | F | 406 | 56 | 5 | 0 | 153 | 38 |
| **1** | *P. monticola* | 0 | BMNH 60.3.19.1312 | Khasi Hills, Meghalaya, India | M | 472 | 138 | 0 | 3 | 191 | 86 |
| **2** | *P. monticola* | 0 | CIB 10163 | Xizang, China | F | 530 | 131 | ? | 0 | 189 | 72 |
| **3** | *P. monticola* | 0 | MZMU 1335 | Mizoram, India | F | 403 | 113 | 0 | 3 | 199 | 83 |
| **4** | *P. monticola* | 0 | MZMU 1485 | Mizoram, India | F | 531 | 143 | 0 | 3 | 195 | 76 |
| **5** | *P. monticola* | 0 | MZMU 1486 | Mizoram, India | F | 487 | 136 | 0 | 3 | 197 | 84 |
| **6** | *P. monticola* | 0 | MZMU 851 | Mizoram, India | F | 508 | ? | 0 | 3 | 198 | ? |
| **7** | *P. monticola* | Holotype | NHMUK 1912235 | Naga Hillas, Assam, India | M | 477 | 145 | 0 | 1 | 192 | 82 |
| **8** | *P. monticola* | 0 | NHMUK 1912238 | Darjiling, west Bengal, India | M | 425 | 126 | 0 | 1 | 187 | 81 |
| **9** | *P. monticola* | 0 | NHMUK 1912240 | Khasi Hills, Meghalaya, India | F | 555 | 151 | 0 | 3 | 190 | 75 |
| **10** | *P. monticola* | 0 | NHMUK 1912242a | Darjiling, west Bengal, India | F | 418 | 110 | 0 | 1 | 188 | 76 |
| **11** | *P. monticola* | 0 | NHMUK 1912242b | Darjiling, west Bengal, India | M | 362 | 110 | 0 | 1 | 185 | 81 |
| **12** | *P. monticola* | 0 | NHMUK 1912242c | Darjiling, west Bengal, India | F | 401 | 99 | 0 | 1 | 183 | 69 |
| **13** | *P. monticola* | 0 | NHMUK 1912242d | Darjiling, west Bengal, India | F | 379 | 97 | 0 | 1 | 186 | 72 |
| **14** | *P. monticola* | 0 | NHMUK 1912244 | Jaipur, Rajasthan, India (erroneous) | M | 428 | 134 | 0 | 1 | 196 | 86 |
| **15** | *P. monticola* | 0 | NHMUK 1912245a | Abor Hills, Arunachal Pradesh, India | F | 493 | 141 | 0 | 1 | 189 | 78 |
| **16** | *P. monticola* | 0 | NHMUK 1912245b | Abor Hills, Arunachal Pradesh, India | M | 415 | 128 | 0 | 3 | 182 | 82 |
| **17** | *P. monticola* | 0 | NHMUK 1912246 | Mishmi Hills, Arunachal Pradesh, India | F | 496 | 123 | 0 | 1 | 187 | 72 |
| **18** | *P. monticola* | 0 | NMW 28127 | Darjiling, west Bengal, India | F | 422 | 99 | ? | 0 | 184 | 69 |
| **19** | *P. monticola* | 0 | ZMH R05510 | Darjiling, west Bengal, India | F | 431 | 125 | 0 | 1 | 196 | 82 |
| **20** | *P. monticola* | 0 | ZMMU R-16630 | Indawgyi, Kachin, Myanmar | F | 482 | 123 | 0 | 1 | 178 | 69 |
| **21** | *P. monticola* | 0 | ZMMU R-16631 | Ban Mauk, Sagaing, Myanmar | M | 386 | 115 | 0 | 1 | 187 | 83 |
| **22** | *P. monticola* | 0 | ZMMU R-166312 | Ban Mauk, Sagaing, Myanmar | M | 319 | 101 | 0 | 1 | 195 | 90 |
| **23** | *P. monticola* | 0 | ZMMU R-16633 | Ban Mauk, Sagaing, Myanmar | J | 343 | 98 | 0 | 1 | 178 | 77 |
| **24** | *P. monticola* | 0 | ZMMU R-16635 | Ban Mauk, Sagaing, Myanmar | J | 184 | 53 | 0 | 1 | 185 | 81 |
| **1** | *P. niger* | 0 | DL 2019.05.29 | Kunming, Yunnan, China | M | 348 | 107 | 9 | 1 | 164 | 68 |
| **2** | *P. niger* | 0 | MH 2015.08S001 | Honghe, Yunnan, China | F | 328 | 79 | 7 | 3 | 164 | 54 |
| **1** | *P. stanleyi* | 0 | CIB 10165 | Fujian, China | M | 423 | 87 | 9 | 3 | 156 | 44 |
| **2** | *P. stanleyi* | 0 | FMNH 24992 | Fujian, China | M | 188 | 45 | 9 | 0 | 152 | 57 |
| **3** | *P. stanleyi* | 0 | FMNH 24991 | Fujian, China | F | 156 | 31 | 7 | 3 | 157 | 49 |
| **4** | *P. stanleyi* | 0 | FMNH 24990 | Fujian, China | F | 176 | 34 | 7 | 1 | 159 | 47 |
| **1** | *P. victorianus* | Holotype | CAS 235254 | Mindat, Chin, Myanmar | M | 385 | 102 | 7 | 1 | 164 | 58 |
| **1** | *P. xuelinensis* | 0 | AUP 01573 | Doi Inthanon, Chiang Mai, Thailand | M | 362 | 122 | 7 | 1 | 190 | 90 |
| **2** | *P. xuelinensis* | 0 | AUP 00175 | Doi Inthanon, Chiang Mai, Thailand | M | 346 | 79 | 6 | 1 | 188 | 68 |
| **3** | *P. xuelinensis* | 0 | AUP 00176 | Doi Inthanon, Chiang Mai, Thailand | M | 423 | 155 | 7 | 1 | 197 | 85 |
| **1** | *P.* cf. *yunnanensis* | 0 | ZMB 27660 | Dali, Yunnan, China | F | 387 | ? | 7 | 1 | 172 | 65 |
| **2** | *P.* cf. *yunnanensis* | Holotype | ZMB 65431 | Dali, Yunnan, China | F | 405 | ? | 5 | 1 | 175 | 64 |
| **3** | *P.* cf. *yunnanensis* | 0 | MH no label | Dali, Yunnan, China | F | 345 | 76 | 5 | 3 | 171 | 57 |

**Supplementary Table S15. (Continued).**

| # | ***Species*** | **Voucher number** | **SL** | **IL** | **At** | **Pt** | **Lor** | **Lor-E** | **SL-E** | **PrO** | **SoO** | **PoO** |
| --- | --- | --- | --- | --- | --- | --- | --- | --- | --- | --- | --- | --- |
| **1** | *P. andersonii* | CAS 233330 | 7/7 | 7/7 | 2/2 | 3/3 | 1/1 | 0/0 | 0/0 | 1/1 | Full | Full |
| **2** | *P. andersonii* | CAS 235218 | 7/7 | 8/7 | ? | ? | 1/1 | 0/0 | 0/0 | 1/1 | Full | Full |
| **3** | *P. andersonii* | CAS 235359 | 6/7 | 8/8 | 2/1 | 3/2 | 1/1 | 0/0 | 0/0 | 1/1 | Full | Full |
| **4** | *P. andersonii* | CAS 241270 | 7/7 | 7/8 | 2/2 | 3/3 | 1/1 | 0/0 | 0/0 | 1/1 | Full | Full |
| **5** | *P. andersonii* | CAS 245296 | 7/7 | 8/8 | 2/2 | 3/2 | 1/1 | 0/0 | 0/0 | 1/1 | Full | Full |
| **6** | *P. andersonii* | CAS 245377 | 7/7 | 8/8 | 2/2 | 3/3 | 1/1 | 0/0 | 0/0 | 1/1 | Full | Full |
| **7** | *P. andersonii* | MZMU 916 | 7/7 | 7/7 | 2/2 | 3/3 | 1/1 | 0/0 | 0/0 | 1/1 | Full | Full |
| **8** | *P. andersonii* | NHMUK 19121115 | 7/7 | 8/8 | 2/2 | 3/3 | 1/1 | 0/0 | 0/0 | 1/1 | Full | Full |
| **9** | *P. andersonii* | NHMUK 1912117b | 7/7 | 7/7 | 2/2 | 3/3 | 1/1 | 0/0 | 0/0 | 1/1 | Full | Full |
| **10** | *P. andersonii* | NHMUK 1912117a | 7/7 | 7/7 | 2/2 | 4/3 | 1/1 | 0/0 | 0/0 | 1/1 | Full | Full |
| **11** | *P. andersonii* | NHMUK 1912119 | 7/7 | 7/8 | 2/2 | 3/3 | 1/1 | 0/0 | 0/0 | 1/1 | Full | Full |
| **12** | *P. andersonii* | NHMUK 430232 | 7/7 | 7/7 | 2/2 | 3/3 | 1/1 | 0/0 | 0/0 | 1/1 | Full | Full |
| **13** | *P. andersonii* | NHMUK 430234 | 7/7 | 7/7 | 2/2 | 3/2 | 1/1 | 0/0 | 0/0 | 1/1 | Full | Full |
| **1** | *P. atayal* | FMNH 127998 | 7/7 | 7/7 | 2/2 | 3/3 | 1/1 | 0/0 | 0/0 | 1/1 | 1/1 | 1/1 |
| **2** | *P. atayal* | FMNH 169315 | 7/7 | 7/7 | 2/2 | 3/3 | 1/1 | 0/0 | 0/0 | 1/1 | 1/1 | 1/1 |
| **3** | *P. atayal* | FMNH 169392 | 7/7 | 6/6 | 2/2 | 3/3 | 1/1 | 0/0 | 0/0 | 1/1 | 1/1 | 1/1 |
| **4** | *P. atayal* | FMNH 169395 | 7/7 | 7/7 | 2/2 | 3/3 | 2/1 | 0/0 | 0/0 | 1/1 | 1/1 | 1/1 |
| **5** | *P. atayal* | NMW 28130.17 | 7/7 | 7/6 | 2/2 | 3/3 | 1/1 | 0/0 | 0/0 | 1/1 | Full | Full |
| **6** | *P. atayal* | ZMMU-R14435 | 7/7 | 7/8 | 2/2 | 3/3 | 1/1 | 0/0 | 0/0 | 1/1 | 1/1 | 1/1 |
| **1** | *P. boulengeri* | CIB 10084 | 7/7 | 8/8 | 2/2 | 3/3 | 1/1 | 1/1 | 0/0 | 0/0 | 1/1 | 0/0 |
| **2** | *P. boulengeri* | DL 026 | 7/7 | 7/7 | 1/1 | 2/2 | 1/1 | 1/1 | 0/0 | 0/0 | Full | Full |
| **3** | *P. boulengeri* | DL 027 | 8/8 | 8/8 | 2/2 | 3/3 | 1/1 | 0/0 | 0/0 | 1/1 | 1/1 | 0/0 |
| **4** | *P. boulengeri* | DL 2018.06.29.01 | 8/7 | 7/8 | 2/2 | 3/3 | 1/1 | 1/1 | 0/0 | 0/0 | Full | Full |
| **5** | *P. boulengeri* | DL 2018.06.29.02 | 8/7 | 7/7 | 2/2 | 3/3 | 1/1 | 0/0 | 0/0 | 0/0 | 1/1 | 0/0 |
| **6** | *P. boulengeri* | DL 2018.08.10.01 | 8/7 | 8/8 | 2/2 | 3/3 | 1/1 | 1/1 | 0/0 | 0/0 | Full | Full |
| **7** | *P. boulengeri* | DL 2019.08.16.01 | 8/7 | 8/8 | 2/2 | 3/3 | 1/1 | 1/1 | 0/0 | 0/0 | 1/1 | 1/1 |
| **8** | *P. boulengeri* | DL 2019.09.23.04 | 8/6 | 8/8 | 2/2 | 3/3 | 1/1 | 1/1 | 0/0 | 0/0 | Full | Full |
| **9** | *P. boulengeri* | MNHN 1912.349 | 8/7 | 8/8 | 2/2 | 3/3 | 1/1 | 1/1 | 0/0 | 0/0 | Full | Full |
| **10** | *P. boulengeri* | MNHN 1912.350 | 8/8 | 8/8 | 2/2 | 3/3 | 1/1 | 1/1 | 0/0 | 0/0 | 1/1 | 0/0 |
| **11** | *P. boulengeri* | MNHN 1912.351 | 8/8 | 8/8 | 2/2 | 3/3 | 1/1 | 1/1 | 0/0 | 0/0 | Full | Full |
| **1** | *P. chinensis* | DL 051 | 7/7 | 9/8 | 2/2 | 3/3 | 1/1 | 0/0 | 0/0 | 1/2 | 1/1 | 0/1 |
| **2** | *P. chinensis* | FMNH 170632 | 8/8 | 9/8 | 2/2 | 3/3 | 1/1 | 0/0 | 0/0 | 1/1 | 1/1 | 1/1 |
| **3** | *P. chinensis* | FMNH 232812 | 7/7 | 7/7 | 2/2 | 3/3 | 1/1 | 0/0 | 0/0 | 1/1 | Full | Full |
| **4** | *P. chinensis* | FMNH 232813 | 6/6 | 8/8 | 2/2 | 3/3 | 1/1 | 0/0 | 0/0 | 1/1 | Full | Full |
| **5** | *P. chinensis* | FMNH 232814 | 8/8 | 8/8 | 2/2 | 3/4 | 1/1 | 0/0 | 0/0 | 1/1 | Full | Full |
| **6** | *P. chinensis* | NMW 39540.1 | 7/7 | 8/8 | 2/2 | 3/3 | 1/1 | 0/0 | 0/0 | 1/2 | Full | Full |
| **7** | *P. chinensis* | NMW 39540.2 | 7/7 | 8/8 | 2/2 | 3/3 | 1/1 | 0/0 | 0/0 | 1/1 | 1/1 | 1/1 |
| **1** | *P. formosensis* | BMNH 1924.5.22.11 | 8/8 | 8/8 | 2/2 | 3/3 | 1/1 | 0/0 | 0/0 | 1/1 | 1/1 | 1/1 |
| **2** | *P. formosensis* | CIB 10145 | 7/7 | 7/7 | 2/2 | 3/3 | 2/2 | 0/0 | 0/0 | 1/1 | Full | Full |
| **3** | *P. formosensis* | CIB 10147 | 7/7 | 7/7 | 3/2 | 2/2 | 2/2 | 0/0 | 0/0 | 1/1 | 1/1 | 1/1 |
| **4** | *P. formosensis* | CIB GX201304417 | 7/7 | 7/7 | 2/2 | 3/3 | 1/1 | 0/0 | 0/0 | 1/1 | Full | Full |
| **5** | *P. formosensis* | DTU 488 | 7/7 | 7/7 | 2/2 | 2/2 | 1/1 | 0/0 | 0/0 | 1/1 | 1/1 | 1/1 |
| **6** | *P. formosensis* | DTU 489 | 7/7 | 7/7 | 1/1 | 2/2 | 1/1 | 0/0 | 0/0 | 1/1 | Full | Full |
| **7** | *P. formosensis* | FMNH 24988 | 7/7 | 7/7 | 2/2 | 3/3 | 2/2 | 0/0 | 0/0 | 1/1 | 1/1 | 1/1 |
| **8** | *P. formosensis* | FMNH 24989 | 7/7 | 7/7 | 2/2 | 3/3 | 1/2 | 0/0 | 0/0 | 1/1 | 1/1 | 1/1 |
| **9** | *P. formosensis* | FMNH 255567 | 7/7 | 8/8 | 2/2 | 3/3 | 1/1 | 0/0 | 0/0 | 1/1 | 1/1 | 1/1 |
| **10** | *P. formosensis* | MNHN 1908.206 | 7/7 | 8/8 | 2/2 | 2/2 | 1/1 | 0/0 | 0/0 | 1/1 | 2/2 | 2/2 |
| **11** | *P. formosensis* | NMW 39665.1 | 7/7 | 7/7 | 2/2 | 3/3 | 1/1 | 0/0 | 0/0 | 1/1 | 1/1 | 1/1 |
| **12** | *P. formosensis* | NHMUK 1912148 | 7/7 | 7/7 | 2/2 | 3/3 | 1/1 | 0/0 | 0/0 | 1/1 | Full | Full |
| **13** | *P. formosensis* | NHMUK 1912152 | 7/7 | 7/7 | 2/2 | 3/3 | 1/1 | 0/0 | 0/0 | 1/1 | 1/Full | 1/Full |
| **14** | *P. formosensis* | NHMUK 1912153 | 8/7 | 7/7 | 2/2 | 3/3 | 1/1 | 0/0 | 0/0 | 1/1 | Full | Full |
| **15** | *P. formosensis* | NHMUK 1912160a | 7/7 | 6/6 | 2/2 | 3/3 | 1/1 | 0/0 | 0/0 | 1/1 | Full/1 | Full/1 |
| **16** | *P. formosensis* | NHMUK 1912160b | 6/7 | 7/6 | 2/2 | 3/3 | 1/1 | 0/0 | 0/0 | 1/1 | Full | Full |
| **17** | *P. formosensis* | NHMUK 1912160c | 7/7 | 7/7 | 2/2 | 3/3 | 1/1 | 0/0 | 0/0 | 1/1 | 1/1 | 1/1 |
| **18** | *P. formosensis* | NMW 28130.12 | 6/7 | 6/6 | 2/2 | 3/3 | 1/1 | 0/0 | 0/0 | 1/1 | Full | Full |
| **19** | *P. formosensis* | NMW 28130.14 | 7/7 | 7/8 | 2/3 | 3/3 | 1/1 | 0/0 | 0/0 | 1/1 | 1/Full | 1/Full |
| **20** | *P. formosensis* | NMW 28130.16 | 7/7 | 8/8 | 3/3 | 3/3 | 2/3 | 0/0 | 0/0 | 1/1 | Full | Full |
| **21** | *P. formosensis* | NMW 28130.18 | 7/7 | 6/7 | 2/2 | 3/3 | 1/1 | 0/0 | 0/0 | 1/1 | Full | Full |
| **22** | *P. formosensis* | NMW 28130.20 | 7/7 | 6/6 | 2/2 | 4/3 | 1/1 | 0/0 | 0/0 | 1/1 | Full | Full |
| **23** | *P. formosensis* | NMW 28130.3 | 7/7 | 6/7 | 2/2 | 3/3 | 1/1 | 0/0 | 0/0 | 1/1 | 1/Full | 1/Full |
| **24** | *P. formosensis* | NMW 28130.7 | 7/7 | 7/7 | 2/2 | 4/3 | 1/1 | 0/0 | 0/0 | 1/1 | Full | Full |
| **25** | *P. formosensis* | NMW 28130.8 | 7/7 | 6/6 | 2/2 | 3/3 | 1/1 | 0/0 | 0/0 | 1/1 | Full | Full |
| **26** | *P. formosensis* | NMW 28130.9 | 8/7 | 7/7 | 2/2 | 3/3 | 1/1 | 0/0 | 0/0 | 1/1 | Full | Full |
| **27** | *P. formosensis* | ZMB 27661 | 7/7 | 7/7 | 2/2 | 3/3 | 1/1 | 0/0 | 0/0 | 2/2 | 1/1 | 1/1 |
| **28** | *P. formosensis* | ZMB 30585 | 7/7 | 6/7 | 2/2 | 3/3 | 1/1 | 0/0 | 0/0 | 0/1 | Full | Full |
| **29** | *P. formosensis* | ZMB 65430 | 7/8 | 7/8 | 2/2 | 3/3 | 1/1 | 0/0 | 0/0 | 1/1 | 1/1 | 1/1 |
| **30** | *P. formosensis* | ZMB 65437 | 7/7 | ? | 2/2 | 3/3 | 1/1 | 0/0 | 0/0 | 1/1 | 1/1 | 1/1 |
| **1** | *P. geminatus* | CIB 118021 | 6/7 | 8/8 | 1/1 | 2/2 | 1/1 | 0/0 | 0/0 | 1/1 | Full | Full |
| **2** | *P. geminatus* | CIB 118022 | 7/7 | 8/8 | 1/1 | 2/2 | 1/1 | 0/0 | 0/0 | 1/1 | Full | Full |
| **3** | *P. geminatus* | CIB 118023 | 7/7 | 8/8 | 2/2 | 2/2 | 1/1 | 0/0 | 0/0 | 1/1 | Full | Full |
| **4** | *P. geminatus* | DL 2019072910 | 7/7 | 8/8 | 2/2 | 2/2 | 1/1 | 0/0 | 0/0 | 1/1 | Full | Full |
| **5** | *P. geminatus* | DL 20190930001 | 7/7 | 8/8 | 2/2 | 2/2 | 1/1 | 0/0 | 0/0 | 1/1 | Full | Full |
| **6** | *P. geminatus* | DL 20190930002 | 7/7 | 8/8 | 2/2 | 2/2 | 1/1 | 0/0 | 0/0 | 1/1 | Full | Full |
| **7** | *P. geminatus* | MNHN 171S | 8/8 | 8/8 | 2/2 | 3/3 | 1/1 | 0/0 | 0/0 | 1/1 | Full | Full |
| **8** | *P. geminatus* | QSMI 1013 | 7/7 | 8/8 | 2/2 | 2/2 | 1/1 | 0/0 | 0/0 | 1/1 | Full | Full |
| **1** | *P. hamptoni* | CAS 221489 | 7/7 | 8/7 | 2/2 | 3/3 | 1/1 | 1/1 | 0/0 | 1/0 | 1/1 | 1/1 |
| **2** | *P. hamptoni* | MNHN-RA 1935.87 | 7/7 | 7/7 | 2/2 | 3/3 | 1/1 | 0/0 | 0/0 | 1/1 | 1/1 | 1/1 |
| **3** | *P. hamptoni* | MNHN-RA 1935.88 | 7/7 | 8/8 | 2/2 | 2/3 | 1/1 | 0/0 | 0/0 | 1/1 | 1/1 | 1/1 |
| **4** | *P. hamptoni* | NHMUK 430223 | 7/8 | 6/7 | 1/1 | 2/2 | 1/1 | 0/0 | 0/0 | 1/1 | Full | Full |
| **5** | *P. hamptoni* | RMNH 6512 | 8/7 | 9/8 | 2/2 | 3/3 | 1/1 | 0/0 | 0/0 | 1/1 | Full | Full |
| **1** | *P. kaduri* | CAS 224415 | 7/7 | 7/7 | 2/2 | 3/3 | 1/1 | 0/0 | 0/0 | 0/0 | 1/1 | 1/1 |
| **1** | *P. komaii* | NMW 28130.1 | 7/7 | 7/7 | 2/2 | 3/3 | 1/1 | 0/0 | 0/0 | 1/1 | Full | Full |
| **2** | *P. komaii* | NMW 28130.10 | 7/7 | 8/7 | 2/2 | 3/3 | 1/1 | 0/0 | 0/0 | 1/1 | Full | Full |
| **3** | *P. komaii* | NMW 28130.13 | 6/7 | 6/7 | 2/2 | 3/3 | 1/1 | 0/0 | 0/0 | 1/1 | Full | Full |
| **4** | *P. komaii* | NMW 28130.2 | 7/7 | 7/7 | 2/2 | 3/4 | 1/1 | 0/0 | 0/0 | 1/1 | Full | Full |
| **5** | *P. komaii* | NMW 28130.21 | 7/7 | 8/7 | 3/3 | 4/3 | 1/1 | 0/0 | 0/0 | 1/1 | Full | Full |
| **6** | *P. komaii* | NMW 28130.22 | 7/7 | 7/7 | 2/2 | 3/3 | 1/1 | 0/0 | 0/0 | 1/1 | Full | Full |
| **7** | *P. komaii* | NMW 28130.23 | 7/7 | 9/7 | 3/3 | 4/4 | 1/1 | 0/0 | 0/0 | 1/1 | 1/1 | 1/1 |
| **8** | *P. komaii* | NMW 28130.24 | 7/7 | 8/8 | 3/2 | 4/3 | 3/2 | 1/0 | 0/0 | 1/2 | 1/1 | 2/1 |
| **9** | *P. komaii* | NMW 28130.5 | 7/7 | 7/7 | 2/2 | 3/3 | 1/1 | 0/0 | 0/0 | 1/1 | Full | Full |
| **1** | *P. macularius* | BMNH 1947.1.1.14 | 7/7 | 8/8 | 2/2 | 3/3 | 1/1 | 0/0 | 0/0 | 1/1 | Full | Full |
| **2** | *P. macularius* | CAS 206620 | 7/7 | 7/7 | 2/2 | 3/3 | 1/1 | 0/0 | 0/0 | 1/1 | Full | Full |
| **3** | *P. macularius* | CAS 247899 | 7/7 | 8/8 | 3/3 | 3/2 | 1/1 | 0/0 | 0/0 | 1/1 | Full | Full |
| **4** | *P. macularius* | CIB 10155 (725035) | 7/7 | 8/7 | 2/2 | 3/3 | 1/1 | 0/0 | 0/0 | 1/1 | Full | Full |
| **5** | *P. macularius* | DL 2019.07.29012 | 7/7 | 6/6 | 2/2 | 3/3 | 1/1 | 0/0 | 0/0 | 1/1 | Full | Full |
| **6** | *P. macularius* | DTU 479 | 7/7 | 8/8 | 2/2 | 3/3 | 1/1 | 0/0 | 0/0 | 1/1 | Full | Full |
| **7** | *P. macularius* | FMNH 135331 | 7/7 | 7/8 | 2/2 | 3/3 | 1/1 | 0/0 | 0/0 | 1/1 | Full | Full |
| **8** | *P. macularius* | FMNH 175332 | 7/7 | 7/7 | 2/2 | 3/3 | 1/1 | 0/0 | 0/0 | 1/1 | Full | Full |
| **9** | *P. macularius* | MNHN 1938.148 | 7/7 | 8/7 | 2/2 | 4/3 | 1/1 | 0/0 | 0/0 | 1/1 | Full | Full |
| **10** | *P. macularius* | MNHN 1938.89 | 7/7 | 8/7 | 2/2 | 3/3 | 1/1 | 0/0 | 0/0 | 1/1 | Full | Full |
| **11** | *P. macularius* | NHMUK 1912159 | 7/7 | 8/8 | 2/2 | 2/3 | 1/1 | 0/0 | 0/0 | 1/1 | Full | Full |
| **12** | *P. macularius* | NMW 39964.1 | 7/7 | 7/7 | 2/2 | 3/3 | 1/1 | 0/0 | 0/0 | 1/1 | Full | Full |
| **13** | *P. macularius* | ZFMK 82925 | 7/7 | 8/7 | 2/2 | 3/3 | 1/1 | 0/0 | 0/0 | 1/1 | Full | Full |
| **14** | *P. macularius* | ZFMK 86446 | 7/7 | 8/7 | 2/2 | 3/3 | 1/1 | 0/0 | 0/0 | 1/1 | Full | Full |
| **15** | *P. macularius* | ZMMU R-16629 | 7/7 | 7/7 | 2/2 | 3/3 | 1/1 | 0/0 | 0/0 | 1/1 | Full | Full |
| **1** | *P. margaritophorus* | CAS 14949 | 7/7 | 8/8 | 2/2 | 2/1 | 1/1 | 0/0 | 0/0 | 1/1 | Full | Full |
| **2** | *P. margaritophorus* | CIB 10157 (665081) | 7/7 | 8/7 | 2/2 | 2/2 | 1/1 | 0/0 | 0/0 | 1/1 | Full | Full |
| **3** | *P. margaritophorus* | CIB 10158 (665080) | 7/7 | 7/7 | 2/2 | 2/2 | 1/1 | 0/0 | 0/0 | 1/1 | Full | Full |
| **4** | *P. margaritophorus* | CIB 10160 (705015) | 6/7 | 7/5 | 2/2 | 2/2 | 1/1 | 0/0 | 0/0 | 1/1 | Full | Full |
| **5** | *P. margaritophorus* | CIB 10162 (64III5159) | 7/7 | 6/8 | 2/2 | 4/4 | 1/1 | 0/0 | 0/0 | 1/1 | Full | Full |
| **6** | *P. margaritophorus* | CIB 83792 (665082) | 7/7 | 8/8 | 2/2 | 2/2 | 1/1 | 0/0 | 0/0 | 1/1 | Full | Full |
| **7** | *P. margaritophorus* | DTU 475 | 7/7 | 7/7 | 2/2 | 3/3 | 1/1 | 0/0 | 0/0 | 1/1 | Full | Full |
| **8** | *P. margaritophorus* | DTU 476 | 7/7 | 7/7 | 2/2 | 3/3 | 1/1 | 0/0 | 0/0 | 1/1 | Full | Full |
| **9** | *P. margaritophorus* | DTU 477 | 7/7 | 7/7 | 2/2 | 3/3 | 1/1 | 0/0 | 0/0 | 1/1 | Full | Full |
| **10** | *P. margaritophorus* | DTU 478 | 7/7 | 7/7 | 2/2 | 3/3 | 1/1 | 0/0 | 0/0 | 1/1 | Full | Full |
| **11** | *P. margaritophorus* | FMNH 178389 | 7/7 | 7/7 | 2/2 | 1/1 | 1/1 | 0/0 | 0/0 | 1/1 | Full | Full |
| **12** | *P. margaritophorus* | FMNH 178390 | 7/7 | 7/8 | 2/2 | 2/2 | 1/1 | 0/0 | 0/0 | 1/1 | Full | Full |
| **13** | *P. margaritophorus* | FMNH 180219 | 8/8 | 8/8 | 2/2 | 1/2 | 1/1 | 0/0 | 0/0 | 1/1 | Full | Full |
| **14** | *P. margaritophorus* | FMNH 180220 | 7/7 | 7/7 | 2/2 | 2/2 | 1/1 | 0/0 | 0/0 | 1/1 | Full | Full |
| **15** | *P. margaritophorus* | FMNH 233357 | 6/7 | 7/7 | 2/2 | 2/1 | 1/1 | 0/0 | 0/0 | 1/1 | Full | Full |
| **16** | *P. margaritophorus* | FMNH 252128 | 7/7 | 8/8 | 2/2 | 2/2 | 1/1 | 0/0 | 0/0 | 1/1 | Full | Full |
| **17** | *P. margaritophorus* | FMNH 256973 | 7/7 | 6/7 | 2/2 | 2/2 | 1/1 | 0/0 | 0/0 | 1/1 | Full | Full |
| **18** | *P. margaritophorus* | FMNH 263022 | 7/7 | 8/7 | 2/2 | 1/3 | 1/1 | 0/0 | 0/0 | 1/1 | Full | Full |
| **19** | *P. margaritophorus* | FMNH 263791 | 7/7 | 8/7 | 2/2 | 3/3 | 1/1 | 0/0 | 0/0 | 1/1 | Full | Full |
| **20** | *P. margaritophorus* | FMNH 267738 | 7/7 | 7/7 | 2/2 | 2/2 | 1/1 | 0/0 | 0/0 | 1/1 | Full | Full |
| **21** | *P. margaritophorus* | FMNH 6661 | 7/7 | 8/7 | 2/2 | 2/2 | 1/1 | 0/0 | 0/0 | 1/1 | Full | Full |
| **22** | *P. margaritophorus* | FMNH 66621 | 7/7 | 7/7 | 2/2 | 2/2 | 1/1 | 0/0 | 0/0 | 1/1 | Full | Full |
| **23** | *P. margaritophorus* | FMNH 71137 | 6/7 | 7/6 | 2/2 | 2/3 | 1/1 | 0/0 | 0/0 | 1/1 | Full | Full |
| **24** | *P. margaritophorus* | FMNH 71704 | 7/7 | 7/8 | 2/2 | 1/1 | 1/1 | 0/0 | 0/0 | 1/1 | Full | Full |
| **25** | *P. margaritophorus* | FMNH 71705 | 7/7 | 7/6 | 2/2 | 2/1 | 1/1 | 0/0 | 0/0 | 1/1 | Full | Full |
| **26** | *P. margaritophorus* | MNHN 599 | 7/7 | 8/9 | 2/2 | 2/2 | 1/1 | 0/0 | 0/0 | 1/1 | Full | Full |
| **27** | *P. margaritophorus* | NMW 28128.10 | 8/9 | 7/7 | 2/2 | 3/3 | 1/1 | 0/0 | 0/0 | 1/1 | Full | Full |
| **28** | *P. margaritophorus* | NMW 28128.12 | 9/8 | 8/7 | 2/2 | 3/3 | 1/1 | 0/0 | 0/0 | 1/1 | Full | Full |
| **29** | *P. margaritophorus* | NMW 28128.2 | 7/7 | 7/7 | 2/2 | 3/3 | 1/1 | 0/0 | 0/0 | 1/1 | Full | Full |
| **30** | *P. margaritophorus* | NMW 28128.3 | 7/7 | 9/8 | 2/2 | 2/3 | 1/1 | 0/0 | 0/0 | 1/1 | Full | Full |
| **31** | *P. margaritophorus* | NMW 28128.4 | 8/8 | 7/8 | 2/2 | 3/2 | 1/1 | 0/0 | 0/0 | 1/1 | Full | Full |
| **32** | *P. margaritophorus* | NMW 28128.5 | 7/7 | 7/8 | 2/3 | 2/2 | 1/1 | 0/0 | 0/0 | 1/1 | Full | Full |
| **33** | *P. margaritophorus* | NMW 28128.6 | 8/8 | 7/7 | 2/2 | 2/2 | 1/1 | 0/0 | 0/0 | 1/1 | Full | Full |
| **34** | *P. margaritophorus* | NMW 28128.8 | 7/7 | 7/7 | 2/3 | 2/2 | 1/1 | 0/0 | 0/0 | 1/1 | Full | Full |
| **35** | *P. margaritophorus* | NMW 28128.9 | 8/8 | 7/7 | 2/3 | 2/2 | 1/1 | 0/0 | 0/0 | 1/1 | Full | Full |
| **36** | *P. margaritophorus* | NMW 28129.3 | 7/7 | 8/7 | 2/2 | 2/2 | 1/1 | 0/0 | 0/0 | 1/1 | Full | Full |
| **37** | *P. margaritophorus* | NMW 39964.1 | 7/7 | 7/7 | 2/1 | 2/2 | 1/1 | 0/0 | 0/0 | 1/1 | Full | Full |
| **38** | *P. margaritophorus* | SMF 20790 | 7/7 | 7/6 | 2/2 | 3/3 | 1/1 | 0/0 | 0/0 | 1/1 | Full | Full |
| **39** | *P. margaritophorus* | SMF 20791 | 7/7 | 7/7 | 2/2 | 2/2 | 1/1 | 0/0 | 0/0 | 1/1 | Full | Full |
| **40** | *P. margaritophorus* | SMF 20792 | 7/7 | 7/7 | 2/2 | 2/2 | 1/1 | 0/0 | 0/0 | 1/1 | Full | Full |
| **41** | *P. margaritophorus* | ZFMK 70584 | 7/7 | 7/7 | 2/2 | 4/3 | 1/1 | 0/0 | 0/0 | 1/1 | Full | Full |
| **42** | *P. margaritophorus* | ZFMK 76107 | 7/7 | 7/7 | 2/2 | 3/3 | 1/1 | 0/0 | 0/0 | 1/1 | Full | Full |
| **43** | *P. margaritophorus* | ZFMK 80664 | 8/7 | 8/8 | 2/2 | 3/3 | 1/1 | 0/0 | 0/0 | 1/1 | Full | Full |
| **44** | *P. margaritophorus* | ZFMK 81479 | 7/7 | 7/7 | 2/2 | 1/1 | 1/1 | 0/0 | 0/0 | 1/1 | Full | Full |
| **45** | *P. margaritophorus* | ZFMK 82924 | 7/7 | 8/7 | 2/2 | 2/2 | 1/1 | 0/0 | 0/0 | 1/1 | Full | Full |
| **46** | *P. margaritophorus* | ZFMK 90378 | 7/7 | 7/7 | 2/2 | 2/2 | 1/1 | 0/0 | 0/0 | 1/1 | Full | Full |
| **47** | *P. margaritophorus* | ZFMK 92636 | 7/7 | 7/7 | 2/2 | 2/1 | 1/1 | 0/0 | 0/0 | 1/1 | Full | Full |
| **48** | *P. margaritophorus* | ZFMK 92637 | 7/7 | 7/7 | 2/2 | 1/1 | 1/1 | 0/0 | 0/0 | 1/1 | Full | Full |
| **49** | *P. margaritophorus* | ZFMK 95197 | 8/7 | 7/7 | 2/2 | 2/1 | 1/1 | 0/0 | 0/0 | 1/1 | Full | Full |
| **50** | *P. margaritophorus* | ZMB 50680 | 7/7 | 7/7 | 2/2 | 3/3 | 1/1 | 0/0 | 0/0 | 1/1 | Full | Full |
| **51** | *P. margaritophorus* | ZSM 2271.0 | 7/8 | 8/8 | 2/2 | 3/2 | 1/1 | 0/0 | 0/0 | 1/1 | Full | Full |
| **1** | *P. modestus* | MZMU 1193 | 7/7 | 7/7 | 2/2 | 2/2 | 1/1 | 0/0 | 0/0 | 1/1 | Full | Full |
| **2** | *P. modestus* | MZMU 1293 | 7/7 | 7/7 | 2/2 | 3/3 | 1/1 | 0/0 | 0/0 | 1/1 | Full | Full |
| **3** | *P. modestus* | MZMU 1487 | 7/7 | 7/7 | 2/2 | 3/3 | 1/1 | 0/0 | 0/0 | 1/1 | Full | Full |
| **4** | *P. modestus* | MZMU 1537 | 7/7 | 7/7 | 2/2 | 3/3 | 1/1 | 0/0 | 0/0 | 1/1 | Full | Full |
| **5** | *P. modestus* | MZMU 1604 | 7/7 | 7/7 | 2/2 | 3/3 | 1/1 | 0/0 | 0/0 | 1/1 | Full | Full |
| **6** | *P. modestus* | MZMU 1665 | 7/7 | 7/7 | 2/2 | 2/2 | 1/1 | 0/0 | 0/0 | 1/1 | Full | Full |
| **7** | *P. modestus* | MZMU 274 | 7/7 | 7/7 | 2/2 | 3/3 | 1/1 | 0/0 | 0/0 | 1/1 | Full | Full |
| **8** | *P. modestus* | MZMU 275 | 7/7 | 7/7 | 2/2 | 3/3 | 1/1 | 0/0 | 0/0 | 1/1 | Full | Full |
| **1** | *P. monticola* | BMNH 60.3.19.1312 | 7/7 | 7/7 | 2/2 | 3/3 | 1/1 | 0/0 | 1/1 | 1/1 | 0/0 | 2/2 |
| **2** | *P. monticola* | CIB 10163 | 7/7 | 9/8 | 2/2 | 3/3 | 1/1 | 0/0 | 1/1 | 1/1 | 0/0 | 3/3 |
| **3** | *P. monticola* | MZMU 1335 | 7/7 | 7/7 | 2/2 | 3/3 | 1/1 | 0/0 | 0/0 | 1/1 | 1/1 | 2/2 |
| **4** | *P. monticola* | MZMU 1485 | 7/7 | 8/9 | 2/2 | 3/3 | 1/1 | 0/0 | 1/1 | 1/1 | 0/0 | 2/2 |
| **5** | *P. monticola* | MZMU 1486 | 7/7 | 7/7 | 2/2 | 3/3 | 1/1 | 0/0 | 1/1 | 1/1 | 0/0 | 2/2 |
| **6** | *P. monticola* | MZMU 851 | 7/7 | 8/7 | 2/2 | 3/3 | 1/1 | 0/0 | 1/1 | 1/1 | 0/0 | 2/2 |
| **7** | *P. monticola* | NHMUK 1912235 | 7/7 | 7/7 | 2/2 | 3/3 | 1/1 | 0/0 | 1/1 | 1/1 | 0/0 | 2/2 |
| **8** | *P. monticola* | NHMUK 1912238 | 7/7 | 8/8 | 2/2 | 3/3 | 1/1 | 0/0 | 1/1 | 1/1 | 0/0 | 2/2 |
| **9** | *P. monticola* | NHMUK 1912240 | 7/6 | 7/7 | 2/2 | 3/3 | 1/1 | 0/0 | 1/1 | 1/1 | 0/0 | 2/2 |
| **10** | *P. monticola* | NHMUK 1912242a | 7/7 | 7/7 | 2/2 | 3/3 | 1/1 | 0/0 | 1/1 | 1/1 | 0/0 | 2/2 |
| **11** | *P. monticola* | NHMUK 1912242b | 7/7 | 7/7 | 2/2 | 3/3 | 1/1 | 0/0 | 1/1 | 1/1 | 0/0 | 2/2 |
| **12** | *P. monticola* | NHMUK 1912242c | 7/7 | 7/7 | 2/2 | 3/3 | 1/1 | 0/0 | 1/1 | 1/1 | 0/0 | 2/2 |
| **13** | *P. monticola* | NHMUK 1912242d | 7/7 | 7/7 | 2/2 | 3/3 | 1/1 | 0/0 | 1/1 | 1/1 | 0/0 | 2/2 |
| **14** | *P. monticola* | NHMUK 1912244 | 8/7 | 8/8 | 2/2 | 3/3 | 1/1 | 0/0 | 0/0 | 1/1 | 0/0 | 2/2 |
| **15** | *P. monticola* | NHMUK 1912245a | 8/7 | 7/7 | 2/2 | 3/4 | 1/1 | 0/0 | 1/1 | 1/1 | 0/0 | 2/2 |
| **16** | *P. monticola* | NHMUK 1912245b | 7/7 | 8/7 | 2/2 | 3/3 | 1/1 | 0/0 | 0/0 | 1/1 | 1/1 | 2/2 |
| **17** | *P. monticola* | NHMUK 1912246 | 7/7 | 7/7 | 2/2 | 3/3 | 1/1 | 0/0 | 0/0 | 1/1 | 0/0 | 2/2 |
| **18** | *P. monticola* | NMW 28127 | 7/7 | 8/8 | 2/2 | 3/3 | 1/1 | 0/0 | 0/0 | 1/1 | 1/1 | 2/2 |
| **19** | *P. monticola* | ZMH R05510 | 7/7 | 7/7 | 2/2 | 3/3 | 1/1 | 0/0 | 0/0 | 1/1 | 1/1 | 2/2 |
| **20** | *P. monticola* | ZMMU R-16630 | 7/7 | 7/7 | 2/2 | 3/3 | 1/1 | 0/0 | 0/0 | 1/1 | 1/1 | 2/2 |
| **21** | *P. monticola* | ZMMU R-16631 | 7/7 | 7/7 | 2/2 | 3/3 | 1/1 | 0/0 | 0/0 | 1/1 | 1/1 | 2/2 |
| **22** | *P. monticola* | ZMMU R-166312 | 7/7 | 8/8 | 2/2 | 3/3 | 1/1 | 0/0 | 0/0 | 1/1 | 1/1 | 2/2 |
| **23** | *P. monticola* | ZMMU R-16633 | 7/7 | 7/7 | 2/2 | 3/3 | 1/1 | 0/0 | 0/0 | 1/1 | 1/1 | 2/2 |
| **24** | *P. monticola* | ZMMU R-16635 | 7/7 | 7/7 | 2/2 | 3/3 | 1/1 | 0/0 | 0/0 | 1/1 | 1/1 | 2/2 |
| **1** | *P. niger* | DL 2019.05.29 | 7/7 | 7/7 | 1/1 | 2/2 | 1/1 | 0/0 | 0/0 | 1/1 | 2/2 | 1/1 |
| **2** | *P. niger* | MH 2015.08S001 | 7/7 | 7/7 | 2/2 | 3/3 | 1/1 | 0/0 | 0/0 | 1/1 | Full | Full |
| **1** | *P. stanleyi* | CIB 10165 | 8/? | 8/9 | 2/2 | 3/4 | 1/1 | 1/1 | 1/1 | 0/0 | 1/1 | 1/1 |
| **2** | *P. stanleyi* | FMNH 24992 | 7/8 | 5/5 | 1/1 | 3/3 | 1/1 | 1/1 | 1/1 | 0/0 | 1/1 | 1/1 |
| **3** | *P. stanleyi* | FMNH 24991 | 7/8 | 7/6 | 1/1 | 3/3 | 1/1 | 1/1 | 1/1 | 1/1 | Full | Full |
| **4** | *P. stanleyi* | FMNH 24990 | 7/7 | 7/7 | 1/1 | 3/3 | 1/1 | 1/1 | 0/0 | 0/0 | Full | Full |
| **1** | *P. victorianus* | CAS 235254 | 7/7 | 7/6 | 2/2 | 2/3 | 1/1 | 0/0 | 0/0 | 0/0 | 1/1 | 1/1 |
| **1** | *P. xuelinensis* | AUP 01573 | 7//7 | 8//8 | 2//2 | 3//3 | 1//1 | 0//0 | 0/0 | 1//1 | Full | Full |
| **2** | *P. xuelinensis* | AUP 00175 | 7//7 | 8//8 | 2//2 | 3//3 | 1//1 | 0//0 | 0/0 | 1//1 | Full | Full |
| **3** | *P. xuelinensis* | AUP 00176 | 7//7 | 8//8 | 2//2 | 3//3 | 1//1 | 0//0 | 0/0 | 1//1 | Full | Full |
| **1** | *P.* cf. *yunnanensis* | ZMB 27660 | ? | ? | 2//2 | 3//2 | 1//1 | 0//0 | 0/0 | 1//1 | Full | Full |
| **2** | *P.* cf. *yunnanensis* | ZMB 65431 | 7//6 | 7//7 | 2//2 | 3//3 | 1//1 | 0//0 | 0/0 | 1//1 | Full | Full |
| **3** | *P.* cf. *yunnanensis* | MH no label | 8//8 | 7//? | 1//1 | 2//2 | 1//1 | 0//0 | 0/0 | 1//1 | Full | Full |
