# Supplementary material for: An integrative taxonomic revision of slug-eating snakes (Squamata: Pareidae: Pareineae) reveals unprecedented diversity in Indochina
Source: PeerJ. 2022 Jan 10;10:e12713. doi: 10.7717/peerj.12713 (PMC8757378; doi:10.7717/peerj.12713)
Supplement: Supplemental Information 17 [file peerj-10-12713-s017.docx]

### Appendix 1. *Museum abbreviations*:

1. AUP: School of Agriculture and Natural Resources, University of Phayao, Phayao, Thailand;
2. BNHS: Bombay Natural History Society, Mumbai, India;
3. CAS: California Academy of Sciences Museum, California, USA;
4. CHS: Song Huang’s private collection, College of Life Sciences, Anhui Normal University, Wuhu, Anhui, China;
5. CIB: Chengdu Institute of Biology, Chengdu, China;
6. DL: Ding Lee‘s private collection, Chengdu, China;
7. DTU: Duy Tan University, Da Nang, Vietnam;
8. FMNH: Field Museum of Natural History, Chicago, USA;
9. GP: Guo Peng‘s private collection, College of Life Science and Food Engineering, Yibin University, Yibin, China;
10. KIZ: Kunming Institute of Zoology, Chinese Academy of Sciences, Kunming, Yunnan, China;
11. MNHN: Muséum National d‘Histoire Naturelle, Paris, France;
12. LSUHC: La Sierra University Herpetological Collection, Riverside, California, USA;
13. MSNG: Museo Civico di Storia Naturale “Giacomo Doria,” Genova, Liguria, Italy;
14. MZB: Museum Zoologicum Bogoriense, Juanda 3, Kebun Raya, Bogor, Java, Indonesia;
15. MZMU: Departmental Museum of Zoology, Mizoram University, Mizoram, India;
16. NHMUK (formerly BMNH): The Natural History Museum, London, UK;
17. NMNS: National Museum of Natural Science, Taichung, Taiwan;
18. NMW: Naturhistorisches Museum Wien, Vienna, Austria;
19. QSMI: Queen Saovabha Memorial Institute, Thai Red Cross Society, Bangkok, Thailand;
20. RMNH: Naturalis-Nationaal Natuurhistorisch Museum [formerly Rijksmuseum van Natuurlijke Historie], Leiden, the Netherlands (includes MHNPB & ZMA);
21. SIEZC: Southern Institute of Ecology, Ho Chi Minh City, Vietnam;
22. SMF: Naturmuseum Senckenberg, Frankfurt am Main, Germany;
23. UNS: University of Science, Ho Chi Minh City, Vietnam;
24. USNM: National Museum of Natural History [formerly United States National Museum], Smithsonian Institution, Washington, District of Columbia, USA;
25. YPX: Field number for tissue samples stored in KIZ;
26. ZFMK: Zoologisches Forschungsmuseum Alexander Koenig, Bonn, Germany;
27. ZMB: Zoologisches Museum für Naturkunde der Humboldt-Universität zu Berlin, Berlin, Germany;
28. ZMH: Zoologisches Institut und Museum, Universität Hamburg, Hamburg, Germany;
29. ZMMU: Zoological Museum of Moscow University, Moscow, Russia;
30. ZSM: Zoologische Staatssammlung, München, Germany.
