# Supplementary material for: An integrative taxonomic revision of slug-eating snakes (Squamata: Pareidae: Pareineae) reveals unprecedented diversity in Indochina
Source: PeerJ. 2022 Jan 10;10:e12713. doi: 10.7717/peerj.12713 (PMC8757378; doi:10.7717/peerj.12713)
Supplement: Supplemental Information 18 — Localities are given as with the specimens. [file peerj-10-12713-s018.docx]

**Appendix II**. **List of *Aplopeltura, Asthenodipsas*, and *Pareas* specimens examined morphologically**

(Localities are given as with the specimens)

***Aplopeltura boa* (2 specimens)**. **Indonesia**: Batang Gadis N.P., Sumatra: ZSM 512.1909; Nias, Sumatra: ZSM 363.1920.

***Asthenodipsas (Asthenodipsas) stuebingi* (1 specimen)**. **Malaysia**: Mt. Kinabalu, Borneo: MZB 65429.

***Asthenodipsas (Asthenodipsas) borneensis* (1 specimen)**. **Indonesia**: Kalimantan, Borneo: ZSM 143.1907

***Asthenodipsas (Asthenodipsas) laevis* (15 specimens)**. **Indonesia**: Banka Isiland: ZMA 16245; Bogo City, Java: SMF 20788–89; Java: ZMB 5231, RMNH 986A–C (Lectotype of *Asthenodipsas laevis*); Padang City, Sumatra: NMW 13382, NMW 21823:2; Bukit Lawang, Sumatra: MZB 2728; Sumatra: SMF 81195, ZSM 126/1947; **Malaysia**: Bintulu, Sarawak, Borneo: FMNH 273617, NMW 28123.5.

***Asthenodipsas (Asthenodipsas) malaccanus* (10 specimens)**. **Indonesia**: Malacca: ZMB 5041 (Holotype of *Asthenodipsas malaccana*); Bengkulu, Sumatra: MZB 3592; west Sumatra: NMW 28126.1–2; Batavia, Java: MNHN 1939.201–204. **Malaysia**: Perak State: SMF 32580. **Thailand**: Thung Song District, Nakhon Si Thammarat Province: ZMB 50673.

***Asthenodipsas (Spondylodipsas) lasgalensis* (5 specimens**). **Malaysia**: Cameron Highlands, Pahang State: ZFMK 53098, ZMB 57111–12, 49675, 55198.

***Asthenodipsas (Spondylodipsas) tropidonotus* (5 specimens)**. **Indonesia**: Padang City, Sumatra: NMW 28126.3–4; Kabu Peraku, Lampung Province, Sumatra: MZB 3725; Mt. Pesogi, Lampung Province, Sumatra: MZB 1816; Sumatra: Reza collection:

***Asthenodipsas (Spondylodipsas) vertebralis* (1 specimen)**. **Malaysia**: Fraser’s Hill, Pahang State: ZMB 52072.

***Pareas (Eberhardtia) andersonii*** **(13 specimens)**. **India**: Vanlaiphai, Lunglei District, Mizoram State: MZMU 916. **Myanmar**: Moenyin Township, Myitkyina District, Kachin State: CAS 241270; Lahe Township, Khandi Distinct, Sagaing Division: CAS 245377, 245296; Mandalay Division: NHMUK 19121115 (formerly BMNH 1901.9.14.11), NHMUK 1912117a,b (formerly BMNH 1904.4.26.13–14; respectively); Mongmit Township, Shan State: NHMUK 430232 (formerly BMNH 1925.12.22.1); Kalaw Township, Taunggyi District, Shan State: NHMUK 430234 (formerly BMNH 1926.3.17.9); Kanpatlat Town, Mindat District, Chin State: CAS 235218; Natnataung N.P., Old Kanpetlet Township, Mindat District, Chin State: CAS 235359; Haka Township, Chin State: CAS 233330; specific locations not available: NHMUK 1912119 (formerly BMNH 1908.6.23.94).

Pareas ***(Eberhardtia)*** atayal (6 **specimens)**. Taiwan: Yangminshan N.P., Taipei City: FMNH 127998, 169315, 169392, 169395; Taoyuan City: ZMMU-R14435 (Paratype of Pareas atayal); specific locations not available: NMW 28130.17.

Pareas ***(Eberhardtia)*** boulengeri (11 specimens). China: Kouy Tcheounow, Guizhou Province: MNHN 1912.0349–51 (syntypes of Amblycephalus boulengeri); Fangxiang Village, Leishan County, Guizhou Province: CIB 10084; Ebian County, Sichuan Province: DL 026–027; Xingou Village, Ya'an City, Sichuan Province: DL 2019.09.23.04; Xianju County, Zhejiang Province: DL 2018.06.29.01–02, 2018.08.10.01; Yangjiang County, Guangdong Province: DL 2019.08.16.01.

Pareas ***(Eberhardtia)*** chinensis (7 specimens). China: Mt. Jiguan, Sichuan Province: DL 051, NMW 39540.1–2; Hongya County, Meishan City, Sichuan Province: FMNH 232812–14; Sichuan Province: FMNH 170632.

Pareas ***(Eberhardtia)*** formosensis (30 specimens). China: Mt. Wuzhi, Hainan Province: BMNH 1924.5.22.11 (holotype of Amblycephalus carinatus hainanus), NHMUK 1912152 (formerly BMNH 1937.2.1.18) [paratype of Amblycephalus carinatus hainanus]; Hongkong City: NHMUK 1912160a,b,c (formerly BMNH 1983.230–232; respectively); Mt. Wuyishan, Fujian Province: CIB 10145, 10147 & FMNH 24988–89; Mt. Luofu, Shiliuguan Coutry, Guangdong Province: ZMB 27661 (holotype of *Amblycephalus monticola kuangtungensis*); Longtoushan Forest Park, Guangzhou City, Guangdong Province: ZMB 65437 (paratype of *Amblycephalus monticola kuangtungensis*); Mt. Shiwan, Guangxi Province: CIB GX201304417; Loshiang, Mt. Yao Shan, Guangxi Province: ZMB 65430. Taiwan: Alishan Towship, Chiayi City: NMW 28130.3, 28130.7–9 & ZMB 30585; Kaoshiung City: NMW 28130.20; specific locations not available: NMW 28130.12, 14, 16, 18. Vietnam: Mt. Fansipan, Sapa District, Lao Cai Province: MNHN 1908.206 (holotype of Eberhardtia tonkinensis) & NHMUK 1912148 (formerly BMNH 1930.11.16.11); Tam Dao N.P., Vinh Phuc Province: NMW 39665.1; Pu Mat N.P., Nghe An Province: FMNH 255567; Kon Tum Province: NHMUK 1912153 (formerly BMNH 1927.5.20.12); Di Linh District, Lam Dong Province: DTU 488–89.

Pareas ***(Eberhardtia)*** geminatus (8 specimens). China: Jiangcheng Coutry, Yunnan Province: CIB 118021–23, DL 20190930001–2, DL 2019072910. Laos: Houaphanh Province: MNHN 171S. Thailand: Tak Province: QSMI 1013.

Pareas ***(Eberhardtia)*** hamptoni (5 specimens). Myanmar: Mogok, upper Burma now Mandalay Division: NHMUK 430223 (formerly BMNH 1904.4.26.16) [holotype of Amblycephalus hamptoni]; Naung Mon, Putao District, Kachin State: CAS 221489. Vietnam: Sa Pa District, Lao Cai Province: MNHN-RA 1935.87–88 & RMNH 6512.

***Pareas (Eberhardtia)*** ***kaduri* (1 specimen)**. Myanmar: Hkakabo Razi NP, Kachin State: CAS 224415.

***Pareas (Eberhardtia)*** ***komaii* (9 specimens)**. **Taiwan**: Alishan Towship, Chiayi City: NMW 28130.1–2, 28130.5, 28130.10; Kaoshiung City: NMW 28130.21–24; specific locations not available: NMW 2)8130.13.

***Pareas (Eberhardtia)*** ***macularius* (15 specimens)**. China: Jianfengling, Hainan Province: CIB 10155 (725035); Jiangcheng Coutry, Yunnan Province: DL 2019.07.29012. **Myanmar**: Ban Mauk, Sagaing Division: ZMMU R-16629; Bago, Bago Division: CAS 206620; Dawei, Tanintharyi Division: CAS 247899; Martaban, Tanintharyi Division: NHMUK 1912159 (formerly BMNH 1946.1.20.8) [holotype of *Pareas macularius*]. **Thailand**: Dansai District, Loei Province: FMNH 135331; **Vietnam**: Ngan Son District, Bac Kan Province: FMNH 175332; Tam Dao N.P., Vinh Phuc Province: MNHN 1938.89, 1938.148 (syntypes of *Amblycephalus tamdaoensis*) & NMW 39964.1; Ba Vi N.P., Ha Noi Capital: DTU 479; Nghe An Province: ZFMK 82925; Phong Nha-Ke Bang N.P., Quang Binh Province: ZFMK 86446; Lam Dong Province: BMNH 1947.1.1.14.

***Pareas (Eberhardtia)*** ***margaritophorus* (51 specimens)**. **Cambodia**: Areng Chum Noab, Koh Kong Province: FMNH 267738; Phnom Kulen, Siem Reap Province: ZFMK 92636–37, 90378; specific locations not available: NMW 28128:4. **China**: Hong Kong City: FMNH 256973, 71137, SMF 20791–20792; Lo Fou Shan, Guangzhou Province: SMF 20790 (holotype of *Pareas moellendorff*); Hainan Province: CAS 14949, FMNH 6661, 66621; Yuling, Hainan Province: CIB 10160 (705015); Diaoluo Shan, Hainan Province: CIB 83792 (665082), 10157 (665081), 10158 (665080); Wuzhi Shan, Hainan Province: CIB 10162 (64III5159). **Malaysia**: Pahang State: FMNH 233357; Nahe Ibok, Terengganu State: NMW 39964.1; Kuala Lumpur Capital: ZFMK 70584; Perak State: ZMB 50680. **Thailand**: Pattani Province: FMNH 178389; Chiangmai Province: FMNH 178390, ZFMK 76107; Nakhon Ratchasima Province: FMNH 180219–20; Na Di District, Prachinburi Province: FMNH 263791; specific locations not available: MNHN 599 (holotype of *Leptognathus margaritophorus*). **Vietnam**: Bai Tu Long N.P., Quang Ninh Province: ZFMK 95197; Nghe An Province: ZFMK 82924; Ke Go N.R., Ha Tinh Province: ZFMK 81479; Phong Nha-Ke Bang N.P., Quang Binh Province: ZFMK 80664; Phuoc Son District, Quang Nam Province: NMW 28128. 6, 28128.8–10, 28128.12; An Khe District, Gia Lai Province: FMNH 252128; specific locations not available: NMW 28128:2–3, 28128.5, 28129.3 & ZSM 22710.

***Pareas (Eberhardtia)*** ***modestus* (8 specimens).** **Mizoram, India:** Sawleng Village, Aizawl District: MZMU 274–275, 1293; Selesih Village, Aizawl District: MZMU 1487; Tanhril, Aizawl District: MZMU 1193; Suangpuilawn Village, Aizawl District: MZMU 1604; Aizawl District: MZMU 1665; Khawrihnim, Mamit District: MZMU 1537.

Pareas ***(Eberhardtia)*** monticola (24 specimens). China: Motuo Coutry, Southeast Xizang: CIB 10163. India: Naga Hillas, Assam State: NHMUK 1912235 (formerly BMNH 1946.1.20.5) [holotype of Dipsas monticola]; Shillong District, Khasi Hills, Meghalaya State: NHMUK 1912240 (formerly BMNH 1907.12.16.26) & BMNH 60.3.19.1312; Darjiling, west Bengal: NHMUK 1912242a,b,c,d (formerly BMNH 1909.3.9.18–21; respectively), NHMUK 1912238 (formerly BMNH 1880.11.10.147), NMW 28127 & ZMH R05510; Jaipur Capital, Rajasthan State (erroneous): NHMUK 1912244 (formerly BMNH 1910.12.31.7); Mishmi Hills, Arunachal Pradesh State: NHMUK 1912246 (formerly BMNH 1940.3.7.2); Abor Hills, Arunachal Pradesh State: NHMUK 1912245a,b (formerly BMNH 1940.3.9.15–16; respectively); Mizoram State: MZMU 851, 1335, 1485–1486. Myanmar: Indawgyi Township, Kachin State: ZMMU R-16630; Ban Mauk Township, Sagaing Division: ZMMU R-16631–16634.

***Pareas (Eberhardtia)*** ***niger* (2 spciemens).** **Yunnan Province, China**: Kunming City: DL 2019.05.29; Honghe City: MH 2015.08S001.

Pareas ***(Eberhardtia)*** stanleyi (4 specimens). China: Fujian Province: CIB 10165; Fukien, Ch'unganHsien now Wuyishan City, Fujian Province: FMNH 24990–92.

Pareas ***(Eberhardtia)*** vidumi (1 specimen). Myanmar: Lukpwi Village, Chipwi Township, Kachin State: CAS 248147 (holotype of Pareas vidumi).

***Pareas (Eberhardtia)*** xuelinensis **(3 spciemens).** **Thailand**: Chiangmai Province, Doi Inthanon NP: AUP 01573, AUP 00175-76.***Pareas* cf. *(Eberhardtia)*** yunnan***ensis* (3 spciemens).** **China**: Yunnan Province, Dali City: ZMB 27660, ZMB 65431 (holotype of *Amblycephalus yunnanensis)* and MH no label.

***Pareas (Pareas) abros* sp. nov. (3 specimens)**. **Vietnam**: Song Thanh N.P., Quang Nam Province: ZMMU R-16393 (holotype of *Pareas abros*); Sao La N.R., Thua Thien-Hue Province: ZMMU R-14788 & R-16392 (paratypes of *Pareas abros*).

***Pareas (Pareas) berdmorei* (20 specimens)**. **Cambodia**: Kampong Speu (or Kampong Spoe), Kampong Spoe Province: MNHN 1938.0149 (holotype of *Pareas carinatus unicolor*); MNHN 1970.480: specific locations not available. **Laos**: Nahin, Khammuoan Province: ZMMU R-16801 (holotype of *Pareas berdmorei annamiticus*); Luang Prabang Province: MNHN RA-1896.655–56, RA-1896.656–57; NHMUK 62.7.28.8: specific locations not available. **Myanmar**: Mon State: ZSI 8022 (holotype of *Pareas berdmorei*); Kinpon Chaung Village, Kyaikhto Township, Mon State: CAS 240362. **Thailand**: Suan Phueng District, Ratchaburi Province: ZMMU R-16803. **Vietnam**: Lai Chau Province: NHMUK 1912147 (formerly BMNH 1924.12.9.202); Tam Dao N.P., Vinh Phuc Province: NMW 39664.3; Gia Bac Commune, Di Linh District, Lam Dong Province: SIEZC 20216; Cat Tien N.P., Dong Nai Province: ZMMU NAP-10584–85; DTU 472–74; Trang Bom District, Dong Nai Province: MNHN RA-1937.27.

***Pareas (Pareas) carinatus* (26 specimens)**. **Indonesia**: Bogor City, West Java Province: SMF 20797, 25995; Karimundjava Island, Central Java Province: SMF 55295; East Java: ZMH R11546-48; West Java: ZMH R11542; specific locations not available in Java: RMNH 954 (Syntype of *Amblycephalus carinatus*); NMW 28134.3–4, 28134.8, ZMH R05520.1; Ranau Lake, Sumatra: SMF 37825–26; north Sumatra: ZSM 154.199; Kutai N.P., East Kalimantan, east Borneo: ZMH 4053; Moara Terweh, central Kalimantan, Borneo: NMW 28131.3. **Malaysia**: Frazers Hills, Pahang State: NMW 39664.2; Terengganu State: NMW 39664.15; specific locations not available in west Malaysia: NMW 39664.9, 39664.11; Borneo Island: NMW 28131.1–2. **Thailand**: Suan Phueng, Ratchaburi Province: ZMMU R-16800.

***Pareas (Pareas) kuznetsovorum* sp. nov. (1 specimen)**. **Vietnam**: Song Hinh District, Phu Yen Province: ZMMU R-16802 (holotype of *Pareas kuznetsovorum*).

***Pareas (Pareas)*** ***nuchalis* (9 specimens)**. **Sumatra, Indonesia**: Kepahiang, Bengkulu Province: USNM 070863; Indragiri, Riau Province: ZMH R3971. **Borneo, Malaysia**: Saribas, Betong Division, Sarawak State: NHMUK 1912247 (holotype of *Amblycephalus nuchalis*); Niah N.P., Sarawak State: FMNH 131635–36; Bintulu Town, Sarawak State: FMNH 269040–41; Tenom District, Sabah State: FMNH 239902–03.

***Pareas (Pareas) temporalis* (6 specimens)**: **Vietnam**: Cat Tien N.P, Cat Loc District, Lam Dong Province: ZMMU R-13656; Gia Bac Commune, Di Linh Distric, Lam Dong Province: SIEZC 20214, DTU 471, 487-488; Biduop-Nui Ba N.P., Lam Dong Province: SIEZC 20215.
