# Supplementary material for: An integrative taxonomic revision of slug-eating snakes (Squamata: Pareidae: Pareineae) reveals unprecedented diversity in Indochina
Source: PeerJ. 2022 Jan 10;10:e12713. doi: 10.7717/peerj.12713 (PMC8757378; doi:10.7717/peerj.12713)
Supplement: Supplemental Information 19 — For each sequence the GenBank Accession Number, museum voucher ID and the species name is provided, for details see Table S1. [file peerj-10-12713-s019.pdf]

SEQUENCES OF PAREINAE

NEWLY GENERATED FOR THE PRESENT STUDY

(NB: For each sequence the GenBank Accession Number, museum voucher ID and the species name is provided, for details see Supplementary Table S1).

cyt b **mtDNA gene**

>MZ712215_NMNH05625_[Pareas_komaii]

ATGTTTCACCAACACACACTAAAACCATTTAACCTCCTACCCGTAAGCACAAACATTTCA

ACCTGGTGAAATTTCGGGTCTATGTTATTAACCTGCCTAGTAATCCAGACCATAACCGGA

CTCTTTCTAGCCATACACTACACAGCCGACATCAACATAGCATTCTCATCCATCATCCAC

ATTACCCGAGATGTGCCCCACGGATGGGTCATACAAAACACACACGCCATTGGAGCCTCA

TTATTCTTTATCTGCGTCTACACCCATGTCGCACGTGGCCTCTATTACGGCTCCTACCTA

AATAAAGAAGTTTGAATAACAGGTACAACCCTAATAATCACCCTAATAGCAACCGCCTTC

TTTGGCTATGTGTTACCATGAGGACAAATATCATTCTGAGCAGCAACAGTTATCACAAAC

CTACTAACCGCTGTGCCCTACTTAGGTAACACTCTAACCACCTGACTCTGAGGCGGCTTT

GCCATTAACAGCCCAACCCTTACTCGATTCTTTGCCCTACACTTCATTCTGCCATTCACT

ATCATCGCCCTAACCTCAATCCACATCATCTTACTACACAACCACGGCTCCAGCAACCCG

CTGGGGACCAACTCAGATATTGATAAAATCCCATTCCACCCCTATCACTCCCACAAAGAC

CTCCTACTAATATCTCTACTAATTACCATCCTACTGTTAGCCATATCATTTTTCCCAAAT

ATACTCAACGACCCAGAAAACTTCTCTAAAGCCAACCCACTGGTCACACCACAACACATC

AAACCCGAGTGATACTTTCTATTTGCTTATGGAATTCTCCGCTCAGTTCCAAATAAATTA

GGAGGGGCGTTGGCCTTGGTTTTAGCCATTTGCATCCTCCTCACAACACCATTTACCCAC

ACAAGCTACATCCGATCAATATCCTTCCGTCCAATAGCCCAAATAGCATTTTGAACCCTC

ATTACCACCTTCATCACCCTCACATGAGCAGCAACCAAACCCGTAGAAACCCCTTACACA

ACTATCGGCCAAATTGCCTCATTCCTGTACTTCTCATTCTTTGCTATTAACCCAATACTG

GGTTGACTAGAAAATAAAACCCTAAAAACCCCTAACTGC

>MZ712216_NMNH05654_[Pareas_iwasakii]

CTACCCGTGAGCACAAACATTTCAACCTGGTGAAATTTCGGATCTATGTTATTAACCTGC

TTAATAATCCAAACCATAACCGGATTTTTCCTAGCCATACACTACACAGCCAACATCAAC

ACGGCATTCTCATCCATCATCCACATCACCCGAGATGTGCCCCACGGGTGGGTCATACAA

AACACACACGCCATTGGGGCCTCCTTGTTCTTTATCTGCGCCTACACCCATATTGCACGA

GGTATTTACTACGGCTCCTACCTAAATAAAGAAGTCTGAATAACAGGTACAACACTAATA

ATCACCCTAATAGCAACTGCCTTCTTTGGCTACGTGTTACCATGAGGACAAATATCGTTC

TGAGCAGCAACAGTTATCACAAACCTGCTAACCGCTGTACCCTACTTAGGTAACACCCTA

ACCACTTGACTTTGGGGTGGTTTTGCCATTAACGACCCAACCCTTACTCGATTCTTTGCC

CTACATTTCATTCTACCATTTACTATCGTCGCCTTAACCTCAACCCACGTCATCTTACTA

CACAACCAAGGCTCCAGTAACCCATTAGGCACCAACTCAGACATTGATAAAATCCCATTC

CACCCCTACCACTCCTACAAAGACCTCTTACTAATAACCACACTAATTACCACCTTGTTA

GTAATCTTAACTTTTTTCCCAAATATACTCAACGACCCAGAAAATTTCTCTAAAGCCAAC

CCACTGGTCACACCACAACACATCAAACCCGAGTGATACTTTCTATTTGCTTATGGAATT

CTTCGCTCAGTTCCAAATAAATTAGGAGGGGCGTTGGCCTTGGTTTTAGCCGTTTGTATC

CTCCTCACAACACCATTCACCCACACAAGCTACATCCGATCAATATCCTTCCGTCCAGTA

GCCCAAATAGCATTTTGAACCCTCATCACCACCTTCATCATCCTCACATGAGCAGCAACC

AAACCAGTAGAGACCCCCTACACAACTATCGGCCAAGTTGCCTCATTCCTGTACTTCTCA

TTC

>MZ712217_ZMMUNAP09759_[Pareas_margaritophorus]

ATGCTCCACCAACACACACTAACACTATTTAACCTACTGCCCGTAGGTTCAAACATTTCA

ACCTGATGGAACTTCGGTTCTATGCTAATAACTTGCCTACTAATCCAAACCCTGACCGGG

TTATTCCTATCCATACACTATACAGCCAACATCAACATAGCATTTTCATCCATTATACAC

ATTTCCCGAGACGTCCCATACGGATGACTCGTACAAAACACACACGCCATCGGGGCATCT

CTATTCTTTATCTGCATCTACACCCACATCGCACGCGGCCTCTACTACGGCTCCTATTTA

AATAAAGAAGTCTGAATCTCGGGCACTCTCCTACTAATCACCCTAATAGCAACAGCCTTC

TTTGGCTACGTCCTACCATGAGGACAAATATCATTCTGAGCGGCAACCGTAATCACAAAC

TTATTAACCGCAGTACCATACCTAGGAAACACCCTCACCACTTGAGTGTGGGGGGGATTC

GCCATCGACAATCCAACTCTGACCCGATTCTTCGCCCTCCACTTCCTTCTCCCATTCGGA

CTCATCGCTATAACCTCAATCCATGTTATCCTACTACACAACGTAGGATCCAACAACCCC

CTTGGAACCAACTCAGACACCGATAAAATCCCATTCCACCCGTACCACTCCTACAAAGAC

TTCTTACTAATAACAATACTAGTAACTAGCCTACTATTAACCCTATCCTTTTTCCCAAAC

TCACTGAATGACCCAGAAAACTTCTCCAAAGCAAACCCCATAATCACCCCACAACACATC

AAACCAGAGTGATACTTTCTATTTGCTTACGGAATCCTCCGTTCCGTACCAGACAAACTA

GGAGGAGTGTTGGCCTTGATCTTAGCCGTCTGTATCCTACTCACACTACCATTCACCCAC

ACTAGTCCCATCCGATCAATATCCTTCCGACCACTAGCACAAACAGCATTTTGAACCCTC

ATCACAACCTTTATAATCCTCACATGAACAGCCACTAAACCAGTAGAACCCCCCTTTACA

ACCATCAGTCAAGCAGCCTCACTACTATACTTTTCATTTTTTGTTTCCGGCCCAGTCCTT

GGACTATTAGAAAACAAAACCCTAAAAACCCCTAACTGC

>MZ712218_AUP01573_[Pareas_berdmorei_berdmorei]

TTTCACCAACACATACTTATCATTATTAACCTCCTCCCTGTAAGCTCCAACATCTCAACA

TGATGAAACTTTGGTTCAATACTTATATCCTGTTTGATAATTCAAATCATAACTGGATTC

TTCCTAGCTATTCACTACACAGCCAACATCAACATAGCATTCTCATCCATTGTTCATATT

TCACGAGACGTACCATACGGATGAATTATACAAAACATTCACGCCATTGGAGCCTCCCTG

TTCTTCATTTGCATTTATATCCACATTGCACGAGGCCTCTATTACGGATCCTACTTAAAT

AAAGAAGTATGAATATCAGGCATAATTCTTTTAATTACCCTTATAGCAACAGCCTTCTTT

GGCTACGTCCTGCCCTGAGGACAAATATCTTTTTGAGCTGCAACAGTAATCACCAACCTA

TTAACTGCAGTACCATATTTAGGAGTAACATTAACAACCTGGTTCTGAGGAGGATTTGCA

ATTAACGATCCAACCCTCACTCGATTCTTTGCCCTTCACTTTATTCTACCATTCGGATTA

CTATCCTTAGCCATAATGCACATTATACTTCTACATAACGAAGGCTCTAATAACCCACTG

GGAACAAATTCAAATATTGACAAAATCCCATTACACCCATATCACTCTTACAAAGACATT

CTATTAGCAACCATCATAATTACCGTCCTGTTTACTATTTTAACTTTTTTTCCAAATATA

TTAAACGACCCAGAAAATTTTTCCAAAGCAAATCCATTAATCACACCTCAACACATCAAA

CCAGAATGGTACTTTCTATTCGCTTACGGAATTCTACGATCAATTCCAAATAAACTAGGA

GGAGCTATAGCCCTGATCTTATCCATCTGCATTCTATTCACAACACCATTTACCCACACA

AGTTCAATCCGAACTATATCCTTTCGCCCTATAGCACAAACAATATTCTGAACCCTAATT

ATCACCTCCATTATCATTACCTGAACAGCCACTAAAACAGTAGAACCACCATTCACCATT

ATTAGCCAAATAGCCTCATTATTGTATTTCTCATATTTCATCATTAGTCCCCTGTTAGGC

CTAGTGGAAAACAAAATAACAAAAACCCAT

>MZ712219_CAS240362_[Pareas_berdmorei_berdmorei]

ACAAAAATGTTACACCAACACATACTTATCATTATTAACCTCCTCCCTGTAAGCTCCAAC

ATCTCAACATGATGAAACTTTGGTTCAATACTTATATCCTGTCTGATAGTTCAAATCATA

ACTGGATTCTTCCTAGCTATTCACTACACAGCCAACATCAACATAGCATTCTCATCCATT

GTTCATATTTCACGAGACGTACCATACGGATGAATTATACAAAACATTCACGCCATTGGA

GCCTCTCTATTCTTCATTTGCATTTATATCCACATTGCACGAGGCCTCTATTACGGATCC

TACTTAAATAAAGAAGTATGAATATCAGGCATAATTCTTTTAATTACCCTTATAGCAACA

GCCTTCTTTGGCTACGTCCTGCCCTGAGGACAAATATCTTTTTGAGCTGCAACAGTAATC

ACCAACCTATTAACTGCAGTACCATATTTAGGAGTAACATTAACAACCTGGTTCTGAGGA

GGGTTTGCAATTAACGATCCAACCCTCACTCGATTCTTTGCCCTTCACTTTATTCTACCA

TTCGGGTTACTATCCTTAGCCATAATGCACATTATACTTCTACATAACGAAGGCTCTAAT

AACCCACTAGGAACAAATTCAAATATTGACAAAATCCCATTACACCCATATCACTCTTAC

AAAGACATTCTATTAGCAACCATCATAATTACCGTCCTGTTTATTATTTTAACTTTTTTT

CCAAATATATTAAACGACCCAGAAAATTTTTCCAAAGCAAATCCATTAATCACACCTCAA

CACATCAAACCAGAATGGTACTTTCTATTCGCTTACGGAATTCTACGATCAATTCCAAAT

AAACTAGGAGGAGCTATAGCCCTAATCTTATCCATCTGCATTCTATTCACAACACCATTT

ACCCACACAAGTTCAATCCGAACTATATCCTTTCGCCCTATAGCACAAACAATATTCTGA

ACCCTAATTATCACCTCCATTATCATTACCTGAACAGCCACTAAAACAGTAGAACCACCA

TTCACCATTATTAGCCAAATAGCCTCATTATTGTATTTCTTATATTTCATCATTAGTCCC

CTGTTAGGTCTAGTGGAAAACAAAATTACAAAAACCCATATCTGCT

>MZ712220_ZMMUR16803_[Pareas_berdmorei_berdmorei]

ACAAAAATGTTACACCAACACATACTTATCATTATTAACCTCCTCCCTGTAAGCTCCAAC

ATCTCAACATGATGAAACTTTGGTTCAATACTTATATCCTGTCTGATAGTTCAAATCATA

ACTGGATTCTTCCTAGCTATTCACTACACAGCCAACATCAACATAGCATTCTCATCCATT

GTTCATATTTCACGAGACGTACCATACGGATGAATTATACAAAACATTCACGCCATTGGA

GCCTCTCTATTCTTCATTTGCATTTATATCCACATTGCACGAGGCCTCTATTACGGATCC

TACTTAAATAAAGAAGTATGAATATCAGGCATAATTCTTTTAATTACCCTTATAGCAACA

GCCTTCTTTGGCTACGTCCTGCCCTGAGGACAAATATCTTTTTGAGCTGCAACAGTAATC

ACCAACCTATTAACTGCAGTACCATATTTAGGAGTAACATTAACAACCTGGTTCTGAGGA

GGGTTTGCAATTAACGATCCAACCCTCACTCGATTCTTTGCCCTTCACTTTATTCTACCA

TTCGGGTTACTATCCTTAGCCATAATGCACATTATACTTCTACATAACGAAGGCTCTAAT

AACCCACTAGGAACAAATTCAAATATTGACAAAATCCCATTACACCCATATCACTCTTAC
AAAGACATTCTATTAGCAACCATCATAATTACCGTCCTGTTTATTATTTTAACTTTTTTT
CCAAATATATTAAACGACCCAGAAAATTTTTCCAAAGCAAATCCATTAATCACACCTCAA
CACATCAAACCAGAATGGTACTTTCTATTCGCTTACGGAATTCTACGATCAATTCCAAAT
AAACTAGGAGGAGCTATAGCCCTAATCTTATCCATCTGCATTCTATTCACAACACCATTT
ACCCACACAAGTTCAATCCGAACTATATCCTTTCGCCCTATAGCACAAACAATATTCTGA
ACCCTAATTATCACCTCCATTATCATTACCTGAACAGCCACTAAAACAGTAGAACCACCA
TTCACCATTATTAGCCAAATAGCCTCATTATTGTATTTCTTATATTTCATCATTAGTCCC
CTGTTAGGTCTAGTGGAAAACAAAATTACAAAAACCCATATCTGCT

>MZ712221_ZMMUR16801_[Pareas_berdmorei_truongsonicus]

TTACACCAACATACACTTATCACTATTAACCTCCTCCCTGTAAGTTCTAACATCTCAACA
TGATGAAACTTTGGTTCAATACTTATAACCTGTCTGATAATTCAAATCATAACCGGATTT
TTCCTAGCTATTCACTACACAGCCAACATCAACATAGCATTTTCATCCGTTATTCATATT
TCACGAGACGTACCATACGGATGAATCATACAAAATATTCACGCCATCGGAGCCTCTCTA
TTCTTCATTTGCATTTATATCCACATTGCACGAGGTCTCTATTACGGGTCCTACTTAAAT
AAAGAAGTATGAATATCGGGCATAATTCTTCTAATTACCCTCATAGCAACAGCCTTCTTT
GGTTACGTCCTGCCCTGAGGACAAATATCTTTTTGAGCTGCAACAGTAATCACCAACCTA
TTAACTGCAGTACCATATTTAGGAGTAACATTAACAACCTGGTTCTGAGGCGGATTTGCA
ATTAACGATCCTACCCTCACTCGATTCTTTGCCCTTCACTTTATTCTACCATTTGGATTA
CTATCTTTAACCATAATACACATTATACTTCTTCACAACGAAGGCTCTAACAATCCACTG
GGAACAAATTCAAATATTGACAAAATCCCATTACACCCATATCACTCTTACAAAGACATT
CTATTAGCAACCATCATAATTACCGTCCTGTTTATTATTTTAACTTTTTTCCCAAATATA
TTAAACGACCCAGAAAATTTTTCTAAAGCAAACCCATTAATCACACCTCAACACATTAAA
CCAGAGTGATACTTTCTATTCGCTTACGGAATTCTACGATCAATTCCGAATAAACTAGGT
GGAGCTATAGCCCTAATCTTATCTATCTGCATTCTATTCACAACGCCATTCACCCACACA
AGTTCAATCCGAACTATATCCTTTCGTCCTATAGCACAAACAATATTCTGAACCCTTATT
ATCGCCTCCATTATCATTACCTGAACAGCCACTAAAACAGTAGAACCCCCATTCACTATT
ATCAGCCAAATAGCCTCATTATTGTATTTCTCATATTTCATCATTAGTCCCCTGTTAGGC
CTAATAGAAAACAAAATT

>MZ712222_ZMMUR14796_[Pareas_berdmorei_truongsonicus]

TTACACCAACATACACTTATCACTATTAACCTCCTCCCTGTAAGTTCTAACATCTCAACA

TGATGAAACTTTGGTTCAATACTTATAACCTGTCTGATAATTCAAATCATAACCGGATTT

TTCCTAGCTATTCACTACACAGCCAACATCAACATAGCATTTTCATCCGTTATTCATATT

TCACGAGACGTACCATACGGATGAATCATACAAAATATTCACGCCATCGGAGCCTCTCTA

TTCTTCATTTGCATTTATATCCACATTGCACGAGGTCTCTATTACGGGTCCTACTTAAAT

AAAGAAGTATGAATATCGGGCATAATTCTTCTAATTACCCTCATAGCAACAGCCTTCTTT

GGTTACGTCCTGCCCTGAGGACAAATATCTTTTTGAGCTGCAACAGTAATCACCAACCTA

TTAACTGCAGTACCATATTTAGGAGTAACATTAACAACCTGGTTCTGAGGCGGATTTGCA

ATTAACGATCCTACCCTCACTCGATTCTTTGCCCTTCACTTTATTCTACCATTTGGATTA

CTATCTTTAACCATAATACACATTATACTTCTTCACAACGAAGGCTCTAACAATCCACTG

GGAACAAATTCAAATATTGACAAAATCCCATTACACCCATATCACTCTTACAAAGACATT

CTATTAGCAACCATCATAATTACCGTCCTGTTTATTATTTTAACTTTTTTCCCAAATATA

TTAAACGACCCAGAAAATTTTTCTAAAGCAAACCCATTAATCACACCTCAACACATTAAA

CCAGAGTGATACTTTCTATTCGCTTACGGAATTCTACGATCAATTCCAAATAAACTAGGT

GGAGCTATAGCCCTAATCTTATCTATCTGCATTCTATTCACAACGCCATTCACCCACACA

AGTTCAATCCGAACTATATCCTTTCGTCCTATAGCACAAACAATATTCTGAACCCTTATT

ATCGCCTCCATTATCATTACCTGAACAGCCACTAAAACAGTAGAACCCCCATTCACTATT

ATCAGCCAAATAGCCTCATTATTGTATTTCTTATATTTCATCATTAGTCCCCTGTTAGGC

CTAATAGAAAACAAAATTCTAAAAACCCAT

>MZ712223_ZMMUR137531_[Pareas_berdmorei_unicolor]

CAAAAAATGTTACACCAACACATACTTATCATTATTAACCTCCTCCCTGTGAGCTCTAAC

ATCTCAACATGATGGAACTTTGGTTCAATACTCATAACCTGCCTGATAATTCAAATCATA

ACCGGATTTTTCCTAGCTATTCACTATACAGCCAACATCAACATAGCATTTTCATCCATT

ATTCACATTTCACGAGACGTACCATACGGATGAATCATACAAAACATTCACGCCATCGGA

GCTTCTCTATTCTTCATTTGTATTTATATCCACATTGCACGAGGCCTCTATTACGGGTCC

TACCTAAATAAAGAAGTATGAATATCAGGCATAATTCTTCTAATTACCCTTATAGCAACA

GCTTTCTTTGGTTATGTCCTGCCCTGAGGACAAATATCTTTTTGAGCCGCAACAGTAATT

ACTAATCTACTAACTGCAGTACCATATTTAGGAATAACATTAACAACCTGGTTATGAGGC

GGATTTGCAATTAACGATCCAACCCTCACTCGATTCTTTGCCCTTCACTTTATCCTACCA

TTTGGATTACTATCTCTAACCACAATACACATTATACTTCTTCACAACAAAGGATCTAAT

AACCCACTGGGAACAAATTCAAATATTGACAAAATCCCATTACACCCATATCACTCTTAC

AAAGATATTCTATTAGCAACCACCTTAATTACTGTCCTGTTTATTATTTTAACCTTTTTT

CCAAATACACTAAACGACCCAGAAAATTTTTCCAAGGCAAATCCATTAATCACACCTCAA

CACATTAAACCAGAGTGATACTTCCTATTCGCTTACGGAATTCTACGATCGATTCCAAAT

AAATTAGGCGGAGCTATAGCCCTAGTCTTATCTATTTTCATTCTATTCACAACACCATTC

ACCCATACAAGTTCAATCCGAACTATATCCTTTCGCCCTATAGCACAAACAATATTCTGA

ACTCTCATTATCACCTCCATTATCATTACCTGAACAGCCACTAAAACAGTAGAACCCCCA

TTCATTTTTATCAGCCAAATAGCCTCATTACTGTATTTCTCATATTTCATCATTAGTCCA

CTGCTAGGCCTAATGGAAAACAAAATTCTAAAAACCCCAATCTGCT

>MZ712224_ZMMUR137532_[Pareas_berdmorei_unicolor]

CCAAAAATGTTACACCAACACATACTTATCATTATTAACCTCCTCCCTGTGAGCTCTAAC

ATCTCAACATGATGGAACTTTGGTTCAATACTCATAACCTGCCTGATAATTCAAATCATA

ACCGGATTTTTCCTAGCTATTCACTATACAGCTAACATCAACATAGCATTTTCATCCATT

ATTCACATTTCACGAGACGTACCATACGGATGAATCATACAAAACATTCACGCCATCGGA

GCTTCTCTATTCTTCATTTGCATCTATATCCACATTGCACGAGGCCTCTATTACGGGTCC

TACCTAAATAAAGAAGTATGAATATCAGGCATAATTCTTCTAATTACCCTTATAGCAACA

GCTTTCTTTGGTTATGTCCTGCCCTGAGGACAAATATCTTTTTGAGCCGCAACAGTAATT

ACTAATCTACTAACTGCAGTACCATATTTAGGAATAACATTAACAACCTGGTTGTGAGGC

GGATTTGCAATTAACGATCCAACCCTCACTCGATTCTTTGCCCTTCACTTTATCCTACCA

TTTGGATTACTATCTCTAACCATAATACACATTATACTTCTTCACAACAAAGGATCTAAT

AACCCACTGGGAACAAATTCAAATATTGACAAAATCCCATTACACCCATATCACTCTTAC

AAAGATATTCTATTAGCAACCATCTTAATTACTGTCCTGTTTATTATTTTAACCTTTTTT

CCAAATATATTAAACGACCCAGAAAATTTTTCCAAGGCAAACCCATTAATCACACCTCAA

CACATTAAACCAGAGTGATACTTCCTATTCGCTTACGGAATTCTACGATCAATTCCAAAT

AAATTAGGCGGAGCTATAGCCCTAGTCTTATCTATTTTCATTCTATTCACAACACCATTC

ACCCATACAAGTTCAATCCGAACTATATCCTTTCGCCCTATAGCACAAACAATATTCTGA

ACTCTCATTATCACCTCCATTATCATTACCTGAACAGCCACTAAAACAGTAGAACCCCCA

TTCATTTTTATCAGCCAAATAGCCTCATTACTATATTTCTCATATTTCATCATTAGTCCA

CTGCTAGGCCTAATGGAAAACAAAATTCTAAAAACCCCAATCTGCT

>MZ712225_ZMMUR14013_[Pareas_berdmorei_unicolor]

CAAAAAATGTTACACCAACACATACTTATCATTATTAACCTCCTCCCTGTGAGCTCTAAC

ATCTCAACATGATGGAACTTTGGTTCAATACTCATAACCTGCCTGATAATTCAAATCATA

ACCGGATTTTTCCTAGCTATTCACTATACAGCCAACATCAACATAGCATTTTCATCCATT

ATTCACATTTCACGAGACGTACCATACGGATGAATCATACAAAACATTCACGCCATCGGA

GCTTCTCTATTCTTCATTTGTATTTATATCCACATTGCACGAGGCCTCTATTACGGGTCC

TACCTAAATAAAGAAGTATGAATATCAGGCATAATTCTTCTAATTACCCTTATAGCAACA

GCTTTCTTTGGTTATGTCCTGCCCTGAGGACAAATATCTTTTTGAGCCGCAACAGTAATT

ACTAATCTACTAACTGCAGTACCATATTTAGGAATAACATTAACAACCTGGTTATGAGGC

GGATTTGCAATTAACGATCCAACCCTCACTCGATTCTTTGCCCTTCACTTTATCCTACCA

TTTGGATTACTATCTCTAACCACAATACACATTATACTTCTTCACAACAAAGGATCTAAT

AACCCACTGGGAACAAATTCAAATATTGACAAAATCCCATTACACCCATATCACTCTTAC

AAAGATATTCTATTAGCAACCACCTTAATTACTGTCCTGTTTATTATTTTAACCTTTTTT

CCAAATATACTAAACGACCCAGAAAATTTTTCCAAGGCAAATCCATTAATCACACCTCAA

CACATTAAACCAGAGTGATACTTCCTATTCGCTTACGGAATTCTACGATCGATTCCAAAT

AAATTAGGCGGAGCTATAGCCCTAGTCTTATCTATTTTCATTCTATTCACAACACCATTC

ACCCATACAAGTTCAATCCGAACTATATCCTTTCGCCCTATAGCACAAACAATATTCTGA

ACTCTCATTATCACCTCCATTATCATTACCTGAACAGCCACTAAAACAGTAGAACCCCCA

TTCATTTTTATCAGCCAAATAGCCTCATTACTGTATTTCTCATATTTCATCATTAGTCCA

CTGCTAGGCCTAATGGAAAACAAAATTCTAAAAACCCCAATCTGCT

>MZ712226_ZMMUR14263_[Pareas_berdmorei_unicolor]

CCAAAAATGTTACACCAACACATACTTATCATTATTAACCTCCTCCCTGTGAGCTCTAAC

ATCTCAACATGATGGAACTTTGGTTCAATACTCGTAACCTGCCTGATAATTCAAATCATA

ACCGGATTTTTCCTAGCTATTCACTATACAGCCAACATCAACATAGCATTTTCATCCATT

ATTCACATTTCACGAGACGTACCATACGGATGAATCATACAAAACATTCACGCCATCGGA

GCTTCTCTATTCTTCATTTGCATTTATATCCACATTGCACGAGGCCTCTATTACGGGTCC

TACCTAAATAAAGAAGTATGAATATCAGGCATAATTCTTCTAATTACCCTTATAGCAACA

GCTTTCTTTGGTTATGTCCTGCCCTGAGGACAAATATCTTTTTGAGCCGCAACAGTAATT

ACTAATCTACTAACTGCAGTACCATATTTAGGAATAACATTAACAACCTGGTTATGAGGC

GGATTTGCAATTAACGATCCAACCCTCACTCGATTCTTTGCCCTTCACTTTATCCTACCA

TTTGGATTACTATCTCTAACCACAATACACATTATACTTCTTCACAACAAAGGATCTAAT

AACCCACTGGGAACAAATTCAAATATTGACAAAATCCCATTACACCCATATCACTCTTAC

AAAGATATTCTATTAGCAACCACCTTAATTACTTTCCTGTTTATTATTTTAACCTTTTTT

CCAAATATATTAAACGACCCAGAAAATTTTTCCAAGGCAAACCCATTAATCACACCTCAA

CACATTAAACCAGAGTGATACTTCCTATTCGCTTACGGAATTCTACGATCGATTCCAAAT

AAATTAGGCGGAGCTATAGCCCTAGTCTTATCTATTTCCATTCTATTCACAACACCATTC

ACCCATACAAGTTCAATCCGAACTATATCCTTTCGCCCTATAGCACAAACAATATTCTGA

ACTCTCATTATCACCTCCATTATCATTACCTGAACAGCCACTAAAACAGTAGAACCCCCA

TTCATTTTTATCAGCCAAATAGCCTCATTACTGTATTTCTCATATTTCATCATTAGTCCA

CTGCTAGGCCTAATGGAAAACAAAATTCTAAAAACCCCAATCTGCT

>MZ712227_ZMMUR14421_[Pareas_berdmorei_unicolor]

CCACAAATGTTACACCAACACATACTTATCATTATTAACCTCCTCCCTGTGAGCTCTAAC

ATCTCAACATGATGGAACTTTGGTTCAATACTCGTAACCTGCCTGATAATTCAAATCATA

ACCGGATTTTTCCTAGCTATTCACTATACAGCCAACATCAACATAGCATTTTCATCCATT

ATTCACATTTCACGAGACGTACCATACGGATGAATCATACAAAACATTCACGCCATCGGA

GCTTCTCTATTCTTCATTTGCATTTATATCCACATTGCACGAGGCCTCTATTACGGGTCC

TACCTAAATAAAGAAGTATGAATATCAGGCATAATTCTTCTAATTACCCTTATAGCAACA

GCTTTCTTTGGTTATGTCCTGCCCTGAGGACAAATATCTTTTTGAGCCGCAACAGTAATT

ACTAATCTACTAACTGCAGTACCATATTTAGGAATAACATTAACAACCTGGTTATGAGGC

GGATTTGCAATTAACGATCCAACTCTCACTCGATTCTTTGCCCTTCACTTTATCCTACCA

TTTGGATTACTATCTCTAACCACAATACACATTATACTTCTTCACAACAAAGGATCTAAT

AACCCACTGGGAACAAATTCAAATATTGACAAAATCCCATTACACCCATATCACTCTTAC

AAAGATATTCTATTAGCAACCACCTTAATTACTGTCCTGTTTATTATTTTAACCTTTTTT

CCAAATATATTAAACGACCCAGAAAATTTTTCCAAGGCAAACCCATTAATCACACCTCAA

CACATTAAACCAGAGTGATACTTCCTATTCGCTTACGGAATTCTACGATCGATTCCAAAT

AAATTAGGCGGAGCTATAGCCCTAGTCTTATCTATTTCCATTCTATTCACAACACCATTC

ACCCATACAAGTTCAATCCGAACTATATCCTTTCGCCCTATAGCACAAACAATATTCTGA
ACTCTCATTATCACCTTCATTATCATTACCTGAACAGCCACTAAAACAGTAGAACCCCCA
TTCATTTTTATCAGCCAAATAGCCTCATTACTGTATTTCTCATATTTCATCATTAGTCCA
CTGCTAGGCCTAATGGAAAACAAAATTCTAAAAACCCCAATCTGCT
>MZ712228_SIEZC20216_[Pareas_berdmorei_unicolor]
CCAAAAATGTCACACAAACACATACTTACCATTATTAACCTCCTCCCTGTGAGCTCTAAC
ATCTCACCATGATGAAACTTTGGTTCAATGCTCATAACCTGTCTGATAATTCAAATCATA
ACTGGATTTTTCCTAGCTATTCACTATACAGCCAACATCAACATAGCATTTTCATCCATT
ATTCACATTTCACGAGACGTGCCATACGGATGAATCATACAAAATATTCACGCCATCGGA
GCTTCTCTATTCTTCATTTGCATTTATATCCACATTGCACGAGGCCTCTATTACGGGTCC
TACCTAAATAAAGAAGTATGAATATCAGGCATAATTCTTCTAATTACCCTTATAGCAACA
GCTTTCTTTGGTTACGTCCTGCCCTGAGGACAAATATCTTTTTGAGCCGCAACAGTAATT
ACTAATCTACTAACTGCAGTACCATATTTAGGAATAACATTAACATCCTGGTTATGAGGC
GGATTTGCAATTAACGATCCAACCCTCACTCGATTCTTTGCCCTTCACTTTATTCTACCA
TTTGGGTTACTATCTCTAACTATAATACACATTTTACTTCTTCACAACGAAGGGTCTAAT
AATCCACTGGGAACAAATTCAAATATTGACAAAATCCCATTACACCCATACCACTCTTAC
AAAGATATTCTATTAGCAACCACTTTAATTACTGTCCTATTTATTATTTTAACCTTTTTT
CCAAATATATTAAACGACCCAGAAAATTTCTCCAAGGCAAACCCACTAATCACACCTCAA
CACATTAAACCAGAATGATACTTCCTATTCGCTTACGGAATTCTACGGTCAATTCCAAAT
AAATTAGGCGGAGCTATAGCCCTGCTCTTATCTATTTTCATTCTATTCACAACACCATTC
ACCCATACAAGTTCAATCCGAACTATATCATTTCGCCCTATAGCACAAACAATATTCTGA
ACTCTCATTATCACCTCCATTATCATTACCTGAACAGCCACTAAAACAGTAGAACCCCCA
TTCATTATTATCAGCCAAATAGCCTCATTATTGTATTTCTCATATTTCATCATTAGTCCA
CTGCTAGGCCTAATGGAAAACAAA
>MZ712229_ZMMUR136791_[Pareas_berdmorei_unicolor]
CCAAAAATGTCACACAAACACATACTTACCATTATTAACCTCCTCCCTGTGAGCTCTAAC
ATCTCAACATGATGGAACTTTGGTTCAATACTCGTAACCTGCCTGATAATTCAAATCATA
ACCGGATTTTTCCTAGCTATTCACTATACAGCCAACATCAACATAGCATTTTCATCCATT
ATTCACATTTCACGAGACGTACCATACGGATGAATCATACAAAACATTCACGCCATCGGA

GCTTCTCTATTCTTCATTTGCATTTATATCCACATTGCACGAGGCCTCTATTACGGGTCC

TACCTAAATAAAGAAGTATGAATATCAGGCATAATTCTTCTAATTACCCTTATAGCAACA

GCTTTCTTTGGTTATGTCCTGCCCTGAGGACAAATATCTTTTTGAGCCGCAACAGTAATT

ACTAATCTACTAACTGCAGTACCATATTTAGGAATAACATTAACAACCTGGTTATGAGGC

GGATTTGCAATTAACGATCCAACTCTCACTCGATTCTTTGCCCTTCACTTTATCCTACCA

TTTGGATTACTATCTCTAACCACAATACACATTATACTTCTTCACAACAAAGGATCTAAT

AACCCACTGGGAACAAATTCAAATATTGACAAAATCCCATTACACCCATATCACTCTTAC

AAAGATATTCTATTAGCAACCACCTTAATTACTGTCCTGTTTATTATTTTAACCTTTTTT

CCAAATATATTAAACGACCCAGAAAATTTTTCCAAGGCAAACCCATTAATCACACCTCAA

CACATTAAACCAGAGTGATACTTCCTATTCGCTTACGGAATTCTACGATCGATTCCAAAT

AAATTAGGCGGAGCTATAGCCCTAGTCTTATCTATTTCCATTCTATTCACAACACCATTC

ACCCATACAAGTTCAATCCGAACTATATCCTTTCGCCCTATAGCACAAACAATATTCTGA

ACTCTCATTATCACCTCCATTATCATTACCTGAACAGCCACTAAAACAGTAGAACCCCCA

TTCATTTTTATCAGCCAAATAGCCTCATTACTGTATTTCTCATATTTCATCATTAGTCCA

CTGCTAGGCCTAATGGAAAACAAA

>MZ712230_ZMMUR136792_[Pareas_berdmorei_unicolor]

CCAAAAATGTCACACAAACACATACTTACCATTATTAACCTCCTCCCTGTGAGCTCTAAC

ATCTCAACATGATGAAACTTTGGTTCAATGCTCATAACCTGTCTGATAATTCAAACCATA

ACTGGATTTTTCCTAGCTATTCACTACACAGCCAACATCAACATAGCATTTTCATCCATT

ATTCACATTTCACGAGACGTACCATACGGATGAATCATACAAAATATTCACGCCATCGGA

GCCTCTCTATTCTTCATTTGCATTTATATCCACATTGCACGAGGCCTCTATTACGGGTCC

TACCTAAATAAAGAAGTATGAATATCAGGCATAATTCTTCTAATTACCCTTATAGCAACA

GCTTTCTTTGGTTACGTCCTGCCCTGAGGACAAATATCTTTTTGAGCCGCAACAGTAATC

ACTAATCTATTAACTGCAGTACCATATTTAGGAATAACATTAACAACCTGGTTATGAGGC

GGATTTGCAATTAACGATCCGACCCTCACTCGATTCTTTGCCCTTCACTTTATTCTACCA

TTTGGATTACTATCTCTAACCATAATACACATTATACTTCTTCACAACGAAGGATCTAAT

AACCCACTGGGAACAAATTCAAATATTGACAAAATCCCATTACACCCATATCACTCTTAC

AAAGATATTCTATTAGCAACCACCTTAATTACTGTCCTGTTTATTATTTTAACCTTTTTT

CCAAATATATTAAACGACCCAGAAAATTTTTCCAAGGCAAACTCATTAATCACACCTCAA

CACATTAAACCAGAGTGATACTTCCTATTCGCTTACGGAATTCTACGATCGATTCCAAAT

AAATTAGGCGGAGCTATAGCCCTAGTCTTATCTATTTTCATTCTATTCACAACACCATTC

ACCCATACAAGTTCAATCCGAACTATATCCTTTCGCCCTATAGCACAAACAATATTTTGA

ACTCTCATTATCACCTCCATTATCATTACCTGAACAGCCACTAAAACAGTAGAACCCCCA

TTCATTATTATCAGCCAAATAGCCTCATTATTGTATTTCTCATATTTCATCATTAGTCCA

CTGCTAGGCCTAATGGAAAACAAA

>MZ712231_ZMMUR14165_[Pareas_berdmorei_unicolor]

CCAAACATGTCACACCAACACATACTTATCATTATTAACCTCCTCCCTGTGAGCTCTAAC

ATCTCAACATGATGAAACTTTGGTTCAATGCTCATAACCTGTCTGATAATTCAAACCATA

ACTGGATTTTTCCTAGCTATTCACTACACAGCCAACATCAACATAGCATTTTCATCCATT

ATTCACATTTCACGAGATGTACCATACGGATGAATCATACAAAATATTCACGCCATCGGA

GCCTCTCTATTCTTCATTTGCATTTATATCCACATTGCACGAGGCCTCTATTACGGGTCT

TACCTAAATAAAGAAGTGTGAATATCAGGCATAATTATTCTAATTACCCTTATAGCAACA

GCTTTCTTTGGTTACGTCCTGCCATGAGGACAAATATCTTTTTGAGCCGCAACAGTAATC

ACTAATCTATTAACTGCAGTACCATATTTAGGAATAACACTAACAACCTGGTTATGAGGC

GGATTTGCAATTAACGATCCAACCCTCACTCGATTCTTTGCCCTTCACTTTATTCTACCA

TTTGGGTTACTATCTCTAACTATAATACACATTTTACTTCTTCACAACGAAGGGTCTAAT

AATCCACTGGGAACAAATTCAAATATTGACAAAATCCCATTACACCCATACCACTCTTAC

AAAGATATTCTATTAGCAACCACTTTAATTACTGTCCTATTTATTATTTTAACCTTTTTT

CCAAATATATTAAACGACCCAGAAAATTTCTCCAAGGCAAACCCACTAATCACACCTCAA

CACATTAAACCAGAATGATACTTCCTATTCGCTTACGGAATTCTACGGTCAATTCCAAAT

AAATTAGGCGGAGCTATAGCCCTGCTCTTATCTATTTTCATTCTATTCACAACACCATTC

ACCCATACAAGTTCAATCCGAACTATATCATTTCGCCCTATAGCACAAACAATATTCTGA

ACTCTCATTATCACCTCCATTATCATTACCTGAACAGCCACTAAAACAGTAGAACCCCCA

TTCATTATTATCAGCCAAATAGCCTCATTATTGTATTTCTCATATTTCATCATTAGTCCA

CTGCTAGGCCTAATGGAAAACAAA

>MZ712232_ZMMUR16802_[Pareas_kuznetsovorum]

TACAAAATGTCACACCAACACACACTAACTATTATTAACCTCCTTCCTGTAAGCTCTAAT

ATCTCAACCTGATGAAACTTTGGTTCAATACTTATAACCTGTTTAATAATTCAAATCATA

ACCGGATTTTTTCTAGCTATTCATTACACAGCCAACATCAACATAGCATTTTCATCCATT

ATTCACATCTCACGAGATGTACCTTACGGATGAATCATACAAAACACCCACGCCATCGGA

GCCTCTCTATTCTTTATTTGTGTCTATATTCACATTGCACGCAGCCTCTATTACGGGTCT

TACTTAAATAAAGAGGTGTGAATATCAGGCATTATCCTCCTAATCACCCTTATAGCAACA

GCCTTCTTTGGTTATGTCCTACCATGAGGACAAATATCTTTTTGAGCTGCAACAGTAATC

ACCAACCTATTAACCGCAGTACCATATCTAGGGGTAACATTAACAACTTGGTTCTGGGGG

GGATTTGCAATCAACGACCCAACCCTTACTCGATTTTTTGCTCTTCACTTTATCATGCCA

TTTGGATTACTATCTTTAACCATAATTCATATTATACTTCTACACAACGAAGGCTCTAAC

AACCCACTAGGTACTAATTCAAATATCGACAAAATTCCCTTACACCCATACCACTCCTAC

AAAGACACCCTATTGGCAACCATCTTAATTACTACCCTATTTATTATTTTAACATTCTTT

CCAAACATCTTAAACGATCCAGAAAATTTCTCCAAAGCCAACCCCCTAATCACACCACAA

CACATTAAACCAGAATGATACTTTTTATTTGCTTACGGAATTCTACGATCAATCCCTAAT

AAATTAGGCGGGGCCCTAGCCTTAGTCTTATCTATTTGTATTTTAATTACCACACCATTT

ACCCACACAAGCTCAATCCGATCAATATCATTTCGTCCTATAGCACAAACAATATTTTGA

ACTCTAATTATCACCTCTATTATTATTACCTGAACTGCCACTAAAACAGTAGAACCACCA

TTTATTACTATTAGTCAAATAGCCTCATTATTATATTTCTCATACTTCATCATTAGCCCA

CTATTAGGACTGCTAGAAAACAAAATTCTAAAAACACAAACCTGCT

>MZ712233_CAS247982_[Pareas_carinatus_tenasserimicus]

TCAAAAATGTCACACCAACACATACTTACCGTTATTAATCTCCTCCCTGTAAGCTCTAAC

ATCTCAACCTGATGAAACTTCGGCTCAATACTTATAACCTGTCTAATAATTCAAATCATG

ACTGGATTCTTCCTAGCTATTCACTATACAGCCAACATCAACATAGCATTTTCATCCATT

ATTCATATCTCACGAGATGTACCATACGGATGAATCATACAAAATATTCACGCCATTGGA

GCATCTCTGTTCTTCATTTGCATTTACATTCATATTGCACGCGGTCTTTATTACGGATCT

TACTTAAATAAAGAAGTGTGAGTATCAGGCATCGTCCTCCTAATTACCCTCATAGCAACC

GCCTTTTTCGGTTACGTTCTACCATGAGGACAGATATCTTTTTGAGCCGCAACAGTAATC

ACCAACCTATTAACTGCAGTACCATACCTAGGAGCCACATTAACAACCTGATTCTGAGGG

GGGTTTGCAATTAACGACCCAACTCTTACTCGATTCTTTGCCCTTCACTTCATTCTACCA

TTCGGGCTACTCTCTTTAGCTATCATTCACATTATACTTCTACACAACGAAGGGTCTAGC

AACCCCCTAGGGACAAATTCAAATATTGACAAAATCCCACTACACCCATACCACTCTTAC

AAAGACATTTTATTAGCGACTATTCTAATTACCTCCCTATTTATTATTTTAACTTTTTTT

CCGAATACCTTAAACGACCCAGAAAACTTTTCCAAAGCCAACCCACTAGTCACACCACAA

CACATTAAACCAGAGTGGTACTTTTTATTTGCTTACGGAATTCTTCGATCAATCCCTAAT

AAGCTGGGTGGAGCCCTAGCCTTAATCTTATCCATTTGCATTTTACTTACTTCACCTTTC

ACCCACACGAGCTCAATTCGATCAATGTCCTTCCGCCCAATAGCACAAATAATATTTTGA

GCTTTAATTACCACCTCCATTATCATTACCTGAACAGCCACTAAGACAGTAGAACCACCA

TTCATTACCGTAAGCCAAATAACCTCCTTATTGTATTTCTCATACTTCATCATTAGTCCA

TTATTAGGCCTAATAGAAAATAAAATTATAAAAACTCAAATATGCT

>MZ712234_ZMMUR16800_[Pareas_carinatus_tenasserimicus]

ATGTCACACCAACACACACTACCATTATTTAACCTACTCCCTGTAAGCTCTAACATCTCA

ACCTGATGAAACTTCGGCTCAATACTTATAACCTGCCTAATAATTCAAGTCATAACTGGG

TTCTTCCTAGCTATTCATTATACAGCCAACATCAACATAGCATTTTCATCCATTATTCAT

ATTTCACGAGATGTGCCATACGGATGAATCATACAAAATATTCACGCCATCGGAGCATCT

CTATTCTTCATTTGCATTTACATTCATATTGCACGCGGTCTTTATTACGGGTCTTACTTA

AATAAAGAAGTGTGAGTTTCAGGCATCATTCTACTAATTACCCTCATAGCAACCGCCTTT

TTCGGTTATGTTCTACCATGAGGACAGATGTCTTTTTGAGCCGCAACAGTAATCACCAAC

CTACTAACTGCAGTACCGTACCTAGGAGCTACATTAACAACCTGGTTCTGAGGGGGATTT

GCAATCAACGACCCAACTCTTACTCGATTCTTTGCCCTTCACTTCATTCTACCATTCGGA

CTACTCTCTTTAGCTATTATTCATATTATACTTCTACACAACGAAGGGTCTAGCAACCCC

CTAGGGACAAATTCAAATATTGACAAAATCCCACTTCATCCATACCATTCTTACAAAGAC

ATTTTATTAGCGACTATTCTAATTACCACCCTATTTATTATTTTAACTTTTTTTCCAAAT

ACCTTAAACGACCCAGAAAATTTTTCCAAAGCCAACCCACTAGTCACACCACAACACATT

AAACCAGAATGGTACTTTTTATTTGCTTACGGAATCCTTCGGTCAATCCCTAATAAGCTA

GGAGGGGCCCTAGCCCTAATCTTATCCATTTGCATTTTACTTACTACACCTTTCACCCAC

ACAAGCTCAATCCGATCAATATCCTTCCGCCCAATAGCACAAATAGTGTTTTGAACTCTA

ATTACTACTTCCATTATCATTACCTGAACAGCCACTAAAACAGTAGAACCCCCATTCATT

ACCGTAAGCCAAATAGCCTCCTTATTGTATTTCTCATACTTCATCATTAGTCCCATACTA

GGCCTAATGGAAAATAAAATTATAAAAACCCCTAACT

>MZ712235_ZMMUR16393_[Pareas_abros]

ACAAAAATGCCTCACCAACACGCACTTATACTAACCAACCTCCTTCCTGTAAGCACTAAC

ATCTCAACGTGGTGAAACTTTGGCTCAATACTTATAACCTGCTTAGCAGTTCAAACTATA

ACCGGATTCTTCCTGGCCATCCACTACACAGCTGACATTAACATAGCTTTTTCATCCGTC

ATCCATATCTCACGAGATGTACCTTGTGGATGAATTATTCAAAACACACACGCAATTGGA

GCCTCCTTATTCTTTATCTGCATCTATATCCATATCGCACGAGGTCTTTATTACGGGTCC

TATATAAATAAAGGAGTCTGAACTTCTGGTGTCACTTTATTAATCACCCTAATAGCAACA

GCCTTCTTTGGTTATGTGCTGCCTTGAGGACAAATATCCTTTTGAGCCGCAACAGTAATC

ACAAACCTACTAACTGCAGTACCCTACCTGGGCAGCACACTGACAAGTTGGTTTTGAGGG

GGATTTGCAATCAATAATCCTACACTTACCCGATTTTTTGCCCTCCACTTTAATCTCCCT

TTTAAATTAATCTCCATATCCTTAATCCAGATTATACTACTACACAATGAAGGTTCCAGT

AATCCACTAGGAACAAATTCAGATATTGACAAAATCCCGCTCCATCCATACCACTCTCAC

AAAGACCTCCTCGTAATAACTATCCTTATTACCATCTTATTTACCATTATAACCTTCCTC

CCAAACATTTTCAACGACCCAGAAAACTTCTCCAAAGCCAACCCAATAATTACACCACAA

CACATTAAACCAGAATGATATTTCCTATTTGCCTACGGCGTCCTTCGAACTGTCCCAAAT

AAATTGGGGGGTACTTTAGCCTTGGTGTTATCCATCTGTGTCCTATTCATTATGCCATTT

ACCCACACAAGCTCAATCCGATCAATATATTTCCGTCCTATAGCACAAACAACATTCTGA

GCCCTAATCGTCACCTCCATCACCCTTACCTGAACAGCTACTAAAACAGTAGAACCACCA

TTTACAACCATTGGTCAAGCAGCCTCATTCCTATATTTCTCCTGCTTCACAGTCAGTCCA

ATACTGGGCTGACTGGAAAACAAAATCATAAAAACCTCAAACTGCT

>MZ712236_ZMMUR16392_[Pareas_abros]

ACAAAAATGCCACACCAACACGCACTTATACTAACCAACCTCCTTCCTGTAAGCACTAAC

ATCTCAACGTGGTGAAACTTTGGCTCAATACTTATAACCTGCTTAGCAGTTCAAACTATA

ACCGGATTCTTCCTGGCCATCCACTACACAGCTGACATTAACATAGCTTTTTCATCCGTC

ATCCATATCTCGCGAGATGTGCCTTGTGGGTGAATTATTCAAAACACACACGCAATTGGA

GCCTCCTTATTCTTTATCTGCATCTATATCCATATCGCACGAGGTCTTTATTACGGGTCC

TATATAAATAAAGGAGTCTGAACTTCTGGTGTCACTTTATTAATCACCCTAATAGCAACA

GCCTTCTTTGGTTATGTGCTGCCTTGAGGACAAATATCCTTTTGAGCCGCAACAGTAATC

ACAAACCTACTAACTGCAGTACCCTACCTGGGCAGCACACTGACAAGTTGGTTTTGAGGA

GGATTTGCAATCAATAATCCTACACTTACCCGATTTTTTGCCCTCCACTTTATTCTCCCT

TTTATATTAATCTCCATATCCTTAATCCACATTATACTACTACACAATGAAGGTTCCAGT

AATCCACTAGGAACAAATTCAGATATTGACAAAATCCCGCTCCATCCATACCACTCTCAC

AAAGACCTCCTCGTAATAACTATCCTTATTACCATCTTATTTACCATTATAACCTTCCTC

CCAAACATTTTCAACGACCCAGAAAACTTCTCCAAAGCCAACCCAATAATTACACCACAA

CACATTAAACCAGAATGATATTTCCTATTTGCCTACGGCGTCCTTCGAACTGTCCCAAAT

AAATTGGGGGGGTGCTTAGCCTTGGTGTTATCCATCTGTATCCTATTCATTATGCCATTT

ACCCACACAAGCTCAATCCGATCAATATATTTCCGTCCTATAGCACAAACAACATTCTGA

GCCCTAATCGTCACCTCCATCACCCTTACCTGTACAGCTACAAAAACAGTAGAACCACCA

TTGACAACCATTGGTCAAGCAGCCTCATTCCTATATTTCTCATGCTTCACAGTCAGTCCA

ATACTGGGCTGACTGGAAAACAAAATCATAAAAACCTCAAACTGCT

>MZ712237_ZMMUR14788_[Pareas_abros]

ACAAAAATGCCACACCAACACGCACTTATACTAACCAACCTCCTTCCTGTAAGCACTAAC

ATCTCAACGTGGTGAAACTTTGGCTCAATACTTATAACCTGCTTAGCAGTTCAAACTATA

ACCGGATTCTTCCTGGCCATCCACTACACAGCTGACATTAACATAGCTTTTTCATCCGTC

ATCCATATCTCGCGAGATGTGCCTTGTGGGTGAATTATTCAAAACACACACGCAATTGGA

GCCTCCTTATTCTTTATCTGCATCTATATCCATATCGCACGAGGTCTTTATTACGGGTCC

TATATAAATAAAGGAGTCTGAACTTCTGGTGTCACTTTATTAATCACCCTAATAGCAACA

GCCTTCTTTGGTTATGTGCTGCCTTGAGGACAAATATCCTTTTGAGCCGCAACAGTAATC

ACAAACCTACTAACTGCAGTACCCTACCTGGGCAGCACACTGACAAGTTGGTTTTGAGGA

GGATTTGCAATCAATAATCCTACACTTACCCGATTTTTTGCCCTCCACTTTATTCTCCCT

TTTATATTAATCTCCATATCCTTAATCCACATTATACTACTACACAATGAAGGTTCCAGT

AATCCACTAGGAACAAATTCAGATATTGACAAAATCCCGCTCCATCCATACCACTCTCAC

AAAGACCTCCTCGTAATAACTATCCTTATTACCATCTTATTTACCATTATAACCTTCCTC

CCAAACATTTTCAACGACCCAGAAAACTTCTCCAAAGCCAACCCAATAATTACACCACAA

CACATTAAACCAGAATGATATTTCCTATTTGCCTACGGCGTCCTTCGAACTGTCCCAAAT

AAATTGGGGGGGTGCTTAGCCTTGGTGTTATCCATCTGTATCCTATTCATTATGCCATTT

ACCCACACAAGCTCAATCCGATCAATATATTTCCGTCCTATAGCACAAACAACATTCTGA

GCCCTAATCGTCACCTCCATCACCCTTACCTGTACAGCTACAAAAACAGTAGAACCACCA

TTGACAACCATTGGTCAAGCAGCCTCATTCCTATATTTCTCATGCTTCACAGTCAGTCCA

ATACTGGGCTGACTGGAAAACAAAATCATAAAAACCTCAAACTGCT

>MZ712238_ZMMUR13656_[Pareas_temporalis]

TCTCAAATGCTACACCAACACACACTTATGTTAACCAACCTCCTTCCTGTAAGCACCAAT

ATTTCAACATGGTGAAACTTCGGCTCAATACTTATAACATGCTTAGCGATTCAAACTGTA

ACTGGATTTTTCCTAGCCATCCACTACACAGCTGACATCAACACAGCTTTTTCATCCATC

ATTCATATTTCACGAGATGTACCCTACGGATGAGTTATACAAAATACACACGCAATCGGA

GCATCCCTGTTCTTCATCTGTATCTACATTCACATTGCACGAGGCCTTTATTATGGCTCC

TACATAAATAAAGAAGTGTGAGCTTCAGGTATCACTTTATTAATTATCGTGATAGCAACA

GCCTTCTTTGGTTATGTTCTACCGTGAGGACAAATATCATTTTGAGCCGCAACAGTAATT

ACAAACCTATTAACTGCAGTACCCTACCTGGGCAGCACATTAACAACTTGATTTTGGGGG

GGGTTTGCAATCAACAACCCCACACTTACCCGATTTTTTGCACTTCACTTCATTCTCCCT

TTTATTTTAATCTCCTTATCTCTAATCCATATCATACTTCTACACAATGAAGGCTCCAGT

AATCCACTAGGAACAAATTCAGATGTCGACAAAATCCCACTACATCCATATCACTCTTAC

AAAGACCTCCTCCTAATAACCATCCTTATTACCACTTTATTTATCATTATAACCTTTCTC

CCAAACATTTTAAACGACCCAGAAAACTTCTCCAAAGCCAACCCACTAGTTACACCACAA

CACATTAAACCAGAGTGGTATTTCCTATTTGCGTACAGCGTTCTCCGAACAATCCCAAAT

AAATTAGGGGGGGCTTTAGCCTTAGTGTTATCCATCAGTATCCTATTCATTATACCATTC

ATTCATACGAGCTCAGTTCGATCAATATCTTTCCGCCCAATAGCACAAATAACATTTTGA

ACCCTAATCGTCACTTCCATTATCCTTACCTGAACAGCCACTAAAACAGTAGAACCACCA

TTTACAACCATTGGTCAAACAGCCTCGCTCCTATACTTTTCTTACTTCATAATCAGTCCA

GTGTTGGGTTGATTAGAGAACAAAATCATAAAAACTCAAACGTGCT

>MZ712239_SIEZC20215_[Pareas_temporalis]

TCTCAAATGCTACACCAACACACACTTATGTTAACCAACCTCCTTCCTGTAAGCACCAAT

ATTTCAACATGGTGAAACTTCGGCTCAATACTTATAACATGCTTAGCGATTCAAACTGTA

ACTGGATTTTTCCTAGCCATCCACTACACAGCTGACATCAACACAGCTTTTTCATCCGTC

ATTCATATTTCACGAGATGTACCCTACGGATGAGTTATACAAAATACACACGCAATCGGA

GCATCCCTGTTCTTCATCTGTATCTACATTCACATTGCACGAGGCCTTTATTATGGCTCC

TACATAAATAAAGAAGTGTGAGCTTCAGGTATCACTTTATTAATTATCCTGATAGCAACA

GCCTTCTTTGGTTATGTTCTACCCTGAGGACAAATATCATTTTGAGCCGCAACAGTAATT

ACAAACCTATTAACTGCAGTACCCTACCTGGGCAGCACATTAACAACTTGATTTTGGGGG

GGGTTTGCAATCAACAACCCCACACTTACCCGATTTTTTGCACTTCACTTCATTCTCCCT

TTTATCTTAATCTCCTTATCTCTAATCCATATCATACTTCTACACAATGAAGGCTCCAGT

AATCCACTAGGAACAAATTCAGATGTCGACAAAATCCCACTACATCCATATCACTCTTAC

AAAGACCTCCTCCTAATAACCATCCTTATTACCACTTTATTTATCATTATAACCTTTCTC

CCAAACATTTTAAACGACCCAGAAAACTTCTCCAAAGCCAACCCACTAGTTACACCACAA

CACATTAAACCAGAGTGGTATTTCCTATTTGCCTACAGCGTTCTCCGAACAATCCCAAAT

AAATTAGGGGGGGCTTTAGCCTTAGTGTTATCCATCAGTATCCTATTCATTATACCATTC

ATTCATACGAGCTCAGTTCGATCAATATCTTTCCGCCCAATAGCACAAATAACATTTTGA

ACCCTAATCGTCACTTCCATTATCCTTACCTGAACAGCCACTAAAACAGTAGAACCACTA

TTTACAACCATTGGTCAAACAGCCTCGCTCCTATACTTTTCTTACTTCATAATCAGTCCA

GTGTTGGGTTGATTAGAGAACAAAATCATAAAAACTCAAACGTGCT

***ND4* mtDNA gene**

>MZ712240_NMNH05625_[Pareas_komaii]

GCAATCCTTTTAAAACTGGGCGGATATGGTATAATCCGAATAATACAAGTTATACCAACC

ATAAAAACAGACGTGTTTCTCCCATTCTTAGTTCTAGCGCTATGAGGCGCAATTCTAGCC

AACCTGACCTGTTTACAACAAACGGATTTGAAGTCTCTAATCGCATACTCCTCTGTTAGC

CATATGGGTTTAGTAATCGCATCAATCACACTACAAACTCAATGGGGTCTTTCCGGGGCC

ATATCACTAATAATTGCCCATGGATTCACCTCCTCAACACTATTTTGTCTGGCCAACACC

ACCTATGAGCGAACAAACACCCGTATTTTGATCCTAACCCGTGGGTTCCACAACATCTTA

CCAATAACTACAACCTGGTGATTAGTGGCTAATCTTATAAACATAGCTATTCCACCCAGC

CTTAACTTCACGGGGGAGTTACTAATCGCATCCTCTATGTTCAATTGGTGTCCAACAACA

ATTATTATATTTGGACTCTCCGTACTAATTACCGCCTTATATTCCCTACACATATTCCTA

TCTACCCAAATAGGCACCCCCCTATTAAACCTCCCCACGCAACCAACACACTCACGAGAG

CACCTACTCATGATTCTCCACACCACGCCACTTATTATAATCTCAATGAAGCCAGAACTC

GTA

>MZ712241_ZMMUR16628_[Pareas_macularius]

CTAGCAGCAATCCTCCTAAAACTCGGGGGATACGGTTTAATCCGAATAACACAAATCCTA

CCTACTATAAAAACTGACACCTTCCTCCCGTTCCTAATTCTCGCCCTCTGAGGGGCCATC

TTAGCTAACCTGACATGCCTTCAACAAACCGACTTAAAATCCTTAATCGCATACTCGTCC

ATTAGCCACATGGGCCTGGTAATCGCATCAATCACAATCCAAACCCCCTGGGGCTTATCC

GGGGCCATGTCTTTAATAATCGCCCACGGCTTTACCTCCTCTGCCCTATTCTGTCTAGCT

AACACCACTTACGAACGAACAAACACCCGCATCTTAGCCCTAACCCGTGGATTCCACAAC

ATCCTACCAATAGCTACAACCTGATGACTACTAATCAACCTTATAAACATCGCCATCCCT

CCTAGCCTGAACTTTACAGGAGAACTATTAATTGCTTCCTCTCTATTCAACTGATCCCCA

ATAACAATTATTATATTCGGACTTTCTATACTAATCACCGCCTCTTATTCTCTTCACGTA

TTCCTATCAACCCAAATAGGCACACCCCTATTAAACCTACCGACACAACCAACACACTCA

CGAGAGCACCTACTCATAATTCTCCACATTCTCCCACTTATTATAGTATCACTAAAGCCG

GAACTAGTACTC

>MZ712242_CIB098271_[Pareas_margaritophorus]

TTAGCAGCAATCCTGGTAAAACTAGGGGGGTACGGCATAATCCGAATAATACAAATTCTC

CCAACTACAAAAACCGATACCTTCCTACCCCTCCTAATTCTCGCCCTTTGGGGGGCCATC

TTGTCCAACCTCACCTGCCTACAACAAACAGATCTAAAGTCCTTAATCGCATACTCCTCT

ATCAGCCACATGGGCCTAGTAATTGCATCAATCACGCTCCAAACCCAATGAGGCCTGTCC

GGAGCCATATCTCTAATAATCGCCCACGGCTTTACCTCCTCAGCCTTATTTTGTTTAGCC

AACACCACCTACGAACGAACAAACACCCGAATCTTAGCCCTAACACGAGGATTCCACAAC

ATCCTGCCAATAGCTACAACTTGATGACTGCTCGCTAACCTTATAAACATCGCCATCCCC

CCAAGCCTAAATTTCACAGGAGAACTACTGATTGCCTCCTCCCTATTCAATTGGTGTCCA

ACAACAATTATTATATTCGGACTATCTATATTAATCACAGCCTCCTATTCCCTACACATA

TTTATATCGACCCAAATAGGCACACCCCTGTTAAACCTGCCCACACAACCAACCCACTCA

CGAGAACACCTACTTATAATACTACACATCCTACCACTTATAATAATCTCACTAAAACCA

GGACTTGTACTC

>MZ712243_ZMMUNAP09759_[Pareas_margaritophorus]

TTAGCAGCAGTTCTACTAAAACTGGGGGGGTACGGCTTAATCCGGATAATACAAATCCTC

CCAACCACAAAAACCGATACCTTTCTACCCCTCCTGGTTTTAGCCCTTTGGGGGGCCATC

TTATCCAATCTAACCTGCCTACAACAAACAGATCTAAAGTCCTTAATCGCATACTCCTCT

ATCAGTCACATGGGCCTAGTAATCGCATCAATCACACTCCAAACCCAATGAGGCCTATCT

GGAGCCATGTATTTAATAATCGCCCACGGCTTTACCTCCTCAGCCTTATTTTGTCTAGCC

AACACCACCTACGAACGAACAAACACCCGAATCCTAGCCCTAACACGAGGGTTCCACAAC

ATCCTACCAATAGCTACAACCTGATGACTATTTACTAACCTCATAAACATCGCCATCCCT

CCAAGCCTAAATTTCACAGGAGAACTACTAATTGCCTCCTCCCTATTCAACTGGTGTCCA

ACAACAATTATTATATTCGGACTATCTATGTTAATCACAGCCTCCTATTCCCTACACATA

TTTATATCAACCCAAATAGGCACACCAGTGTTAAACCTACCCACACAACCAACCCACTCA

CGAGAACACCTACTTATAATCCTACACATCCTGCCACTTATAATAGTCTCACTAAAACCA

GGACTAGTACTC

>MZ712244_AUP01573_[Pareas_berdmorei_berdmorei]

TTAGCAGCAATTCTTCTAAAGCTGGGGGGGTATGGACTAATACGAATAATTCAAATCATA

CCACCAATAAAAACTAATTTATTCCTACCATTCCTTATTCTAGCCCTATGGGGGGCAGTT

TTAGCCAATCTAACATGTTTACAACAAACAGACCTGAAATCTCTAATTGCATACTCATCT

ATCAGTCACATAGGATTAGTAATCGCATCAATTCTACTACAAACCCAGTGAGGTTTATCA

GGAGCTATATCTTTAATAATTGCCCACGGTTTCACATCATCAATATTATTCTGTCTCGCT

AATACAACCTATGAACGAACAAACACCCGCATCCTAATCTTAACACGAGGGTTTCACAAT

ATCCTACCAATAACTACAGCCTGATGACTTATAGCCAACTTAATAAATATTGCTATTCCA

CCAAGCCTAAACTTTACAGGAGAACTACTAATCGCATCATCTTTATTCAACTGATGCCCA

ACTACAATAATCTTATTTGGTATATCTATGCTAATTACCATTTCATATTCTCTACAAGTA

TTTTTATCTACTCAGATGGGTACATCCCTATTAAATACACCTACACAGCCAACACACTCA

CGAGAACACCTACTAATAATACTACACACCCTCCCTCTTATTATAATTTCAATAAAACCA

GAACTTGTATTT

>MZ712245_CAS240362_[Pareas_berdmorei_berdmorei]

TTAGCAGCAATTCTTCTAAAGTTGGGGGGGTACGGACTAATACGAATAATTCAAATCATA

CCACCAACAAAAACAAATTTATTCCTACCATTCCTTATTCTAGCCCTATGAGGGGCAGTT

TTAGCCAATCTAACATGTCTACAACAAACAGACCTGAAATCTCTAATTGCATACTCATCT

ATTAGTCACATAGGATTAGTAATCGCATCAATTCTACTACAAACCCAGTGAGGTTTATCA

GGAGCTATATCTTTAATAATTGCCCACGGTTTCACATCATCAATATTATTCTGTCTCGCT

AATACAACCTATGAACGAACAAACACCCGCATCCTAATCTTAACACGAGGGTTTCACAAT

ATCCTACCAATAACTACAGCCTGATGACTTATAGCCAACTTAATAAATATTGCTATTCCA

CCAAGCCTAAACTTTACAGGAGAACTACTAATTGCATCATCTTTATTCAACTGATGCCCA

ACTACAATAATCTTATTTGGTATATCTATGCTAATTACCATTTCATATTCTCTACAAGTA

TTTTTATCTACTCAGATAGGTACACCCCTATTAAATACACCTACACAGCCAACACACTCA

CGAGAACACCTACTAATAATACTACACATCCTCCCTCTTATTATAATTTCAATAAAACCA

GAACTTGTATTT

>MZ712246_ZMMUR16803_[Pareas_berdmorei_berdmorei]

TTAGCAGCAATTCTTCTAAAGTTGGGGGGGTACGGACTAATACGAATAATTCAAATCATA

CCACCAACAAAAACAAATTTATTCCTACCATTCCTTATTCTAGCCCTATGAGGGGCAGTT

TTAGCCAATCTAACATGTCTACAACAAACAGACCTGAAATCTCTAATTGCATACTCATCT

ATTAGTCACATAGGATTAGTAATCGCATCAATTCTACTACAAACCCAGTGAGGTTTATCA

GGAGCTATATCTTTAATAATTGCCCACGGTTTCACATCATCAATATTATTCTGTCTCGCT

AATACAACCTATGAACGAACAAACACCCGCATCCTAATCTTAACACGAGGGTTTCACAAT

ATCCTACCAATAACTACAGCCTGATGACTTATAGCCAACTTAATAAATATTGCTATTCCA

CCAAGCCTAAACTTTACAGGAGAACTACTAATTGCATCATCTTTATTCAACTGATGCCCA

ACTACAATAATCTTATTTGGTATATCTATGCTAATTACCATTTCATATTCTCTACAAGTA

TTTTTATCTACTCAGATAGGTACACCCCTATTAAATACACCTACACAGCCAACACACTCA

CGAGAACACCTACTAATAATACTACACATCCTCCCTCTTATTATAATTTCAATAAAACCA

GAACTTGTATTT

>MZ712247_ZMMUR16801_[Pareas_berdmorei_truongsonicus]

TTAGCAGCAATCCTTCTAAAACTTGGAGGATACGGACTAATACGAATAATTCAAATCATA

CCACCAACAAAAACAAATTTATTCCTCCCATTCCTTATTCTAGCCCTGTGGGGGGCTGTT

TTAGCCAATCTAACATGTTTACAACAAACAGACCTAAAATCTCTAATTGCATACTCATCT

ATCAGCCACATAGGATTAGTAATCGCATCAATTCTACTACAAACCAAATGAGGCTTATCT

GGGGCTATATCTTTAATAATTGCCCATGGATTCACATCGTCAATGTTATTCTGTCTCGCT

AATACAACCTATGAACGAACAAACACCCGCATTCTAATCTTAACACGAGGATTTCACAAT

ATCCTGCCAATAACTACAGCCTGATGACTTATAGCCAACTTAATAAATATTGCCATTCCA

CCAAGCCTAAACTTTACAGGAGAACTACTAATTGCATCATCTTTATTTAACTGATGCCCA

ACTACAATAATCCTACTTGGCATGTCTATGCTAATTACCATTTCATACTCCCTACAAGTA

TTTATATCTACCCAAATAGGTACATCACTATTAAATGCACCTACACAGCCAACACACTCA

CGAGAACACCTATTAATAACCCTGCACATCCTTCCTCTTATTATAATTTCAATAAAACCA
GAACTTGTATTC
>MZ712248_ZMMUR14796_[Pareas_berdmorei_truongsonicus]
TTAGCAGCAATCCTTCTAAAACTTGGAGGATACGGACTAATACGAATAATTCAAATCATA
CCACCAACAAAAACAAATTTATTCCTCCCATTCCTTATTCTAGCCCTGTGGGGGGCTGTT
TTAGCCAATCTAACATGTTTACAACAAACAGACCTGAAATCTCTAATTGCATACTCATCT
ATCAGCCACATAGGATTAGTAATCGCATCAATTCTACTACAAACCAAATGAGGCTTATCT
GGGGCTATATCTTTAATAATTGCCCATGGATTCACATCGTCAATGTTATTCTGTCTCGCT
AATACAACCTATGAACGAACAAACACCCGCATTCTAATCTTAACACGAGGATTTCACAAT
ATCCTGCCAATAACTACAGCCTGATGACTTATAGCCAACTTAATAAATATTGCCATTCCA
CCAAGCCTAAACTTTACAGGAGAACTACTAATTGCATCATCTTTATTTAACTGATGCCCA
ACTACAATAATCCTACTTGGCATGTCTATGCTAATTACCATTTCATACTCCCTACAAGTA
TTTATATCTACCCAAATAGGTACATCACTATTAAATGCACCTACACAGCCAACACACTCA
CGAGAACACCTATTAATAACCCTGCACATCCTTCCTCTTATTATAATTTCAATAAAACCA
GAACTTGTATTC
>MZ712249_ZMMUR137531_[Pareas_berdmorei_unicolor]
CTAGCAGCAATTCTTCTAAAACTTGGGGGGTACGGCCTAATACGAATAATTCAAATCATA
CCACCAACAAAAACAAATTTATTCCTGCCATTCCTCATTCTAGCCCTGTGGGGAGCAATT
TTAGCCAATCTAACATGTTTACAACAAACAGACCTGAAATCTCTAATTGCATACTCATCT
ATCAGCCACATAGGGTTAGTAATCGCATCAATTCTACTACAAACCCAGTGAGGCTTATCT
GGGGCAATATCCTTAATAATCGCCCATGGATTCACATCGTCAATACTATTCTGTCTCGCT
AATACAACCTATGAACGAACAAACACCCGCATTCTAATCTTAACACGAGGGTTTCACAAC
ATCCTACCAATAACCACAGCCTGATGACTTATGGCCAACTTAATAAATATTGCCATTCCA
CCAAGCCTAAACTTTACAGGAGAACTACTAATTGCATCATCTTTATTCAACTGATGCCCA
ACTACAATAGTCTTATTTGGCATGTCTATACTAATTACCATTTCATACTCTCTACAAGTA
TTTATATCTACCCAGATAGGTACGTCACTATTAGATGCACCTACACAGCCAACACACTCA
CGAGAACACCTACTAATAACTCTACACATCCTTCCCCTTATTATAATTTCAATAAAACCA
GAACTTGTATTC
>MZ712250_ZMMUR137532_[Pareas_berdmorei_unicolor]

CTAGCAGCAATTCTTCTAAAACTTGGGGGGTACGGCCTAATACGAATAATTCAAATCATA
CCACCAACAAAAACAAATTTATTCCTGCCATTCCTCATTCTAGCCCTGTGGGGAGCAATT
TTAGCCAATCTAACATGTTTACAACAAACAGACCTGAAATCTCTAATTGCATACTCATCT
ATCAGCCACATAGGATTAGTAATCGCATCAATTCTACTACAAACCCAGTGAGGCTTATCT
GGGGCAATATCCTTAATAATCGCCCATGGATTCACATCGTCAATACTATTCTGTCTCGCT
AATACAACCTATGAACGAACAAACACCCGCATTCTAATCTTAACACGAGGGTTTCACAAC
ATCCTACCAATAACCACAGCCTGATGACTTATGGCCAACTTAATAAATATTGCCATTCCA
CCAAGCCTAAACTTTACAGGAGAACTACTAATTGCATCATCTTTATTCAACTGATGCCCA
ACTACAATAGTCTTATTTGGCATGTCTATACTAATTACCATTTCATACTCTCTACAAGTA
TTTATATCTACCCAGATAGGTACGTCACTATTAGATGCACCTACACAGCCAACACACTCA
CGAGAACACCTACTAATAACTCTACACATCCTTCCTCTTATTATAATTTCAATAAAACCA
GAACTTGTATTC
>MZ712251_ZMMUR14013_[Pareas_berdmorei_unicolor]
CTAGCAGCAATTCTTCTAAAACTTGGGGGGTACGGCCTAATACGAATAATTCAAATCATA
CCACCAACAAAAACAAATTTATTCCTGCCATTCCTCATTCTAGCCCTGTGGGGAGCAATT
TTAGCCAATCTAACATGTTTACAACAAACAGACCTGAAATCTCTAATTGCATACTCATCT
ATCAGCCACATAGGATTAGTAATCGCATCAATTCTACTACAAACCCAGTGAGGCTTATCT
GGGGCAATATCCTTAATAATCGCCCATGGATTCACATCGTCAATACTATTCTGTCTCGCT
AATACAACCTATGAACGAACAAACACCCGCATTCTAATCTTAACACGAGGGTTTCACAAC
ATCCTACCAATAACCACAGCCTGATGACTTATGGCCAACTTAATAAATATTGCCATTCCA
CCAAGCCTAAACTTTACAGGAGAACTACTAATTGCATCATCTTTATTCAACTGATGCCCA
ACTACAATAGTCTTATTTGGCATGTCTATACTAATTACCATTTCATACTCTCTACAAGTA
TTTATATCTACCCAGATAGGTACGTCACTATTAGATGCACCTACACAGCCAACACACTCA
CGAGAACACCTACTAATAACTCTACACATCCTTCCTCTTATTATAATTTCAATAAAACCA
GAACTTGTATTC
>MZ712252_ZMMUR14263_[Pareas_berdmorei_unicolor]
CTAGCAGCAATTCTTCTAAAACTTGGGGGGTACGGCCTAATACGAATAATTCAAATCATA
CCACCAACAAAAACAAATTTATTCCTGCCATTCCTCATTCTAGCCCTGTGGGGAGCAATT
TTAGCCAATCTAACATGTTTACAACAAACAGACCTGAAATCTCTAATTGCATACTCATCT

ATCAGCCACATAGGATTAGTAATCGCATCAATTCTACTACAAACCCAGTGAGGCTTATCT

GGGGCAATATCCTTAATAATCGCCCATGGATTCACATCGTCAATACTATTCTGTCTCGCT

AATACAACCTATGAACGAACAAACACCCGCATTCTAATCTTAACACGAGGGTTTCACAAC

ATCCTACCAATAACCACAGCCTGATGACTTATGGCCAACTTAATAAATATTGCCATTCCA

CCAAGCCTAAACTTTACAGGAGAACTACTAATTGCATCATCTTTATTCAACTGATGCCCA

ACTACAATAGTCTTATTTGGCATGTCTATACTAATTACCATTTCATACTCTCTACAAGTA

TTTATATCTACCCAGATAGGTACGTCACTATTAGATGCACCTACACAGCCAACACACTCA

CGAGAACACCTACTAATAACTCTACACATCCTTCCTCTTATTATAATTTCAATAAAACCA

GAACTTGTATTC

>MZ712253_ZMMUR14421_[Pareas_berdmorei_unicolor]

CTAGCAGCAATTCTTCTAAAACTTGGGGGGTACGGCCTAATACGAATAATTCAAATCATA

CCACCAACAAAAACAAATTTATTCCTGCCATTCCTCATTCTAGCCCTGTGGGGAGCAATT

TTAGCCAATCTAACATGTTTACAACAAACAGACCTGAAATCTCTAATTGCATACTCATCT

ATCAGCCACATAGGATTAGTAATCGCATCAATTCTACTACAAACCCAGTGAGGCTTATCT

GGGGCAATATCCTTAATAATCGCCCATGGATTCACATCGTCAATACTATTCTGTCTCGCT

AATACAACCTATGAACGAACAAACACCCGCATTCTAATCTTAACACGAGGGTTTCACAAC

ATCCTACCAATAACCACAGCCTGATGACTTATGGCCAACTTAATAAATATTGCCATTCCA

CCAAGCCTAAACTTTACAGGAGAACTACTAATTGCATCATCTTTATTCAACTGATGCCCA

ACTACAATAGTCTTATTTGGCATGTCTATACTAATTACCATTTCATACTCTCTACAAGTA

TTTATATCTACCCAGATAGGTACGTCACTATTAGATGCACCTACACAGCCAACACACTCA

CGAGAACACCTACTAATAACTCTACACATCCTTCCTCTTATTATAATTTCAATAAAACCA

GAACTTGTATTC

>MZ712254_SIEZC20216_[Pareas_berdmorei_unicolor]

CTAGCAGCAATTCTTCTAAAACTTGGGGGGTACGGCCTAATACGAATAAATCAAATCATA

CCACCAACAAAAACAAATTTATTCCTGCCATTCCTCATTCTAGCCCTGTGGGGAGCAATT

TTAGCCAATCTAACATGTTTACAACAAACAGACCTGAAATCTCTAATTGCATACTCATCT

ATCAGCCACATAGGATTAGTAATCGCATCAATTCTACTACAAACCCAGTGAGGCTTATCT

GGGGCAATATCCTTAATAATCGCCCATGGATTCACATCGTCAATACTATTCTGTCTCGCT

AATACAACCTATGAACGAACAAACACCCGCATTCTAATCTTAACACGAGGGTTTCACAAC

ATCCTACCAATAACCACAGCCTGATGACTTATGGCCAACTTAATAAATATTGCCATTCCA
CCAAGCCTAAACTTTACAGGAGAACTACTAATTGCATCATCTTTATTCAACTGATGCCCA
ACTACAATAGTCTTATTTGGCATGTCTATACTAATTACCATTTCATACTCTCTACAAGTA
TTTATATCTACCCAGATAGGTACGTCACTATTAGATGCACCTACACAGCCAACACACTCA
CGAGAACACCTACTAATAACTCTACACATCCTTCCTCTTATTATAATTTCAATAAAACCA
GAACTTGTATTC
>MZ712255_ZMMUR136791_[Pareas_berdmorei_unicolor]
CTAGCAGCAATTCTTCTAAAACTTGGGGGGTACGGCCTAATACGAATAATTCAAATCATA
CCACCAACAAAAACAAATTTATTCCTGCCATTCCTCATTCTAGCCCTGTGGGGAGCAATT
TTAGCCAATCTAACATGTTTACAACAAACAGACCTGAAATCTCTAATTGCATACTCATCT
ATCAGCCACATAGGATTAGTAATCGCATCAATTCTACTACAAACCCAGTGAGGCTTATCT
GGGGCAATATCCTTAATAATCGCCCATGGATTCACATCGTCAATACTATTCTGTCTCGCT
AATACAACCTATGAACGAACAAACACCCGCATTCTAATCTTAACACGAGGGTTTCACAAC
ATCCTACCAATAACCACAGCCTGATGACTTATGGCCAACTTAATAAATATTGCCATTCCA
CCAAGCCTAAACTTTACAGGAGAACTACTAATTGCATCATCTTTATTCAACTGATGCCCA
ACTACAATAGTCTTATTTGGCATGTCTATACTAATTACCATTTCATACTCTCTACAAGTA
TTTATATCTACCCAGATAGGTACGTCACTATTAGATGCACCTACACAGCCAACACACTCA
CGAGAACACCTACTAATAACTCTACACATCCTTCCTCTTATTATAATTTCAATAAAACCA
GAACTTGTATTC
>MZ712256_ZMMUR136792_[Pareas_berdmorei_unicolor]
CTAGCAGCAATTCTTCTAAAACTTGGGGGGTACGGCCTAATACGAATAATTCAAATCATA
CCACCAACAAAAACAAATTTATTCCTGCCATTCCTCATTCTAGCCCTGTGGGGAGCAATT
TTAGCCAATCTAACATGTTTACAACAAACAGACCTGAAATCTCTAATTGCATACTCATCT
ATCAGCCACATAGGATTAGTAATCGCATCAATTCTACTACAAACCCAGTGAGGCTTATCT
GGGGCAATATCCTTAATAATCGCCCATGGATTCACATCGTCAATACTATTCTGTCTCGCT
AATACAACCTATGAACGAACAAACACCCGCATTCTAATCTTAACACGAGGGTTTCACAAC
ATCCTACCAATAACCACAGCCTGATGACTTATGGCCAACTTAATAAATATTGCCATTCCA
CCAAGCCTAAACTTTACAGGAGAACTACTAATTGCATCATCTTTATTCAACTGATGCCCA
ACTACAATAGTCTTATTTGGCATGTCTATACTAATTACCATTTCATACTCTCTACAAGTA

TTTATATCTACCCAGATAGGTACGTCACTATTAGATGCACCTACACAGCCAACACACTCA

CGAGAACACCTACTAATAACTCTACACATCCTTCCTCTTATTATAATTTCAATAAAACCA

GAACTTGTATTC

>MZ712257_ZMMUR14165_[Pareas_berdmorei_unicolor]

TTAGCAGCAATTCTTCTAAAACTTGGGGGGTACGGCCTAATACGAATAATTCAAATCATA

CCACCAACAAAAACAAACTTATTCCTACCATTCCTTATTCTAGCCCTATGGGGAGCAATT

TTAGCCAATCTAACATGTTTACAACAAACAGACCTGAAATCTCTAATTGCATACTCATCT

ATCAGCCACATAGGATTAGTAATCGCATCAATTCTACTACAAACCCAGTGAGGCTTATCT

GGAGCAATATCCTTAATAATCGCCCATGGATTCACATCGTCAATACTATTCTGTCTCGCT

AATACAACCTATGAACGAACAAACACCCGCATTCTAGTCTTAACACGGGGGTTTCACAAC

ATCCTACCAATAACCACAGCCTGATGACTTATGGCCAACTTAATAAATATTGCCATTCCA

CCAAGCCTAAACTTTACAGGAGAACTACTAATTGCATCATCTTTATTCAACTGATGCCCA

ACTACAATAGTCTTATTTGGCATGTCCATACTAATTACCATTTCATACTCTCTACAAGTA

TTTATATCTACCCAGATAGGTACATCACTATTAGATGCACCTACACAGCCAACACACTCA

CGAGAACACCTACTAATAACCCTACACATCCTTCCTCTTATTATAATTTCAATAAAACCA

GAACTTGTACTC

>MZ712258_ZMMUR16802_[Pareas_kuznetsovorum]

GTACTAGCAGCAATTCTCCTAAAACTTGGCGGATATGGTCTAATCCGAATAATTCAAATC

ATACCACCAACAAAAACAAACCTATTCCTACCATTCCTTATCCTGGCCCTCTGGGGGGCA

ATTTTAGCTAGTCTAACCTGCCTACAACAAACAGACCTAAAATCTCTAATTGCATACTCC

TCAATCAGTCATATAGGACTAGTAATTGCATCAATCCTACTTCAAACTCAATGGGGCTTA

TCTGGGGCTATATATTTAATAATTGCCCACGGATTCACGTCATCAATATTATTCTGCCTA

GCCAATACAACCTATGAACGAACAAACACCCGCATCCTTATACTAACACGAGGGTTTCAC

AACATCCTACCAATAACTACAGCCTGATGACTACTAGCCAATTTAATGAACATCGCCATC

CCACCTAGTCTCAACTTCACAGGAGAATTACTAATTGCATCATCCCTGTTCAACTGGTGT

CCAATTACAATAATCTTATTTGGCCTGTCTATACTAATTACCATCTCATATTCATTACAA

GTATTTTTATCTACCCAAATAGGCAAAACATTATTAAACACACCCACACAACCAATACAC

ACACGAGAACATCTACTAATGACCCTCCACATCATCCCCCTTATTATAATCTCAATAAAA

CCAGAACTTGTATTC

>MZ712259_CAS247982_[Pareas_carinatus_tenasserimicus]

CTAGCAGCAATTCTTCTTAAACTTGGAGGCTATGGATTAATCCGAATAATTCAATTTATA

CCCACAACAAAAACAGATCTATTCCTACCATTCCTTATTATAGCCCTCTGAGGGGCAATT

TTAGCCAGTCTAACCTGCTTGCAACAAACAGATCTAAAATCTCTAATTGCATACTCCTCT

ATTAGCCATATGGGGTTAGTAGTTGCAGCAATTCTTCTACAAACCCAGTGGGGTTTATCG

GGGGCTATGTCTTTAATAGTTGCTCATGGGTTCACATCATCAATACTATTCTGTCTCGCC

AACACAACCTATGAACGAACAAACACCCGCATCTTAATCTTAACACGAGGCTTTCATAAT

ATCCTTCCTATAACTACAGCCTGATGACTACTAGCCAACTTAATAAACATTGCTATTCCA

CCAAGTCTTAACTTCACAGGAGAACTACTTATTGCATCGTCCCTGTTCAACTGAAGTCCA

ACTACAATAATTTTATTCGGTTTATCTATGTTAATTACCGTTTCATACTCTCTACAAGTA

TTTATATCCACCCAAATAGGCACCCCCCTATTAAACACACCCACACAGCCAACACACTCA

CGAGAACATCTACTTATGATCTTACACATCATTCCCCTTATTATAATCTCAATAAAACCA

GAACTTGTATTC

>MZ712260_ZMMUR16800_[Pareas_carinatus_tenasserimicus]

CTAGCAGCAATTCTTCTTAAACTTGGAGGCTACGGGTTAATCCGAATAATTCAAATCATA

CCCACAACAAAAACAGATCTTTTCCTACCATTCCTTATTATAGCCCTATGGGGGGCAATT

TTAGCCAGTCTAACCTGCTTGCAACAAACAGATCTAAAATCTCTAATTGCATACTCCTCT

ATTAGCCACATAGGACTAGTAATTGCAGCAATTCTTCTACAAACTCAGTGGGGTTTATCT

GGGGCTATATCTTTAATAGTCGCTCATGGGTTCACATCATCAATACTATTCTGTCTCGCC

AACACAACCTATGAACGAACAAACACCCGCATCTTAATCTTAACACGAGGCTTCCATAAT

ATCCTACCTATAACTACAGCCTGATGACTACTAGCCAACTTAATAAATATTGCCATTCCA

CCAAGTCTTAACTTCACAGGAGAACTACTTATCGCATCGTCCCTATTCAACTGATGTCCA

ACTACAATAATTTTATTTGGTTTATCTATGTTAATTACCATTTCATACTCTCTACAAGTA

TTTATATCCACCCAAATAGGCACCCCCCTATTAAACACACCCACACAACCAACACACTCA

CGAGAACATCTACTTATGGTCTTACACATCATTCCCCTTATCATAATCTCAATAAAACCA

GAACTTGTATTC

>MZ712261_LSUHC10604_[Pareas_carinatus_carinatus]

GCAATTCTTCTTAAACTTGGAGGCTATGGATTAATCCGAATAATTCAAATTATACCTTCA

ACAAAAACAGATCTATTCTTACCATTCCTTGTTATAGCCCTCTGAGGGGCAATTTTAGCC

GGTTTAACCTGCTTGCAACAAACAGATCTAAAATCTCTAATTGCATACTCCTCTATTAGC
CACATAGGGCTAGTAATTGCAGCAATTCTTCTACAAACTCAGTGGGGCTTATCGGGGGCC
ATATCTTTAATAGTTGCCCATGGGTTCACATCATCAATACTATTCTGCCTCGCTAACACA
ACCTATGAACGTACAAACACCCGCATCTTAATCTTAACACGAGGCTTTCATAATATCTTG
CCTATAACTACAGCCTGATGACTATTAGCCAACTTAATAAACATTGCCATTCCACCAAGC
CTTAACTTCACAGGAGAACTACTAATTGCATCGTCTCTATTCAACTGATGTACAACTACA
ATAATTTTATTCGGTTTATCTATGTTAATTACCGTTTCATACTCCCTACAAGTATTTATA
TCCACCCAAATAGGCACCCCCCTATTAAACATACCAACACAGCCAACACACTCACGAGAA
CATCTACTTATGATCTTACACATCATCCCCCTTATCATAATCTCAATTAAACCAGAACTT
GTATTC
>MZ712262_ZMMUR16393_[Pareas_abros]
CTAGCAGCAATTCTTCTAAAATTGGGGGGTTATGGCTTGATCCGAATAATACAAATTATA
CCCACCCCAAAAACAGATATATTCACCCCTTTTCTCGTGTTAGCCCTTTGAGGGGCAATT
TTAGCCAACCTAACTTGTCTGCAACAAACAGACCTAAAATCTTTAATCGCATACTCATCT
ATTAGCCACATAGGCCTAGTAATCGCATCAATCATATTAGGAACCGAGTGAAGCCTATCC
GGGGCTATATCGTTAATAATCGCCCACGGATTCACATCATCAATACTATTCTGTCTTGCC
AATACCACCTATGAACGAACAAACACTCGCATCTTAATCCTAACACGAGGATTTCATAAC
CTTTTACCAATGACCACAGCCTGATGATTAATAGCTAACCTGTTAAACATCGCCGTACCA
CCTAGCCTTAACTTTACAGGAGAACTATTAATCGCATCATCCTTATTCAACTGATGTCCA
ACAACAATGTTCATATTTGGCCTCTCTATACTAATTACTGCCTCCTACTCCATACACATG
TTTATATCTACCCAGATAGGGACACCACTACTAAACGTCTCAACACAACCAACACACTCA
CGAGAACATCTACTTATAACCCTACACATAAGCCCTCTTATCCTAATCTCAATAAAACCA
GAGCTTGTATTT
>MZ712263_ZMMUR16392_[Pareas_abros]
CTAGCAGCAATTCTTCTAAAATTGGGGGGTTATGGCTTGATCCGAATAATACAAATTATA
CCCACCCCAAAAACAGATATATTCACCCCTTTTCTCGTGTTAGCCCTTTGAGGGGCAATT
TTAGCCAACCTAACTTGTCTGCAACAAACAGACCTAAAATCTTTAATCGCATACTCATCT
ATTAGCCACATAGGCCTAGTAATCGCATCAATCATATTAGGAACCGAGTGAAGCCTATCC
GGGGCTATATCGTTAATAATCGCCCACGGATTCACATCATCAATACTATTCTGTCTTGCC

AATACCACCTATGAACGAACAAACACTCGCATCTTAATCCTAACACGAGGATTTCATAAC

CTTTTACCAATGACCACAGCCTGATGATTAATAGCTAACCTGTTAAACATCGCCGTACCA

CCTAGCCTTAACTTTACAGGAGAACTATTAATCGCATCATCCTTATTCAACTGATGTCCA

ACAACAATGTTCATATTTGGCCTCTCTATACTAATTACTGCCTCCTACTCCATACACATG

TTTATATCTACCCAGATAGGGACACCACTACTAAACGTCTCAACACAACCAACACACTCA

CGAGAACATCTACTTATAACCCTACACATAAGCCCTCTTATCCTAATCTCAATAAAACCA

GAGCTTGTATTT

>MZ712264_ZMMUR14788_[Pareas_abros]

CTAGCAGCAATTCTTCTAAAATTGGGGGGTTATGGCTTGATCCGAATAATACAAATTATA

CCCACCCCAAAAACAGATATATTCACCCCTTTTCTCGTGTTAGCCCTTTGAGGGGCAATT

TTAGCCAACCTAACTTGTCTGCAACAAACAGACCTAAAATCTTTAATCGCATACTCATCT

ATTAGCCACATAGGCCTAGTAATCGCATCAATCATATTAGGAACCGAGTGAAGCCTATCC

GGGGCTATATCGTTAATAATCGCCCACGGATTCACATCATCAATACTATTCTGTCTTGCC

AATACCACCTATGAACGAACAAACACTCGCATCTTAATCCTAACACGAGGATTTCATAAC

CTTTTACCAATGACCACAGCCTGATGATTAATAGCTAACCTGTTAAACATCGCCGTACCA

CCTAGCCTTAACTTTACAGGAGAACTATTAATTGCTTCATCCTTATTCAACTGATGTCCA

ACAACAATGTTCATATTTGGCCTCTCTATACTAATTACTGCCTCCTACTCCATACACATG

TTTATATCTACCCAGATAGGGACACCACTACTAAACGTCTCAACACAACCAACACACTCA

CGAGAACATCTACTTATAACCCTACACATAAGCCCTCTTATCCTAATCTCAATAAAACCA

GAGCTTGTATTT

>MZ712265_ZMMUR13656_[Pareas_temporalis]

CTAGCAGCCATTCTTCTAAAATTGGGGGGTTACGGGCTGATCCGGATAATACAAATTATA

CCCACCCCAAAAACAGACATGTTTATCCCATTTCTCGTATTAGCCCTTTGAGGGGCTATT

TTAGCCAATCTCACTTGCCTACAACAAACAGACCTAAAATCTTTAATCGCATACTCATCT

ATTAGCCATATGGGTCTAGTAATCGCATCAATCATGCTAGGAACCCAATGAAGTCTATCC

GGGGCTATATCATTAATAATCGCCCACGGATTCACGTCATCCATACTATTCTGTCTTGCC

AACACCACCTATGAACGAACAAATACTCGCATCCTAATCCTGACACGGGGGTTTCATAAC

CTTTTACCGATAACCACCGCCTGATGACTAATAGCCAATCTATTAAACATCGCCGTACCA

CCCAGCCTTAACTTTACAGGAGAACTACTAATCGCATCATCCTTATTCAACTGATGTCCA

ACAACAATAATTATATTTGGTCTCTCTATACTAATCACTGCCTCCTACTCCCTACACATA

TTTATATCCACCCAAATAGGCACACCCTTACTAAACACCCCAACACAACCAACACACTCA

CGAGAACACCTACTTATAACTCTACACATAACCCCTCTTATTATAATCTCAATAAAACCA

GAACTTGTATTT

>MZ712266_SIEZC20215_[Pareas_temporalis]

CTAGCAGCCATTCTTCTAAAATTGGGGGGTTACGGGCTGATCCGGATAATACAAATTATA

CCCACCCCAAAAACAGACATGTTTATCCCATTTCTCGTATTAGCCCTTTGAGGGGCTATT

TTAGCCAATCTCACTTGCCTACAACAAACAGACCTAAAATCTTTAATCGCATACTCATCT

ATTAGCCATATGGGTCTAGTAATCGCATCAATCATGCTAGGAACCCAATGAAGTCTATCC

GGGGCTATATCATTAATAATCGCCCACGGATTCACGTCATCCATACTATTCTGTCTTGCC

AACACCACCTATGAACGAACAAATACTCGCATCCTAATCCTGACACGGGGGTTTCATAAC

CTTTTACCGATAACCACCGCCTGATGACTAATAGCCAATCTATTAAACATCGCCGTACCA

CCCAGCCTTAACTTTACAGGAGAACTACTAATCGCATCATCCTTATTCAACTGATGTCCA

ACAACAATAATTATATTTGGTCTCTCTATACTAATCACTGCCTCCTACTCCCTACACATA

TTTATATCCACCCAAATAGGCACACCCTTACTAAACACCCCAACACAACCAACACACTCA

CGAGAACACCTACTTATAACTCTACACATAACCCCTCTTATTATAATCTCAATAAAACCA

GAACTTGTATTT

>MZ712267_LSUHC9098_[Asthenodipsas_lasgalenensis]

AATCTAGCAGCAATCTTATTAAAATTGGGAGGGTACGGCATTATTCGAATAATACAAACT

TTACCAATTATAAAAACCGACACATTTCTCCCATTCATTATTCTTGCCCTATGGGGAGCA

ATCCTGGCTAATATAACCTGCTTACAACAAACCGACCTAAAATCGCTAATTGCATATTCA

TCAATCAGCCACATAGGCCTAGTAATTGCAGCCGTCGTTTTACAAACTCAATGAGCACTA

TCAGGGGCCATAACCCTAATAATTGCCCATGGCTTTACCTCTTCTGCCCTTTTCTGCCTT

GCCAATAGCACATATGAACGAACCAACACTCGTATTTTAATTCTCACTCGAGGGTTCCAC

AACATCCTTCCAATAGCTACAACCTGGTGAATATTAATTAATCTTTTAAACATCGCCATT

CCGCCTAGCCTAAATTTTACTGGTGAACTACTAATTGCATCATCCCTTTTCAACTGATGT

CCAACAACAATTATTATATTTGGACTATCTATGTTAATCTCCGCCTCCTACTCACTACAT

CTATTTCTATCCACCCAAATAGGAACACCACAATTAAACCAAATAATCTCACCAACACAC

TCTCGAGAACACCTCCTTTTATCTTTACATGTCATTCCACTAATTTTAATTTCAATAAAA

CCTGAACTGGTATTT

>MZ712268_Avpt_[Asthenodipsas_vertebralis]

AATCTAGCAGCAATCTTATTAAAATTGGGAGGGTACGGCATTATTCGAATAATACAAACT

TTACCAATTATAAAAACCGACACATTTCTCCCATTCATTATTCTTGCCCTATGGGGAGCA

ATCCTGGCTAATATAACCTGCTTACAACAAACCGACCTAAAATCGCTAATTGCATATTCA

TCAATCAGCCACATAGGCCTAGTAATTGCAGCCGTCGTTTTACAAACTCAATGAGCACTA

TCAGGGGCCATAACCCTAATAATTGCCCATGGCTTTACCTCTTCTGCCCTTTTCTGCCTT

GCCAATAGCACATATGAACGAACCAACACTCGTATTTTAATTCTCACTCGAGGGTTCCAC

AACATCCTTCCAATAGCTACAACCTGATGAATATTAATTAATCTTTTAAACATCGCCATT

CCGCCTAGCCTAAATTTTACTGGTGAACTACTAATTGCATCATCCCTTTTCAACTGATGT

CCAACAACAATTATTATATTTGGACTATCTATATTAATCTCCGCCTCCTACTCACTACAT

CTATTTCTATCCACCCAAATAGGAACACCACAATTAAACCAAATAATCTCACCAACACAC

TCTCGAGAACACCTCCTTTTATCTTTACATGTCATTCCACTAATTTTAATTTCAATAAAA

CCTGAACTGGTATTT

***cmos* nuDNA gene**

>MZ712269_NMNH05625_[Pareas_komaii]

GATCAGTTATGTCTCTTGCATCTCCTGGGCTCTGGGGGCTTTGGCTCTGTCTACAAGGCA

ACTTACCAAGGAGCTACAGTGGCTGTAAAACAAGTGAAGAGATGCAGTAAGAACCATTTG

GCATCACGGCAAAGCTTCTGGGCAGAACTAAATGTAGCACGTCTTGACCATAACAATGTG

GTACACATAGTAGCTGCTAGCACATGTACCCCTGCTAGTCAGGATAGTTTGGGTGCCATA

ATTATGGAATATGCAGGTAATTGCACTCTACATCATATTATCTATGGGACTGGCTGTTTA

ACAGGAAATAACAAGGATGGCCTTCAGTGTGATCTTGAGTTCTTGAGTACAGCTCAGGCT

GTCATTTACGCCTGTGATATTGTGGCAGGGTTAATGTTCCTCCATTCTCAGTTAATTGTG

CATCTCGATTTAAAACCTGCTAACATATTCATCACGGAGCATAATGTTTGCAAAATTGGA

GACTTTGGATGCTCCCAAAAGCTAGAAGATAGTGAATCATCAGGACTACATCTTTGTCAT

CAAGGGGGAACATACACACATCGTGCTCCTGAACTTCTTAAAGGTGAGAAAATCACACCC

AA

>MZ712270_NMNH05654_[Pareas_iwasakii]

GATCAGTTATGTCTCTTGCATCTCCTGGGCTCTGGGGGCTTTGGCTCTGTCTACAAGGCA

ACTTACCAAGGAGCTACAGTGGCTGTAAAACAAGTGAAGAGATGCAGTAAGAACCATTTG

GCATCACGGCAAAGCTTCTGGGCAGAACTAAATGTAGCACGCCTTGATCATAACAATGTG

GTACACATAGTAGCTGCTAGCACATGTACCCCTGCTAGTCAGGATAGTTTGGGTGCCATA

ATTATGGAATATGCAGGTAATTGCACTCTACATCATATTATCTATGGGACTGGCTGTTTA

ACAGGAAATAACAAGGATGGCCTTCAGTGTGATCTTGAGTTCTTGAGTACAGCTCAGGCT

GTCATTTACGCCTGTGATATTGTGGCAGGGTTAATGTTCCTCCATTCTCAGTTAATTGTG

CATCTCGATTTAAAACCTGCTAACATATTCATCACGGAACATAATGTTTGCAAAATTGGA

GACTTTGGATGCTCCCAAAAGCTAGAAGATAGTGAATCATCAGGACTACATCTTTGTCAT

CAAGGGGGAACATACACACATCGTGCTCCTGAACTTCTTAAAGGTGAGAAAATCACACCC

AA

>MZ712271_ZMMUR16628_[Pareas_macularius]

GATCAGTTATGTTTCTTGCATCTCCTGGGCTCTGGGGGCTTTGGTTGTGTCTACAAGGCA

ACTTACCAAGGAGCTACAGTGGCTGTAAAACAAGTGAAGAGATGCAGTAAGAACCATTTG

GCATCACGGCAAAGCTTCTGGGGAGAACTAAATGTAGCCCGCCTTGACCATAACAATGTG

GTACATATAGTAGCTGCCAGCACATGTACCCCTGCTAGTCAGGATAGTTTGGGTACCATA

ATTATGGAATATGCAGGTAATTGCACTCTACATCATATTATCTATGGGACTGGCTATTTA

ACAGGAAATAACAATGATGGCCTTCAATATGATCTTGAGTTCTTGAGTACAGCTCAGGCT

GTCATTTATGCCTGTGATATTGTGACAGGGTTAATGTTTCTCCATTCTCAGTTAATTGTG

CATATGGATTTAAAACCTGCTAACATATTCATCACAGAACATAATGTTTGCAAAATTGGA

GACTTTGGATGCTCCCAAAAGCTAGAAGATAGTGAATCATCAGGACTACATCTTTGTCAT

CAAGGGGGAACATACACACATCGTGCTCCTGAACTTCTTAAAGGTGAGAAAATCACACCC

AA

>MZ712272_CIB098271_[Pareas_margaritophorus]

GATCAGTTATGTTTCTTGCATCTCCTGGGCTCTGGAGGCTTTGGTTGTGTCTACAAGGCA

ACTTACCAAGGAGCTACAGTGGCTGTAAAACAAGTGAAGAGATGCAGTAAGAACCATTTG

GCATCACGGCAAAGCTTCTGGGCAGAACTAAATGTAGCACGCCTTGACCATAACAATGTG

GTACACATAGTAGCTGCTAGCACATGTACCCCTGCTAGTCAGGATAGTTTGGGTACCATA

ATTATGGAATATGCAGGTAATTGCACTCTACATCATATTATCTATGGAACTGGCTATTTA

ACAGGAAATAACAATGATGGCCTTCAATATGATCTTGAGTTCTTGAGTACAGCTCAGGCT
GTCATTTACGCCTGTGATATTGTGACAGGGTTAATGTTTCTCCATTCTCAGTTAATTGTG
CATCTGGATTTAAAACCTGCTAACATATTCATCACAGAACATAATGTTTGCAAAATTGGA
GACTTTGGATGCTCCCAAAAGCTAGAAGATAGTGAATCATCAGGACTACATCTTTGTCAT
CAAGGGGGAACATACACACATCGTGCTCCTGAACTTCTTAAAGGTGAGAAAATCACACCC
AA
>MZ712273_ZMMUNAP09759_[Pareas_margaritophorus]
TATTGGACTTGGGATCAGTTATGTTTCTTGCATCTCCTGGGCTCTGGGGGCTTTGGTTGT
GTCTACAAGGCAACTTACCAAGGAGCTACAGTGGCTGTAAAACAAGTGAAGAGATGCAGT
AAGAACCATTTGGCATCACGGCAAAGCTTCTGGGCAGAACTAAATGTAGCACGCCTTGAC
CATAACAATGTGGTACACATAGTAGCTGCTAGCACATGTACCCCTGCTAGTCAGGATAGT
TTGGGTACCATAATTATGGAATATGCAGGTAATTGCACTCTACATCATATTATCTATGGG
ACTGGCTATTTAACAGGAAATAACAATGATGGCCTTCAATATGATCTAGAGTTCTTGAGT
ACAGCTCAGGCTGTCATTTACGCCTGTGATATTGTGACAGGGTTAATGTTTCTCCATTCT
CAGTTAATTGTGCATCTGGATTTAAAACCTGCTAACATATTCATCACAGAACATAATGTT
TGCAAAATTGGAGACTTTGGATGCTCCCAAAAGCTAGAAGATAGTGAATCATCAGGACTA
CATCTTTGTCATCAAGGGGGAACATACACACATCGTGCTCCTGAACTTCTTAAAGGTGAG
AAAATCCAAACCCA
>MZ712274_FMNH255567_[Pareas_formosensis]
GATCAGTTATGTCTCTTGCATCTCCTGGGCTCTGGGGGCTTTGGCTCTGTCTACAAGGCA
ACTTACCAAGGAGCTACAGTGGCTGTAAAACAAGTGAAGAGATGCAGTAAGAACCATTTG
GCATCACGGCAAAGCTTCTGGGCAGAACTAAATGTAGCACGCCTTGACCATAACAATGTG
GTACACATAGTAGCTGCTAGCACATGTACCCCTGCTAGTCAGGATAGTTTGGGTGCCATA
ATTATGGAATATGCAGGTAATTGCACTCTACATCATATTATCTATGGGACTGGCTGTTTA
ACAAGAAATAACAAGGATGGCCTTCAGTGTGATCTTGAGTTCTTGAGTACAGCTCAGGCT
GTCATTTACGCCTGTGATATTGTGGCAGGGTTAATGTTCCTCCATTCTCAGTTAATTGTG
CATCTGGATTTAAAACCTGCTAACATATTCATCACGGAACATAATGTTTGCAAAATTGGA
GACTTTGGATGCTCCCAAAAGCTAGAAGATAGTGAATCATCAGGACTACATCTTTGTCAT
CAAGGGGGAACATACACACATCGTGCTCCTGAACTTCTTAAAGGTGAGAAAATCACACCC

AA

>MZ712275_CIB098270_[Pareas_menglaensis]

GATCAGTTATGTCTCCTGCATCTCCTGGGCTCTGGGGGCTTTGGTTCTGTCTACAAGGCA

ACTTACCAAGGAGCTACAGTGGCTGTAAAACAGGTGAAGAGATGCAGTAAGAACCATTTG

GCATCACGGCAAAGCTTCTGGGCAGAACTAAATGTAGCACGCCTTGACCATAACAATGTG

GTACACATAGTAGCTGCTAGCACATGTACTCCTGCTAGTCAGGATAGTCTGGGTACCATA

ATTATGGAATATGCAGGTAATTGCACTCTACATCATATTATCTATGGGACTGGCTATTTA

ACAGGAAATAACAATGATGGCCTTCAATGTGATTTTGAGTTCTTGACTACAGCTCAGGCT

GTCATTTACGCCTGTGATATTGTGGCGGGCTTAATGTTTCTCCATTCTCAGTTAATTGTG

CATCTGGATTTAAAACCTGCTAACATATTCATCACAGAACATAGTGTTTGCAAAATTGGA

GACTTTGGATGCTCCCAAAAGCTAGAAGATAGTGAATCATCAGGACTACATCTTTGTCAT

CAAGGGGGAACATACACACATCGTGCTCCTGAACTTCTTAAAGGTGAGAAAATCACACCC

AA

>MZ712276_AUP01573_[Pareas_berdmorei_berdmorei]

GATCAGTTATGTCTCCTGCATCTCCTGGGCTCTGGGGGCTTTGGTTCTGTCTACAAGGCA

ACTTACCAAGGAGCTACAGTGGCTGTAAAACAGGTGAAGAGATGCAGTAAGAACCATTTG

GCATCACGGCAAAGCTTCTGGGCAGAACTAAATGTAGCACGCCTTGACCATAACAATGTG

GTACACATAGTAGCTGCTAGCACATGTACTCCTGCTAGTCAGGATAGTCTGGGTACCATA

ATTATGGAATATGCAGGTAATTGCACTCTACATCATATTATCTATGGGACTGGCTATTTA

ACAGGAAATAACAATGATGGCCTTCAATGTGATTTTGAGTTCTTGACTACAGCTCAGGCT

GTCATTTACGCCTGTGATATTGTGGCGGGCTTAATGTTTCTCCATTCTCAGTTAATTGTG

CATCTGGATTTAAAACCTGCTAACATATTCATCACAGAACATAGTGTTTGCAAAATTGGA

GACTTTGGATGCTCCCAAAAGCTAGAAGATAGTGAATCATCAGGACTACATCTTTGTCAT

CAAGGGGGAACATACACACATCGTGCTCCTGAACTTCTTAAAGGTGAGAAAATCACACCC

AA

>MZ712277_CAS240362_[Pareas_berdmorei_berdmorei]

GATCAGTTATGTCTCCTGCATCTCCTGGGCTCTGGAGGCTTTGGTTCTGTCTACAAGGCA

ACTTACCAAGGAGCTACAGTGGCTGTAAAACAGGTGAAGAGATGCAGTAAGAACCATTTG

GCATCACGGCAAAGCTTCTGGGCAGAACTAAATGTAGCACGCCTTGACCATAACAATGTG

GTACACATAGTAGCTGCTAGCACATGTACTCCTGCTAGTCAGGATAGTCTGGGTACCATA

ATTATGGAATATGCAGGTAATTGCACTCTACATCATATTATCTATGGGACTGGCTATTTA

ACAGGAAATAACAATGATGGCCTTCAATGTGATTTTGAGTTCTTTACTACAGCTCAGGCT

GTCATTTACGCCTGTGATATTGTGGCGGGCTTAATGTTTCTCCATTCTCAGTTAATTGTG

CATCTGGATTTAAAACCTGCTAACATATTCATCACAGAACATAGTGTTTGCAAAATTGGA

GACTTTGGATGCTCCCAAAAGCTAGAAGATAGTGAATCATCAGGACTACATCTTTGTCAT

CAAGGGGGAACATACACACATCGTGCTCCTGAACTTCTTAAAGGTGAGAAAATCACACCC

AA

>MZ712278_ZMMUR16803_[Pareas_berdmorei_berdmorei]

GATCAGTTATGTCTCCTGCATCTCCTGGGCTCTGGAGGCTTTGGTTCTGTCTACAAGGCA

ACTTACCAAGGAGCTACAGTGGCTGTAAAACAGGTGAAGAGATGCAGTAAGAACCATTTG

GCATCACGGCAAAGCTTCTGGGCAGAACTAAATGTAGCACGCCTTGACCATAACAATGTG

GTACACATAGTAGCTGCTAGCACATGTACTCCTGCTAGTCAGGATAGTCTGGGTACCATA

ATTATGGAATATGCAGGTAATTGCACTCTACATCATATTATCTATGGGACTGGCTATTTA

ACAGGAAATAACAATGATGGCCTTCAATGTGATTTTGAGTTCTTTACTACAGCTCAGGCT

GTCATTTACGCCTGTGATATTGTGGCGGGCTTAATGTTTCTCCATTCTCAGTTAATTGTG

CATCTGGATTTAAAACCTGCTAACATATTCATCACAGAACATAGTGTTTGCAAAATTGGA

GACTTTGGATGCTCCCAAAAGCTAGAAGATAGTGAATCATCAGGACTACATCTTTGTCAT

CAAGGGGGAACATACACACATCGTGCTCCTGAACTTCTTAAAGGTGAGAAAATCACACCC

AA

>MZ712279_ZMMUR137531_[Pareas_berdmorei_unicolor]

GATCAGTTATGTCTCCTGCATCTCCTGGGCTCTGGGGGCTTTGGTTCTGTCTACAAGGCA

ACTTACCAAGGAGCTACAGTGGCGGTAAAACAGGTGAAGAGATGCAGTAAGAACCATTTG

GCATCACGGCAAAGCTTCTGGGCAGAGCTAAATGTAGCACGCCTTGACCATAACAATGTG

GTACACATAGTAGCTGCTAGCACATGTACCCCTGCTAGTCAGGATAGTCTGGGTACCATA

ATTATGGAATATGCAGGTAATTGCACTCTACATCATATTATCTATGGGACTGGCTATTTA

ACAGGAAATAACAATGATGGCCTTCAATGTGATTTTGAGTTCTTGACTACAGCTCAGGCT

GTCATTTACGCCTGTGATATTGTGGCGGGCTTAATGTTTCTCCATTCTCAGTTAATTGTG

CATCTGGATTTAAAACCTGCTAACATATTCATCACAGAACATAGTGTTTGCAAAATTGGA

GACTTTGGATGCTCCCAAAAGCTAGAAGATAGTGAATCATCAGGACTACATCTTTGTCAT

CAAGGGGGAACATACACACATCGTGCTCCTGAACTTCTTAAAGGTGAGAAAATCACACCC

AA

>MZ712280_ZMMUR137532_[Pareas_berdmorei_unicolor]

GATCAGTTATGTCTCCTGCATCTCCTGGGCTCTGGGGGCTTTGGTTCTGTCTACAAGGCA

ACTTACCAAGGAGCTACAGTGGCGGTAAAACACGTGAAGAGATGCAGTAAGAACCATTTG

GCATCACGGCAAAGCTTCTGGGCAGAGTTAAATGTAGCACGCCTTGACCATAACAATGTG

GTACACATAGTAGCTGCTAGCACATGTACCCCTGCTAGTCAGGATAGTCTGGGTACCATA

ATTATGGAATATGCAGGTAATTGCACTCTACATCATATTATCTATGGGACTGGCTATTTA

ACAGGAAATAACAATGATGGCCTTCAATGTGATTTTGAGTTCTTGACTACAGCTCAGGCT

GTCATTTACGCCTGTGATATTGTGGCGGGCTTAATGTTTCTCCATTCTCAGTTAATTGTG

CATCTGGATTTAAAACCTGCTAACATATTCATCACAGAACATAGTGTTTGCAAAATTGGA

GACTTTGGATGCTCCCAAAAGCTAGAAGATAGTGAATCATCAGGACTACATCTTTGTCAT

CAAGGGGGAACATACACACATCGTGCTCCTGAACTTCTTAAAGGTGAGAAAATCACACCC

AA

>MZ712281_ZMMUR14013_[Pareas_berdmorei_unicolor]

GATCAGTTATGTCTCCTGCATCTCCTGGGCTCTGGGGGCTTTGGTTCTGTCTACAAGGCA

ACTTACCAAGGAGCTACAGTGGCGGTAAAACAGGTGAAGAGATGCAGTAAGAACCATTTG

GCATCACGGCAAAGCTTCTGGGCAGAGCTAAATGTAGCACGCCTTGACCATAACAATGTG

GTACACATAGTAGCTGCTAGCACATGTACCCCTGCTAGTCAGGATAGTCTGGGTACCATA

ATTATGGAATATGCAGGTAATTGCACTCTACATCATATTATCTATGGGACTGGCTATTTA

ACAGGAAATAACAATGATGGCCTTCAATGTGATTTTGAGTTCTTGACTACAGCTCAGGCT

GTCATTTACGCCTGTGATATTGTGGCGGGCTTAATGTTTCTCCATTCTCAGTTAATTGTG

CATCTGGATTTAAAACCTGCTAACATATTCATCACAGAACATAGTGTTTGCAAAATTGGA

GACTTTGGATGCTCCCAAAAGCTAGAAGATAGTGAATCATCAGGACTACATCTTTGTCAT

CAAGGGGGAACATACACACATCGTGCTCCTGAACTTCTTAAAGGTGAGAAAATCACACCC

AA

>MZ712282_ZMMUR14263_[Pareas_berdmorei_unicolor]

GATCAGTTATGTCTCCTGCATCTCCTGGGCTCTGGGGGCTTTGGTTCTGTCTACAAGGCA

ACTTACCAAGGAGCTACAGTGGCGGTAAAACAGGTGAAGAGATGCAGTAAGAACCATTTG
GCATCACGGCAAAGCTTCTGGGCAGAGCTAAATGTAGCACGCCTTGACCATAACAATGTG
GTACACATAGTAGCTGCTAGCACATGTACCCCTGCTAGTCAGGATAGTCTGGGTACCATA
ATTATGGAATATGCAGGTAATTGCACTCTACATCATATTATCTATGGGACTGGCTATTTA
ACAGGAAATAACAATGATGGCCTTCAATGTGATTTTGAGTTCTTGACTACAGCTCAGGCT
GTCATTTACGCCTGTGATATTGTGGCGGGCTTAATGTTTCTCCATTCTCAGTTAATTGTG
CATCTGGATTTAAAACCTGCTAACATATTCATCACAGAACATAGTGTTTGCAAAATTGGA
GACTTTGGATGCTCCCAAAAGCTAGAAGATAGTGAATCATCAGGACTACATCTTTGTCAT
CAAGGGGGAACATACACACATCGTGCTCCTGAACTTCTTAAAGGTGAGAAAATCACACCC
AA
>MZ712283_ZMMUR14421_[Pareas_berdmorei_unicolor]
GATCAGTTATGTCTCCTGCATCTCCTGGGCTCTGGGGGCTTTGGTTCTGTCTACAAGGCA
ACTTACCAAGGAGCTACAGTGGCGGTAAAACATGTGAAGAGATGCAGTAAGAACCATTTG
GCATCACGGCAAAGCTTCTGGGCAGAGCTAAATGTAGCACGCCTTGACCATAACAATGTG
GTACACATAGTAGCTGCTAGCACATGTACCCCTGCTAGTCAGGATAGTCTGGGTACCATA
ATTATGGAATATGCAGGTAATTGCACTCTACATCATATTATCTATGGGACTGGCTATTTA
ACAGGAAATAACAATGATGGCCTTCAATGTGATTTTGAGTTCTTGACTACAGCTCAGGCT
GTCATTTACGCCTGTGATATTGTGGCGGGCTTAATGTTTCTCCATTCTCAGTTAATTGTG
CATCTGGATTTAAAACCTGCTAACATATTCATCACAGAACATAGTGTTTGCAAAATTGGA
GACTTTGGATGCTCCCAAAAGCTAGAAGATAGTGAATCATCAGGACTACATCTTTGTCAT
CAAGGGGGAACATACACACATCGTGCTCCTGAACTTCTTAAAGGTGAGAAAATCACACCC
AA
>MZ712284_SIEZC20216_[Pareas_berdmorei_unicolor]
GATCAGTTATGTCTCCTGCATCTCCTGGGCTCTGGGGGCTTTGGTTCTGTCTACAAGGCA
ACTTACCAAGGAGCTACAGTGGCGGTAAAACAGGTGAAGAGATGCAGTAAGAACCATTTG
GCATCACGGCAAAGCTTCTGGGCAGAGCTAAATGTAGCACGCCTTGACCATAACAATGTG
GTACACATAGTAGCTGCTAGCACATGTACCCCTGCTAGTCAGGATAGTCTGGGTACCATA
ATTATGGAATATGCAGGTAATTGCACTCTACATCATATTATCTATGGGACTGGCTATTTA
ACAGGAAATAACAATGATGGCCTTCAATGTGATTTTGAGTTCTTGACTACAGCTCAGGCT

GTCATTTACGCCTGTGATATTGTGGCGGGCTTAATGTTTCTCCATTCTCAGTTAATTGTG

CATCTGGATTTAAAACCTGCTAACATATTCATCTCAGAACATAGTGTTTGCAAAATTGGA

GACTTTGGATGCTCCCAAAAGCTAGAAGATAGTGAATCATCAGGACTACATCTTTGTCAT

CAAGGGGGAACATACACACATCGTGCTCCTGAACTTCTTAAAGGTGAGAAAATCACACCC

AA

>MZ712285_ZMMUR136791_[Pareas_berdmorei_unicolor]

GATCAGTTATGTCTCCTGCATCTCCTGGGCTCTGGGGGCTTTGGTTCTGTCTACAAGGCA

ACTTACCAAGGAGCTACAGTGGCGGTAAAACAGGTGAAGAGATGCAGTAAGAACCATTTG

GCATCACGGCAAAGCTTCTGGGCAGAGCTAAATGTAGCACGCCTTGACCATAACAATGTG

GTACACATAGTAGCTGCTAGCACATGTACCCCTGCTAGTCAGGATAGTCTGGGTACCATA

ATTATGGAATATGCAGGTAATTGCACTCTACATCATATTATCTATGGGACTGGCTATTTA

ACAGGAAATAACAATGATGGCCTTCAATGTGATTTTGAGTTCTTGACTACAGCTCAGGCT

GTCATTTACGCCTGTGATATTGTGGCGGGCTTAATGTTTCTCCATTCTCAGTTAATTGTG

CATCTGGATTTAAAACCTGCTAACATATTCATCACAGAACATAGTGTTTGCAAAATTGGA

GACTTTGGATGCTCCCAAAAGCTAGAAGATAGTGAATCATCAGGACTACATCTTTGTCAT

CAAGGGGGAACATACACACATCGTGCTCCTGAACTTCTTAAAGGTGAGAAAATCACACCC

AA

>MZ712286_ZMMUR136792_[Pareas_berdmorei_unicolor]

GATCAGTTATGTCTCCTGCATCTCCTGGGCTCTGGGGGCTTTGGTTCTGTCTACAAGGCA

ACTTACCAAGGAGCTACAGTGGCGGTAAAACAGGTGAAGAGATGCAGTAAGAACCATTTG

GCATCACGGCAAAGCTTCTGGGCAGAGCTAAATGTAGCACGCCTTGACCATAACAATGTG

GTACACATAGTAGCTGCTAGCACATGTACCCCTGCTAGTCAGGATAGTCTGGGTACCATA

ATTATGGAATATGCAGGTAATTGCACTCTACATCATATTATCTATGGGACTGGCTATTTA

ACAGGAAATAACAATGATGGCCTTCAATGTGATTTTGAGTTCTTGACTACAGCTCAGGCT

GTCATTTACGCCTGTGATATTGTGGCGGGCTTAATGTTTCTCCATTCTCAGTTAATTGTG

CATCTGGATTTAAAACCTGCTAACATATTCATCACAGAACATAGTGTTTGCAAAATTGGA

GACTTTGGATGCTCCCAAAAGCTAGAAGATAGTGAATCATCAGGACTACATCTTTGTCAT

CAAGGGGGAACATACACACATCGTGCTCCTGAACTTCTTAAAGGTGAGAAAATCACACCC

AA

>MZ712287_ZMMUR14165_[Pareas_berdmorei_unicolor]

GATCAGTTATGTCTCCTGCATCTCCTGGGCTCTGGAGGCTTTGGTTCTGTCTACAAGGCA

ACTTACCAAGGAGCTACAGTGGCGGTAAAACAGGTGAAGAGATGCAGTAAGAACCATTTG

GCATCACGGCAAAGCTTCTGGGCAGAGCTAAATGTAGCACGCCTTGACCATAACAATGTG

GTACACATAGTAGCTGCTAGCACATGTACCCCTGCTAGTCAGGATAGTCTGGGTACCATA

ATTATGGAATATGCAGGTAATTGCACTCTACATCATATTATCTATGGGACTGGCTATTTA

ACAGGAAATAACAATGATGGCCTTCAATGTGATTTTGAGTTCTTGACTACAGCTCAGGCT

GTCATTTACGCCTGTGATATTGTGGCGGGCTTAATGTTTCTCCATTCTCAGTTAATTGTG

CATCTGGATTTAAAACCTGCTAACATATTCATCACAGAACATAGTGTTTGCAAAATTGGA

GACTTTGGATGCTCCCAAAAGCTAGAAGATAGTGAATCATCAGGACTACATCTTTGTCAT

CAAGGGGGAACATACACACATCGTGCTCCTGAACTTCTTAAAGGTGAGAAAATCACACCC

AA

>MZ712288_ZMMUR16801_[Pareas_berdmorei_truongsonicus]

GATCAGTTATGTCTCCTGCATCTCCTGGGCTCTGGGGGCTTTGGTTCTGTCTACAAGGCA

ACTTACCAAGGAGCTACAGTGGCGGTAAAACAGGTGAAGAGATGCAGTAAGAACCATTTG

GCATCACGGCAAAGCTTCTGGGCAGAGCTAAATGTAGCACGCCTTGACCATAACAATGTG

GTACACATAGTAGCTGCTAGCACATGTACCCCTGCTAGTCAGGATAGTCTGGGTACCATA

ATTATGGAATATGCAGGTAATTGCACTCTACATCATATTATCTATGGGACTGGCTATTTA

ACAGGAAATAACAATGATGGCCTTCAATGTGATTTTGAGTTCTTGACTACAGCTCAGGCT

GTCATTTACGCCTGTGATATTGTGGCGGGCTTAATGTTTCTCCATTCTCAGTTAATTGTG

CATCTGGATTTAAAACCTGCTAACATATTCATCACAGAACATAGTGTTTGCAAAATTGGA

GACTTTGGATGCTCCCAAAAGCTAGAAGATAGTGAATCATCAGGACTACATCTTTGTCAT

CAAGGGGGAACATACACACATCGTGCTCCTGAACTTCTTAAAGGTGAGAAAATCACACCC

AA

>MZ712289_ZMMUR14796_[Pareas_berdmorei_truongsonicus]

GATCAGTTATGTCTCCTGCATCTCCTGGGCTCTGGGGGCTTTGGTTCTGTCTACAAGGCA

ACTTACCAAGGAGCTACAGTGGCGGTAAAACAGGTGAAGAGATGCAGTAAGAACCATTTG

GCATCACGGCAAAGCTTCTGGGCAGAGCTAAATGTAGCACGCCTTGACCATAACAATGTG

GTACACATAGTAGCTGCTAGCACATGTACCCCTGCTAGTCAGGATAGTCTGGGTACCATA

ATTATGGAATATGCAGGTAATTGCACTCTACATCATATTATCTATGGGACTGGCTATTTA

ACAGGAAATAACAATGATGGCCTTCAATGTGATTTTGAGTTCTTGACTACAGCTCAGGCT

GTCATTTACGCCTGTGATATTGTGGCGGGCTTAATGTTTCTCCATTCTCAGTTAATTGTG

CATCTGGATTTAAAACCTGCTAACATATTCATCACAGAACATAGTGTTTGCAAAATTGGA

GACTTTGGATGCTCCCAAAAGCTAGAAGATAGTGAATCATCAGGACTACATCTTTGTCAT

CAAGGGGGAACATACACACATCGTGCTCCTGAACTTCTTAAAGGTGAGAAAATCACACCC

AA

>MZ712290_ZMMUR16800_[Pareas_carinatus_tenasserimicus]

CTATGGACTTGGGATCAGTTATGTCTCCTGCATCTCCTGGGCTCTGGGGGCTTTGGTTCT

GTCTACAAGGCAACTTACCAAGGAGCTACAGTGGCTGTAAAACAGGTGAAGAGATGCAGT

AAGAACCATTTGGCATCACGGCAAAGCTTCTGGGCAGAACTAAATGTAGCACGCCTTGAC

CATAACAATGTGGTACACATAGTAGCTGCTAGCACATGTACCCCTGCTAGTCAGGATAGT

TTGGGTACCATAATTATGGAATATGCAGGTAATTGCACTCTACATCATATTATCTATGGG

ACTGGCTATTTAACAGGAAATAACAATGATGGCCTTCAATGTGATTTTGAGTTCTTGACT

ACAGCTCAGGCTGTCATTTACGCCTGTGATATTGTGGCGGGCTTAATGTTTCTCCATTCT

CAGTTAATTGTGCATCTGGATTTAAAACCTGCTAACATATTCATCACAGAACATAGTGTT

TGCAAAATTGGAGACTTTGGATGCTCCCAAAAGCTAGAAGATAGTGAATCATCAGGACTA

CATCTTTGTCATCAAGGGGGAACATACACACATCGTGCTCCTGAACTTCTTAAAGGTGAG

AAAATCCAAACCCA

>MZ712291_CAS247982_[Pareas_carinatus_tenasserimicus]

GATCAGTTATGTCTCCTGCATCTCCTGGGCTCTGGGGGCTTTGGTTCTGTCTACAAGGCA

ACTTACCAAGGAGCTACAGTGGCTGTAAAACAGGTGAAGAGATGCAGTAAGAACCATTTG

GCATCACGGCAAAGCTTCTGGGCAGAACTAAATGTAGCACGCCTTGACCATAACAATGTG

GTACACATAGTAGCTGCTAGCACATGTACCCCTGCTAGTCAGGATAGTTTGGGTACCATA

ATTATGGAATATGCAGGTAATTGCACTCTACATCATATTATCTATGGGACTGGCTATTTA

ACAGGAAATAACAATGATGGCCTTCAATGTGATTTTGAGTTCTTGACTACAGCTCAGGCT

GTCATTTACGCCTGTGATATTGTGGCGGGCTTAATGTTTCTCCATTCTCAGTTAATTGTG

CATCTGGATTTAAAACCTGCTAACATATTCATCACAGAACATAGTGTTTGCAAAATTGGA

GACTTTGGATGCTCCCAAAAGCTAGAAGATAGTGAATCATCAGGACTACATCTTTGTCAT

CAAGGGGGAACATACACACATCGTGCTCCTGAACTTCTTAAAGGTGAGAAAATCACACCC

AA

>MZ712292_LSUHC10604_[Pareas_carinatus_carinatus]

GATCAGTTATGTCTCCTGCATCTCCTGGGCTCTGGGGGCTTTGGTTCTGTCTACAAGGCA

ACTTACCAAGGAGCTACAGTGGCTGTAAAACAGGTGAAGAGATGCAGTAAGAACCATTTG

GCATCACGGCAAAGCTTCTGGGCAGAACTAAATGTAGCACGCCTTGACCATAACAATGTG

GTACACATAGTAGCTGCTAGCACATGTACCCCTGCTAGTCAGGATAGTTTGGGTACCATA

ATTATGGAATATGCAGGTAATTGCACTCTACATCATATTATCTATGGGACTGGCTATTTA

ACAGGAAATAACAATGATGGCCTTCAATGTGATTTTGAGTTCTTGACTACAGCTCAGGCT

GTCATTTACGCCTGTGATATTGTGGCGGGCTTAATGTTTCTCCATTCTCAGTTAATTGTG

CATCTGGATTTAAAACCTGCTAACATATTCATCACAGAACATAGTGTTTGCAAAATTGGA

GACTTTGGATGCTCCCAAAAGCTAGAAGATAGTGAATCATCAGGACTACATCTTTGTCAT

CAAGGGGGAACATACACACATCGTGCTCCTGAACTTCTTAAAGGTGAGAAAATCACACCC

AA

>MZ712293_ZMMUR16393_[Pareas_abros]

GATCAGTTATGTCTCCTGCATCTCCTGGGCTCTGGAGGCTTTGGTTCTGTCTACAAGGCA

ACTTACCAAGGAGCTACAGTGGCTGTAAAACAAGTGAAGAGATGCAGTAAGAACCATTTG

GCATCACGGCAAAGCTTCTGGGCAGAACTAAATGTAGCACGCCTTGACCATAACAATGTG

GTACACATAGTAGCTGCTAGCACATGTACCCCTACTAGTCAGGATAGTCTGGGTACCATA

ATTATGGAATATGCAGGTAATTGCACTCTACATCATATTATCTATGGGATTGGCTATTTA

ACAGGAAATAACAATGATGGCCTTCAATGTGATCTTGAGTTCTTGACTACAGCTCAGGCT

GTCATTTACGCCTGTGATATTGTGGCAGGCTTAATGTTTCTCCATTCTCAGTTAATTGTG

CATCTGGATTTAAAACCTGCTAACATATTCATCACAGAACATAGTGTTTGCAAAATTGGA

GACTTTGGATGCTCCCAAAAGCTAGAAGATAGTGAATCATCAGGACTACATCTTTGTCAT

CAAGGGGGAACATACACACATCGTGCTCCTGAACTTCTTAAAGGTGAGAAAATCACACCC

AA

>MZ712294_ZMMUR16392_[Pareas_abros]

GATCAGTTATGTCTCCTGCATCTCCTGGGCTCTGGAGGCTTTGGTTCTGTCTACAAGGCA

ACTTACCAAGGAGCTACAGTGGCTGTAAAACAAGTGAAGAGATGCAGTAAGAACCATTTG

GCATCACGGCAAAGCTTCTGGGCAGAACTAAATGTAGCACGCCTTGACCATAACAATGTG

GTACACATAGTAGCTGCTAGCACATGTACCCCTACTAGTCAGGATAGTCTGGGTACCATA

ATTATGGAATATGCAGGTAATTGCACTCTACATCATATTATCTATGGGATTGGCTATTTA

ACAGGAAATAACAATGATGGCCTTCAATGTGATCTTGAGTTCTTGACTACAGCTCAGGCT

GTCATTTACGCCTGTGATATTGTGGCAGGCTTAATGTTTCTCCATTCTCAGTTAATTGTG

CATCTGGATTTAAAACCTGCTAACATATTCATCACAGAACATAGTGTTTGCAAAATTGGA

GACTTTGGATGCTCCCAAAAGCTAGAAGATAGTGAATCATCAGGACTACATCTTTGTCAT

CAAGGGGGAACATACACACATCGTGCTCCTGAACTTCTTAAAGGTGAGAAAATCACACCC

AA

>MZ712295_ZMMUR14788_[Pareas_abros]

GATCAGTTATGTCTCCTGCATCTCCTGGGCTCTGGAGGCTTTGGTTCTGTCTACAAGGCA

ACTTACCAAGGAGCTACAGTGGCTGTAAAACAAGTGAAGAGATGCAGTAAGAACCATTTG

GCATCACGGCAAAGCTTCTGGGCAGAACTAAATGTAGCACGCCTTGACCATAACAATGTG

GTACACATAGTAGCTGCTAGCACATGTACCCCTACTAGTCAGGATAGTCTGGGTACCATA

ATTATGGAATATGCAGGTAATTGCACTCTACATCATATTATCTATGGGATTGGCTATTTA

ACAGGAAATAACAATGATGGCCTTCAATGTGATCTTGAGTTCTTGACTACAGCTCAGGCT

GTCATTTACGCCTGTGATATTGTGGCAGGCTTAATGTTTCTCCATTCTCAGTTAATTGTG

CATCTGGATTTAAAACCTGCTAACATATTCATCACAGAACATAGTGTTTGCAAAATTGGA

GACTTTGGATGCTCCCAAAAGCTAGAAGATAGTGAATCATCAGGACTACATCTTTGTCAT

CAAGGGGGAACATACACACATCGTGCTCCTGAACTTCTTAAAGGTGAGAAAATCACACCC

AA

>MZ712296_ZMMUR16802_[Pareas_kuznetsovorum]

CTCATGGACTGGGATCAGTTATGTCTCCTGCATCTCCTGGGCTCTGGGGGCTTTGGTTCT

GTCTACAAGGCAACTTACCAAGGAGCTACAGTGGCTGTAAAACAGGTGAAGAGATGCAGT

AAGAACCATTTGGCATCACGGCAAAGCTTCTGGGCAGAACTAAATGTAGCACGCCTTGAC

CATAACAATGTGGTACACATAGTAGCTGCTAGCACATGTACCCCTGCTAGTCAGGATAGT

CTGGGTACCATAATTATGGAATATGCAGGTAATTGCACTCTACATCATATTATCTATGGG

ACTGGCTATTTAACAGGAAATAACAATGATGGCCTTCAATGTGATTTTGAGTTCTTGACT

ACAGCTCAGGCTGTCATTTACGCCTGTGATATTGTGGCGGGCTTAATGTTTCTCCATTCT

CAGTTAATTGTGCATCTGGATTTAAAACCTGCTAACATATTCATCACAGAACATAGTGTT

TGCAAAATTGGAGACTTTGGATGCTCCCAAAAGCTAGAAGATAGTGAATCATCAGGACTA

CATCTTTGTCATCAAGGGGGAACATACACACATCGTGCTCCTGAACTTCTTAAAGGTGAG

AAAATCACACCCAA

>MZ712297_ZMMUR13656_[Pareas_temporalis]

GAGCAGTTATGTTTCCTGCATCTCCTGGGCTCTGGGGGCTTTGGTTCTGTCTACAAGGCA

ACTTACCAAGGAGCTACAGTGGCTGTAAAACAAGTGAAGAGATGCAGTAAGAACCATTTG

GCATCACGGCAAAGCTTCTGGGCAGAACTAAATGTAGCACGCCTTGACCATAACAATGTG

GTACACATAGTAGCTGCTAGCACATGTACCCCTGCTAGTCAGGATAGTCTGGGTACCATA

ATTATGGAATATGCAGGTAATTGCACTCTACATCATATTATCTATGGGATTGGCTATTTA

ACAGGAAATAACAATGATGGCCTTCAATGTGATCTTGAGTTCTTGACTACAGCTCAGGCT

GTCATTTACGCCTGTGATATTGTGGCAGGCTTAATGTTTCTCCATTCTCAGTTAATTGTG

CATCTGGATTTAAAACCTGCTAACATATTCATCACAGAACATAGTGTTTGCAAAATTGGA

GACTTTGGATGCTCCCAAAAGCTAGAAGATAGTGAATCATCAGGACTACATCTTTGTCAT

CAAGGGGGAACATACACACATCGTGCTCCTGAACTTCTTAAAGGTGAGAAAATCACAACC

CA

>MZ712298_SIEZC20215_[Pareas_temporalis]

GAGCAGTTATGTTTCCTGCATCTCCTGGGCTCTGGGGGCTTTGGTTCTGTCTACAAGGCA

ACTTACCAAGGAGCTACAGTGGCTGTAAAACAAGTGAAGAGATGCAGTAAGAACCATTTG

GCATCACGGCAAAGCTTCTGGGCAGAACTAAATGTAGCACGCCTTGACCATAACAATGTG

GTACACATAGTAGCTGCTAGCACATGTACCCCTGCTAGTCAGGATAGTCTGGGTACCATA

ATTATGGAATATGCAGGTAATTGCACTCTACATCATATTATCTATGGGATTGGCTATTTA

ACAGGAAATAACAATGATGGCCTTCAATGTGATCTTGAGTTCTTGACTACAGCTCAGGCT

GTCATTTACGCCTGTGATATTGTGGCAGGCTTAATGTTTCTCCATTCTCAGTTAATTGTG

CATCTGGATTTAAAACCTGCTAACATATTCATCACAGAACATAGTGTTTGCAAAATTGGA

GACTTTGGATGCTCCCAAAAGCTAGAAGATAGTGAATCATCAGGACTACATCTTTGTCAT

CAAGGGGGAACATACACACATCGTGCTCCTGAACTTCTTAAAGGTGAGAAAATCACAACC

CA

>MZ712299_LSUHC9098_[Asthenodipsas_lasgalenensis]

AGCTGCTCTGGGGGCTTTGGTTCTGTCTACAAGGCAACTTACCAAGGAGCTATAGTTGCT

GTAAAACAAGTGAAGAGATGCAGTAAGAACCATTTGGCATCCCGGCAAAGCTTCTGGGCT

GAACTAAATGTAGCACGTCTTGACCATAACAATGTGGTACGCATAGTAGCTGCTAGCACA

TGTACCCCTGCTAGTCAGGATAGTTTGGGTACCATAATTATGGAATATGCAGGTCATTGC

ACTCTACATCATATTATCTATTGGACTGGCTATTTAACAGATAATAACAACGATGGCCTT

CAATGTGATCATGAGTTTTTGAGTACAGCTCAGGCTGTCATTTACGCCTGTGATATTGTG

GCAGGGTTAATGTTTCTCCATTCTCAGTTAATCGTGCATCTGGATTTAAAACCTGCTAAC

ATATTCATCACAGAACATAATGTTTGCAAAATTGGAGACTTTGGATCCTCCCAAAAGCTG

GAAGATAGTGAATCATCAGGACTACATCTTTATCATCAAGGGGGAACATACACACATCGT

GCTCCTGAACTTCTTAAAGGTGAGAAAACCCCCCCCAA

>MZ712300_Avpt_[Asthenodipsas_vertebralis]

AGCTGCTCTGGGGGCTTTGGTTCTGTCTACAAGGCAACTTACCAAGGAGCTACAGTTGCT

GTAAAACAAGTGAAGAGATGCAGTAAGAACCATTTGGCATCCCGGCAAAGCTTCTGGGCT

GAACTAAATGTAGCACGTCTTGACCATAACAATGTGGTACGCATAGTAGCTGCTAGCACA

TGTACCCCTGCTAGTCAGGATAGTTTGGGTACCATAATTATGGAATATGCAGGTCATTGC

ACTCTACATCATATTATCTATTGGACTGGCTATTTAACAGATAATAACAACGATGGCCTT

CAATGTGATCATGAGTTTTTGAGTACAGCTCAGGCTGTCATTTACGCCTGTGATATTGTG

GCAGGGTTAATGTTTCTCCATTCTCAGTTAATCGTGCATCTGGATTTAAAACCTGCTAAC

ATATTCATCACAGAACATAATGTTTGCAAAATTGGAGACTTTGGATCCTCCCAAAAGCTG

GAAGATAGTGAATCATCAGGACTACATCTTTATCATCAAGGGGGAACATACACACATCGT

GCTCCTGAACTTCTTAAAGGTGAGAAAACCCCCCCCAA

***RAG-1* nuDNA gene**

>MZ712301_CAS235254_[Pareas_victorianus]

GACAGCCACCAGGCTAACTCTGCTAAAGAAAATGCAACAGGTTCTATGGACGTCACTGAT

GAACAACCTAAGGCAGCAGCTATAATGTCACAAGTGCCCTTTGAAACAGGCACTGAGTTG

AATAAAAATAGCTTGGCAAGAGAGAAAGATGTCTTTCACATGAACCAAAGGGAGGTGGAA

GCTCACCAAGCAAATCTGCAGTGCCTCTGTCGCATCTGTGGAGGCTCATTGAAAACTGAT

CCTTGCAAAAAGTTTCATCCAGTTCATGGACCAGTGGACGAGGAGACCCAGGCTCTTTTG

AGAAAGAAAGAAAAAAGAGCCACTTCTTGGCCAGATCTTCTTGCCAAAGTTTTTAAGATT

GATGTACGGGGAGATATTGACACAACCCATCCTACTCGTTTTTGTCACAATTGTTGGAAA

GTGATTCAAAGAAAAGTCAGCAATGCCCCCCATGAAGCAGATCTTCTAGGGAAAGACCCA

GTGGAGTGGCGGTCCCATTCAACAAGCTGTGACATTTGTGTCACTTCCTTTCGTGGAGTC

AAGAGAAAGAAACCAATGCTGAATTCACAGCTGAGCAAAAAACTCAGGATTATTGCTGGA

CGTGCGAGAAAAATAAGATGCATAAGAAAGGTAAAGCAGCTGAAAAACAAAAGTTTAATG

AAAAAGATTTCAAACTGTAAGCAAATCCATCTCAGTACAAAAGCCCTTGCAATAGATTAT

CCTGTGGACTTTGTAAAGTCAATATCTTGCCAGATCTGTGAACACATCCTGTCCGATCCA

GTAGAAACAACATGTAAACACTTATTCTGTAGAGTTTGCATTCTTAAATGCCTTAAAGTA

ATGGGAAGCTATTGCCCATCCTGCCAATATCCTTGCTTTCCTACTGATCTAGTGAGTCCT

GTAAAATCCTTCCTAAACATCCTCAACAATTTGGTTTTGAGATGCCCAATAAAAGGCTGT

CATGAGGATGTCTTTTTGGAAAAATACTGCCAACATCGTTCTAACCATAAAGAGGCGGAA

AGC

>MZ712302_ZMMUR16631_[Pareas_monticola]

GACAGCCACCAGGCTAACTCTGCTAAAGAAAATGCAACAAGTTCTATGGACGTCACTGAT

GAACAACCTAGGGCAGCAGCTGTAATGTCACAAGTGCCCTTTGAAACAGGCACTGAGTTG

AATAAAAATAGCTTGGCAAGAGAGAAAGATGTCTTTCACATGAGCCAAAGGGAGGTGGAA

GCTCACCAAGCAAATCTGCAGTGCCTCTGTCGCATCTGTGGAGGCTCACTGAAAACTGAT

CCTTGCAAAAAGTTTCATCCAGTTCATGGACCAGTGGACGAGGAGACCCAGGCTCTTTTG

AGAAAGAAAGAAAAAAGAGCCACTTCTTGGCCAGATCTTCTTGCCAAAGTTTTTAAGATT

GATGTACGGGGAGATATTGACACAACCCATCCGACTCGTTTTTGTCACAATTGTTGGAAA

GTGATTCAAAGAAAAGTCAGCAATGCCCCCCATGAAGCACATCTTCTAGGGAAAGACCCA

GTGGAGTGGCGGTCCCATTCAACAAGCTGTGACATTTGTGTCACTTCCTTTCGTGGAGTC

AAGAGAAAGAAACCAATGCTGAATTCACAGCTGAGCAAAAAACTCAGGATTATTGCTGGA

CGTACGAGAAAAATAAGATGCATAAGAAAGGTAAAGCAGCTGAAAAACAAAAGTTTAATG

AAAAAGATTTCAAACTGTAAGCAAATCCATCTCAGTACAAAAGCCCTTGCAATAGATTAT

CCTGTGGACTTTGTAAAGTCAATATCTTGCCAGATCTGTGAACACATCTTGTCCGATCCA

GTAGAAACAACATGTAAACACTTATTCTGTAGAGTTTGCATTCTTAAATGCCTTAAAGTA

ATGGGAAGCTATTGCCCATCCTGCCAATATCCTTGCTTTCCTACTGATCTAGTGAGTCCT

GTAAAATCCTTCCTAAACATCCTCAACAATTTGGTTTTGAGATGCCCAATAAAAGGCTGT

CATGAGGATGTCGTTTTGGAAAAATACTGCCAACATCGTTCTAACCATAAAGAGGTGGAA

AGC

>MZ712303_CAS235359_[Pareas_andersonii]

GACAGCCACCAGGCTAACTCTGCGAAAGAAAATGCAACAGGTTCTATGGACGTCACTGAT

GAACAACCTAAGGCAGCAGCTGTAATGTCACAAGTGCCCTTTGAAACAGGCACTGAGTTG

AATAAAAATAGCTTGGCAAGAGAGAAAGATGACTTTCACATGAGCCAAAGGGAGGTGGAA

GCTCACCAAGCAAATCTGCAGTGCCTCTGTCGCATCTGTGGAAGCTCACTGAAAACTGAC

CCTTGCAAAAAGTGTCATCCAGTTCATGGACCAGTGGACGAGGAGACCCAGGCTCTTTTG

AGAAAGAAAGAAAAAAGAGCCACTTCTTGGCCAGATCTTCTTGCCAAAGTTTTTAAGATT

GATGTACGAGGAGATATCGACACAACCCATCCTACTCGTTTTTGTCACAATTGTTGGAAA

GTGATTCAAAGAAAAGTCAGCAATGCCCCCCATGAAGCACATCTTCTAGGGAAAGACCCA

GTGGAGTGGCAGTCCCATTCAACAAGCTGTGACATTTGTGTCACTTCCTTTCGTGGAGTC

AAGAGAAAGAAACCCATGCTGAATTCACAGCTGAGCAAAAAACTTCGGATTATTGCTGGA

CGTACGAGAAAAATAAGATGCATAAGAAAGGTAAAGCAGCTGAAAAACAAAAGTTTAATG

AAAAAGATTTCAAACTGTAAGCAAATCCATCTCAGTACAAAATCCCTTGCAATAGATTAT

CCTATGGACTTTGTAAAGTCAATATCTTGCCAGATCTGTGAACACATCCTGTCCGATCCA

GTAGAAACAACATGTAAACACTTGTTCTGTAGAGTCTGCATTCTTAAATGCCTTAATGTA

ATGGGAAGCTATTGCCCATCCTGCCAATATCCTTGCTTTCCTACTGATCTAGTGAGTCCT

GTAAAATCCTTCCTAAACATCCTCAACAATTTGGTTTTGAGATGCCCAATAAAAGGCTGT

CATGAGGATGTCTTTTTGGAAAAATACTGCCAACATCGTTCTAATCATAAAGAGGCGGAA

AGC

>MZ712304_ZMMUR13451_[Pareas_margaritophorus]

GACAGCCACCAGGCTAACTCTGCTAAAGAAAATGCAACAGGTTCTATGGACGTCACTGAT

GAACAACCTAAGGCAGCAGCTGTAATGTCACAAGTGCCCTTTGAAACAGGCACTGAGTTG

AATAAAAATAGCTTGGCAAGAGAGAAAGATGTCTTTCACATGAGCCAAAGGGAGGTGGAA

GCTCACCAAGCAAATCTGCAGTGCCTCTGTCGCATCTGTGGAAGCTCACTGAAAACTGAT

CCTTGCGCAAAGTGTCATCCAGTCCATGGACCAGTGGACGAGGAGACCCAGGCTCTTTTG

AGAAAGAAAGAAAAAAGAGCCACTTCTTGGCCAGATCTTCTTGCCAAAGTTTTTAAGATT

GATGTACGGGGAGATATCGACACAACCCATCCTACTCGTTTTTGTCACAATTGTTGGAAA

GTGATTCAAAGAAAAGTCAGCAATGCCCCCCATGAAGCACATCTTCTAGGGAAAGACCCA

GTGGAGTGGCGGTCCCATTCAACAAGCTGTGACGTTTGTGTCATTTCCTTTCGTGGAGTC

AAGAGAAAGAAACCCATGTTGAATTCACAGCTGAGCAAAAAACTCCGGATTATTGCTGGA

CGTACGAGAAAAATAAGATGCATAAGAAAGGTAAAGCAGCTGAAAAACAAAAGTTTAATG

AAAAAGATTTCGAACTGTAAGCAAATCCATCTCAGTACAAAATCCCTTGCAATAGATTAT

CCTGTGGACTTTGTAAAGTCAATATCTTGCCAGATCTGTGAACACATCCTGTCCGATCCA

GTAGAAACAACATGTAAACACTTTTTCTGTAGAGTCTGCATTCTTAAATGCCTTAATGTA

ATGGGAAGCTATTGCCCATCCTGCCAATATCCTTGCTTTTCTACTGATCTAGTGAGTCCT

GTAAAATCCTTCCTAAACATCCTCAACAATTTGGTTTTGAGATGCCCAATAAAAGGCTGT

CATGAGGATGTCTTTTTGGAAAAATACTGCCAACATCGTTCTAATCATAAAGAGGCGGAA

AGC

>MZ712305_ZMMUNAP09759_[Pareas_margaritophorus]

GACAGCCACCAGGCTAACTCTGCGAAAGAAAATGCAGCAGGTTCTATGGACGTCACTGAT

GAACAACCTAAGGCAGCAGCTGTAATGTCACAAGTGCCCTTTGAAACAGGCACTGAGTTG

AATAAAAATAGCTTGGCAAGAGAGAAAGATGTCTTTCACATGAGCCAAAGGGAGGTGGAA

GCTCACCAAGCAAATCTGCAGTGCCTCTGTCGCATCTGTGGAAGCTCACTGAAAACTGAT

CCTTGCGCAAAGTGTCATCCAGTTCATGGACCAGTGGACGAGGAGACCCAGGCTCTTTTG

AGAAAGAAAGAAAAAAGAGCCACTTCTTGGCCAGATCTTCTTGCCAAAGTTTTTAAGATT

GATGTACGAGGAGATATCGACACAACCCATCCTACTCGTTTTTGTCACAATTGCTGGAAA

GTGATTCAAAGAAAAGTCAGCAATGCCCCCCATGAAGCACATCTTCTAGGGAAAGACCCA

GTGGAGTGGCGGTCCCATTCAACAAGCTGTGACGTTTGTGTCACTTCCTTTCGTGGAGTC

AAGAGAAAGAAACCCATGCTGAATTCACAGCTGAGCAAAAAACTCCGGATTATTGCTGGA

CGTACGAGAAAAATAAGATGCATAAGAAAGGTAAAGCAGCTGAAAAACAAAAGTTTAATG

AAAAAGATTTCGAACTGTAAGCAAATCCATCTCAGTACAAAATCCCTTGCAATAGATTAT

CCTGTGGACTTTGTACAGTCAATATCTTGCCAGATCTGTGAACACATCCTGTCCGATCCA

GTAGAAACAACATGTAAACACTTGTTCTGTAGAGTCTGCATTCTTAAATGCCTTAATGTA

ATGGGAAGCTATTGCCCATCCTGCCAATATCCTTGCTTTTCTACTGATCTAGTGAGTCCT

GTAAAATCCTTCCTAAACATCCTCAACAATTTGGTTTTGAGATGCCCAATAAAAGGCTGT

CATGAGGATGTCTTTTTGGAAAAAATACTGCCAACATCGTTCTAATCATAAAGAGGCGGAA

AGC

>MZ712306_ZMMUR16628_[Pareas_macularius]

GACAGCCACCAGGCTAACTCTGCGAAAGAAAATCCAGCAGGTTCTATGGACGTCACTGAT

GAACAACCTAAGGCAGCAGCTGTAATGTCACAAGTGCCCTTTGAAACAGGCACTGAGTTG

AATAAAAATAGCTTGGCAAGAGAGAAAGATGTCTTTCACATGAGCCAACGGGAGGTGGAA

GCTCACCAAGCAAATCTGCAGTGCCTCTGTCGCATCTGTGGAAGCTCACTGAAAACTGAT

CCTTGCAAAAAGTGTCATCCAGTTCATGGACCAGTGGACGAGGAGACCCAGGCTCTTTTG

AGAAAGAAAGAAAAAAGAGCCACTTCTTGGCCAGATCTTCTTGCCAAAGTTTTTAAGATT

GATGTACGAGGAGATATTGACACAACCCATCCTACTCGTTTTTGTCACAATTGTTGGAAA

GTGATTCAAAGAAAAGTCAGCAATGCCCCCCATGAAGCACATCTTCTAGGGAAAGACCCA

GTGGAGTGGCGGTCCCATTCAACAAGCTGTGACGTTTGTGTTACTTCCTTTCGTGGAGTC

AAGAGAAAGAAACCCATGCTGAATTCACAGCTGAGCAAAAAACTCCGGATTATTGCTGGA

CGTACGAGAAAAATAAGATGCATAAGAAAGGTAAAGCAGCTGAAAAACAAAAGTTTAATG

AAAAAGATTTCAAACTGTAAGCAAATCCATCTCAGTACAAAATCCCTTGCAATAGATTAT

CCTGTGGACTTTGTAAAGTCAATATCTTGCCAGATCTGTGAACACATCCTGTCCGATCCA

GTAGAAACAACATGTAAACACTTGTTCTGTAAAGTCTGCATTCTTAAATGCCTTAATGTA

ATGGGAAGCTATTGCCCATCCTGCCAATATCCTTGCTTTCCTACTGATCTAGTGAGTCCT

GTAAAATCCTTCCTAAACATCCTCAACAATTTGGTTTTGAGATGCCCAATAAAAGGCTGT

CATGAGGATGTCTTTTTGGAAAAAATACTGCCAACATCGTTCTAATCATAAAGAGGCGGAA

AGC

>MZ712307_ZMMUR16629_[Pareas_macularius]

GACAGCCACCAGGCTAACTCTGCGAAAGAAAATGCAACAGGTTCTATGGACGTCACTGAT

GAACAACCTAAGGCAGCAGCTGTAATGTCACAAGTGCCCTTTGAAACAGGCACTGAGTTG

AATAAAAATAGCTTGGCAAGAGAGAAAGATGTCTTTCACATGAGCCAAAGGGAGGTGGAA

GCTCACCAAGCAAATCTGCAGTGCCTCTGTCGCATCTGTGGAAGCTCACTGAAAACTGAT

CCTTGCAAAAAGTGTCATCCAGTTCATGGACCAGTGGACGAGGAGACCCAGGCTCTTTTG

AGAAAGAAAGAAAAAAGAGCCACTTCTTGGCCAGATCTTCTTGCCAAAGTTTTTAAGATT

GATGTACGAGGAGATATCGACACGACCCATCCTACTCGTTTTTGTCACAATTGTTGGAAA

GTGATTCAAAGAAAAGTCAGCAATGCCCCCCATGAAGCACATCTTCTAGGGAAAGACCCA

GTGGAGTGGCGGTCCCATTCAACAAGCTGTGACGTTTGTGTCACTTCCTTTCGTGGAGTC

AAGAGAAAGAAACCCATGCTGAATTCACAGCTGAGCAAAAAACTCCGGATTATTGCTGGA

CGTACGAGAAAAATGAGATGCATAAGAAAGGTAAAGCAGCTGAAAAACAAAAGTTTAATG

AAAAAGATTTCAAACTGTAAGCAAATCCATCTCAGTACAAAATCCCTTGCAATAGATTAT

CCTGTGGACTTTGTAAAGTCAATATCTTGCCAGATCTGTGAACACATTCTGTCCGATCCA

GTAGAAACAACATGTAAACACTTGTTCTGTAGAGTCTGCATTCTTAAATGCCTTAATGTA

ATGGGAAGCTATTGCCCATCCTGCCAATATCCTTGCTTTCCTACTGATCTAGTGAGTCCT

GTAAAATCCTTCCTAAACATCCTCAACAATTTGGTTTTGAGATGCCCAATAAAAGGCTGT

CATGAGGATGTCTTTTTGGAAAAATACTGCCAACATCGTTCTAATCATAAAGAGGCGGAA

AGC

>MZ712308_CAS248147_[Pareas_vindumi]

GACAGCCACCAGGCTAACTCTGCGAAAGAAAATCCAACAGGTTCTATGGACGTCACTGAT

GAACAACCTAAGGCAGCAGCTGTAATGTCACAAGCGCCCTTTGAAACAGGCACTGAGTTG

AATAAAAATAGCTTGGCAAGAGAGAAAGATGTCTTTCACATGAGCCGAAGGGAGGTGGAA

GCTCACCAAGCAAATCTGCAGTGCCTCTGTCGCATCTGTGGAGGCTCACTGAAAACTGAT

CCTTGCAAAAAGTGTCATCCAGTTCATGGACCAGTGGACGAGGAGACCCAGGCTCTTTTG

AGAAAGAAAGAAAAAAGAGCCACTTCTTGGCCAGATCTTCTTGCCAAAGTTTTTAAGATT

GATGTACGGGGAGATATCGACACAACCCATCCTACTCGTTTTTGTCACAATTGTTGGAAA

GTGATTCAAAGAAAAGTCAGCAATGCCCCCCATGAAGCACATCTTCTAGGGAAAGACCCA

GTGGAGTGGCGGTCCCATTCAACAAGCTGTGATGTTTGTGTCACTTCCTTTCGTGGAGTC

AAGAGAAAGAAACCCATGCTGAATTCACAGCTGAGCAAAAAACTCAGGATTAATGCTGGA

CGTACGAGAAAAATAAGATGCATAAGAAAGGTAAAGCAGCTGAAAAACAAAAGTTTAATG

AAAAAGATTTCAAACTGTAAGCAAATCCACCTCAGTACAAAATCCCTTGCAATAGATTAT

CCTGTGGACTTTGTAAAGTCAATATCTTGCCAGATCTGTGAACACATCCTGTCCGATCCA

GTAGAAACAACATGTAAACACTTATTCTGTAGAGTCTGCATTCTTAAATGCCTTAAAGTA

ATGGGAAGCTATTGCCCATCCTGCCAATATCCTTGCTTTCCTACTGATCTAGTGACTCCT

GTAAAATCCTTCCTAAACATCCTGAACAATTTGGTTTTGAGATGCCCAATAAAAGGCTGT

CATGAGGATGTCTTTTTGGAAAAATACTGCCAACATCGTTCTAATCATAAAGAGGCGGAA

AGC

>MZ712309_ZMMUNAP09088_[Pareas_hamptoni]

GACAGCCACCAGGCTAACTCTGCGAAAGAAAATCCAGCAGGTTCTATGGACGTCACTGAT

GAACAACCTAAGGCAGCAGCTGTAATGTCACAAGCGCCCTTTGAAACAGGCACTGAGTTG

AATAAAAATAGCTTGGCAAGAGAGAAAGATGTCTTTCACATGAGCCGAAGGGAGGTGGAA

GCTCACCAAGCAAATCTGCAGTGCCTCTGTCGCATCTGTGGAGGCTCCCTGAAAACTGAT

CCTTGCAAAAAGTGTCATCCAGTTCATGGACCAGTGGACGAGGAGACCCAGGCTCTTTTG

AGAAAGAAAGAAAAAAGAGCCACTTCTTGGCCAGATCTTCTTGCCAAAGTTTTTAAGATT

GATGTACGGGGAGATATCGACACAACCCATCCTACTCGTTTTTGTCACAATTGTTGGAAA

GTGATTCAAAGAAAAGTCAGCAATGCCCCCCATGAAGCACATCTTCTAGGGAAAGACCCA

GTGGAGTGGCGGTCTCATTCAACAAGCTGTGATGTTTGTGTCACTTCCTTTCGTGGAGTC

AAGAGAAAGAAACCCATGATGAATTCACAGCTGAGCAAAAAACTCAGGATTATTGCTGGA

CGTACGAGAAAAATAAGATGCATAAGAAAGGTAAAGCAGCTGAAAAACAAAAGTTTAATG

AAAAAGATTTCAAACTGTAAGCAAATCCATCTCAGTACAAAATCCCTTGCAATAGATTAT

CCTGTGGACTTTGTAAAGTCAATATCTTGCCAGATCTGTGAACACATCCTGTCCGATCCA

GTAGAAACTACATGTAAACACTTATTCTGTAGAGTTTGCATTCTTAAATGCCTTAAAGTA

ATGGGAAGCTATTGCCCATCCTGCCAATATCCTTGCTTTCCTACTGATCTAGTGAGTCCT

GTAAAATCCTTCCTAAACATCCTCAACAATTTGGTTTTGAGATGCCCAATAAAAGGCTGT

CATGAGGATGTCTTTTTGGAAAAATACTGCCAACATCGTTCTAATCATAAAGAGGTGGAA

AGC

>MZ712310_ZMMUR16478_[Pareas_geminatus]

GACAGCCACCAGGCTAACTCTGCGAAAGAAAATGCAACAGGTTCTATGGACGTCACTGAT

GAACAACCTAAGGCAGCAGCTGTAATGTCACAAGCGCCCTTTGAAACAGGCACTGAGTTG

AATAAAAATAGCTTGGCAAGAGAGAAAGATGTCTTTCACATGAGCCGAAGGGAGGTGGAA

GCTCACCAAGCAAATCTGCAGTGCCTCTGTCGCATTTGTGGAGGCTCCCTGAAAACTGAT

CCTTGCAAAAAGTGTCATCCAGTTCATGGACCAGTGGATGAGGAGACCCAGGCTCTTTTG

AGAAAGAAAGAAAAAAGAGCCACTTCATGGCCAGATCTTCTTGCCAAAGTTTTTAAGATT

GATGTACGGGGAGATATCGACACAACCCATCCTACTCGTTTTTGTCACAATTGTTGGAAA

GTGATTCAAAGAAAAGTCAGCAATGCCCCCCATGAAGCACATCTTCTAGGGAAAGACCCA

GTGGAGTGGCGGTCTCATTCAACAAGCTGTGACGTTTGTGTCACTTCCTTTCGTGGAGTC
AAGAGAAAGAAACCCATGCTGAATTCACAGCTGAGCAAAAAACTCAGGATTATTGCTGGA
CGTACGAGAAAAATAAGATGCATAAGAAAGGTAAAGCAGCTGAAAAACAAAAGTTTAATG
AAAAAGATTTCAAACTGTAAGCAAATCCATCTCAGTACAAAATCCCTTGCAATAGATTAT
CCTGTGGACTTTGTAAAGTCAATATCTTGCCAGATCTGTGAACACATCCTGTCCGATCCA
GTAGAAACAACATGTAAACACTTATTCTGTAGAGTCTGCATTCTTAAATGCCTTAAAGTA
ATGGGAAGCTATTGCCCATCCTGCCAATATCCTTGCTTTCCTACTGATCTAGTGAGTCCT
GTAAAATCCTTCCTAAACATCCTCAACAATTTGGTTTTGAGATGCCCAATAAAAGGCTGT
CATGAGGATGTCTTTTTGGAAAAATACTGCCAACATCGTTCTAATCATAAAGAGGCGGAA
AGC
>MZ712311_ZMMUNAP09280_[Pareas_geminatus]
GACAGCCACCAGGCTAACTCTGCGAAAGAAAATGCAACAGGTTCTATGGACGTCACTGAT
GAACAACCTAAGGCAGCAGCTGTAATGTCACAAGCGCCCTTTGAAACAGGCACTGAGTTG
AATAAAAATAGCTTGGCAAGAGAGAAAGATGTCTTTCACATGAGCCGAAGGGAGGTGGAA
GCTCACCAAGCAAATCTGCAGTGCCTCTGTCGCATTTGTGGAGGCTCCCTGAAAACTGAT
CCTTGCAAAAAGTGTCATCCAGTTCATGGACCAGTGGATGAGGAGACCCAGGCTCTTTTG
AGAAAGAAAGAAAAAAGAGCCACTTCATGGCCAGATCTTCTTGCCAAAGTTTTTAAGATT
GATGTACGGGGAGATATCGACACAACCCATCCTACTCGTTTTTGTCACAATTGTTGGAAA
GTGATTCAAAGAAAAGTCAGCAATGCCCCCCATGAAGCACATCTTCTAGGGAAAGACCCA
GTGGAGTGGCGGTCTCATTCAACAAGCTGTGACGTTTGTGTCACTTCCTTTCGTGGAGTC
AAGAGAAAGAAACCCATGCTGAATTCACAGCTGAGCAAAAAACTCAGGATTATTGCTGGA
CGTACGAGAAAAATAAGATGCATAAGAAAGGTAAAGCAGCTGAAAAACAAAAGTTTAATG
AAAAAGATTTCAAACTGTAAGCAAATCCATCTCAGTACAAAATCCCTTGCAATAGATTAT
CCTGTGGACTTTGTAAAGTCAATATCTTGCCAGATCTGTGAACACATCCTGTCCGATCCA
GTAGAAACAACATGTAAACACTTATTCTGTAGAGTCTGCATTCTTAAATGCCTTAAAGTA
ATGGGAAGCTATTGCCCATCCTGCCAATATCCTTGCTTTCCTACTGATCTAGTGAGTCCT
GTAAAATCCTTCCTAAACATCCTCAACAATTTGGTTTTGAGATGCCCAATAAAAGGCTGT
CATGAGGATGTCTTTTTGGAAAAATACTGCCAACATCGTTCTAATCATAAAGAGGCGGAA
AGC

>MZ712312_FMNH255567_[Pareas_formosensis]

GACAGCCACCAGGCTAACTCTGCGAAAGAAAATCCAGCAGGTTCTATGGACATCACTGAT

GAACAACCTAAGGCAGCAGCTGTAATGTCACAAGCGCCCTTTGAAACAGGCACTGAGTTG

AATAAAAATAGCTTGGCAAGAGAGAAAGATGTCTTTCACATGAGCCGAAGGGAGGTGGAA

GCTCACCAAGCAAATCTGCAGTGCCTCTGTCGCATCTGTGGAGGCTCCCTGAAAACTGAT

CCTTGCAAAAAGTGTCATCCAGTTCATGGACCAGTGGACGAGGAGACCCAGGCTCTTTTG

AGAAAGAAAGAAAAAAGAGCCACTTCTTGGCCAGATCTTCTTGCCAAAGTTTTTAAGATT

GATGTACGGGAAGATATCGACACAACCCATCCTACTCGTTTTTGTCACAATTGTTGGAAA

GTGATTCAAAGAAAAGTCAGCAATGCCCCCCATGAAGCACATCTTCTAGGGAAAGACCCA

GTGGAGTGGCGGTCTCATTCAACAAGCTGTGACGTTTGTGTCACTTCCTTTCGTGGAGTC

AAGAGAAAGAAACCCATGATGAATTCACAGCTGAGCAAAAAACTCAGGATTATTGCTGGA

CGTACGAGAAAAATAAGATGCATAAGAAAGGTAAAGCAGCTGAAAAACAAAAGTTTAATG

AAAAAGATTTCAAACTGTAAGCAAATCCATCTCAGTACAAAATCCCTTGCAATAGATTAT

CCTGTGGACTTTGTAAAGTCAATATCTTGCCAGATTTGTGAACACATCCTGTCCGATCCA

GTAGAAACAACATGTAAACACTTATTCTGTAGAGTCTGCATTCTTAAATGCCTTAAAGTA

ATGGGAAGCTATTGCCCATCCTGCCAATATCCTTGCTTTCCTACTGATCTAGTGAGTCCT

GTAAAATCCTTCCTAAACATCCTCAACAATTTGGTTTTGAGATGCCCAATAAAAGGCTGT

CATGAGGATGTCTTTTTGGAAAAATACTGCCAACATCGTTCTAATCATAAAGAGGCGGAA

AGC

>MZ712313_ZMMUNAP07265_[Pareas_formosensis]

GACAGCCACCAGGCTAACTCTGCGAAAGAAAATCCAGCAGGTTCTATGGACATCACTGAT

GAACAACCTAAGGCAGCAGCTGTAATGTCACAAGCGCCCTTTGAAACAGGCACTGAGTTG

AATAAAAATAGCTTGGCAAGAGAGAAAGATGTCTTTCACATGAGCCGAAGGGAGGTGGAA

GCTCACCAAGCAAATCTGCAGTGCCTCTGTCGCATCTGTGGAGGCTCCCTGAAAACTGAT

CCTTGCAAAAAGTGTCATCCAGTTCATGGACCAGTGGACGAGGAGACCCAGGCTCTTTTG

AGAAAGAAAGAAAAAAGAGCCACTTCTTGGCCAGATCTTCTTGCCAAAGTTTTTAAGATT

GATGTACGGGAAGATATCGACACAACCCATCCTACTCGTTTTTGTCACAATTGTTGGAAA

GTGATTCAAAGAAAAGTCAGCAATGCCCCCCATGAAGCACATCTTCTAGGGAAAGACCCA

GTGGAGTGGCGGTCTCATTCAACAAGCTGTGACGTTTGTGTCACTTCCTTTCGTGGAGTC

AAGAGAAAGAAACCCATGATGAATTCACAGCTGAGCAAAAAACTCAGGATTATTGCTGGA

CGTACGAGAAAAATAAGATGCATAAGAAAGGTAAAGCAGCTGAAAAACAAAAGTTTAATG

AAAAAGATTTCAAACTGTAAGCAAATCCATCTCAGTACAAAATCCCTTGCAATAGATTAT

CCTGTGGACTTTGTAAAGTCAATATCTTGCCAGATTTGTGAACACATCCTGTCCGATCCA

GTAGAAACAACATGTAAACACTTATTCTGTAGAGTCTGCATTCTTAAATGCCTTAAAGTA

ATGGGAAGCTATTGCCCATCCTGCCAATATCCTTGCTTTCCTACTGATCTAGTGAGTCCT

GTAAAATCCTTCCTAAACATCCTCAACAATTTGGTTTTGAGATGCCCAATAAAAGGCTGT

CATGAGGATGTCTTTTTGGAAAAATACTGCCAACATCGTTCTAATCATAAAGAGGCGGAA

AGC

>MZ712314_NMNH05637_[Pareas_formosensis]

GACAGCCACCAGGCTAACTCTGCGAAAGAAAATCCAGCAGGTTCTATGGACATCACTGAT

GAACAACCTAAGGCAGCAGCTGTAATGTCACAAGCGCCCTTTGAAACAGGCACTGAGTTG

AATAAAAATAGCTTGGCAAGAGAGAAAGATGTCTTTCACATGAGCCGAAGGGAGGTGGAA

GCTCACCAAGCAAATCTGCAGTGCCTCTGTCGCATCTGTGGAGGCTCCCTGAAAACTGAT

CCTTGCAAAAAGTGTCATCCAGTTCATGGACCAGTGGACGAGGAGACCCAGGCTCTTTTG

AGAAAGAAAGAAAAAAGAGCCACTTCTTGGCCAGATCTTCTTGCCAAAGTTTTTAAGATT

GATGTACGGGAAGATATCGACACAACCCATCCTACTCGTTTTTGTCACAATTGTTGGAAA

GTGATTCAAAGAAAAGTCAGCAATGCCCCCCATGAAGCACATCTTCTAGGGAAAGACCCA

GTGGAGTGGCGGTCTCATTCAACAAGCTGTGACGTTTGTGTCACTTCCTTTCGTGGAGTC

AAGAGAAAGAAACCCATGATGAATTCACAGCTGAGCAAAAAACTCAGGATTATTGCTGGA

CGTACGAGAAAAATAAGATGCATAAGAAAGGTAAAGCAGCTGAAAAACAAAAGTTTAATG

AAAAAGATTTCAAACTGTAAGCAAATCCATCTCAGTACAAAATCCCTTGCAATAGATTAT

CCTGTGGACTTTGTAAAGTCAATATCTTGCCAGATTTGTGAACACATCCTGTCCGATCCA

GTAGAAACAACATGTAAACACTTATTCTGTAGAGTCTGCATTCTTAAATGCCTTAAAGTA

ATGGGAAGCTATTGCCCATCCTGCCAATATCCTTGCTTTCCTACTGATCTAGTGAGTCCT

GTAAAATCCTTCCTAAACATCCTCAACAATTTGGTTTTGAGATGCCCAATAAAAGGCTGT

CATGAGGATGTCTTTTTGGAAAAATACTGCCAACATCGTTCTAATCATAAAGAGGCGGAA

AGC

>MZ712315_ZMMUNAP08868_[Pareas_formosensis]

GACAGCCACCAGGCTAACTCTGCGAAAGAAAATCCAGCAGGTTCTATGGACGTCACTGAT

GAACAACCTAAGGCAGCAGCTGTAATGTCACAAGCGCCCTTTGAAACAGGCACTGAGTTG

AATAAAAATAGCTTGGCAAGAGAGAAAGATGTCTTTCACATGAGCCGAAGGGAGGTGGAA

GCTCACCAAGCAAATCTGCAGTGCCTCTGTCGCATCTGTGGAGGCTCCCTGAAAACTGAT

CCTTGCAAAAAGTGTCATCCAGTTCATGGACCAGTGGACGCGGAGACCCAGGCTCTTTTG

AGAAAGAAAGAAAAAAGAGCCACTTCTTGGCCAGATCTTCTTGCCAAAGTTTTTAAGATT

GATGTACGGGGAGATATCGACACAACCCATCCTACTCGTTTTTGTCACAATTGTTGGAAA

GTGATTCAAAGAAAAGTCAGCAATGCCCCCCATGAAGCACATCTTCTAGGGAAAGACCCA

GTGGAGTGGCGGTCTCATTCAACAAGCTGTGACGTTTGTGTCACTTCCTTTCGTGGAGTC

AAGAGAAAGAAACCCATGATGAATTCACAGCTGAGCAAAAAACTCAGGATTATTGCTGGA

CGTACGAGAAAAATAAGATGCATAAGAAAGGTAAAGCAGCTGAAAAACAAAAGTTTAATG

AAAAAGATTTCAAACTGTAAGCAAATCCATCTCAGTACAAAATCCCTTGCAATAGATTAT

CCTGTGGACTTTGTAAAGTCAATATCTTGCCAGATCTGTGAACACATCCTGTCCGATCCA

GTAGAAACAACATGTAAACACTTATTCTGTAGAGTCTGCATTCTTAAATGCCTTAAAGTA

ATGGGAAGCTATTGCCCATCCTGCCAATATCCTTGCTTTCCTACTGATCTAGTGAGTCCT

GTAAAATCCTTCCTAAACATCCTCAACAATTTGGTTTTGAGATGCCCAATAAAAGGCTGT

CATGAGGATGTCTTTTTGGAAAAATACTGCCAACATCGTTCTAATCATAAAGAGGCGGAA

AGC

>MZ712316_ZMMUR14072_[Pareas_formosensis]

GACAGCCACCAGGCTAACTCTGCGAAAGAAAATCCAGCAGGTTCTATGGACGTCACTGAT

GAACAACCTAAGGCAGCAGCTGTAATGTCACAAGCGCCCTTTGAAACAGGCACTGAGTTG

AATAAAAATAGCTTGGCAAGAGAGAAAGATGTCTTTCACATGAGCCGAAGGGAGGTGGAA

GCTCACCAAGCAAATCTGCAGTGCCTCTGTCGCATCTGTGGAGGCTCCCTGAAAACTGAT

CCTTGCAAAAAGTGTCATCCAGTTCATGGACCAGTGGACGCGGAGACCCAGGCTCTTTTG

AGAAAGAAAGAAAAAAGAGCCACTTCTTGGCCAGATCTTCTTGCCAAAGTTTTTAAGATT

GATGTACGGGGAGATATCGACACAACCCATCCTACTCGTTTTTGTCACAATTGTTGGAAA

GTGATTCAAAGAAAAGTCAGCAATGCCCCCCATGAAGCACATCTTCTAGGGAAAGACCCA

GTGGAGTGGCGGTCTCATTCAACAAGCTGTGACGTTTGTGTCACTTCCTTTCGTGGAGTC

AAGAGAAAGAAACCCATGATGAATTCACAGCTGAGCAAAAAACTCAGGATTATTGCTGGA

CGTACGAGAAAAATAAGATGCATAAGAAAGGTAAAGCAGCTGAAAAACAAAAGTTTAATG

AAAAAGATTTCAAACTGTAAGCAAATCCATCTCAGTACAAAATCCCTTGCAATAGATTAT

CCTGTGGACTTTGTAAAGTCAATATCTTGCCAGATCTGTGAACACATCCTGTCCGATCCA

GTAGAAACAACATGTAAACACTTATTCTGTAGAGTCTGCATTCTTAAATGCCTTAAAGTA

ATGGGAAGCTATTGCCCATCCTGCCAATATCCTTGCTTTCCTACTGATCTAGTGAGTCCT

GTAAAATCCTTCCTAAACATCCTCAACAATTTGGTTTTGAGATGCCCAATAAAAGGCTGT

CATGAGGATGTCTTTTTGGAAAAATACTGCCAACATCGTTCTAATCATAAAGAGGCGGAA

AGC

>MZ712317_ZMMUR13709_[Pareas_formosensis]

GACAGCCACCAGGCTAACTCTGCGAAAGAAAATCCAGCAGGTTCTATGGACGTCACTGAT

GAACAACCTAAGGCAGCAGCTGTAATGTCACAAGCGCCCTTTGAAACAGGCACTGTGTTG

AATAAAAATAGCTTGGCAAGAGAGAAAGATGTCTTTCACATGAGCCGAAGGGAGGTGGAA

GCTCACCAAGCAAATCTGCAGTGCCTCTGTCGCATCTGTGGAGGCTCCCTGAAAACTGAT

CCTTGCAAAAAGTGTCATCCAGTTCATGGACCAGTGGACGCGGAGACCCAGGCTCTTTTG

AGAAAGAAAGAAAAAAGAGCCACTTCTTGGCCAGATCTTCTTGCCAAAGTTTTTAAGATT

GATGTACGGGGAGATATCGACACAACCCATCCTACTCGTTTTTGTCACAATTGTTGGAAA

GTGATTCAAAGAAAAGTCAGCAATGCCCCCCATGAAGCACATCTTCTAGGGAAAGACCCA

GTGGAGTGGCGGTCTCATTCAACAAGCTGTGACGTTTGTGTCACTTCCTTTCGTGGAGTC

AAGAGAAAGAAACCCATGATGAATTCACAGCTGAGCAAAAAACTCAGGATTATTGCTGGA

CGTACGAGAAAAATAAGATGCATAAGAAAGGTAAAGCAGCTGAAAAACAAAAGTTTAATG

AAAAAGATTTCAAACTGTAAGCAAATCCATCTCAGTACAAAATCCCTTGCAATAGATTAT

CCTGTGGACTTTGTAAAGTCAATATCTTGCCAGATCTGTGAACACATCCTGTCCGATCCA

GTAGAAACAACATGTAAACACTTATTCTGTAGAGTCTGCATTCTTAAATGCCTTAAAGTA

ATGGGAAGCTATTGCCCATCCTGCCAATATCCTTGCTTTCCTACTGATCTAGTGAGTCCT

GTAAAATCCTTCCTAAACATCCTCAACAATTTGGTTTTGAGATGCCCAATAAAAGGCTGT

CATGAGGATGTCTTTTTGGAAAAATACTGCCAACATCGTTCTAATCATAAAGAGGCGGAA

AGC

>MZ712318_ZMMUR16333_[Pareas_formosensis]

GACAGCCACCAGGCTAACTCTGCGAAAGAAAATCCAGCAGGTTCTATGGACGTCACTGAT

GAACAACCTAAGGCAGCAGCTGTAATGTCACAAGCGCCCTTTGAAACAGGCACTGAGTTG

AATAAAAATAGCTTGGCAAGAGAGAAAGATGTCTTTCACATGAGCCGAAGGGAGGTGGAA

GCTCACCAAGCAAATCTGCAGTGCCTCTGTCGCATCTGTGGAGGCTCCCTGAAAACTGAT

CCTTGCAAAAAGTGTCATCCAGTTCATGGACCAGTGGACGCGGAGACCCAGGCTCTTTTG

AGAAAGAAAGAAAAAAGAGCCACTTCTTGGCCAGATCTTCTTGCCAAAGTTTTTAAGATT

GATGTACGGGGAGATATCGACACAACCCATCCTACTCGTTTTTGTCACAATTGTTGGAAA

GTGATTCAAAGAAAAGTCAGCAATGCCCCCCATGAAGCACATCTTCTAGGGAAAGACCCA

GTGGAGTGGCGGTCTCATTCAACAAGCTGTGACGTTTGTGTCACTTCCTTTCGTGGAGTC

AAGAGAAAGAAACCCATGATGAATTCACAGCTGAGCAAAAAACTCAGGATTATTGCTGGA

CGTACGAGAAAAATAAGATGCATAAGAAAGGTAAAGCAGCTGAAAAACAAAAGTTTAATG

AAAAAGATTTCAAACTGTAAGCAAATCCATCTCAGTACAAAATCCCTTGCAATAGATTAT

CCTGTGGACTTTGTAAAGTCAATATCTTGCCAGATCTGTGAACACATCCTGTCCGATCCA

GTAGAAACAACATGTAAACACTTATTCTGTAGAGTCTGCATTCTTAAATGCCTTAAAGTA

ATGGGAAGCTATTGCCCATCCTGCCAATATCCTTGCTTTCCTACTGATCTAGTGAGTCCT

GTAAAATCCTTCCTAAACATCCTCAACAATTTGGTTTTGAGATGCCCAATAAAAGGCTGT

CATGAGGATGTCTTTTTGGAAAAATACTGCCAACATCGTTCTAATCATAAAGAGGCGGAA

AGC

>MZ712319_AUP01573_[Pareas_berdmorei_berdmorei]

GACAGCCACCAGGCTAACTCTGCTAAAGAAAATGCAACAGGTTCTATGGACATCACTGAT

GAACAACCTGAGGCAGCAGCTGTAATGTCACAAGTGCCCTTTGAAACAGGCACTGAGTTG

AATAAAAATAGCTTGGCAAGAGAGAAAGATGTCTTTCACATGAGCCAAAGGGAGGTGGAA

GCTCATCAAGCAAACCTGCAGTGTCTCTGTCGCATCTGTGGAGGCTCACTGAAAACTGAT

CCTTGCAAAAAGTGTCATCCAGTTCATGGACCAGTGGATGAGGAGACCCAGGCTCTTTTG

AGAAAGAAAGAAAAAAGAGCCACTTCTTGGCCAGATCTTCTTGCCAAAGTTTTTAAGATT

GATGTACGGGGAGATATTGACACAACCCATCCCACTCGTTTTTGTCACAATTGTTGGAAA

GTGATTCAAAGAAAAGTCAGCAATGCCCCCCATGAAGCACATCTTCTAGGGAAAGACCCA

GTGGAATGGCGGTCCCATTCAACGAGCTGTGATGTTTGTGTCACTTCCTTTCGTGGAGTC

AAGAGAAAAAAACCAATGTTGAATTCACAGCTGAGCAAAAAACTCAGGATTATTGCTGGA

CGTACGAGAAAAATAACATGCATAAGAAAGGTAAAGCAACTGAAAAATAAAAGTTTAATG

AAAAAGATTTCAAACTGTAAGCAAATCCATCTCAGTACAAAATCCCTTGCAATAGATTAT

CCTGTGGACTTTGTAAAGTCAATATCTTGCCAGATCTGTGAACACATCCTGTCCGATCCA

GTAGAAACAACATGTAAGCACTTATTCTGTAGAGTCTGCATTCTTAAATGCCTTAAAGTA

ATGGGCAGCTATTGCCCATCCTGCCGATATCCTTGCTTTCCTACTGATCTAGTGAGTCCT

GTAAAATCCTTCCTAAACATCCTCAACAATTTGGTTTTGAGATGCCCAATAAAAGGCTGT

CATGAGGATGTCTTTTTGGAAAAATACTGCCAACATCGTTCTAATCATAAAGAGGCAGAA

AGC

>MZ712320_CAS240362_[Pareas_berdmorei_berdmorei]

GACAGCCACCAGGCTAACTCTGCTAAAGAAAATGCAACAGGTTCTATGGACATCACTGAT

GAACAACCTGAGGCAGCAGCTGTAATGTCACAAGTGCCCTTTGAAACAGGCACTGAGTTG

AATAAAAATAGCTTGGCAAGAGAGAAAGATGTCTTTCACATGAGCCAAAGGGAGGTGGAA

GCTCATCAAGCAAACCTGCAGTGTCTCTGTCGCATCTGTGGAGGCTCACTGAAAACTGAT

CCTTGCAAAAAGTGTCATCCAGTTCATGGACCAGTGGATGAGGAGACCCAGGCTCTTTTG

AGAAAGAAAGAAAAAAGAGCCACTTCTTGGCCAGATCTTCTTGCCAAAGTTTTTAAGATT

GATGTACGGGGAGATATTGACACAACCCATCCCACTCGTTTTTGTCACAATTGTTGGAAA

GTGATTCAAAGAAAAGTCAGCAATGCCCCCCATGAAGCACATCTTCTAGGGAAAGACCCA

GTGGAATGGCGGTCCCATTCAACGAGCTGTGATGTTTGTGTCACTTCCTTTCGTGGAGTC

AAGAGAAAAAAACCAATGTTGAATTCACAGCTGAGCAAAAAACTCAGGATTATTGCTGGA

CGTACGAGAAAAATAACATGCATAAGAAAGGTAAAGCAACTGAAAAATAAAAGTTTAATG

AAAAAGATTTCAAACTGTAAGCAAATCCATCTCAGTACAAAATCCCTTGCAATAGATTAT

CCTGTGGACTTTGTAAAGTCAATATCTTGCCAGATCTGTGAACACATCCTGTCCGATCCA

GTAGAAACAACATGTAAGCACTTATTCTGTAGAGTCTGCATTCTTAAATGCCTTAAAGTA

ATGGGCAGCTATTGCCCATCCTGCCGATATCCTTGCTTTCCTACTGATCTAGTGAGTCCT

GTAAAATCCTTCCTAAACATCCTCAACAATTTGGTTTTGAGATGCCCAATAAAAGGCTGT

CATGAGGATGTCTTTTTGGAAAAATACTGCCAACATCGTTCTAATCATAAAGAGGCAGAA

AGC

>MZ712321_ZMMUR16803_[Pareas_berdmorei_berdmorei]

GACAGCCACCAGGCTAACTCTGCTAAAGAAAATGCAACAGGTTCTATGGACATCACTGAT

GAACAACCTGAGGCAGCAGCTGTAATGTCACAAGTGCCCTTTGAAACAGGCACTGAGTTG

AATAAAAATAGCTTGGCAAGAGAGAAAGATGTCTTTCACATGAGCCAAAGGGAGGTGGAA

GCTCATCAAGCAAACCTGCAGTGTCTCTGTCGCATCTGTGGAGGCTCACTGAAAACTGAT

CCTTGCAAAAAGTGTCATCCAGTTCATGGACCAGTGGATGAGGAGACCCAGGCTCTTTTG

AGAAAGAAAGAAAAAAGAGCCACTTCTTGGCCAGATCTTCTTGCCAAAGTTTTTAAGATT

GATGTACGGGGAGATATTGACACAACCCATCCCACTCGTTTTTGTCACAATTGTTGGAAA

GTGATTCAAAGAAAAGTCAGCAATGCCCCCCATGAAGCACATCTTCTAGGGAAAGACCCA

GTGGAATGGCGGTCCCATTCAACGAGCTGTGATGTTTGTGTCACTTCCTTTCGTGGAGTC

AAGAGAAAAAAACCAATGTTGAATTCACAGCTGAGCAAAAAACTCAGGATTATTGCTGGA

CGTACGAGAAAAATAACATGCATAAGAAAGGTAAAGCAACTGAAAAATAAAAGTTTAATG

AAAAAGATTTCAAACTGTAAGCAAATCCATCTCAGTACAAAATCCCTTGCAATAGATTAT

CCTGTGGACTTTGTAAAGTCAATATCTTGCCAGATCTGTGAACACATCCTGTCCGATCCA

GTAGAAACAACATGTAAGCACTTATTCTGTAGAGTCTGCATTCTTAAATGCCTTAAAGTA

ATGGGCAGCTATTGCCCATCCTGCCGATATCCTTGCTTTCCTACTGATCTAGTGAGTCCT

GTAAAATCCTTCCTAAACATCCTCAACAATTTGGTTTTGAGATGCCCAATAAAAGGCTGT

CATGAGGATGTCTTTTTGGAAAAATACTGCCAACATCGTTCTAATCATAAAGAGGCAGAA

AGC

>MZ712322_ZMMUR14796_[Pareas_berdmorei_truongsonicus]

GACAGCCACCAGGCTAACTCTGCTAAAGAAAATGCAACAGGTTCTATGGACGTCACTGAT

GAACAACCTGAGGCAGCAGCTGTAATGTCACAAATGTCCTTTGAAACAGGCACTGAGTTG

AATAAAAATAGCTTGGCAAGAGAGAAAGATGTCTTTCACATGAGCCAAAGGGAGGTGGAA

GCTCACCAAGCAAACCTGCAATGCCTCTGTCGCATCTGTGGAGGCTCATTGAAAACTGAT

CCTTGCAAAAAGTGTCATCCAGTTCATGGACCAGTGGATGAGGAGACCCAGGCTCTTTTG

AGAAAGAAAGAAAAAAGAGCCACTTCTTGGCCAGATCTTCTTGCCAAAGTTTTTAAGATT

GATGTACGGGGAGATATTGACACAACCCATCCCACTCGTTTTTGTCACAATTGTTGGAAA

GTGATTCAAAGAAAAGTCAGCAATGCCCCCCATGAAGCACATCTTCTAGGGAAAGACCCA

GTGGAATGGCAGTCCCATTCAACGAGCTGTGATGTTTGTGTCACTTCCTTTCGTGGAGTC

AAGAGAAAAAAACCAATGCTGAATTCACAGCTGAGAAAAAAACTCAGGATTATTGCTGGA

CGTACGAGAAAAATAACATGCATAAGAAAGGTAAAGCAACTGAAAAATAAAAGTTTAATG

AAAAAGATTTCAAACTGTAAGCAAATCCATCTCAGTACAAAATCCCTTGCAATAGATTAT

CCTGTGGACTTTGTAAAGTCAATATCTTGCCAGATCTGTGAACACATCCTGTCCGATCCA
GTAGAAACAACATGTAAGCACTTATTCTGTAGAGTCTGCATTCTTAAATGCCTTAAAGTA
ATGGGCAGCTATTGCCCATCCTGCCGATATCCTTGCTTTCCTACTGATCTAGTGAGTCCT
GTAAAATCCTTCCTAAACATCCTCAACAATTTGGTTTTGAGATGCCCAATAAAAGGCTGT
CATGAGGATGTCTTTTTGGAAAAATACTGCCAACATCGTTCTAATCATAAAGAGGCAGAA
AGC

>MZ712323_ZMMUR16801_[Pareas_berdmorei_truongsonicus]
GACAGCCACCAGGCTAACTCTGCTAAAGAAAATGCAACAGGTTCTATGGACGTCACTGAT
GAACAACCTGAGGCAGCAGCTGTAATGTCACAAATGTCCTTTGAAACAGGCACTGAGTTG
AATAAAAATAGCTTGGCAAGAGAGAAAGATGTCTTTCACATGAGCCAAAGGGAGGTGGAA
GCTCACCAAGCAAACCTGCAATGCCTCTGTCGCATCTGTGGAGGCTCATTGAAAACTGAT
CCTTGCAAAAAGTGTCATCCAGTTCATGGACCAGTGGATGAGGAGACCCAGGCTCTTTTG
AGAAAGAAAGAAAAAAGAGCCACTTCTTGGCCAGATCTTCTTGCCAAAGTTTTTAAGATT
GATGTACGGGGAGATATTGACACAACCCATCCCACTCGTTTTTGTCACAATTGTTGGAAA
GTGATTCAAAGAAAAGTCAGCAATGCCCCCCATGAAGCACATCTTCTAGGGAAAGACCCA
GTGGAATGGCAGTCCCATTCAACGAGCTGTGATGTTTGTGTCACTTCCTTTCGTGGAGTC
AAGAGAAAAAAACCAATGCTGAATTCACAGCTGAGAAAAAAACTCAGGATTATTGCTGGA
CGTACGAGAAAAATAACATGCATAAGAAAGGTAAAGCAACTGAAAAATAAAAGTTTAATG
AAAAAGATTTCAAACTGTAAGCAAATCCATCTCAGTACAAAATCCCTTGCAATAGATTAT
CCTGTGGACTTTGTAAAGTCAATATCTTGCCAGATCTGTGAACACATCCTGTCCGATCCA
GTAGAAACAACATGTAAGCACTTATTCTGTAGAGTCTGCATTCTTAAATGCCTTAAAGTA
ATGGGCAGCTATTGCCCATCCTGCCGATATCCTTGCTTTCCTACTGATCTAGTGAGTCCT
GTAAAATCCTTCCTAAACATCCTCAACAATTTGGTTTTGAGATGCCCAATAAAAGGCTGT
CATGAGGATGTCTTTTTGGAAAAATACTGCCAACATCGTTCTAATCATAAAGAGGCAGAA
AGC

>MZ712324_ZMMUR137531_[Pareas_berdmorei_unicolor]
GACAGCCACCAGGCTAACTCTGCTAAAGAAAATGCAACAGGTTCTATGGACATCACTGAT
GAACAACCTGAGGCAGCAGCTGTAATGTCACAAGTGTCCTTTGAAACAGGCACTGAGTTG
AATAAAAATAGCTTGGCAAGAGAGAAAGATGTCTTTCACATGAGCCAAAGGGAGGTGGAA

GCTCACCAAGCAAACCTGCAATGCCTCTGTCGCATCTGTGGAGGCTCACTGAAAACTGAT

CCTTGCAAAAAGTGTCATCCAGTTCATGGACCAGTGGATGAGGAGACCCAGGCTCTTTTG

AGAAAGAAAGAAAAAAGAGCCACTTCTTGGCCAGATCTTCTTGCCAAAGTTTTTAAGATT

GATGTACGGGGAGATATTGACACAACCCATCCCACTCGTTTTTGTCACAATTGTTGGAAA

GTGATTCAAAGAAAAGTCAGCAATGCCCCCCATGAAGCACATCTTCTAGGGAAAGACCCA

GTGGAATGGCAGTCCCATTCAACGAGCTGTGATGTTTGTGTCACTTCCTTTCGTGGAGTC

AAGAGAAAAAAACCAATGCTGAATTCACAGCTGAGCAAAAAACTCAGGATTATTGCTGGA

CGTACGAGAAAAATAACATGCATAAGAAAGGTAAAGCAACTGAAAAATAAAAGTTTAATG

AAAAAGATTTCAAACTGTAAGCAAATCCATCTCAGTACAAAATCCCTTGCAATAGATTAT

CCTGTGGACTTTGTAAAGTCAATATCTTGCCAGATCTGTGAACACATCCTGTCCGATCCA

GTAGAAACAACATGTAAGCACTTATTCTGTAGAGTCTGCATTCTTAAATGCCTTAAAGTA

ATGGGCAGCTATTGCCCATCCTGCCGATATCCTTGCTTTCCTACTGATCTAGTGAGTCCT

GTAAAATCCTTCCTAAACATCCTCAACAATTTGGTTTTGAGATGCCCAATAAAAGGCTGT

CATGAGGATGTCTTTTTGGAAAAATACTGCCAACATCGTTCTAATCATAAAGAGGCAGAA

AGC

>MZ712325_ZMMUR137532_[Pareas_berdmorei_unicolor]

GACAGCCACCAGGCTAACTCTGCTAAAGAAAATGCAACAGGTTCTATGGACATCACTGAT

GAACAACCTGAGGCAGCAGCTGTAATGTCACAAGTGTCCTTTGAAACAGGCACTGAGTTG

AATAAAAATAGCTTGGCAAGAGAGAAAGATGTCTTTCACATGAGCCAAAGGGAGGTGGAA

GCTCACCAAGCAAACCTGCAATGCCTCTGTCGCATCTGTGGAGGCTCACTGAAAACTGAT

CCTTGCAAAAAGTGTCATCCAGTTCATGGACCAGTGGATGAGGAGACCCAGGCTCTTTTG

AGAAAGAAAGAAAAAAGAGCCACTTCTTGGCCAGATCTTCTTGCCAAAGTTTTTAAGATT

GATGTACGGGGAGATATTGACACAACCCATCCCACTCGTTTTTGTCACAATTGTTGGAAA

GTGATTCAAAGAAAAGTCAGCAATGCCCCCCATGAAGCACATCTTCTAGGGAAAGACCCA

GTGGAATGGCAGTCCCATTCAACGAGCTGTGATGTTTGTGTCACTTCCTTTCGTGGAGTC

AAGAGAAAAAAACCAATGCTGAATTCACAGCTGAGCAAAAAACTCAGGATTATTGCTGGA

CGTACGAGAAAAATAACATGCATAAGAAAGGTAAAGCAACTGAAAAATAAAAGTTTAATG

AAAAAGATTTCAAACTGTAAGCAAATCCATCTCAGTACAAAATCCCTTGCAATAGATTAT

CCTGTGGACTTTGTAAAGTCAATATCTTGCCAGATCTGTGAACACATCCTGTCCGATCCA

GTAGAAACAACATGTAAGCACTTATTCTGTAGAGTCTGCATTCTTAAATGCCTTAAAGTA

ATGGGCAGCTATTGCCCATCCTGCCGATATCCTTGCTTTCCTACTGATCTAGTGAGTCCT

GTAAAATCCTTCCTAAACATCCTCAACAATTTGGTTTTGAGATGCCCAATAAAAGGCTGT

CATGAGGATGTCTTTTTGGAAAAATACTGCCAACATCGTTCTAATCATAAAGAGGCAGAA

AGC

>MZ712326_ZMMUR14013_[Pareas_berdmorei_unicolor]

GACAGCCACCAGGCTAACTCTGCTAAAGAAAATGCAACAGGTTCTATGGACATCACTGAT

GAACAACCTGAGGCAGCAGCTGTAATGTCACAAGTGTCCTTTGAAACAGGCACTGAGTTG

AATAAAAATAGCTTGGCAAGAGAGAAAGATGTCTTTCACATGAGCCAAAGGGAGGTGGAA

GCTCACCAAGCAAACCTGCAATGCCTCTGTCGCATCTGTGGAGGCTCACTGAAAACTGAT

CCTTGCAAAAAGTGTCATCCAGTTCATGGACCAGTGGATGAGGAGACCCAGGCTCTTTTG

AGAAAGAAAGAAAAAAGAGCCACTTCTTGGCCAGATCTTCTTGCCAAAGTTTTTAAGATT

GATGTACGGGGAGATATTGACACAACCCATCCCACTCGTTTTTGTCACAATTGTTGGAAA

GTGATTCAAAGAAAAGTCAGCAATGCCCCCCATGAAGCACATCTTCTAGGGAAAGACCCA

GTGGAATGGCAGTCCCATTCAACGAGCTGTGATGTTTGTGTCACTTCCTTTCGTGGAGTC

AAGAGAAAAAAACCAATGCTGAATTCACAGCTGAGCAAAAAACTCAGGATTATTGCTGGA

CGTACGAGAAAAATAACATGCATAAGAAAGGTAAAGCAACTGAAAAATAAAAGTTTAATG

AAAAAGATTTCAAACTGTAAGCAAATCCATCTCAGTACAAAATCCCTTGCAATAGATTAT

CCTGTGGACTTTGTAAAGTCAATATCTTGCCAGATCTGTGAACACATCCTGTCCGATCCA

GTAGAAACAACATGTAAGCACTTATTCTGTAGAGTCTGCATTCTTAAATGCCTTAAAGTA

ATGGGCAGCTATTGCCCATCCTGCCGATATCCTTGCTTTCCTACTGATCTAGTGAGTCCT

GTAAAATCCTTCCTAAACATCCTCAACAATTTGGTTTTGAGATGCCCAATAAAAGGCTGT

CATGAGGATGTCTTTTTGGAAAAATACTGCCAACATCGTTCTAATCATAAAGAGGCAGAA

AGC

>MZ712327_ZMMUR14263_[Pareas_berdmorei_unicolor]

GACAGCCACCAGGCTAACTCTGCTAAAGAAAATGCAACAGGTTCTATGGACATCACTGAT

GAACAACCTGAGGCAGCAGCTGTAATGTCACAAGTGTCCTTTGAAACAGGCACTGAGTTG

AATAAAAATAGCTTGGCAAGAGAGAAAGATGTCTTTCACATGAGCCAAAGGGAGGTGGAA

GCTCACCTAGCAAACCTGCAATGCCTCTGTCGCATCTGTGGAGGCTCACTGAAAACTGAT

CCTTGCAAAAAGTGTCATCCAGTTCATGGACCAGTGGATGAGGAGACCCAGGCTCTTTTG

AGAAAGAAAGAAAAAAGAGCCACTTCTTGGCCAGATCTTCTTGCCAAAGTTTTTAAGATT

GATGTACGGGGAGATATTGACACAACCCATCCCACTCGTTTTTGTCACAATTGTTGGAAA

GTGATTCAAAGAAAAGTCAGCAATGCCCCCCATGAAGCACATCTTCTAGGGAAAGACCCA

GTGGAATGGCAGTCCCATTCAACGAGCTGTGATGTTTGTGTCACTTCCTTTCGTGGAGTC

AAGAGAAAAAAACCAATGCTGAATTCACAGCTGAGCAAAAAACTCAGGATTATTGCTGGA

CGTACGAGAAAAATAACATGCATAAGAAAGGTAAAGCAACTGAAAAATAAAAGTTTAATG

AAAAAGATTTCAAACTGTAAGCAAATCCATCTCAGTACAAAATCCCTTGCGATAGATTAT

CCTGTGGACTTTGTAAAGTCAATATCTTGCCAGATCTGTGAACACATCCTGTCCGATCCA

GTAGAAACAACATGTAAGCACTTATTCTGTAGAGTCTGCATTCTTAAATGCCTTAAAGTA

ATGGGCAGCTATTGCCCATCCTGCCGATATCCTTGCTTTCCTACTGATCTAGTGAGTCCT

GTAAAATCCTTCCTAAACATCCTCAACAATTTGGTTTTGAGATGCCCAATAAAAGGCTGT

CATGAGGATGTCTTTTTGGAAAAATACTGCCAACATCGTTCTAATCATAAAGAGGCAGAA

AGC

>MZ712328_ZMMUR14421_[Pareas_berdmorei_unicolor]

GACAGCCACCAGGCTAACTCTGCTAAAGAAAATGCAACAGGTTCTATGGACATCACTGAT

GAACAACCTGAGGCAGCAGCTGTAATGTCACAAGTGTCCTTTGAAACAGGCACTGAGTTG

AATAAAAATAGCTTGGCAAGAGAGAAAGATGTCTTTCACATGAGCCAAAGGGAGGTGGAA

GCTCACCAAGCAAACCTGCAATGCCTCTGTCGCATCTGTGGAGGCTCACTGAAAACTGAT

CCTTGCAAAAAGTGTCATCCAGTTCATGGACCAGTGGATGAGGAGACCCAGGCTCTTTTG

AGAAAGAAAGAAAAAAGAGCCACTTCTTGGCCAGATCTTCTTGCCAAAGTTTTTAAGATT

GATGTACGGGGAGATATTGACACAACCCATCCCACTCGTTTTTGTCACAATTGTTGGAAA

GTGATTCAAAGAAAAGTCAGCAATGCCCCCCATGAAGCACATCTTCTAGGGAAAGACCCA

GTGGAATGGCAGTCCCATTCAACGAGCTGTGATGTTTGTGTCACTTCCTTTCGTGGAGTC

AAGAGAAAAAAACCAATGCTGAATTCACAGCTGAGCAAAAAACTCAGGATTATTGCTGGA

CGTACGAGAAAAATAACATGCATAAGAAAGGTAAAGCAACTGAAAAATAAAAGTTTAATG

AAAAAGATTTCAAACTGTAAGCAAATCCATCTCAGTACAAAATCCCTTGCGATAGATTAT

CCTGTGGACTTTGTAAAGTCAATATCTTGCCAGATCTGTGAACACATCCTGTCCGATCCA

GTAGAAACAACATGTAAGCACTTATTCTGTAGAGTCTGCATTCTTAAATGCCTTAAAGTA

ATGGGCAGCTATTGCCCATCCTGCCAATATCCTTGCTTTCCTACTGATCTAGTGAGTCCT

GTAAAATCCTTCCTAAACATCCTCAACAATTTGGTTTTGAGATGCCCAATAAAAGGCTGT

CATGAGGATGTCTTTTTGGAAAAATACTGCCAACATCGTTCTAATCATAAAGAGGCAGAA

AGC

>MZ712329_SIEZC20216_[Pareas_berdmorei_unicolor]

GACAGCCACCAGGCTAACTCTGCTAAAGAAAATGCAACAGGTTCTATGGACATCACTGAT

GAACAACCTGAGGCAGCAGCTGTAATGTCACAAGTGTCCTTTGAAACAGGCACTGAGTTG

AATAAAAATAGCTTGGCAAGAGAGAAAGATGTCTTTCACATGAGCCAAAGGGAGGTGGAA

GCTCACCAAGCAAACCTGCAATGCCTCTGTCGCATCTGTGGAGGCTCACTGAAAACTGAT

CCTTGCAAAAAGTGTCATCCAGTTCATGGACCAGTGGATGAGGAGACCCAGGCTCTTTTG

AGAAAGAAAGAAAAAAGAGCCACTTCTTGGCCAGATCTTCTTGCCAAAGTTTTTAAGATT

GATGTACGGGGAGATATTGACACAACCCATCCCACTCGTTTTTGTCACAATTGTTGGAAA

GTGATTCAAAGAAAAGTCAGCAATGCCCCCCATGAAGCACATCTTCTAGGGAAAGACCCA

GTGGAATGGCAGTCCCATTCAACGAGCTGTGATGTTTGTGTCACTTCCTTTCGTGGAGTC

AAGAGAAAAAAACCAATGCTGAATTCACAGCTGAGCAAAAAACTCAGGATTATTGCTGGA

CGTACGAGAAAAATAACATGCATAAGAAAGGTAAAGCAACTGAAAAATAAAAGTTTAATG

AAAAAGATTTCAAACTGTAAGCAAATCCATCTCAGTACAAAATCCCTTGCGATAGATTAT

CCTGTGGACTTTGTAAAGTCAATATCTTGCCAGATCTGTGAACACATCCTGTCCGATCCA

GTAGAAACAACATGTAAGCACTTATTCTGTAGAGTCTGCATTCTTAAATGCCTTAAAGTA

ATGGGCAGCTATTGCCCATCCTGCCAATATCCTTGCTTTCCTACTGATCTAGTGAGTCCT

GTAAAATCCTTCCTAAACATCCTCAACAATTTGGTTTTGAGATGCCCAATAAAAGGCTGT

CATGAGGATGTCTTTTTGGAAAAATACTGCCAACATCGTTCTAATCATAAAGAGGCAGAA

AGC

>MZ712330_ZMMUR136791_[Pareas_berdmorei_unicolor]

GACAGCCACCAGGCTAACTCTGCTAAAGAAAATGCAACAGGTTCTATGGACATCACTGAT

GAACAACCTGAGGCAGCAGCTGTAATGTCACAAGTGTCCTTTGAAACAGGCACTGAGTTG

AATAAAAATAGCTTGGCAAGAGAGAAAGATGTCTTTCACATGAGCCAAAGGGAGGTGGAA

GCTCACCAAGCAAACCTGCAATGCCTCTGTCGCATCTGTGGAGGCTCACTGAAAACTGAT

CCTTGCAAAAAGTGTCATCCAGTTCATGGACCAGTGGATGAGGAGACCCAGGCTCTTTTG

AGAAAGAAAGAAAAAAGAGCCACTTCTTGGCCAGATCTTCTTGCCAAAGTTTTTAAGATT

GATGTACGGGGAGATATTGACACAACCCATCCCACTCGTTTTTGTCACAATTGTTGGAAA

GTGATTCAAAGAAAAGTCAGCAATGCCCCCCATGAAGCACATCTTCTAGGGAAAGACCCA

GTGGAATGGCAGTCCCATTCAACGAGCTGTGATGTTTGTGTCACTTCCTTTCGTGGAGTC

AAGAGAAAAAAACCAATGCTGAATTCACAGCTGAGCAAAAAACTCAGGATTATTGCTGGA

CGTACGAGAAAAATAACATGCATAAGAAAGGTAAAGCAACTGAAAAATAAAAGTTTAATG

AAAAAGATTTCAAACTGTAAGCAAATCCATCTCAGTACAAAATCCCTTGCAATAGATTAT

CCTGTGGACTTTGTAAAGTCAATATCTTGCCAGATCTGTGAACACATCCTGTCCGATCCA

GTAGAAACAACATGTAAGCACTTATTCTGTAGAGTCTGCATTCTTAAATGCCTTAAAGTA

ATGGGCAGCTATTGCCCATCCTGCCGATATCCTTGCTTTCCTACTGATCTAGTGAGTCCT

GTAAAATCCTTCCTAAACATCCTCAACAATTTGGTTTTGAGATGCCCAATAAAAGGCTGT

CATGAGGATGTCTTTTTGGAAAAATACTGCCAACATCGTTCTAATCATAAAGAGGCAGAA

AGC

>MZ712331_ZMMUR136792_[Pareas_berdmorei_unicolor]

GACAGCCACCAGGCTAACTCTGCTAAAGAAAATGCAACAGGTTCTATGGACATCACTGAT

GAACAACCTGAGGCAGCAGCTGTAATGTCACAAGTGTCCTTTGAAACAGGCACTGAGTTG

AATAAAAATAGCTTGGCAAGAGAGAAAGATGTCTTTCACATGAGCCAAAGGGAGGTGGAA

GCTCACCAAGCAAACCTGCAATGCCTCTGTCGCATCTGTGGAGGCTCACTGAAAACTGAT

CCTTGCAAAAAGTGTCATCCAGTTCATGGACCAGTGGATGAGGAGACCCAGGCTCTTTTG

AGAAAGAAAGAAAAAAGAGCCACTTCTTGGCCAGATCTTCTTGCCAAAGTTTTTAAGATT

GATGTACGGGGAGATATTGACACAACCCATCCCACTCGTTTTTGTCACAATTGTTGGAAA

GTGATTCAAAGAAAAGTCAGCAATGCCCCCCATGAAGCACATCTTCTAGGGAAAGACCCA

GTGGAATGGCAGTCCCATTCAACGAGCTGTGATGTTTGTGTCACTTCCTTTCGTGGAGTC

AAGAGAAAAAAACCAATGCTGAATTCACAGCTGAGCAAAAAACTCAGGATTATTGCTGGA

CGTACGAGAAAAATAACATGCATAAGAAAGGTAAAGCAACTGAAAAATAAAAGTTTAATG

AAAAAGATTTCAAACTGTAAGCAAATCCATCTCAGTACAAAATCCCTTGCAATAGATTAT

CCTGTGGACTTTGTAAAGTCAATATCTTGCCAGATCTGTGAACACATCCTGTCCGATCCA

GTAGAAACAACATGTAAGCACTTATTCTGTAGAGTCTGCATTCTTAAATGCCTTAAAGTA

ATGGGCAGCTATTGCCCATCCTGCCGATATCCTTGCTTTCCTACTGATCTAGTGAGTCCT

GTAAAATCCTTCCTAAACATCCTCAACAATTTGGTTTTGAGATGCCCAATAAAAGGCTGT
CATGAGGATGTCTTTTTGGAAAAATACTGCCAACATCGTTCTAATCATAAAGAGGCAGAA
AGC
>MZ712332_ZMMUR14165_[Pareas_berdmorei_unicolor]
GACAGCCACCAGGCTAACTCTGCTAAAGAAAATGCAACAGGTTCTATGGACATCACTGAT
GAACAACCTGAGGCAGCAGCTGTAATGTCACAAGTGTCCTTTGAAACAGGCACTGAGTTG
AATAAAAATAGCTTGGCAAGAGAGAAAGATGTCTTTCACATGAGCCAAAGGGAGGTGGAA
GCTCACCAAGCAAACCTGCAATGCCTCTGTCGCATCTGTGGAGGCTCACTGAAAACTGAT
CCTTGCAAAAAGTGTCATCCAGTTCATGGACCAGTGGATGAGGAGACCCAGGCTCTTTTG
AGAAAGAAAGAAAAAAGAGCCACTTCTTGGCCAGATCTTCTTGCCAAAGTTTTTAAGATT
GATGTACGGGGAGATATTGACACAACCCATCCCACTCGTTTTTGTCACAATTGTTGGAAA
GTGATTCAAAGAAAAGTCAGCAATGCCCCCCATGAAGCACATCTTCTAGGGAAAGACCCA
GTGGAATGGCAGTCCCATTCAACGAGCTGTGATGTTTGTGTCACTTCCTTTCGTGGAGTC
AAGAGAAAAAAACCAATGCTGAATTCACAGCTGAGCAAAAAACTCAGGATTATTGCTGGA
CGTACGAGAAAAATAACATGCATAAGAAAGGTAAAGCAACTGAAAAATAAAAGTTTAATG
AAAAAGATTTCAAACTGTAAGCAAATCCATCTCAGTACAAAATCCCTTGCAATAGATTAT
CCTGTGGACTTTGTAAAGTCAATATCTTGCCAGATCTGTGAACACATCCTGTCCGATCCA
GTAGAAACAACATGTAAGCACTTATTCTGTAGAGTCTGCATTCTTAAATGCCTTAAAGTA
ATGGGCAGCTATTGCCCATCCTGCCGATATCCTTGCTTTCCTACTGATCTAGTGAGTCCT
GTAAAATCCTTCCTAAACATCCTCAACAATTTGGTTTTGAGATGCCCAATAAAAGGCTGT
CATGAGGATGTCTTTTTGGAAAAATACTGCCAACATCGTTCTAATCATAAAGAGGCAGAA
AGC
>MZ712333_ZMMUR16800_[Pareas_carinatus_tenasserimicus]
GACAGCCACCAGGCTAACTCTGCTAAAGAAAATGCAACAGGTTCTATGGACATCACTGGT
GAACAACTTGAGGCAGCATCTGTAATGTCACAAGTGCCCTTTGAAACAGGCACTGAGTTG
AATAAAAATAGCTTGGCAAGAGAGAAAGATGTCTTTCACATGAGACAAAGAGAGGTGGAA
GCTCACCAAGCAAACCTGCAATGCCTCTGTCGCATCTGTGGAGGCTCACTGAAAACTGAT
CCTTGCAAAAAGTGTCATCCAGTTCATGGACCAGTGGATGAGGAGACCCAGGCTCTTTTG
AGAAAGAAAGAAAAAAGAGCCACTTCTTGGCCAGATCTTCTTGCCAAAGTTTTTAAGATT

GATGTACGGGGAGATATCGACACAACCCATCCCACTCGTTTTTGTCACAATTGTTGGAAA

GTGATTCAAAGAAAAGTCAGCAATGCCCCCCATGAAGCACATCTTCTAGGGAAAGACCCA

GTGGAATGGCGGTCCCATTCAACGAGCTGTGATGTTTGTGTCACTTCCTTTCGTGGAGTC

AAGAGAAAAAAACCAATGCTGAATTCACAGCTGAGCAAAAAACTCAGGATTATTGCTGGA

CATACGAGAAAAATAACATGCATAAGAAAGGTAAAGCAACTGAAAAATAAAAGTTTAATG

AAAAAGATTTCAAACTGTAAGCAAATCCATCTCAGTACAAAATCCCTTGCAATAGATTAT

CCTGTGGACTTTGTAAAGTCAATATCTTGCCAGATCTGTGAACACATCCTGTCCGATCCA

GTAGAAACAACATGTAAGCACTTATTCTGTAGAGTCTGCATTCTTAAATGCCTTAAAGTA

ATGGGCAGCTATTGCCCATCCTGCCGATATCCTTGCTTTCCTACTGATCTAGTGAGTCCT

GTAAAATCCTTCCTAAACATCCTTAACAATTTGGTTTTGAGATGCCCAATAAAAGGCTGC

CATGAGGATGTCTTTTTGGAAAAATACTGCCAACATCGTTCTAATCATAAAGAGGCAGAA

AGC

>MZ712334_CAS247982_[Pareas_carinatus_tenasserimicus]

GACAGCCACCAGGCTAACTCTGCTAAAGAAAATGCAACAGGTTCTATGGACATCACTGGT

GAACAACTTGAGGCAGCATCTGTAATGTCACAAGTGCCCTTTGAAACAGGCACTGAGTTG

AATAAAAATAGCTTGGCAAGAGAGAAAGATGTCTTTCACATGAGACAAAGAGAGGTGGAA

GCTCACCAAGCAAACCTGCAATGCCTCTGTCGCATCTGTGGAGGCTCACTGAAAACTGAT

CCTTGCAAAAAGTGTCATCCAGTTCATGGACCAGTGGATGAGGAGACCCAGGCTCTTTTG

AGAAAGAAAGAAAAAAGAGCCACTTCTTGGCCAGATCTTCTTGCCAAAGTTTTTAAGATT

GATGTACGGGGAGATATCGACACAACCCATCCCACTCGTTTTTGTCACAATTGTTGGAAA

GTGATTCAAAGAAAAGTCAGCAATGCCCCCCATGAAGCACATCTTCTAGGGAAAGACCCA

GTGGAATGGCGGTCCCATTCAACGAGCTGTGATGTTTGTGTCACTTCCTTTCGTGGAGTC

AAGAGAAAAAAACCAATGCTGAATTCACAGCTGAGCAAAAAACTCAGGATTATTGCTGGA

CATACGAGAAAAATAACATGCATAAGAAAGGTAAAGCAACTGAAAAATAAAAGTTTAATG

AAAAAGATTTCAAACTGTAAGCAAATCCATCTCAGTACAAAATCCCTTGCAATAGATTAT

CCTGTGGACTTTGTAAAGTCAATATCTTGCCAGATCTGTGAACACATCCTGTCCGATCCA

GTAGAAACAACATGTAAGCACTTATTCTGTAGAGTCTGCATTCTTAAATGCCTTAAAGTA

ATGGGCAGCTATTGCCCATCCTGCCGATATCCTTGCTTTCCTACTGATCTAGTGAGTCCT

GTAAAATCCTTCCTAAACATCCTTAACAATTTGGTTTTGAGATGCCCAATAAAAGGCTGC

CATGAGGATGTCTTTTTGGAAAAATACTGCCAACATCGTTCTAATCATAAAGAGGCAGAA

AGC

>MZ712335_ZMMUR16802_[Pareas_kuznetsovorum]

GACAGCCACCAGGCTAACTCTGCTAAAGAAAATGCAACAGATTCTATGGACATCACTGAT

GAACAACCTAAGGCACCATCTGTAATGTCACAAGTGCCCTTTGAAACAGGCACTGAGTTG

AATAAAAATAGCTTGGCAAGAGAGAAAGATGTCTTTCACATGAGCCAAAGGGAGGTGGAA

GCTCACCAAGCAAACCTGCAGTGTCTCTGTCGCATCTGTGGAGGCTCACTGAAAACTGAT

CCTTGCAAAAAGTGTCATCCAGTTCATGGACCAGTGGATGAGGAGACCCAGGCTCTTTTG

AGAAAGAAAGAAAAAAGAGCCACTTCTTGGCCAGATCTTCTTGCCAAAGTGTTTAAGATT

GATGTACGGGGAGATATTGACACAACCCATCCTACTCGTTTTTGTCACAATTGTTGGAAA

GTGATTCAAAGAAAAGTCAGCAATGCCCCCCATGAAGCACATCTTTTAGGGAAAGACCCA

GTGGAATGGCGGTCCCATTCAACGAGCTGTGATGTTTGTGTCACTTCCTTTCGTGGAGTC

AAGAGAAAAAAAACAATGCTGAATTCACAGCTGAGCAAAAAACTCAGGATTATTGCTGGA

CGTACGAGAAAAATAACATGCATAAGAAAGGTAAAGCAACTAAAAAACAAAAGTTTAATG

AAAAAGATTTCAAACTGCAAGCAAATCCATCTCAGTACAAAATCCCTTGCAATAGATTAT

CCTGTGGACTTTGTAAAGTCAATATCTTGCCAGATCTGTGAACACATCCTGTCCGATCCA

GTAGAAACAACATGTAAGCACTTATTCTGTAGAGTCTGCATTCTTAAATGCCTTAAAGTA

ATGGGCAGCTATTGCCCATCCTGCCGATATCCTTGCTTTCCTACTGATCTAGTGAGTCCT

GTAAAATCCTTCCTAAACATCCTCAACAATTTGGTTTTGAGATGCCCAATAAAAGGCTGT

CATGAGGATGTCTTTTTGGAAAAATACTGCCAACATCGTTCTAATCATAAAGAGGCAGAA

AGC

>MZ712336_LSUHC10604_[Pareas_carinatus_carinatus]

GACAGCCACCAGGCTAACTCTGCTAAAGAAAATGCAACAGGTTCTATGGACATCACTGGT

GAACAACTTGAGGCAGCATCTGTAATGTCACAAGTGCCCTTTGAAACAGGCACTGAGTTG

AATAAAAATAGCTTGGCAAGAGAGAAAGATGTCTTTCACATGAGACAAAGAGAGGTGGAA

GCTCACCAAGCAAACCTGCAATGCCTCTGTCGCATCTGTGGAGGCTCACTGAAAACTGAT

CCTTGCAAAAAGTGTCATCCAGTTCATGGACCAGTGGATGAGGAGACCCAGGCTCTTTTG

AGAAAGAAAGAAAAAAGAGCCACTTCTTGGCCAGATCTTCTTGCCAAAGTTTTTAAGATT

GATGTACGGGGAGATATCGACACAACCCATCCCACTCGTTTTTGTCACAATTGTTGGAAA

GTGATTCAAAGAAAAGTCAGCAATGCCCCCCATGAAGCACATCTTCTAGGGAAAGACCCA

GTGGAATGGCGGTCCCATTCAACGAGCTGTGATGTTTGTGTCACTTCCTTTCGTGGAGTC

AAGAGAAAAAAACCAATGCTGAATTCACAGCTGAGCAAAAAACTCAGGATTATTGCTGGA

CATACGAGAAAAATAACATGCATAAGAAAGGTAAAGCAACTGAAAAATAAAAGTTTAATG

AAAAAGATTTCAAACTGTAAGCAAATCCATCTCAGTACAAAATCCCTTGCAATAGATTAT

CCTGTGGACTTTGTAAAGTCAATATCTTGCCAGATCTGTGAACACATCCTGTCCGATCCA

GTAGAAACAACATGTAAGCACTTATTCTGTAGAGTCTGCATTCTTAAATGCCTTAAAGTA

ATGGGCAGCTATTGCCCATCCTGCCGATATCCTTGCTTTCCTACTGATCTAGTGAGTCCT

GTAAAATCCTTCCTAAACATCCTTAACAATTTGGTTTTGAGATGCCCAATAAAAGGCTGC

CATGAGGATGTCTTTTTGGAAAAATACTGCCAACATCGTTCTAATCATAAAGAGGCAGAA

AGC

>MZ712337_ZMMUR16393_[Pareas_abros]

GACAGCCACCAGGCTAACTCTGCTAAAGAAAATGCAACAGGTTCTATGGACATCACTGAT

GAACAACCTGAGGCAGCAGCTGTAATGTCACAAGTGTCCTTTGAAACAGGCACTGAGTTG

AATAAAAATAGCTTGGCAAGAGAGAAAGATGTCTTTCACATGAGCCAAAGGGAGGTGGAA

GCTCACCAAGCAAACCTGCAGTGCCTCTGTCGCATCTGTGGAGGCTCACTGAAAACTGAT

CCTTGCAAAAAGTGTCATCCGGTTCATGGACCAGTGGATGAGGAGACCCAGGCTCTTTTG

AGAAAGAAAGAAAAAAGAGCCACTTCTTGGCCAGATCTTCTTGCCAAAGTTTTTAAGATT

GATGTACGGGGAGATATTGACACAACCCACCCTACTCGTTTTTGTCACAATTGTTGGAAA

GTGATTCAAAGAAAAGTCAGCAATGCCCCCCACGAAGCACATCTTCTAGGGAAAGACCCA

GTGGAATGGCGGCCCCATTCAATGAGCTGTGACGTTTGTGTCACTTCCTTTCGTGGAGTC

AAGAGAAAGAAACCAATGCTGAATTCACAGCTGAGCAAAAAACTCAGGATTATTGCTGGA

CGTACGAGAAAAATAACATGCATAAGAAAGGTAAAGCAACTGAAAAACAAAAGTTTAATG

AAAAAGATTTCAAACTGTAAGCAAATCCATCTCAGTACAAAATCCCTTGCAATAGATTAT

CCTGTGGACTTTGTAAAGTCAATATCTTGCCAGATCTGTGAACACATCCTGTCCGATCCA

GTAGAAACAACATGTAAGCACTTATTCTGTAGAGTCTGCATTCTTAAATGCCTTAAAGTA

ATGGGAAGCTATTGCCCATCCTGCCGATATCCTTGCTTTTCTACTGATCTAGTGAGTCCT

GTAAAATCCTTCCTAAACATCCTCAACAATTTGGTTTTGAGATGCCCAATAAAAGGCTGT

CATGAGGATGTCTTTTTGGAAAAATACTGCCAACATCGTTCTAATCATAAAGAGGCAGAA

AGC

>MZ712338_ZMMUR16392_[Pareas_abros]

GACAGCCACCAGGCTAACTCTGCTAAAGAAAATGCAACAGGTTCTATGGACATCACTGAT

GAACAACCTGAGGCAGCAGCTGTAATGTCACAAGTGCCCTTTGAAACAGGCACTGAGTTG

AATAAAAATAGCTTGGCAAGAGAGAAAGATGTCTTTCACATGAGCCAAAGGGAGGTGGAA

GCTCACCAAGCAAACCTGCAGTGCCTCTGTCGCATCTGTGGAGGCTCACTGAAAACTGAT

CCTTGCAAAAAGTGTCATCCGGTTCATGGACCAGTGGATGAGGAGACCCAGGCTCTTTTG

AGAAAGAAAGAAAAAAGAGCCACTTCTTGGCCAGATCTTCTTGCCAAAGTTTTTAAGATT

GATGTACGGGGAGATATTGACACAACCCACCCTACTCGTTTTTGTCACAATTGTTGGAAA

GTGATTCAAAGAAAAGTCAGCAATGCCCCCCACGAAGCACATCTTCTAGGGAAAGACCCA

GTGGAATGGCGGCCCCATTCAATGAGCTGTGACGTTTGTGTCACTTCCTTTCGTGGAGTC

AAGAGAAAGAAACCAATGCTGAATTCACAGCTGAGCAAAAAACTCAGGATTATTGCTGGA

CGTACGAGAAAAATAACATGCATAAGAAAGGTAAAGCAACTGAAAAACAAAAGTTTAATG

AAAAAGATTTCAAACTGTAAGCAAATCCATCTCAGTACAAAATCCCTTGCAATAGATTAT

CCTGTGGACTTTGTAAAGTCAATATCTTGCCAGATCTGTGAACACATCCTGTCCGATCCA

GTAGAAACAACATGTAAGCACTTATTCTGTAGAGTCTGCATTCTTAAATGCCTTAAAGTA

ATGGGAAGCTATTGCCCATCCTGCCGATATCCTTGCTTTTCTACTGATCTAGTGAGTCCT

GTAAAATCCTTCCTAAACATCCTCAACAATTTGGTTTTGAGATGCCCAATAAAAGGCTGT

CATGAGGATGTCTTTTTGGAAAAATACTGCCAACATCGTTCTAATCATAAAGAGGCAGAA

AGC

>MZ712339_ZMMUR14788_[Pareas_abros]

GACAGCCACCAGGCTAACTCTGCTAAAGAAAATGCAACAGGTTCTATGGACATCACTGAT

GAACAACCTGAGGCAGCAGCTGTAATGTCACAAGTGCCCTTTGAAACAGGCACTGAGTTG

AATAAAAATAGCTTGGCAAGAGAGAAAGATGTCTTTCACATGAGCCAAAGGGAGGTGGAA

GCTCACCAAGCAAACCTGCAGTGCCTCTGTCGCATCTGTGGAGGCTCACTGAAAACTGAT

CCTTGCAAAAAGTGTCATCCGGTTCATGGACCAGTGGATGAGGAGACCCAGGCTCTTTTG

AGAAAGAAAGAAAAAAGAGCCACTTCTTGGCCAGATCTTCTTGCCAAAGTTTTTAAGATT

GATGTACGGGGAGATATTGACACAACCCACCCTACTCGTTTTTGTCACAATTGTTGGAAA

GTGATTCAAAGAAAAGTCAGCAATGCCCCCCACGAAGCACATCTTCTAGGGAAAGACCCA

GTGGAATGGCGGCCCCATTCAATGAGCTGTGACGTTTGTGTCACTTCCTTTCGTGGAGTC

AAGAGAAAGAAACCAATGCTGAATTCACAGCTGAGCAAAAAACTCAGGATTATTGCTGGA

CGTACGAGAAAAATAACATGCATAAGAAAGGTAAAGCAACTGAAAAACAAAAGTTTAATG

AAAAAGATTTCAAACTGTAAGCAAATCCATCTCAGTACAAAATCCCTTGCAATAGATTAT

CCTGTGGACTTTGTAAAGTCAATATCTTGCCAGATCTGTGAACACATCCTGTCCGATCCA

GTAGAAACAACATGTAAGCACTTATTCTGTAGAGTCTGCATTCTTAAATGCCTTAAAGTA

ATGGGAAGCTATTGCCCATCCTGCCGATATCCTTGCTTTTCTACTGATCTAGTGAGTCCT

GTAAAATCCTTCCTAAACATCCTCAACAATTTGGTTTTGAGATGCCCAATAAAAGGCTGT

CATGAGGATGTCTTTTTGGAAAAATACTGCCAACATCGTTCTAATCATAAAGAGGCAGAA

AGC

>MZ712340_ZMMUR13656_[Pareas_temporalis]

GACAGCCACCAGGCTAACTCTGCTAAAGAAAATGCAGCAGGTTCTATGGACATCACTGAT

GAACAACCTGAGGCAGCAGCTGTAATGTCACAAGTGCCCTTTGAAACAGGCACTGAGTTG

AATAAAAATAGCTTGGCAAGAGAGAAAGGTGTCTTTCACATGAGCCAAAGGGAGGTGGAA

GCTCACCAAGCAAACCTGCAGTGCCTCTGTCGCATCTGTGGAGGCTCACTGAAAACTGAT

CCTTGCAAAAAGTGTCATCCAGTTCACGGACCAGTGGATGAGGAGACCCTGGCTCTTTTG

AGAAAGAAAGAAAAAAGAGCCACTTCTTGGCCAGATCTTCTTGCCAAAGTTTTTAAGATT

GATGTACGGGGAGATATTGACACAACCCACCCTACTCGTTTTTGTCACAATTGTTGGAAA

GTGATTCAAAGAAAAGTCAGCAATGCCCCCCATGAAGCACATCTTCTAGGGAAAGACCCA

GTGGAATGGCGGCCCCATTCAATGAGCTGTGACGTTTGTGTCACTTCCTTTCGTGGAGTC

AAGAGAAAGAAACCAATGCTGAATTCACAGCTGAGCAAAAAACTCAGGATTATTGCTGGA

CGTACGAGAAAAATAACATGCATAAGAAAGGTAAAGCAACTGAAAAACAAAAGTTTAATG

AAAAAGATTTCAAACTGTAAGCAAATCCATCTCAGTACAAAATCCCTTGCAATAGATTAT

CCTGTGGACTTTGTAAAGTCAATATCTTGCCAGATCTGTGAACACATCCTGTCCGATCCA

GTAGAAACAACATGTAAGCACTTATTCTGTAGAGTCTGCATTCTTAAATGCCTTAAAGTA

ATGGGAAGCTATTGCCCATCCTGCCGATATCCTTGCTTTTCTACTGATCTAGTGAGTCCT

GTAAAATCCTTCCTAAACATCCTCAACAATTTGGTTTTGAGATGCCCAATAAAAGGCTGT

CATGAGGATGTCTTTTTGGAAAAATACTGCCAACATCGTTCTAATCATAAAGAGGCAGAA

AGC

>MZ712341_SIEZC20215_[Pareas_temporalis]

GACAGCCACCAGGCTAACTCTGCTAAAGAAAATGCAGCAGGTTCTATGGACATCACTGAT

GAACAACCTGAGGCAGCAGCTGTAATGTCACAAGTGCCCTTTGAAACAGGCACTGAGTTG

AATAAAAATAGCTTGGCAAGAGAGAAAGGTGTCTTTCACATGAGCCAAAGGGAGGTGGAA

GCTCACCAAGCAAACCTGCAGTGCCTCTGTCGCATCTGTGGAGGCTCACTGAAAACTGAT

CCTTGCAAAAAGTGTCATCCAGTTCACGGACCAGTGGATGAGGAGACCCTGGCTCTTTTG

AGAAAGAAAGAAAAAAGAGCCACTTCTTGGCCAGATCTTCTTGCCAAAGTTTTTAAGATT

GATGTACGGGGAGATATTGACACAACCCACCCTACTCGTTTTTGTCACAATTGTTGGAAA

GTGATTCAAAGAAAAGTCAGCAATGCCCCCCATGAAGCACATCTTCTAGGGAAAGACCCA

GTGGAATGGCGGCCCCATTCAATGAGCTGTGACGTTTGTGTCACTTCCTTTCGTGGAGTC

AAGAGAAAGAAACCAATGCTGAATTCACAGCTGAGCAAAAAACTCAGGATTATTGCTGGA

CGTACGAGAAAAATAACATGCATAAGAAAGGTAAAGCAACTGAAAAACAAAAGTTTAATG

AAAAAGATTTCAAACTGTAAGCAAATCCATCTCAGTACAAAATCCCTTGCAATAGATTAT

CCTGTGGACTTTGTAAAGTCAATATCTTGCCAGATCTGTGAACACATCCTGTCCGATCCA

GTAGAAACAACATGTAAGCACTTATTCTGTAGAGTCTGCATTCTTAAATGCCTTAAAGTA

ATGGGAAGCTATTGCCCATCCTGCCGATATCCTTGCTTTTCTACTGATCTAGTGAGTCCT

GTAAAATCCTTCCTAAACATCCTCAACAATTTGGTTTTGAGATGCCCAATAAAAGGCTGT

CATGAGGATGTCTTTTTGGAAAAATACTGCCAACATCGTTCTAATCATAAAGAGGCAGAA

AGC

>MZ712342_LSUHC9098_[Asthenodipsas_lasgalenensis]

GACAGCCACCAGGCTAACTCTGTTAAAGAAAATGCAACAGGTTGTCTGGACATCACTGAT

GAGCAACCTAAGGCAGCAGCTGTAATGTCACAAGTGCCCTTTGTAACAGGCATCGAGATG

AATAAAAATAGCTGGGCAAGAGAGAAAGATGTCTTTCACATGAGCCAAAGGGAGGTGGAA

GCTCACCAAGCAAACCTGCAGTGCCTCTGTCGCATCTGTGGAGACTCACTGAAAACTGAT

CCTTGCAAACAGTGTCATCCAGTCCACGGGCCAGTGGATGAGGAGACCCAGGCTCTTTTG

AGAAAGAAAGAGAAAAGAGCCACTTCTTGGCCAGATCTTCTTGCCAAAGTTTTTAAGATT

GATGTAAGGGAAGATATTGACACAACCCATCCTACTCGTTTTTGTCACAATTGTTGGAAA

GTGATTCAAAGAAAAGTCAGCAATGCTCCCCATGAAGTACATCTTCTAGGGAAAGATCCA

GTGGAGTGGCAGTCCCATTCAACAAGCTGTGATGTTTGTGTCACTTCCTTTTGTGGAGTC

AAGAGAAAGAAACCAATGCTGAATTCACAGCTGAGCAAAAAACTCAGGATTATTGCTGGA

CGTACGAGAAAAATAAAATGCATAAGGAAGATAAAGCAACTGAAAAACAAAAGTTTAATG

AAAAAGATTTCAAACTGTAAGCAAATTCATCTCAGTATAAAAGCCCTTGCAATAGATTAT

CCTGTGGACTTTGTAAAGTCAATCTCTTGCCAGATCTGTGAACACATCCTGTCTGATCCA

GTAGAAACAACATGTAAACACTTATTCTGTAGAGTATGCATTCTTAAATGTCTTAAAGTA

ATGGGAAGCTATTGCCCATCCTGCCAGTATCCTTGCTTTCCTACTGATCTAGTGAGCCCT

GTAAAATCCTTCCTAAACATCCTTAACAATTTGGTTTTGAGATGCCCAATAAAAGGCTGT

CATGAGGAGGTCTTTTTGGAAAAATACTGCCAACATCGTTCTAATCATAAAGACGCAGAA

AGC

>MZ712343_Avpt_[Asthenodipsas_vertebralis]

GACAGCCACCAGGCTAACTCTGTTAAAGAAAATGCAACAGGTTGTCTGGACATCACTGAT

GAGCAACCTAAGGCAGCAGCTGTAATGTCACAAGTGCCCTTTGTAACAGGCATCGAGATG

AATAAAAATAGCTGGGCAAGAGAGAAAGATGTCTTTCACATGAGCCAAAGGGAGGTGGAA

GCTCACCAAGCAAACCTGCAGTGCCTCTGTCGCATCTGTGGAGACTCACTGAAAACTGAT

CCTTGCAAACAGTGTCATCCAGTCCACGGGCCAGTGGATGAGGAGACCCAGGCTCTTTTG

AGAAAGAAAGAGAAAAGAGCCACTTCTTGGCCAGATCTTCTTGCCAAAGTTTTTAAGATT

GATGTAAGGGAAGATATTGACACAACCCATCCTACTCGTTTTTGTCACAATTGTTGGAAA

GTGATTCAAAGAAAAGTCAGCAATGCTCCCCATGAAGTACATCTTCTAGGGAAAGATCCA

GTGGAGTGGCAGTCCCATTCAACAAGCTGTGATGTTTGTGTCACTTCCTTTTGTGGAGTC

AAGAGAAAGAAACCAATGCTGAATTCACAGCTGAGCAAAAAACTCAGGATTATTGCTGGA

CGTACGAGAAAAATAAAATGCATAAGGAAGATAAAGCAACTGAAAAACAAAAGTTTAATG

AAAAAGATTTCAAACTGTAAGCAAATTCATCTCAGTATAAAAGCCCTTGCAATAGATTAT

CCTGTGGACTTTGTAAAGTCAATCTCTTGCCAGATCTGTGAACACATCCTGTCTGATCCA

GTAGAAACAACATGTAAACACTTATTCTGTAGAGTATGCATTCTTAAATGTCTTAAAGTA

ATGGGAAGCTATTGCCCATCCTGCCAGTATCCTTGCTTTCCTACTGATCTAGTGAGCCCT

GTAAAATCCTTCCTAAACATCCTTAACAATTTGGTTTTGAGATGCCCAATAAAAGGCTGT

CATGAGGAGGTCTTTTTGGAAAAATACTGCCAACATCGTTCTAATCATAAAGACGCAGAA

AGC
