# Supplementary figures and images for: An integrative taxonomic revision of slug-eating snakes (Squamata: Pareidae: Pareineae) reveals unprecedented diversity in Indochina
Source: PeerJ. 2022 Jan 10;10:e12713. doi: 10.7717/peerj.12713 (PMC8757378; doi:10.7717/peerj.12713)

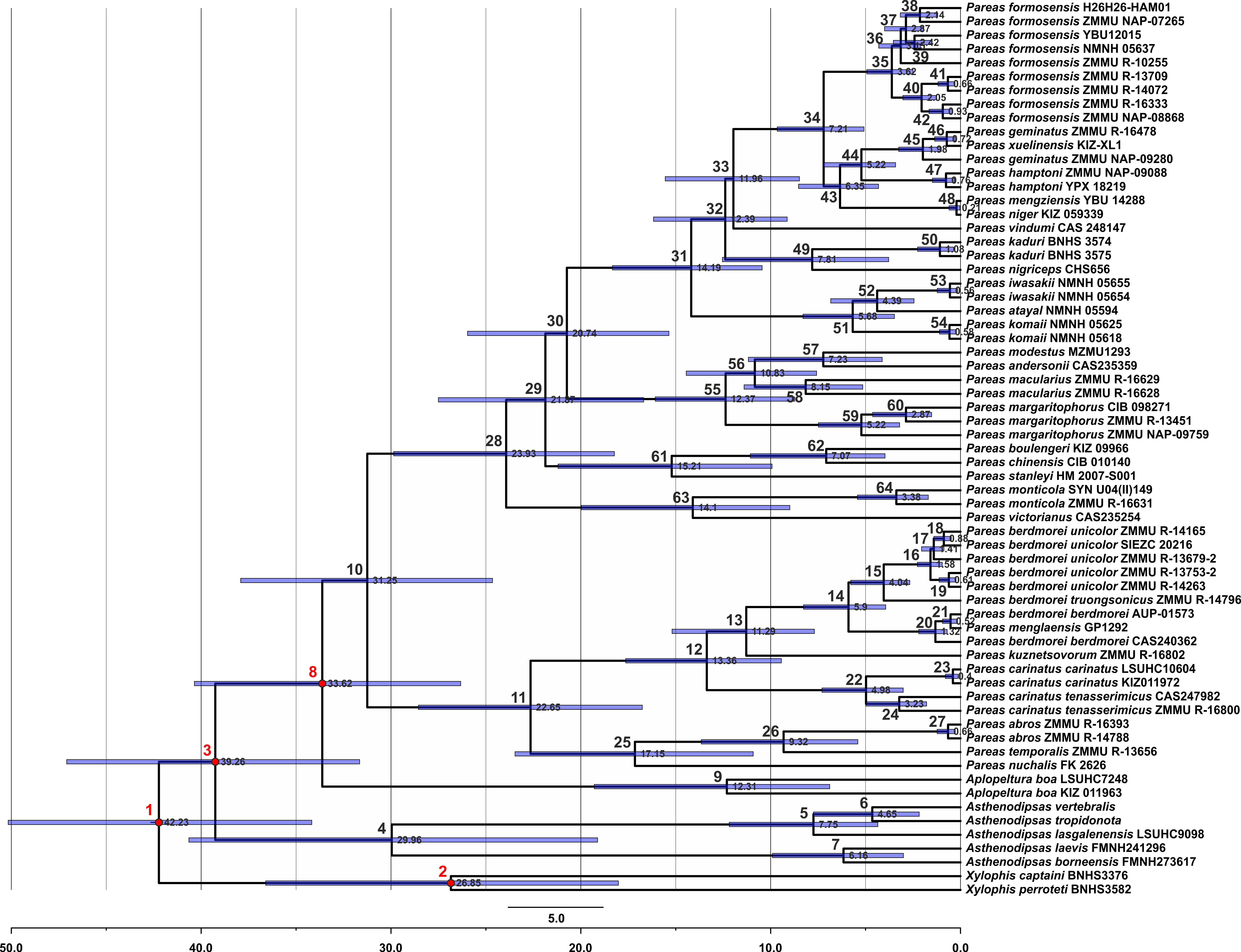

Supplement: Supplemental Information 20 — Node values in bold correspond to node numbers; node values given in smaller font correspond to estimated mean divergence times (in mya), for details see Table S9. Red circles correspond to calibration points used in molecular dating analysis, for details see Table S4. Blue bars correspond to 95%-confidence intervals. [file peerj-10-12713-s020.png]

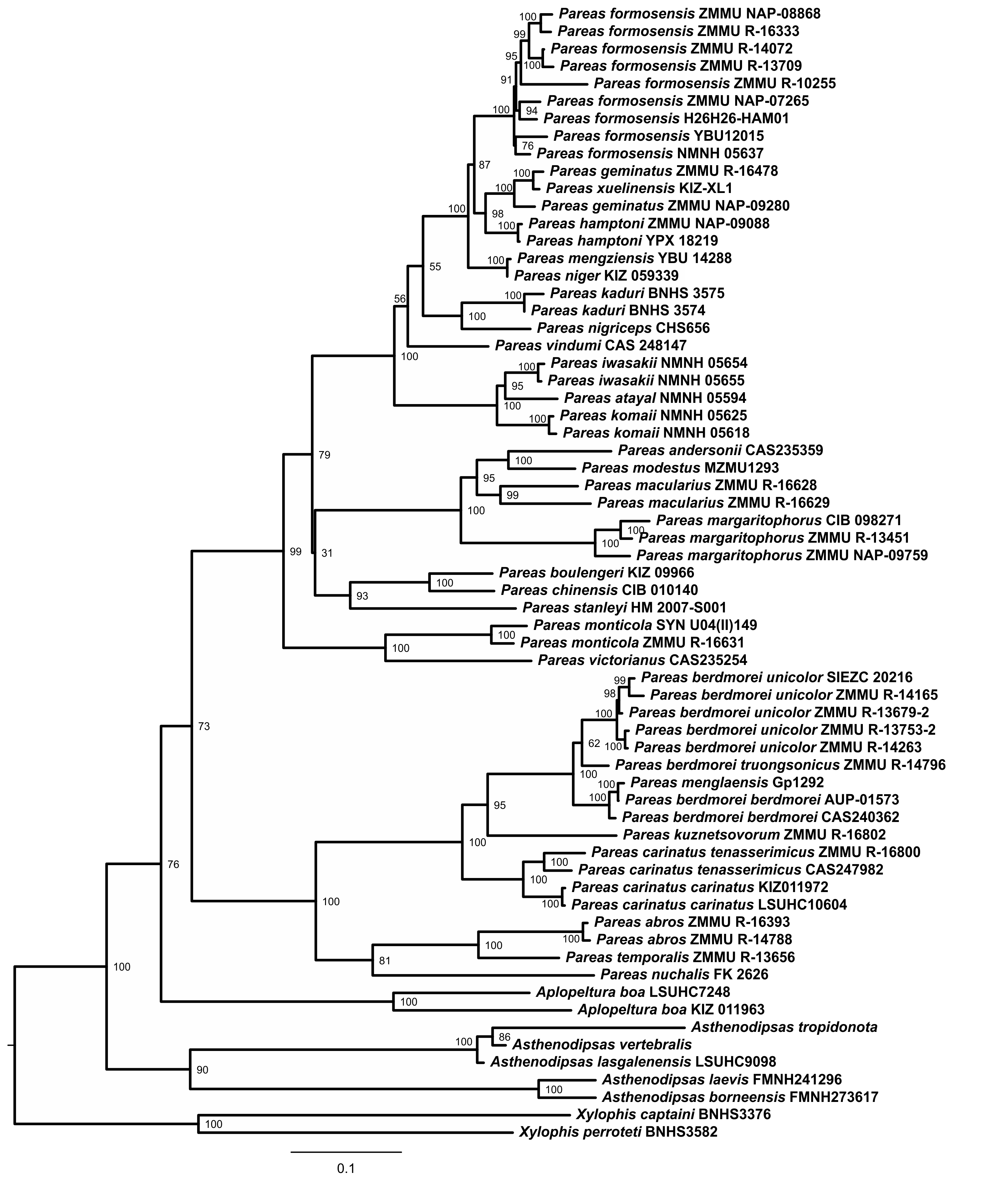

Supplement: Supplemental Information 21 — For voucher specimen information and GenBank accession numbers see Table S1. Numbers at tree nodes correspond to UFBS support values. [file peerj-10-12713-s021.png]

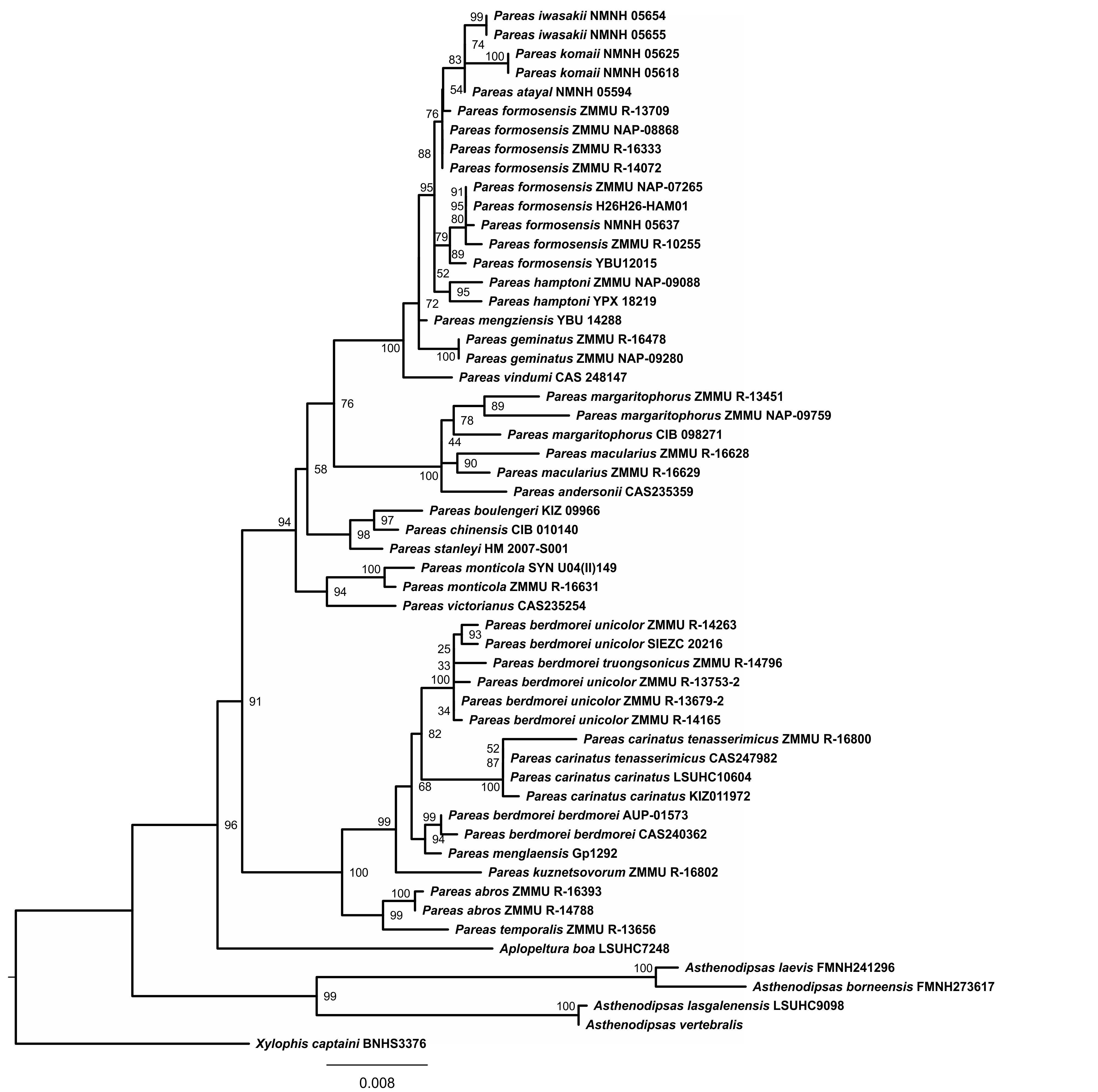

Supplement: Supplemental Information 22 — For voucher specimen information and GenBank accession numbers see Table S1. Numbers at tree nodes correspond to UFBS support values. [file peerj-10-12713-s022.png]
